# Metabolomic and transcriptomic profiling of hepatocellular carcinomas in Hras12V transgenic mice

**DOI:** 10.1002/cam4.1177

**Published:** 2017-09-21

**Authors:** Tingting Fan, Zhuona Rong, Jianyi Dong, Juan Li, Kangwei Wang, Xinxin Wang, Huiling Li, Jun Chen, Fujin Wang, Jingyu Wang, Aiguo Wang

**Affiliations:** ^1^ Laboratory animal center Dalian medical University Dalian Liaoning 116044 China

**Keywords:** Hepatocellular carcinoma, metabolomics, *Ras* oncogene, transcriptomics

## Abstract

Activation of the Ras/MAPK pathway is prevalently involved in the occurrence and development of hepatocellular carcinoma (HCC). However, its effects on the deregulated cellular metabolic processes involved in HCC *in vivo* remain unknown. In this study, a mouse model of HCC induced by hepatocyte‐specific expression of the *Hras12V* oncogene was investigated using an integrative analysis of metabolomics and transcriptomics data. Consistent with the phenotype of abundant lipid droplets in HCC, the lipid biosynthesis in HCC was significantly enhanced by (1) a sufficient supply of acetyl‐CoA from enhanced glycolysis and citrate shuttle activity; (2) a sufficient supply of NADPH from enhanced pentose phosphate pathway (PPP) activity; (3) upregulation of key enzymes associated with lipid biosynthesis; and (4) downregulation of key enzymes associated with bile acid biosynthesis. In addition, glutathione (GSH) was significantly elevated, which may result from a sufficient supply of 5‐oxoproline and L‐glutamate as well as an enhanced reduction in the process of GSSG being turned into GSH by NADPH. The high level of GSH along with elevated *Bcl2* and *Ucp2* expression may contribute to a normal level of reactive oxygen species (ROS) in HCC. In conclusion, our results suggest that the lipid metabolism, glycolysis, PPP, tricarboxylic acid (TCA) cycle, citrate shuttle activity, bile acid synthesis, and redox homeostasis in the HCC induced by *ras* oncogene are significantly perturbed, and these altered metabolic processes may play crucial roles in the carcinogenesis, development, and pathological characteristics of HCC.

## Introduction

Hepatocellular carcinoma (HCC) is a heterogeneous cancer with no promising treatments. It remains one of the most prevalent and lethal malignancies in the world. Approximately 600,000 new cases are diagnosed each year, among which 55% of cases have been identified in China, and an increasing incidence has been observed in Western countries 1. The late diagnosis and consequently poor survival associated with the disease are often attributed to a lack of pathogenomic symptoms and the limitations of diagnostic modalities. Improving both the understanding of HCC etiology and biological processes and the early detection of the disease is an important first step toward the design of effective prevention strategies aimed at early diagnosis and the reduction in HCC.

Ras is a small signal‐transducing guanosine triphosphatase that plays a central role in the control of cell growth and differentiation 2. Activated mutations in *ras* have been found in all human tumors, and the frequency of *ras* mutations is the highest among the genes associated with human cancers 3. In the occurrence and development of human HCC, although the mutational activation of Ras protein occurs with an incidence of 5%, hyperactivation of the Ras/MAPK pathway is a frequent event 4. Consistently, murine HCCs also express H‐*ras*, which is activated in 70% of cases. This evidence supports the view that the Ras pathway plays crucial roles in hepatocarcinogenesis 5. Because animal models are useful tools for dissecting etiological factors and understanding the process of hepatocarcinogenesis, we established Hras12V transgenic (Ras‐Tg) mice lineages in which a hepatocyte‐specific expressed *ras* oncogene under the control of a mouse albumin enhancer/promoter could induce multicentric spontaneous hepatic tumorigenesis at the appropriate time and with a high level of reproducibility and male prevalence 5. The histopathological characteristics of this disease model are that a nontumor liver shows rare inflammation, no signs of fibrosis or cirrhosis, and significantly higher levels of apoptosis and cell cycle arrest. Altered foci (typically < 2 mm) include the aggregation of atypical hepatocytes with basophilic cytoplasm and hyperchromatic nuclei. Phenotypes of hepatic adenoma are similar to altered foci but with a larger size (typically 2–5 mm) and are relatively well circumscribed; HCCs are large in size (typically >5 mm), have a trabecular arrangement of tumor cells, and have highly anaplastic cells with evidence of necrosis 5. Specifically, these hepatic alterations are in dominant PCNA‐positive cells with abundant droplets but have rare apoptotic signs 5. Moreover, the hyperactivation of the MEK/ERK and PI3K/AKT/mTOR signaling pathways was a marked molecular event in HCC 6. Reports based on the Ras‐Tg mouse demonstrate that it is a valuable model to investigate molecular mechanisms, diagnosis and therapeutic strategies, and biomarkers of hepatocarcinogenesis 5, 6, 7, 8, 9, 10, 11, 12, 13, 14, 15, 16, 17, 18. For example, by exploring Ras‐Tg mice, we found that differences in the molecular responses to the deregulated Ras oncoprotein between females and males determine the onset of HCC, which may contribute to the striking male prevalence of HCC 5, 6. In addition, we and other researchers found that B lymphocytes, FoxM1, and miR‐221 play crucial roles in HCC by using Ras‐Tg mice 7, 9, 12.

Molecular profiling approaches, such as metabolomics and transcriptomics to monitor pathological processes of disease, have received a great deal of attention. Metabolomic strategies play an increasingly important role in clinical and observational studies, and they will offer new perspectives not only in understanding the processes of disease development but also in the identification of diagnostic/prognostic markers and targeted healthcare 19. Global gene expression studies using RNA‐Seq can provide insights into regulatory genes and critical pathways that might lead to HCC 20. The integrative analysis of metabolomics and transcriptomics data has the potential to greatly increase our understanding of metabolic networks and biological systems, leading to various potential clinical applications 21. Recently, several studies integrating metabolomics and transcriptomics to uncover the biological processes of tumors have been reported and have proven the valuable and powerful applications of this approach 22. However, an analysis of HCC using this approach has rarely been reported.

In this study, we characterized the metabolite profiles of HCC in Ras‐Tg mice by GC‐TOF‐MS analysis to identify the significantly changed metabolic pathways in HCC induced by the *ras* oncogene. In addition, metabolomic analysis was integrated with transcriptomic analysis of HCC by next‐generation sequencing (NGS) to obtain a better understanding of the metabolic profiles in HCC.

## Materials and Methods

### Experimental animals, sampling, and histopathological examination

The aim of this study is to investigate the metabolomic and transcriptomic differences between HCC and wild‐type liver tissues. Because Ras‐Tg males develop HCC at a relatively early stage (8–9 months of age) with a high incidence (almost 100%) compared to females (over 15 months of age and 30% incidence) 5, 6, males were chosen for this study. Experimental animals and histopathological examination procedures for animal handling and tissue sampling were conducted in compliance with protocols approved by the Animal Care and Use Committee of Dalian Medical University. Ras‐Tg mice (C57BL/6J genetic background), which are an animal model of hepatic tumors induced by an *Hras12V* oncogene specifically expressed in hepatocytes 5, and wild‐type mice (C57BL/6J) were bred and housed in the Laboratory Animal Center of Dalian Medical University. Nine‐month‐old male mice were selected for this study. Parts of the hepatic tumor (over 8 mm in diameter) or wild‐type liver tissues of sacrificed mice were removed, rinsed in cold saline, cut into ~ 1 cm^3^ tissue blocks, and immediately flash frozen in liquid nitrogen. The remaining tissue parts were fixed in 10% neutral buffered formalin, embedded in paraffin, sectioned, and stained with hematoxylin and eosin using standard methods. The histopathology was assessed, and the tissues with a confirmed morphological diagnosis were used for subsequent experimental procedures.

### Sample preparation for GC‐MS analysis

The histopathologically identified HCC from 9‐month‐old Ras‐Tg males and liver tissues from 9‐month‐old wild‐type C57BL/6J males (eight individual samples for each group, for a total of over 32 slices with at least 2 per individual, identified by a licensed pathologist) were subjected to two derivatization steps for GC‐MS analysis. Approximately 0.05 mg of each sample was collected in 2‐mL EP tubes and extracted with 0.4 mL methanol–chloroform (V methanol:V chloroform = 3:1). Then, 20 *μ*L of L‐2‐chlorophenylalanine (1 mg/mL stock in dH_2_O) was added as an internal standard, and the sample was homogenized in a ball mill for 3 min at 65 Hz. After the samples were centrifuged, the supernatant was transferred to a 2‐mL GC‐MS glass vial. An equal volume of 14 *μ*L was taken from each sample and placed into the 2‐mL GC‐MS glass vial as a mixed sample for quality control. The extracts were dried in a vacuum concentrator without heating at 37°C for approximately 2 h. Then, 80 *μ*L of methoxylamine hydrochloride (20 mg/mL) was added to the dried metabolites and incubated at 80°C for 20 min in an oven after mixing and sealing. The lid was opened, and 100 *μ*L of Bis(trimethylsilyl) trifluoroacetamide (BSTFA) (containing 1% trimethylchlorosilane (TMCS), v/v) was added to each sample, which was sealed again and incubated at 70°C for an hour. Then, 5 *μ*L of FAMEs (a standard mixture of fatty acid methyl esters, C8‐C16: 1 mg/mL; C18‐C24: 0.5 mg/mL in chloroform) was added to the mixed sample, cooled to room temperature, and mixed well for GC‐MS analysis.

### GC‐TOF‐MS analysis

GC‐TOF‐MS analysis was performed using an Agilent 7890 gas chromatograph system coupled with a Pegasus HT time‐of‐flight mass spectrometer. The system utilized an Rxi‐5Sil MS column (30 m × 250 *μ*m inner diameter, 0.25 *μ*m film thickness; Restek, USA). A 1‐*μ*L aliquot of the analyte was injected in splitless mode. Helium was used as the carrier gas, the front inlet purge flow was 3 mL/min, and the gas flow rate through the column was 20 mL/min. The initial temperature was 50°C for 1 min, then it was increased to 330°C at a rate of 10°C/min, and then maintained for 5 min at 330°C. The injection, transfer line, and ion source temperatures were 280, 280, and 250°C, respectively. The energy was −70 eV in electron impact mode. The mass spectrometry data were acquired in full‐scan mode with an m/z range 30–600 at a rate of 20 spectra per second after a solvent delay of 366 sec.

### Analysis of GC‐MS data

First, the chroma TOF4.3X software of the LECO Corporation and LECO‐Fiehn Rtx5 database were used for the extraction of raw peaks, the filtering of data baselines, and the calibration of the baseline, peak alignment, deconvolution analysis, peak identification, and integration of the peak area. The RI (retention time index) method was used in peak identification, and the RI tolerance was 5000. Then, identified GC‐MS data were imported into LineUp (Infometrix, Bothell, WA) and PiroTrans (GL Science) to align the chromatograms based on the peak intensity and the retention time of the internal standard, 2‐hydroxyundecanoic acid. The generated peak lists were imported into Pirouette software (Infometrix, Woodinville, WA) for multivariate statistical analysis. The following composition analysis of the samples was performed using MetaboAnalyst version 3.0 (http://www.metaboanalyst.ca/). The analysis methods included Principal Component Analysis (PCA), Partial Least Squares‐Discriminant Analysis (PLS‐DA), Orthogonal Projections to Latent Structures‐Discriminant Analysis (OPLS‐DA), and model validation.

### Metabolite set enrichment

Metabolite set enrichment was investigated using metabolite set enrichment analysis (MSEA) and the overrepresentation analysis (ORA) tools offered by MetaboAnalyst version 3.0. Briefly, all metabolites from the GS‐MS array dataset were entered into MSEA as a list of metabolites. The mouse pathway library was chosen, and the ORA algorithm was selected using a hypergeometric test. The pathway ORA tool then used the proportion of differentially expressed metabolites in the whole array to determine whether a particular pathway was significant. A list of pathways associated with the uploaded metabolites was produced. *P *<* *0.05 was considered to represent significant differences.

### Different gene expression analysis by NGS

Different gene expression was detected using NGS analysis. HCC tissues from 9‐month‐old Ras‐Tg mice and wild‐type liver tissues from 9‐month‐old wild‐type C57BL/6J mice (5 for each group) were chosen for total RNA extraction using TRIzol reagent (Invitrogen, Grand Island, NY). Due to the consistent inbred genetic background, the definite etiology (*ras* oncogene) for HCC development, and the consistent pathological features of the samples in each group, the total RNA samples from the same group were mixed equivalently to generate two composite samples, that is, HCC and wild‐type liver tissues. Preparation of the mRNA sample for RNA‐Seq analysis was performed using the TruSeq RNA LT Sample Prep Kit v2 (Illumina, San Diego, CA). The template molecules were used for cluster generation and sequencing on an Illumina HiSeq 2000 (Illumina, San Diego, CA) instrument. One sample per lane was used to generate 100‐bp paired‐end reads. Read alignment to the UCSC Mouse Reference Genome (mm10, http://genome.ucsc.edu/) was performed using Tophat v1.3.2. Transcript assembly and quantification were performed by Cufflinks v2.0.2 to process the Tophat alignments. The assembled Cufflinks transcripts were processed with Cuffcompare analytical strategies to detect differentially expressed genes. The database for annotation, visualization, and integrated discovery (DAVID) v6.7 was used to interpret the differentially expressed gene pools and KEGG pathway enrichment assay.

### Metabolite detection

*NADPH*: The level of NADPH was detected using a NADP/NADPH Quantification Colorimetric Kit (Cat No. k347‐100; BioVision, Milpitas, USA) according to the instructions of the manufacturer. Briefly, the frozen sample (~50 mg) was washed with ice‐cold 1 × PBS and homogenized in 0.5 mL of NADPH extraction buffer. After keeping the samples on ice for 10 min, the homogenate supernatant was obtained by centrifugation. Two‐hundred microliters of supernatant was transferred into an Eppendorf tube and heated at 60°C for 30 min in a water bath. Then, a 50‐*μ*L aliquot was added to the labeled 96‐well plate in duplicate, and the NADPH level was detected by following the instructions for the kit.

#### Triglyceride and cholesterol

The frozen samples (~50 mg) were used for lipid extraction. Each frozen tissue was added to a 10‐mL heptane–isopropanol–tween mixture (3:2:0.01 by volume) and homogenized. This homogenate was centrifuged, and the supernatants were collected and evaporated with nitrogen flow. The triglyceride and cholesterol contents were determined using commercial kits (Cat No. A0‐10017 and A0‐10027; Dongou Biological Engineering Co Ltd, Wenzhou, China). All samples were measured in duplicate.

#### Pyruvate, lactate, and bile acid

The frozen samples (~50 mg) were added to 0.5 mL of 1 × PBS (0°C, pH 7.2–7.4) and homogenized on ice. The homogenate supernatant was obtained by centrifugation, and the levels of pyruvate, lactate, and bile acid were detected using commercial kits according to the instructions of the manufacturer (Cat No. A081, A019‐2, and E003‐2; Nanjing Jiancheng Bioengineering Institute, Nanjing, China). All samples were measured in duplicate.

#### GSH

Fresh samples (~50 mg) were added to 1 mL of ice‐cold 1 × PBS and homogenized with a glass homogenizer on ice. The resulting suspension was sonicated with an ultrasonic cell disrupter until the solution was clarified. Then, the homogenates were centrifuged for 5 min at 10,000×*g*. The supernatant was collected and detected immediately using an enzyme‐linked immunosorbent assay kit according to the instructions of the manufacturer (Cat No. CEA294Ge; Cloud‐Clone Corp, Wuhan, China). All samples were measured in duplicate.

#### ROS

The mouse reactive oxygen species (ROS) ELISA Kit (BH8443, BOYAO Biotechnology, Shanghai, China) was used to detect the ROS levels, and the procedures were performed according to the instructions of the manufacturer. Briefly, the fresh tissue samples were cut, weighed, and placed into a grinding tube containing ceramic beads and 1 mL of pH 7.4 PBS. Then, the tissue was homogenized and centrifuged, and the supernatant was carefully collected and assayed with an ELISA Kit. The final ROS level was calculated by dividing the total ROS content by the original tissue weight.

### Statistical analysis

The peak intensities of metabolites and the signal values of gene expression were statistically analyzed by a Student's *t*‐test between HCC and wild‐type liver tissues. All the data were presented as the mean ($\overset{¯}{X}$), with a level of probability of 0.05 as the criterion for significance. Fold change is a measure describing how much a quantity changes going from the initial to the final value, and we recorded it as a log value.

## Results

### Gross and histological identification

**Figure 1:**
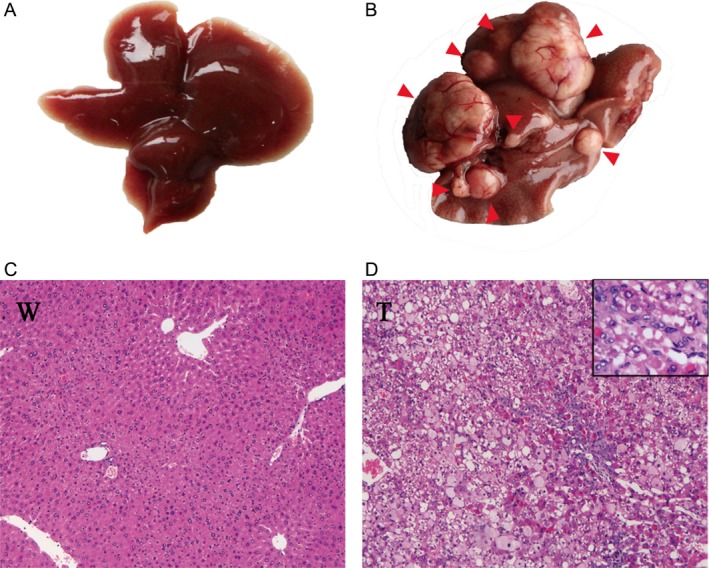
Gross anatomic and histopathological analysis of hepatic alterations. (A) Representative liver stereogram image of a liver from a 9‐month‐old wild‐type male. (B) Representative liver stereogram image of a liver carrying multiple tumors from a 9‐month‐old Ras‐Tg male. The red arrows indicate the hepatic alterations. (C) Relative histopathological H&E staining images (100 × ) showing liver tissue from a wild‐type mouse. (D) Relative histopathological H&E staining images (100 × ; upright corner: 400 × ) showing HCC from a Ras‐Tg mouse. W: wild‐type liver tissue; T: hepatocellular carcinoma (HCC).

To investigate the common metabolomics and transcriptomics related to HCC induced by the *ras* oncogene, Ras‐Tg and wild‐type males were sacrificed at 9 months of age, and the hepatic tumors and wild‐type liver tissues were sampled. The gross and histological findings were the same as our previous reports 5. The hepatic tumors were well developed with visible yellowish‐whitish lesions that were round or oval with enriched blood vessels, had soft and fragile qualities, and were of various sizes. The lesions were protruding onto or embedded in the livers of Ras‐Tg males (Fig. 1B). In contrast, the liver tissues from wild‐type males were bright red in color and flexible in texture with no lesions (Fig. 1A). Histopathological diagnosis showed that the HCC (typically over 5 mm in diameter) had a trabecular arrangement of tumor cells, which were highly anaplastic cells with evidence of necrosis and signs of lymphocytic infiltration (Fig. 1D). In contrast, the wild‐type liver cells were arranged neatly with no inflammatory cell infiltration or hepatic necrosis (Fig. 1C). In all of the sampled mice, lesions were not found in any other organs. The histopathologically identified HCC from eight individual Ras‐Tg males, and liver tissues from eight individual wild‐type males were chosen for further analysis.

### GC‐MS chromatograms of HCC and wild‐type liver tissues

Typical GC‐MS total ion current (TIC) chromatograms of HCC and wild‐type liver tissue extracts are shown in Figure S1. The retention time of the internal standard was consistent for each run. The data showed marked differences in the chromatogram patterns between HCC and wild‐type liver tissues.

### Clustering of HCC and wild‐type liver tissue samples

First, the missing values of raw data were calculated by using half of the minimum value; then, 631 peaks were detected, and 76 metabolites were excluded through the interquartile range denoising method. In addition, the internal standard normalization method was employed in this data analysis. The resulting two‐dimensional data involving the peak number, sample name, and normalized peak area were entered into the SIMCA‐P 14.0 software package (Umetrics, Umea, Sweden) for principal component analysis (PCA), partial least squares‐discriminant analysis (PLS‐DA), and orthogonal projections to latent structure‐discriminant analysis (OPLS).

**Figure 2:**
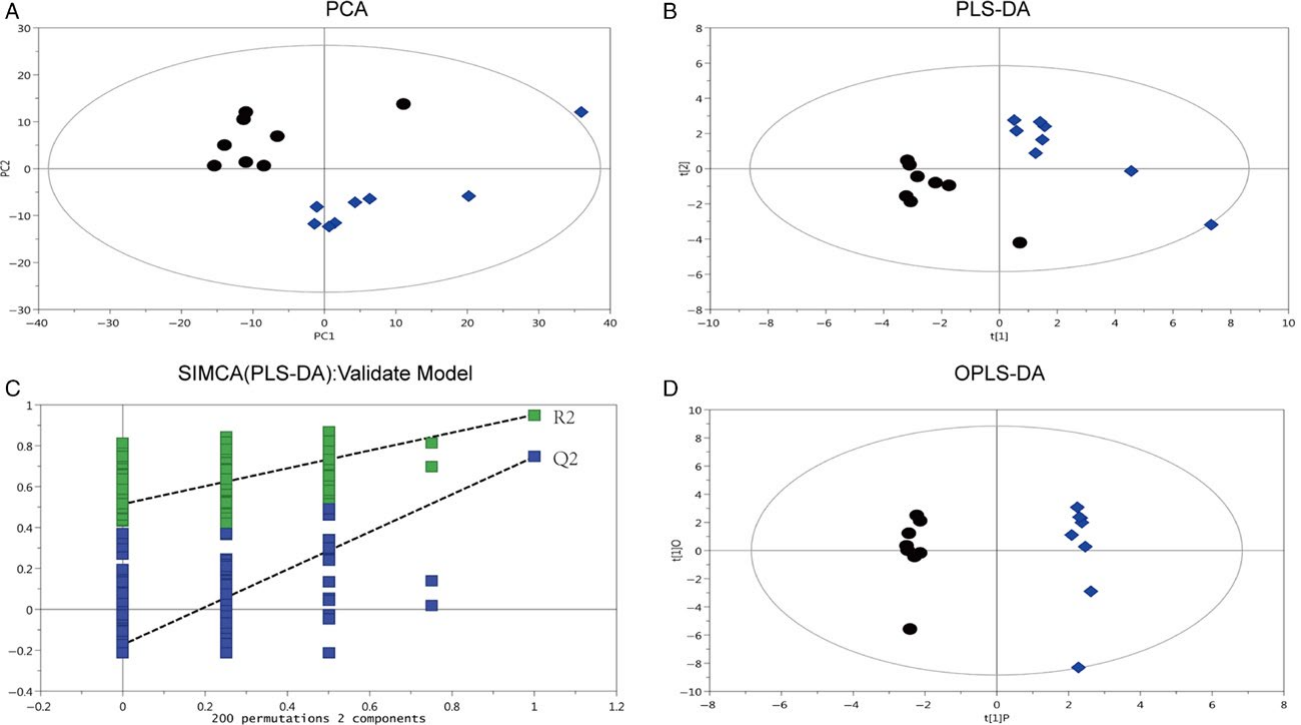
Score plots of PCA, PLS‐DA, and OPLS‐DA based on the metabolite profile data of HCC and wild‐type liver tissues. (A) Score plots of PCA based on the metabolite profile data for wild‐type liver tissues (black circles) and HCC (blue rhombi). The principal components PC1 (t[1]) and PC2 (t[2]) described 33.6% and 15.6% of the variation, respectively (*n* = 8). (B) The plot of PLS‐DA scores showing almost complete separation of wild‐type liver tissues (black circles) and HCC (blue rhombi). The classification parameters were R2X (cum) = 0.539, R2Y (cum) = 0.951, and Q2 (cum) = 0.746. (C) Validation model of PLS‐DA for wild‐type liver tissues (blue squares) and HCC (green squares) for 200 permutations of the data showing the degradation of R2 to below 0.515 and Q2 to below −0.173. (D) OPLS‐DA showing the contribution of variables to the difference between wild‐type liver tissues (black circles) and HCC (blue rhombi). The classification parameters were R2X (cum) = 0.816, R2Y (cum) = 0.996, and Q2 (cum) = 0.874.

PCA is an unsupervised analysis method reflecting the original state of the data. The PCA algorithm generates a single point that represents the metabolites in a sample and their concentrations (each dot in the score plots shown in Fig. 2A represents a single mouse). The close clustering of dots indicates that the samples have similar compositions (classification parameters: R2X (cum) = 0.587; Q2 (cum) = 0.221). The score plots of samples were clustered into two distinct groups (Fig. 2A), which indicates that the profiles of the metabolites in HCC tissues derived from Ras‐Tg males differed from those in liver tissues derived from wild‐type males.

PLS‐DA was applied to obtain a higher level of group separation and produce a better understanding of the variables responsible for classification (Fig. 2B). The classification parameters were R2X (cum) = 0.539; R2Y (cum) = 0.951; and Q2 (cum) = 0.746. Seven‐fold cross‐validation was used to estimate the robustness and the predictive ability of our model, and this permutation test was used to further validate the model. The R2 and Q2 intercept values were 0.515 and −0.173, respectively, after 200 permutations (Fig. 2C). The low values of the Q2 intercept indicate the robustness of the models and therefore show a low risk of overfitting and reliability.

OPLS‐DA was applied to show the contribution of variables to the difference between the two groups (Fig. 2D). The classification parameters were R2X (cum) = 0.816; R2Y (cum) = 0.996; and Q2 (cum) = 0.874. The results also showed important variables that were situated far from the origin, but the loading plot was complex because of many variables. To refine this analysis, the first‐principal component of variable importance projection (VIP) was obtained. The VIP values exceeding 1.0 were first selected as altered metabolites. In step 2, the remaining variables were then assessed by a Student's t‐test (t‐test), *P *>* *0.05, and variables were discarded between the two comparison groups. In addition, commercial databases, including KEGG (http://www.genome.jp/kegg/) and NIST (http://www.nist.gov/index.html), were utilized to search for the metabolite pathways.

### Metabolite differences between HCC and wild‐type liver tissues screened by PCA

In addition to the score plot, which is based on the composition of the samples, the PCA algorithm also creates a loading plot, which is based on metabolite values. Loading plots identify metabolites that contribute to the differential clustering of HCC and wild‐type liver tissues in the score plots. The electron impact spectra of these discriminatory metabolites were then compared with the NIST 05 Mass Spectral Library (NIST). Putatively identified discriminatory metabolites with matching NIST spectra are shown in Table 1. Fold changes and *P*‐values of these metabolites between HCC and wild‐type liver tissues were calculated by the TICs of GC‐MS chromatograms. Overall, the metabolic profiles in HCC and wild‐type liver tissues were different. Among the detected 555 metabolites, 36 common discriminatory metabolites contributed to this difference. However, the array of changes in the individual discriminatory metabolites in HCC and wild‐type liver tissues was more diverse.

**Table 1:** Fold change and relative P‐value of the discriminatory metabolites characterizing hepatic carcinoma and wild‐type liver tissues were calculated by the TICs of GC‐MS chromatograms

Metabolites	Retention time (min)	W mean	T mean	Fold change	*P*‐value
Glycolysis; TCA cycle; Pentose phosphate pathway; Lipid biosynthesis					
Glucose 1‐phosphate (glucose‐1p)	16.7179	1.09E‐01	2.14E‐01	9.73E‐01	2.81E‐03
Adenosine	24.0355	6.81E‐03	4.06E‐03	−7.46E‐01	1.17E‐02
Cytidine diphosphate (CDP)	22.5710	3.47E‐03	1.08E‐03	−1.68E+00	2.81E‐03
Citrate	17.2200	1.00E‐02	4.55E‐02	2.19E+00	3.18E‐02
Cytidine monophosphate (CMP)	19.0875	3.49E‐03	1.46E‐03	−1.26E+00	1.69E‐03
Deoxycytidine monophosphate (dCMP)	21.3202	7.62E‐04	1.82E‐04	−2.07E+00	7.87E‐03
Fructose	17.8171	9.71E‐01	3.30E+00	1.76E+00	2.45E‐02
Fructose 1,6‐bisphosphate (fructose‐1, 6p2)	11.5276	6.02E+00	1.30E+01	1.11E+00	1.65E‐02
Fructose 6‐phosphate (fructose‐6p)	21.2896	3.78E‐03	5.70E‐03	5.93E‐01	4.18E‐02
Glucose 6‐phosphate (glucose‐6p)	21.4070	7.04E‐02	7.72E‐01	3.45E+00	9.13E‐04
Lactate	8.8119	2.20E‐02	7.75E‐03	−1.51E+00	4.97E‐02
Malate	13.8313	2.25E+00	5.46E+00	1.28E+00	4.02E‐02
Oxaloacetate	13.5829	1.22E‐02	2.51E‐02	1.04E+00	4.88E‐02
Pyruvate	18.9793	2.10E‐01	4.21E‐01	1.00E+00	3.40E‐02
Succinate	11.8994	1.40E‐01	6.21E‐02	−1.17E+00	2.41E‐03
Thymine	13.0700	5.41E‐03	1.82E‐02	1.75E+00	4.35E‐03
Uridine diphosphate (UDP)	21.5776	2.73E‐03	6.28E‐03	1.20E+00	1.15E‐02
2‐hydroxybutanoic acid	9.5741	4.89E‐02	2.55E‐02	−9.36E‐01	1.81E‐03
Aspartate	14.2188	1.36E‐01	2.61E‐01	9.40E‐01	4.35E‐02
Fructose 2,6‐bisphosphate (fructose‐2, 6p2)	20.6541	4.81E‐01	3.42E‐01	−4.92E‐01	5.43E‐03
Fucose	16.4754	4.34E‐03	7.03E‐04	−2.63E+00	7.12E‐06
Fumarate	12.2976	1.56E‐01	2.29E‐01	5.54E‐01	5.66E‐04
Xylitol	16.1919	3.77E‐01	1.08E‐01	−1.81E+00	7.84E‐05
Uridine monophosphate (UMP)	24.2838	4.53E‐02	1.26E‐01	1.48E+00	1.97E‐02
Uracil	12.2391	1.57E‐02	7.11E‐02	2.18E+00	2.94E‐03
Uridine	22.6655	7.55E‐04	1.53E‐03	1.02E+00	1.06E‐02
Xanthosine	24.2838	1.07E‐03	1.82E‐04	−2.56E+00	4.95E‐02
*β*‐alanine	13.2484	3.79E‐02	1.86E‐02	−1.03E+00	7.11E‐05
Nicotinamide adenine dinucleotide phosphate (NADP+)	4.7442	4.03E‐01	2.53E‐01	−6.72E‐01	1.22E‐04
Glutathione metabolism					
L‐glutamate	15.2758	6.10E‐03	1.55E‐02	1.35E+00	2.02E‐02
5‐oxoproline	14.4595	3.84E‐01	7.26E‐01	9.19E‐01	1.17E‐02
Glycine	11.8509	2.22E+00	1.63E+00	−4.46E‐01	2.26E‐02
Nicotinamide adenine dinucleotide phosphate (NADP+)	4.7442	4.03E‐01	2.53E‐01	−6.72E‐01	1.22E‐04
Glutathione (GSH)	17.7547	2.23E‐01	2.95E‐01	4.04E‐01	4.52E‐02
*γ*‐aminobutyric acid (GABA)	10.0654	6.19E‐03	1.53E‐02	1.31E+00	4.15E‐02
Succinate	11.8994	1.40E‐01	6.21E‐02	−1.17E+00	2.41E‐03
Cholesterol and bile acid biosynthesis					
Glycine	11.8509	2.22E+00	1.63E+00	−4.46E‐01	2.26E‐02
Cholesterol	10.7894	5.41E‐03	1.82E‐02	1.75E+00	4.35E‐03
Cholate	24.7399	2.30E+00	3.60E‐01	−2.68E+00	2.68E‐04

The different cluster of metabolites identified by PCA analysis between wild‐type liver tissues (*n* = 8) and HCC (*n* = 8) was shown. The metabolites were classified into corresponding pathways analyzed in Figure 4. *P *<* *0.05 means the significant difference. W, wild‐type liver; T, hepatocellular carcinoma (HCC); Fold change: log_2_ (T mean/W mean).

### Transcriptional differences between HCC and wild‐type liver tissues screened by NGS

Transcriptional data for 28225 well‐identified transcripts in HCC and wild‐type liver tissues were analyzed by NGS. Among them, 3747 transcripts were significantly and differentially expressed between the two groups based on *P*‐values (*P *<* *0.05) (Table S1). Values for differentially expressed enzymes involved in the metabolic pathways of glycolysis, TCA cycle, PPP, lipid, glutathione, and cholesterol are shown in Table 2.

**Table 2:** Differently expressed genes identified by Next‐Generation Sequencing (NGS) between HCC and wild‐type liver tissues related to the pathways analyzed in Figure 4

mRNA accession NO.	Gene symbol	Full name	W value	T value	Fold change	*P*‐value
Glycolysis; TCA cycle; Pentose phosphate pathway; Lipid biosynthesis						
NM_133904	Acacb	Acetyl‐Coenzyme A carboxylase beta	5.65E+00	2.03E+01	1.28E+00	1.27E‐02
NM_001199296	Acly	ATP‐citrate synthase isoform 1	1.43E+01	1.23E+02	2.15E+00	2.47E‐02
NM_019477	Acsl4	Long‐chain fatty‐acid–CoA ligase 4 isoform 2	7.41E+00	5.07E+01	1.92E+00	6.63E‐04
NM_028176	Cda	Cytidine deaminase	2.51E+00	1.40E+01	1.72E+00	7.28E‐03
NM_023203	Dctpp1	dCTP pyrophosphatase 1	5.87E+01	2.50E+01	−8.54E‐01	3.99E‐02
NM_007861	Dld	Dihydrolipoyl dehydrogenase	9.25E+01	3.90E+01	−8.64E‐01	4.69E‐02
NM_170778	Dpyd	Dihydropyrimidine dehydrogenase	1.30E+02	2.91E+01	−1.50E+00	3.64E‐03
NM_001164466	Dpys	Dihydropyrimidinase	7.85E+01	9.94E+00	−2.07E+00	1.39E‐07
NM_007988	Fasn	Fatty acid synthase	7.90E+01	2.58E+02	1.18E+00	5.74E‐03
NM_010209	Fh	Fumarate hydratase 1	1.37E+02	5.65E+01	−8.86E‐01	6.23E‐05
NM_008061	G6pc	Glucose‐6‐phosphatase	1.99E+02	1.47E+01	−2.61E+00	2.28E‐09
NM_019468	G6pd2	Glucose‐6‐phosphate 1‐dehydrogenase 2	2.66E‐01	6.06E+00	3.13E+00	2.63E‐03
NM_011829	Impdh1	Inosine‐5′‐monophosphate dehydrogenase 1	8.62E‐01	3.44E+00	1.38E+00	2.29E‐02
NM_008797	Pcx	Pyruvate carboxylase	6.31E+00	5.19E+01	2.11E+00	1.20E‐04
NM_008826	Pfkl	6‐phosphofructokinase	4.00E+00	1.30E+01	1.18E+00	9.30E‐03
NM_001081274	Pgd	6‐phosphogluconate dehydrogenase	2.31E+01	6.10E+01	9.71E‐01	3.58E‐02
NM_011099	Pkm2	Pyruvate kinase isozymes M1/M2	1.09E+01	2.96E+01	9.99E‐01	2.30E‐02
NM_011506	Sucla2	Succinyl‐CoA ligase [ADP‐forming] subunit beta	8.10E+01	3.23E+01	−9.19E‐01	2.64E‐02
NM_133995	Upb1	Beta‐ureidopropionase	2.14E+02	8.74E+01	−8.95E‐01	3.23E‐05
NM_146006	Lss	Lanosterol synthase	1.18E+01	6.96E+01	1.77E+00	1.02E‐03
NM_007856	Dhcr7	7‐dehydrocholesterol reductase	2.68E+01	9.47E+01	1.26E+00	2.58E‐02
NM_138656	Mvd	Mevalonate (diphospho) decarboxylase	3.42E+00	2.57E+01	2.02E+00	6.45E‐05
NM_145927	Fntb	Protein farnesyltransferase subunit beta	1.61E‐01	1.63E+00	2.31E+00	2.82E‐02
Glutathione metabolism						
NM_172961	Abat	4‐aminobutyrate aminotransferase	3.77E+01	1.14E+01	−1.20E+00	3.64E‐03
NM_019468	G6pd2	Glucose‐6‐phosphate 1‐dehydrogenase 2	2.66E‐01	6.06E+00	3.13E+00	2.63E‐03
NM_001081274	Pgd	6‐phosphogluconate dehydrogenase	2.31E+01	6.10E+01	9.71E‐01	3.58E‐02
NM_008160	Gpx1	Glutathione peroxidase 1	1.38E+03	1.28E+02	−2.38E+00	1.35E‐04
NM_177410	Bcl2	B cell leukemia/lymphoma 2	2.16E‐01	1.03E+00	1.56E+00	1.01E‐02
NM_011671	Ucp2	Uncoupling protein 2	3.94E+00	1.89E+01	1.57E+00	1.79E‐04
Cholesterol and bile acid biosynthesis						
NM_145364	Akr1d1	3‐oxo‐5‐beta‐steroid 4‐dehydrogenase	1.13E+02	9.67E+00	−2.46E+00	2.21E‐09
NM_008537	Amacr	Alpha‐methylacyl‐CoA racemase	4.97E+01	2.17E+01	−8.29E‐01	4.15E‐02
NM_007519	Baat	Bile acid‐CoA:amino acid N‐acyltransferase	1.44E+02	3.16E+01	−1.52E+00	3.42E‐04
NM_016668	Bhmt	Betaine–homocysteine S‐methyltransferase 1	1.57E+03	5.90E+01	−3.28E+00	1.29E‐03
NM_024264	Cyp27a1	Sterol 26‐hydroxylase	1.82E+02	2.49E+01	−1.99E+00	3.36E‐06
NM_010010	Cyp46a1	Cholesterol 24‐hydroxylase	1.59E+00	1.28E‐01	−2.52E+00	6.88E‐04
NM_007824	Cyp7a1	Cholesterol 7‐alpha‐monooxygenase	6.62E+01	3.18E+00	−3.04E+00	1.64E‐13
NM_007825	Cyp7b1	25‐hydroxycholesterol 7‐alpha‐hydroxylase	1.74E+02	9.14E+00	−2.95E+00	3.37E‐12
NM_010012	Cyp8b1	7‐alpha‐hydroxycholest‐4‐en‐3‐one	6.44E+01	1.31E+00	−3.90E+00	0.00E+00
NM_028772	Dmgdh	Dimethylglycine dehydrogenase	2.08E+02	2.70E+01	−2.04E+00	2.72E‐05
NM_010321	Gnmt	Glycine N‐methyltransferase	1.44E+03	8.06E+01	−2.88E+00	2.01E‐06
NM_133943	Hsd3b7	3 beta‐hydroxysteroid dehydrogenase type 7	7.31E+01	2.82E+01	−9.53E‐01	1.79E‐02
NM_008952	Pipox	Peroxisomal sarcosine oxidase	1.88E+02	5.13E+01	−1.30E+00	3.65E‐03
NM_146006	Lss	Lanosterol synthase	1.18E+01	6.96E+01	1.77E+00	1.02E‐03
NM_007856	Dhcr7	7‐dehydrocholesterol reductase	2.68E+01	9.47E+01	1.26E+00	2.58E‐02
NM_138656	Mvd	Mevalonate (diphospho) decarboxylase	3.42E+00	2.57E+01	2.02E+00	6.45E‐05
NM_145927	Fntb	Protein farnesyltransferase subunit beta	1.61E‐01	1.63E+00	2.31E+00	2.82E‐02

The mRNA levels of differently expressed genes related to metabolism pathways of glycolysis, TCA cycle, pentose phosphate, lipid, glutathione, and cholesterol are shown. HCC (*n* = 5) and wild‐type liver (*n* = 5) and tissues were analyzed. *P* values were calculated using Student's *t*‐test and *P *<* *0.05 means the significant difference. W, wild‐type liver; T, hepatocellular carcinoma (HCC); Fold change: ln (T mean/W mean).

### Pathway enrichment and metabolite set enrichment analysis

**Figure 3:**
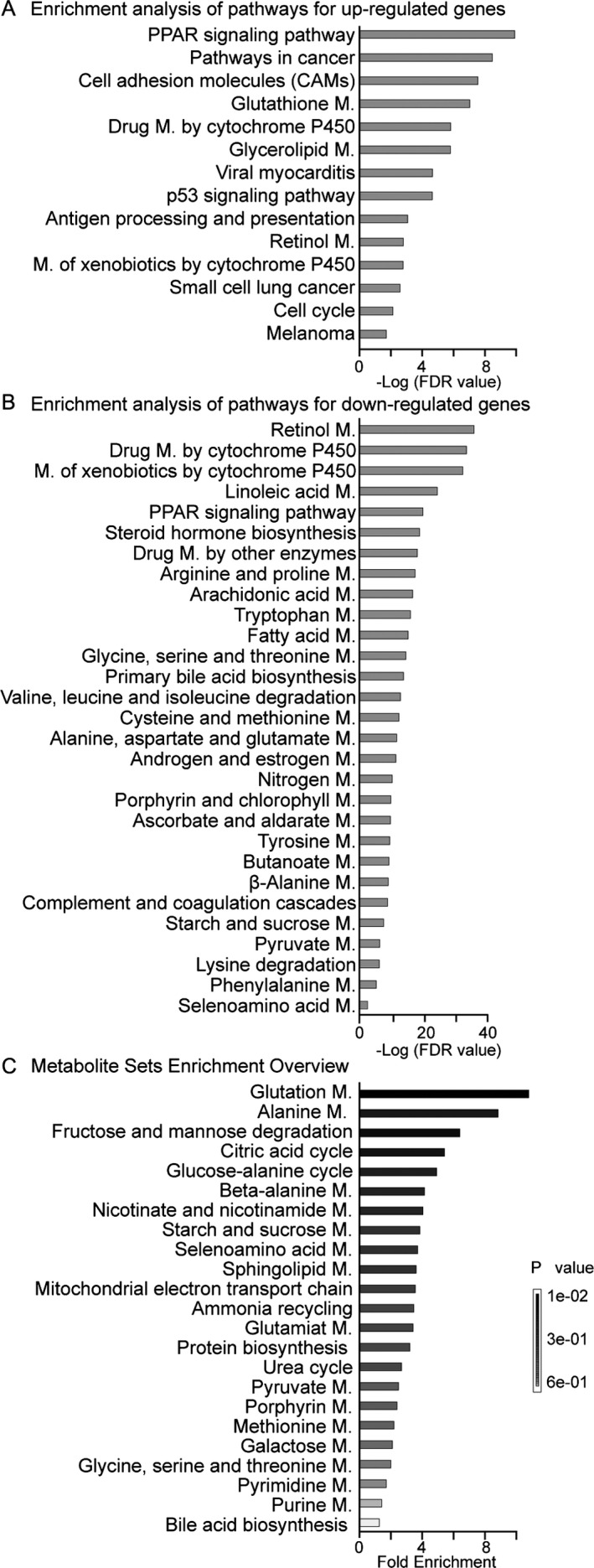
Pathway enrichment analysis for significantly changed genes and metabolites. (A) The significantly upregulated genes and (B) downregulated genes in HCC compared to wild‐type liver tissues were analyzed by KEGG pathway enrichment assays. (C) Significantly changed metabolites in HCC compared to wild‐type liver tissues were analyzed by MetaboAnalyst version 3.0 for metabolite set enrichment. The *p* values for the metabolic pathways are color coded, with dark black representing the most significant values and white representing the least significant values. Detailed information is shown in Tables [Link], [Link], [Link], [Link]. M.: metabolism.

Although we cannot conclusively determine which of the potential metabolic pathways are relevant to a phenotype, KEGG pathway enrichment (Fig. 3A, B; Tables [Link], [Link], [Link]) and metabolite set enrichment analysis (MSEA) (Fig. 3C; Tables [Link], [Link]) identified several key metabolic pathways. The following pathways were selected from the outputs of the KEGG pathway analysis and MSEA based on their biological relevance to HCC.

### Alterations of glycolysis, PPP, TCA cycle, and lipid biosynthesis in HCC

Disordered lipid metabolism has been widely reported in cancer cells. Because we observed a large quantity of lipid droplets in HCC cells based on gross anatomy and histopathological diagnosis (Fig. 1B, D), lipid biosynthesis and its closely related pathways, including glycolysis, PPP, and the TCA cycle, were comprehensively analyzed based on the metabolomics and transcriptomics database.

For the glycolysis metabolism pathway, we found that mainstream metabolites, such as glucose‐1‐phosphate (glucose‐1p), glucose‐6‐phosphate (glucose‐6p), fructose‐6‐phosphase (frucotose‐6p), fructose‐1,6‐bisphosphage (fructose‐1,6p2), and pyruvate, and the related enzymes *Pfk1* and *Pkm2* were significantly increased. In addition, the increased fructose and downregulated G6PC enzyme ensured a sufficient supply of raw materials. This outcome indicates that the enhanced glycolysis may not only maintain the excessive energy need for tumor growth but also provide a large quantity of the final metabolite, pyruvate.

**Figure 4:**
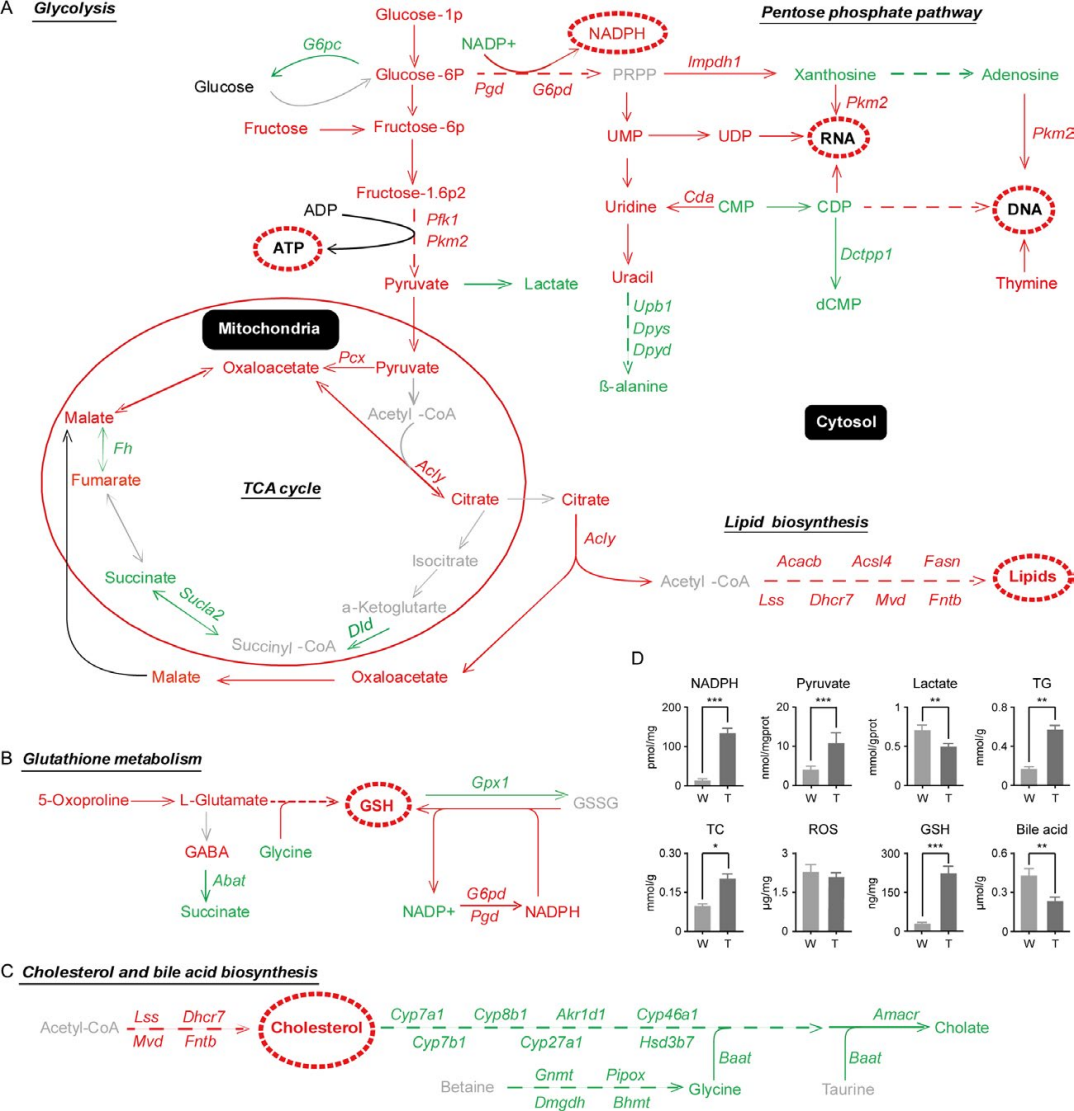
Schematic representations of the most relevant metabolic and transcriptional differences between HCC and wild‐type liver tissues. Metabolic pathways related to glycolysis, the TCA cycle, PPP, and lipid biosynthesis. (B) Metabolic pathways related to glutathione. (C) Metabolic pathways related to cholesterol and bile acid synthesis. Red indicates significantly higher concentrations of metabolites, expression levels of enzymes, or enhanced pathways in HCC; green indicates significantly lower concentrations of metabolites, expression levels of enzymes, or attenuated pathways in HCC; and gray indicates unchanged or undetermined results. The standard letters indicate metabolites, the italic letters indicate enzymes, and the underlined italic letters indicate pathways. The metabolites with broken circles indicate the central metabolites in HCC. (D) The reduced form of nicotinamide adenine dinucleotide phosphate (NADPH), pyruvate, lactate, cholesterol (TC), triglyceride (TG), ROS, GSH, and bile acid (BA) was detected in wild‐type liver (W) and HCC (T) tissues using the methods described in the Materials and Methods section. The data are expressed as the mean ± SEM (*n* = 8). *P <* *0.05, **P <* *0.01, and ***P <* *0.001.

The pyruvate from the glycolysis pathway enters the mitochondria and participates in the TCA cycle. Interestingly, in the TCA cycle, parts of metabolites, such as malate, oxaloacetate, and citrate, and the related enzymes *Pcx* and *Acly* were significantly increased, while parts of other metabolites, such as succinate, and the related enzymes *Dld*,* Fh*, and *Sucl* were significantly decreased. The results indicate that the shuttling of citrate and malate across the membrane of mitochondria may be enhanced, and therefore, the flow of pyruvate to lipid biosynthesis is enhanced by combining TCA and the citrate shuttle, in which pyruvate is taken from the cytosol, converted into acetyl‐CoA, condensed with oxaloacetate to citrate, and transferred back to the cytosol (Fig. 4A, Tables 1, 2).

Another main raw material, hydrogen (H), is dominantly supplied by NADPH from the pentose phosphate pathway (PPP). In PPP, the levels of enzymes *Pgd* and *G6pd* and the concentration of NADPH were significantly elevated. Following the PPP, metabolites such as UMP, UDP, uridine, uracil, and thymine were significantly increased, while xanthosine, adenosine, CMP, CDP, beta‐alanine, and dCMP were significantly decreased. In addition, related enzymes, such as *Impdh1*,* Pkm2*, and *Cda,* were significantly increased, while *Upb1*,* Dpys*,* Dpyd*, and *Dctpp1* were significantly decreased (Fig. 4A; Tables 1, 2). This outcome indicates that the enhanced PPP may supply not only the NADPH but also the raw materials for nucleotide synthesis needed for the rapid growth of tumor cells.

Finally, the enhanced glycolysis pathway and the citrate shuttle offered sufficient acetyl‐CoA, and the enhanced PPP offered sufficient NADPH. Additionally, increased expression levels of enzymes such as *Acacb*,* Acsl*,* Fasn*,* Lss*,* Dhcr7*,* Mvd*, and *Fntb* stimulated lipid synthesis and the accumulation of lipids to form large quantities of lipid droplets in HCC cells (Fig. 4A; Tables 1, 2; Fig. 1B, D).

### Alterations of the glutathione metabolism pathway in HCC

Glutathione (GSH), especially in hepatocytes, plays important roles not only as an antioxidant but also in integrated detoxification. The increase in GSX (GSH+GSSG) in cancer cells indicates that enhanced de novo glutathione synthesis is occurring, which is crucial in cancer biology 23. In this study, GSH and the substrates for its synthesis, such as 5‐oxoproline and L‐glutamate, were significantly increased in HCC compared to wild‐type liver tissues (Table 1). The accumulated GABA resulted from significantly downregulated expression of *Abat* enzymes and subsequently decreased succinate, which may contribute to a sufficient L‐glutamate supply (Tables 1, 2). However, glycine, which is a widely used substrate for synthesis in cells, was significantly decreased, which may be due to its excessive consumption (Table 1). In addition, the significantly increased NADPH resulted from enhanced PPP via significant upregulation of the *Pgd* and *G6pd* enzymes, which may contribute to the enhanced reduction processes from GSSG to GSH (Tables 1, 2). The decreased *Gpx1* level may attenuate the oxidation processes from GSH to GSSG. These changes may play crucial roles in maintaining the high concentration of GSH and low ROS levels in HCC cells. The summary of metabolites and enzymes related to the GSH metabolism pathway based on metabolomics and transcriptomics is shown in Figure 4B.

### Alterations of cholesterol and the bile acid metabolism pathway in HCC

Cholesterol plays an important role not only in the formation of the cell membrane but also the synthesis of bile acids, vitamin D, and steroid hormones 24. Compared to wild‐type liver tissues, in HCC, the concentration of cholesterol was significantly increased, while glycine and cholate were significantly decreased (Table 1). Consistently, the transcriptional analysis showed that the enzymes related to cholesterol synthesis, such as *Lss*,* Dhcr7*,* Mvd*, and *Fntb,* were significantly upregulated in HCC compared to wild‐type liver tissues. However, the enzymes associated with bile acid synthesis from cholesterol, such as *Cyp7a1*,* Cyp27a1*,* Baat*, and *Cyp8b1*, were significantly downregulated in HCC (Table 2). The summary of metabolites and enzymes related to the cholesterol metabolism pathway based on metabolomics and transcriptomics is shown in Figure 4C.

## Discussion

One of the hallmarks of cancers and precancerous lesions is the increase in *de novo* fatty acid synthesis regardless of the serum lipid levels 25. The liver is the major organ for regulating lipid metabolism, and changes in lipid homeostasis are observed in various liver diseases 26. Clinically, fat deposition is often (36%) seen in well‐differentiated HCCs between 1.1 and 1.5 cm in size 27. Recent evidence showed that both *de novo* synthesized and exogenous fatty acids support the growth of HCC cells 28. Although fatty acids are recognized as an important risk factor in hepatocarcinogenesis, the detailed mechanisms related to fatty acid accumulation remain unknown.

In this study, we showed that abundant quantities of lipid droplets (Fig. 1) are a histopathological characteristic of HCC and detected significantly elevated levels of triglyceride and cholesterol in HCC of Ras‐Tg mice compared to the liver tissues of wild‐type mice (Fig. 4A). Further integrative analysis of the metabolomic and transcriptomic data showed that the key enzymes associated with lipid biosynthesis were significantly enhanced at the mRNA level. In addition, acetyl‐CoA, which is a basic raw material for lipid *de novo* biosynthesis, is continuously supplied by (1) the enhanced glycolysis pathway, which produces pyruvate, and (2) the enhanced citrate shuttle, which converts and transfers mitochondrial pyruvate into cytosol acetyl‐CoA for further lipid synthesis. Additionally, another raw material for lipid synthesis, NADPH, was sufficiently supplied by enhanced PPP (Fig. 4A, Tables 1, 2). This evidence suggests that there are underlying molecular mechanisms for the increase in *de novo* lipid synthesis in HCC cells. Importantly, the combination of TCA and the citrate shuttle to switch mitochondrial pyruvate to cytosol acetyl‐CoA may be the key point for lipid synthesis and offers possible targets for the treatment of steatosis and HCC.

The roles of cholesterol in hepatic tumorigenesis and development are obscure. Because the liver is the dominant *de novo* synthesis organ for cholesterol 29, cholesterol synthesis in the liver is reduced, and the serum cholesterol content is gradually depressed along with the progression of liver diseases from chronic hepatitis to cirrhosis 30. Interestingly, several clinical reports showed that the serum cholesterol levels increased in cirrhosis patients with HCC compared to cirrhosis patients without HCC 31, 32, 33. This evidence indicates that the cholesterol levels were elevated in HCC cells 34, and the relatively lower serum level of cholesterol in HCC patients may result from the severity of cirrhosis, which occurred for 80% of HCCs 35. Therefore, the negative correlation of the serum cholesterol level to the mortality of HCC patients 36 may reflect the functional reserve for liver damage 37. Furthermore, several studies have suggested an important role for cholesterol in liver cancer 38, 39. Intriguingly, this study showed that cholesterol was significantly elevated in the HCC cells of Ras‐Tg mice (Fig. 4A). Apart from the sufficient supply of acetyl‐CoA and NADPH and the elevated levels of enzymes related to cholesterol biosynthesis, we found that bile acid synthesis pathways were attenuated via downregulation of the expression of multiple key enzymes that may play important roles in the accumulation of cholesterol in HCC of Ras‐Tg mice (Fig. 4A, C, Tables 1, 2). However, the detailed mechanisms related to cholesterol accumulation and its roles in hepatic tumorigenesis and development need to be investigated further.

Bile acid homeostasis is disrupted in multiple liver diseases, and the association between elevated BA levels and liver cancer has been demonstrated in both human and animal studies. Cell death and inflammation induced by BAs are suggested to be the underlying mechanisms 40. However, other investigations indicate that bile acid tends to be downregulated in clinical human HCC 41, and a significant negative correlation between bile acid levels and tumor burden was also observed 42. Consistent with the latter, we find that bile acid biosynthesis was substantially attenuated and that bile acid levels were significantly decreased in hepatic tumors. ERK is especially suggested to play important roles in suppressing Cyp7a1 and Cyp8b1, which are the rate‐limiting enzymes for bile acid biosynthesis 43. Consistently, activated Ras/ERK is a feature of hepatic tumors in Ras‐Tg mice, and the expression levels of Cyp7a1 and Cyp8b1 were significantly downregulated (Table 2). Because the Ras/ERK signaling pathway is frequently hyperactivated in human HCC 13, we suggest that the Ras/ERK pathway may contribute to the accumulation of cholesterol in HCC cells by suppressing bile acid biosynthesis.

High levels of ROS play dual functions not only in damaging biological macromolecules and destroying cells but also in promoting tumorigenesis 44. However, recent evidence indicates that in vivo tumor cells showed lower ROS levels, which contributes to tumorigenesis 45, 46. This evidence questions the role of ROS in tumorigenesis and development. The expression of the *ras* oncogene leads to the dysfunction of mitochondria and elevation of ROS levels 47. This phenomenon was also observed in nontumor hepatocytes from Ras‐Tg mice, which showed significantly higher ROS levels compared to the hepatocytes from wild‐type mice 11. However, there was no difference in ROS levels between the HCC cells of Ras‐Tg mice and hepatocytes of wild‐type mice (Fig. 4B). This evidence indicates that the roles of high levels of ROS in *ras* oncogene‐expressing hepatocytes in tumorigenesis remain unclear, yet the relatively low levels of ROS are essential for HCC development. However, the strategies employed by tumor cells to downregulate ROS levels are still unknown. Glutathione, which is one of the main ROS‐eliminating molecules, has been shown to be elevated in tumor cells 48. In HCC of Ras‐Tg mice, a significantly elevated quantity of GSH was also observed (Fig. 4B, Table 1). However, *Gpx1*, which is the main enzyme that eliminates ROS by converting GSH into GSSG, was significantly decreased in HCC cells (Fig. 4B, Table 2). Therefore, whether the high ratio of GSH/GSSG contributes to low ROS levels has yet to be investigated. In addition, *Bcl2* and *Ucp2*, which are two molecules that have been recognized to play important roles in eliminating ROS 49, 50, were also significantly overexpressed in HCC cells (Table 2). Taken together, although the mechanisms associated with a normal level of ROS in HCC cells have yet to be elucidated, the high ratio of GSH/GSSG and high expression levels of *Bcl2* and *Ucp2* may be involved.

The interpretation of our findings may be limited to the mouse HCC induced by the *Hras12V* oncogene and the analysis based on static metabolic phenotypes. In human HCC, although hyperactivation of Ras/MAPK is a frequent event, a portion of patients do not show signs of hyperactivation 51. In addition, various metabolic profiles have been reported due to the complicated causes, different grades, or subgroups of human HCC, and there have been different study designs 41, 52, 53, 54. Similar to the findings on HCC of Ras‐Tg mice, it was suggested that there was a high glycolytic conversion of glucose into pyruvate in the cytosol followed by conversion into acetyl‐CoA and entry into the mitochondrial citric acid cycle with little conversion of pyruvate into lactate or alanine based on a panel of 31 pairs of human HCC tumors and corresponding nontumor liver tissues 52. Further ascertaining the metabolic fluxes in HCC of Ras‐Tg mice by using dynamic data through isotopic substrate labeling experiments will contribute to generalizable knowledge of HCC.

In addition, several limitations of this study must be mentioned. (1) We only presented a snapshot of differences in metabolic pools. Without dynamic 13C‐isotopic labeling experiments, it is hard to ascertain metabolic fluxes and rates. Therefore, the interpretation offered in this study needs further verification. (2) The metabolic profile of the nontumor liver harboring the *ras* oncogene of Ras‐Tg mice was not investigated in this study. It will be of great significance to detect differences between cells that transformed into malignant tumors and those that did not transform while expressing the same *ras* oncogene were detected. (3) The metabolic profile of female HCC was not investigated in this study. It will be interesting to compare metabolic profiles of hepatic tumorigenesis between males and females, which should offer valuable information for determining the source of male prevalence in HCC. The investigations addressing these issues are ongoing in our laboratory.

In summary, the mouse HCC induced by the *Hras12V* oncogene was analyzed by integrative analysis of metabolomics and transcriptomics. The accumulation of triglycerides and cholesterol in HCC is related to the overexpression of lipid biosynthesis‐related enzymes and sufficient supply of acetyl‐CoA and NADPH by accelerated glycolysis, the citrate shuttle, and PPP. Specifically, the downregulation of bile acid synthesis‐associated enzymes may play an important role in cholesterol accumulation. In addition, the elevated quantities of GSH, the GSH/GSSG ratio, and *Bcl2* and *Ucp2* may contribute to a normal level of ROS in HCC.

## Conflict of Interest

The authors declare no conflicts of interest.

## Supporting information


**Figure S1.** Typical GC/MS total ion current (TIC) chromatograms of HCC of Ras‐Tg mice and liver tissues of wild‐type mice.

**Figure S2.** Tissue lipids detected by oil red staining.

**Table S1.** Differentially expressed transcripts between HCC of Ras‐Tg mice and liver tissues of wild‐type mice.

**Table S2.** KEGG pathway enrichment analysis for upregulated genes in HCC of Ras‐Tg mice compared to liver tissues of wild‐type mice.

**Table S3.** KEGG pathway enrichment analysis for downregulated genes in HCC of Ras‐Tg mice compared to liver tissues of wild‐type mice.

**Table S4.** The number of metabolites in the pathways output by MSEA.

**Table S5.** Significant metabolites identified in the pathways output by MSEA.

**Table S6.** Overrepresentation analysis of the metabolic pathways output by MSEA.

## References

[1] Parkin, D. M. , F. Bray , J. Ferlay , and P. Pisani . 2005 Global cancer statistics, 2002. CA Cancer J. Clin. 55:74–108. 15761078 10.3322/canjclin.55.2.74

[2] Boguski, M. S. , and F. McCormick . 1993 Proteins regulating Ras and its relatives. Nature 366:643–654. 8259209 10.1038/366643a0

[3] Hunter, T. 1997 Oncoprotein networks. Cell 88:333–346. 9039260 10.1016/s0092-8674(00)81872-3

[4] Newell, P. , S. Toffanin , A. Villanueva , D. Y. Chiang , B. Minguez , L. Cabellos , et al. 2009 Ras pathway activation in hepatocellular carcinoma and anti‐tumoral effect of combined sorafenib and rapamycin in vivo. J. Hepatol. 51:725–733. 19665249 10.1016/j.jhep.2009.03.028 PMC2970800

[5] Wang, A. G. , H. B. Moon , M. R. Lee , C. Y. Hwang , K. S. Kwon , S. L. Yu , et al. 2005 Gender‐dependent hepatic alterations in H‐ras12V transgenic mice. J. Hepatol. 43:836–844. 16087271 10.1016/j.jhep.2005.04.012

[6] Rong, Z. , T. Fan , H. Li , J. Li , K. Wang , X. Wang , et al. 2017 Differential proteomic analysis of gender‐dependent hepatic tumorigenesis in Hras12V transgenic mice. Mol. Cell Proteomics 16:1475–1490. 28512230 10.1074/mcp.M116.065474 PMC5546199

[7] Wang, K. , X. Nie , Z. Rong , T. Fan , J. Li , X. Wang , et al. 2017 B lymphocytes repress hepatic tumorigenesis but not development in Hras12V transgenic mice. Int. J. Cancer 141:1201–1214. 28580661 10.1002/ijc.30823

[8] Kim, J. H. , H. J. Shin , H. L. Ha , Y. H. Park , T. H. Kwon , M. R. Jung , et al. 2014 Methylsulfonylmethane suppresses hepatic tumor development through activation of apoptosis. World J. Hepatol. 6:98–106. 24575169 10.4254/wjh.v6.i2.98 PMC3934636

[9] Kopanja, D. , A. Pandey , M. Kiefer , Z. Wang , N. Chandan , J. R. Carr , et al. 2015 Essential roles of FoxM1 in Ras‐induced liver cancer progression and in cancer cells with stem cell features. J. Hepatol. 63:429–436. 25828473 10.1016/j.jhep.2015.03.023 PMC4508215

[10] Wang, A. G. , H. B. Moon , J. I. Chae , J. M. Kim , Y. E. Kim , D. Y. Yu , et al. 2011 Steatosis induced by the accumulation of apolipoprotein A‐I and elevated ROS levels in H‐ras12V transgenic mice contributes to hepatic lesions. Biochem. Biophys. Res. Commun. 409:532–538. 21600874 10.1016/j.bbrc.2011.05.039

[11] Kim, S. K. , H. Kim , G. Y. Koh , D. S. Lim , D. Y. Yu , M. D. Kim , et al. 2015 Mouse hepatic tumor vascular imaging by experimental selective angiography. PLoS ONE 10:e0131687. 26131558 10.1371/journal.pone.0131687 PMC4489182

[12] Bae, H. J. , K. H. Jung , J. W. Eun , Q. Shen , H. S. Kim , S. J. Park , et al. 2015 MicroRNA‐221 governs tumor suppressor HDAC6 to potentiate malignant progression of liver cancer. J. Hepatol. 63:408–419. 25817558 10.1016/j.jhep.2015.03.019

[13] Wang, A. G. , Y. N. Song , J. Chen , H. L. Li , J. Y. Dong , H. P. Cui , et al. 2014 Activation of RAS/ERK alone is insufficient to inhibit RXRalpha function and deplete retinoic acid in hepatocytes. Biochem. Biophys. Res. Commun. 452:801–807. 25218146 10.1016/j.bbrc.2014.09.007

[14] Kim, Y. K. , A. Minai‐Tehrani , J. H. Lee , C. S. Cho , M. H. Cho , and H. L. Jiang . 2013 Therapeutic efficiency of folated poly(ethylene glycol)‐chitosan‐graft‐polyethylenimine‐Pdcd4 complexes in H‐ras12V mice with liver cancer. Int. J. Nanomedicine 8:1489–1498. 23620665 10.2147/IJN.S42949 PMC3630991

[15] Lee, H. S. , S. Y. Lee , H. L. Ha , D. C. Han , J. M. Han , T. S. Jeong , et al. 2009 2′‐Benzoyloxycinnamaldehyde inhibits tumor growth in H‐ras12V transgenic mice via downregulation of metallothionein. Nutr. Cancer 61:723–734. 19838947 10.1080/01635580902825613

[16] Wang, A. G. , H. B. Moon , S. Y. Chun , T. H. Lee , D. Y. Yu , and D. S. Lee . 2006 Orchiectomy reduces hepatotumorigenesis of H‐ras12V transgenic mice via the MAPK pathway. Life Sci. 79:1974–1980. 16846616 10.1016/j.lfs.2006.06.032

[17] Moon, E. Y. , M. R. Lee , A. G. Wang , J. H. Lee , H. C. Kim , H. M. Kim , et al. 2006 Delayed occurrence of H‐ras12V‐induced hepatocellular carcinoma with long‐term treatment with cinnamaldehydes. Eur. J. Pharmacol. 530:270–275. 16405947 10.1016/j.ejphar.2005.11.053

[18] Heo, C. K. , M. K. Woo , D. Y. Yu , J. Y. Lee , J. S. Yoo , H. S. Yoo , et al. 2010 Identification of autoantibody against fatty acid synthase in hepatocellular carcinoma mouse model and its application to diagnosis of HCC. Int. J. Oncol. 36:1453–1459. 20428769 10.3892/ijo_00000631

[19] Nicholson, J. K. , E. Holmes , J. M. Kinross , A. W. Darzi , Z. Takats , and J. C. Lindon . 2012 Metabolic phenotyping in clinical and surgical environments. Nature 491:384–392. 23151581 10.1038/nature11708

[20] Sung, W. K. , H. Zheng , S. Li , R. Chen , X. Liu , Y. Li , et al. 2012 Genome‐wide survey of recurrent HBV integration in hepatocellular carcinoma. Nat. Genet. 44:765–769. 22634754 10.1038/ng.2295

[21] Cuperlovic‐Culf, M. , N. Belacel , and A. Culf . 2008 Integrated analysis of transcriptomics and metabolomics profiles. Expert. Opin. Med. Diagn. 2:497–509. 23495739 10.1517/17530059.2.5.497

[22] Chen, K. , C. Zhu , M. Cai , D. Fu , B. Cheng , Z. Cai , et al. 2014 Integrative metabolome and transcriptome profiling reveals discordant glycolysis process between osteosarcoma and normal osteoblastic cells. J. Cancer Res. Clin. Oncol. 140:1715–1721. 24919440 10.1007/s00432-014-1719-y PMC11823902

[23] Wise, D. R. , and C. B. Thompson . 2010 Glutamine addiction: a new therapeutic target in cancer. Trends Biochem. Sci. 35:427–433. 20570523 10.1016/j.tibs.2010.05.003 PMC2917518

[24] Ikonen, E. 2008 Cellular cholesterol trafficking and compartmentalization. Nat. Rev. Mol. Cell Biol. 9:125–138. 18216769 10.1038/nrm2336

[25] Menendez, J. A. , and R. Lupu . 2007 Fatty acid synthase and the lipogenic phenotype in cancer pathogenesis. Nat. Rev. Cancer 7:763–777. 17882277 10.1038/nrc2222

[26] Jiang, J. T. , N. Xu , X. Y. Zhang , and C. P. Wu . 2007 Lipids changes in liver cancer. J. Zhejiang Univ. Sci. B 8:398–409. 17565510 10.1631/jzus.2007.B0398 PMC1879165

[27] Kutami, R. , Y. Nakashima , O. Nakashima , K. Shiota , and M. Kojiro . 2000 Pathomorphologic study on the mechanism of fatty change in small hepatocellular carcinoma of humans. J. Hepatol. 33:282–289. 10952246 10.1016/s0168-8278(00)80369-4

[28] Cao, D. , X. Song , L. Che , X. Li , M. G. Pilo , G. Vidili , et al. 2017 Both de novo synthetized and exogenous fatty acids support the growth of hepatocellular carcinoma cells. Liver Int. 37:80–89. 27264722 10.1111/liv.13183 PMC5140766

[29] Lewis, G. F. 2006 Determinants of plasma HDL concentrations and reverse cholesterol transport. Curr. Opin. Cardiol. 21:345–352. 16755204 10.1097/01.hco.0000231405.76930.a0

[30] Jiang, M. , F. Liu , W. J. Xiong , L. Zhong , W. Xu , F. Xu , et al. 2010 Combined MELD and blood lipid level in evaluating the prognosis of decompensated cirrhosis. World J. Gastroenterol. 16:1397–1401. 20238407 10.3748/wjg.v16.i11.1397 PMC2842532

[31] Alsabti, E. A. 1979 Serum lipids in hepatoma. Oncology 36:11–14. 221872 10.1159/000225310

[32] Venturini, I. , R. Amedei , G. Modonesi , R. Cosenza , L. Miglioli , G. Cioni , et al. 1999 May plasma cholesterol level be considered a neoplastic marker in liver disease from cirrhosis to hepatocellular carcinoma? Ital. J. Gastroenterol. Hepatol. 31:61–65. 10091105

[33] Venturini, I. , M. L. Zeneroli , L. Corsi , C. Baraldi , C. Ferrarese , N. Pecora , et al. 1998 Diazepam binding inhibitor and total cholesterol plasma levels in cirrhosis and hepatocellular carcinoma. Regul. Pept. 74:31–34. 9657356 10.1016/s0167-0115(98)00013-5

[34] Kawata, S. , K. Takaishi , T. Nagase , N. Ito , Y. Matsuda , S. Tamura , et al. 1990 Increase in the active form of 3‐hydroxy‐3‐methylglutaryl coenzyme A reductase in human hepatocellular carcinoma: possible mechanism for alteration of cholesterol biosynthesis. Cancer Res. 50:3270–3273. 2159376

[35] Llovet, J. M. , A. Burroughs , and J. Bruix . 2003 Hepatocellular carcinoma. Lancet 362:1907–1917. 14667750 10.1016/S0140-6736(03)14964-1

[36] Kitahara, C. M. , A. Berrington de Gonzalez , N. D. Freedman , R. Huxley , Y. Mok , S. H. Jee , et al. 2011 Total cholesterol and cancer risk in a large prospective study in Korea. J. Clin. Oncol. 29:1592–1598. 21422422 10.1200/JCO.2010.31.5200 PMC3082977

[37] Wang, Q. , W. Y. Lau , B. Zhang , Z. Zhang , Z. Huang , H. Luo , et al. 2014 Preoperative total cholesterol predicts postoperative outcomes after partial hepatectomy in patients with chronic hepatitis B‐ or C‐related hepatocellular carcinoma. Surgery 155:263–270. 24569301 10.1016/j.surg.2013.08.017

[38] Montero, J. , A. Morales , L. Llacuna , J. M. Lluis , O. Terrones , G. Basanez , et al. 2008 Mitochondrial cholesterol contributes to chemotherapy resistance in hepatocellular carcinoma. Cancer Res. 68:5246–5256. 18593925 10.1158/0008-5472.CAN-07-6161

[39] Morioka, S. , K. Sai , E. Omori , Y. Ikeda , K. Matsumoto , and J. Ninomiya‐Tsuji . 2016 TAK1 regulates hepatic lipid homeostasis through SREBP. Oncogene 35:3829–3838. 26973245 10.1038/onc.2015.453 PMC4956508

[40] Wang, X. , X. Fu , C. Van Ness , Z. Meng , X. Ma , and W. Huang . 2013 Bile Acid Receptors and Liver Cancer. Curr. Pathobiol. Rep. 1:29–35. 23420103 10.1007/s40139-012-0003-6 PMC3571718

[41] Fitian, A. I. , and R. Cabrera . 2017 Disease monitoring of hepatocellular carcinoma through metabolomics. World J. Hepatol. 9:1–17. 28105254 10.4254/wjh.v9.i1.1 PMC5220267

[42] Xiao, J. F. , R. S. Varghese , B. Zhou , M. R. Nezami Ranjbar , Y. Zhao , T. H. Tsai , et al. 2012 LC‐MS based serum metabolomics for identification of hepatocellular carcinoma biomarkers in Egyptian cohort. J. Proteome Res. 11:5914–5923. 23078175 10.1021/pr300673x PMC3719870

[43] Kong, B. , L. Wang , J. Y. Chiang , Y. Zhang , C. D. Klaassen , and G. L. Guo . 2012 Mechanism of tissue‐specific farnesoid X receptor in suppressing the expression of genes in bile‐acid synthesis in mice. Hepatology 56:1034–1043. 22467244 10.1002/hep.25740 PMC3390456

[44] Panieri, E. , and M. M. Santoro . 2016 ROS homeostasis and metabolism: a dangerous liason in cancer cells. Cell Death Dis. 7:e2253. 27277675 10.1038/cddis.2016.105 PMC5143371

[45] DeNicola, G. M. , F. A. Karreth , T. J. Humpton , A. Gopinathan , C. Wei , K. Frese , et al. 2011 Oncogene‐induced Nrf2 transcription promotes ROS detoxification and tumorigenesis. Nature 475:106–109. 21734707 10.1038/nature10189 PMC3404470

[46] Sayin, V. I. , M. X. Ibrahim , E. Larsson , J. A. Nilsson , P. Lindahl , and M. O. Bergo . 2014 Antioxidants accelerate lung cancer progression in mice. Sci. Transl. Med. 6:221ra15. 10.1126/scitranslmed.3007653 24477002

[47] Bellot, G. L. , D. Liu , and S. Pervaiz . 2013 ROS, autophagy, mitochondria and cancer: ras, the hidden master? Mitochondrion 13:155–162. 22750269 10.1016/j.mito.2012.06.007

[48] Marengo, B. , M. Nitti , A. L. Furfaro , R. Colla , C. D. Ciucis , U. M. Marinari , et al. 2016 Redox homeostasis and cellular antioxidant systems: crucial players in cancer growth and therapy. Oxid. Med. Cell. Longev. 2016:6235641. 27418953 10.1155/2016/6235641 PMC4932173

[49] Asher, G. , J. Lotem , R. Kama , L. Sachs , and Y. Shaul . 2002 NQO1 stabilizes p53 through a distinct pathway. Proc. Natl. Acad. Sci. U S A 99:3099–3104. 11867746 10.1073/pnas.052706799 PMC122479

[50] Bae, Y. S. , H. Oh , S. G. Rhee , and Y. D. Yoo . 2011 Regulation of reactive oxygen species generation in cell signaling. Mol. Cells 32:491–509. 22207195 10.1007/s10059-011-0276-3 PMC3887685

[51] Delire, B. , and P. Starkel . 2015 The Ras/MAPK pathway and hepatocarcinoma: pathogenesis and therapeutic implications. Eur. J. Clin. Invest. 45:609–623. 25832714 10.1111/eci.12441

[52] Beyoglu, D. , S. Imbeaud , O. Maurhofer , P. Bioulac‐Sage , J. Zucman‐Rossi , J. F. Dufour , et al. 2013 Tissue metabolomics of hepatocellular carcinoma: tumor energy metabolism and the role of transcriptomic classification. Hepatology 58:229–238. 23463346 10.1002/hep.26350 PMC3695036

[53] Darpolor, M. M. , S. S. Basu , A. Worth , D. S. Nelson , R. H. Clarke‐Katzenberg , J. D. Glickson , et al. 2014 The aspartate metabolism pathway is differentiable in human hepatocellular carcinoma: transcriptomics and (13) C‐isotope based metabolomics. NMR Biomed. 27:381–389. 24497316 10.1002/nbm.3072 PMC3962779

[54] Zhang, L. , Y. Huang , M. Lian , Z. Fan , Y. Tian , Y. Wang , et al. 2017 Metabolic profiling of hepatitis B virus‐related hepatocellular carcinoma with diverse differentiation grades. Oncol. Lett. 13:1204–1210. 28454235 10.3892/ol.2017.5596 PMC5403281

