# Supplementary figures and images for: Metabolomic and transcriptomic profiling of hepatocellular carcinomas in Hras12V transgenic mice
Source: Cancer Med. 2017 Sep 21;6(10):2370–84. doi: 10.1002/cam4.1177 (PMC5633588; doi:10.1002/cam4.1177)

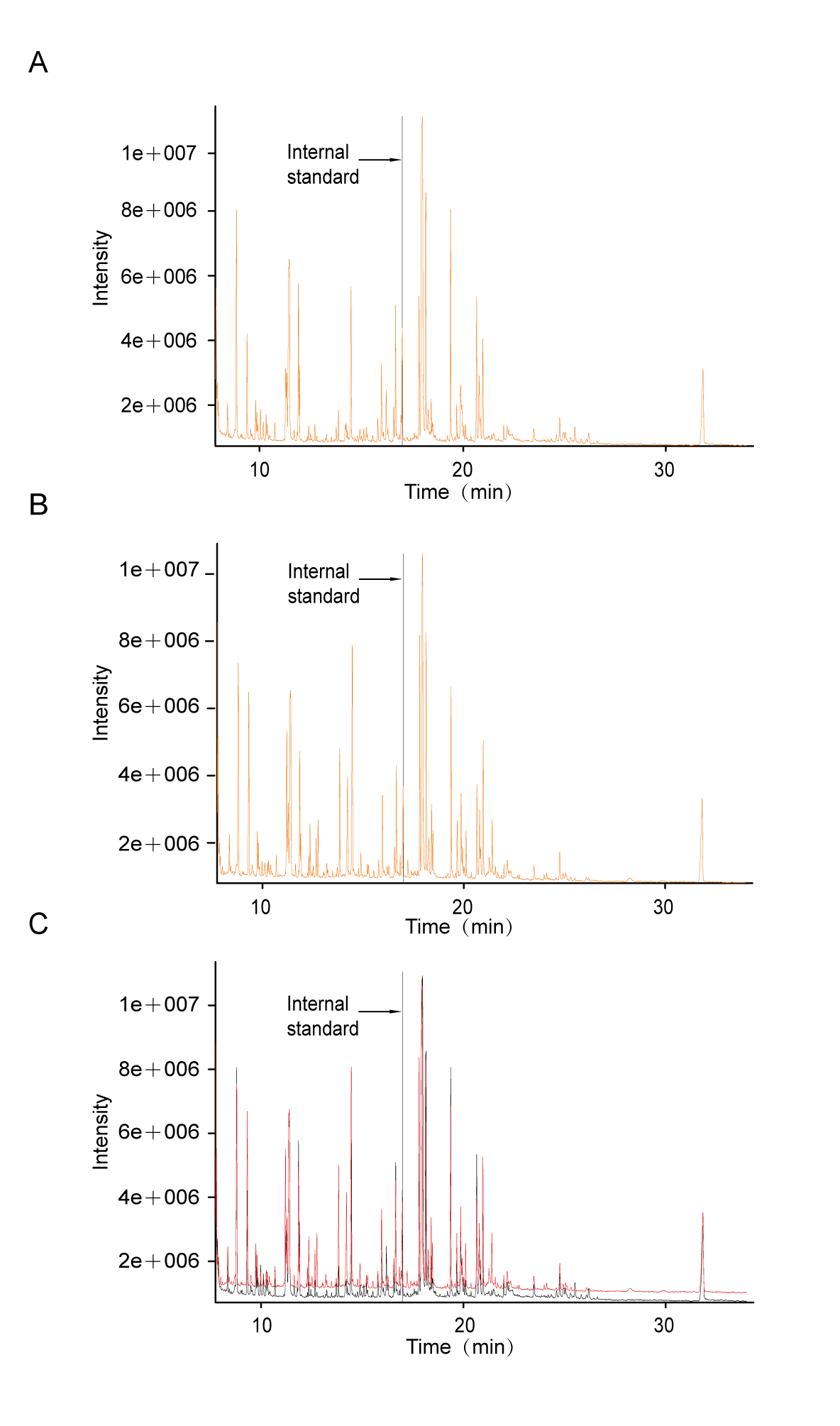

Supplement: Supplementary file 1 — Figure S1. Typical GC/MS total ion current (TIC) chromatograms of HCC of Ras‐Tg mice and liver tissues of wild‐type mice. [file CAM4-6-2370-s001.tif]

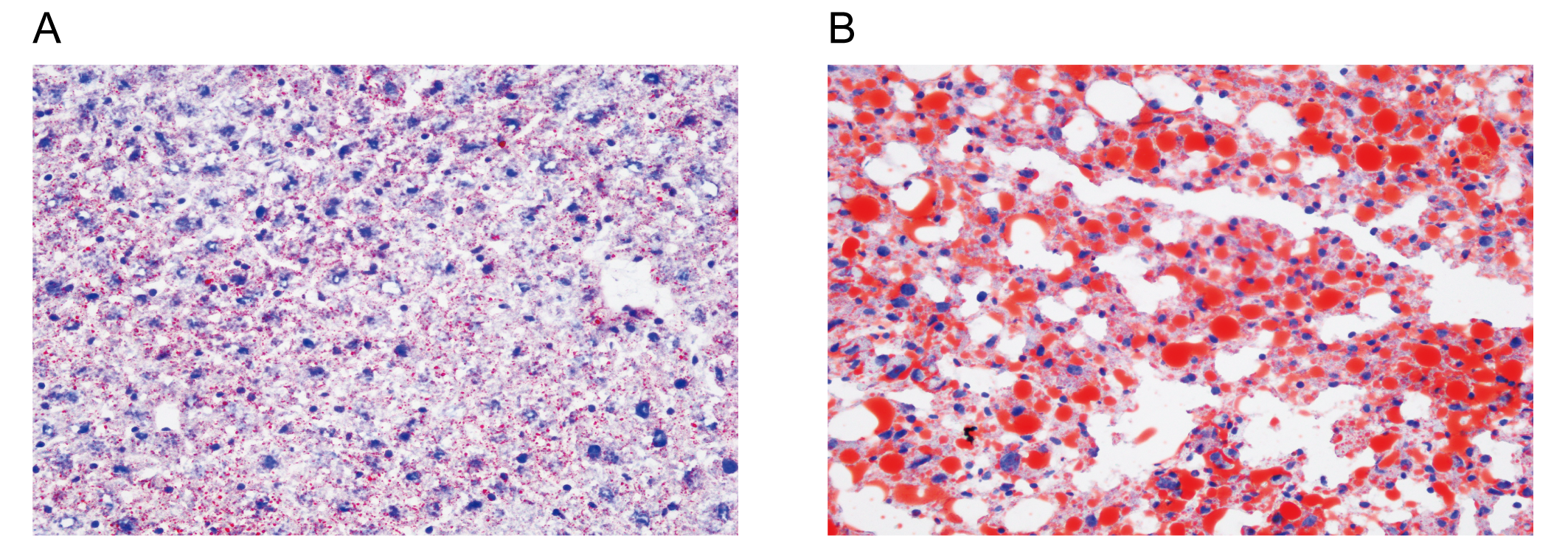

Supplement: Supplementary file 2 — Figure S2. Tissue lipids detected by oil red staining. [file CAM4-6-2370-s002.tif]
