# Supplementary material for: Metabolomic and transcriptomic profiling of hepatocellular carcinomas in Hras12V transgenic mice
Source: Cancer Med. 2017 Sep 21;6(10):2370–84. doi: 10.1002/cam4.1177 (PMC5633588; doi:10.1002/cam4.1177)
Supplement: Supplementary file 3 — Table S1. Differentially expressed transcripts between HCC of Ras‐Tg mice and liver tissues of wild‐type mice. [file CAM4-6-2370-s003.doc]

**Supplementary Table 1.** Differently expressed transcripts between HCC of Ras-Tg and liver tissues of wild-type mice.

| **mRNA accession Name** | **Gene Symbol** | **W value** | **T value** | **Fold change** | **P value** |
| --- | --- | --- | --- | --- | --- |
| NM_001003898 | Tardbp | 1.74E+01 | 0.00E+00 | -1.79769e+308 | 1.35E-04 |
| NM_001008546 | Tardbp | 2.52E+00 | 0.00E+00 | -1.79769e+308 | 1.46E-03 |
| NM_001077190 | Abi1 | 8.62E+00 | 0.00E+00 | -1.79769e+308 | 4.53E-04 |
| NM_001077193 | Abi1 | 5.27E-02 | 0.00E+00 | -1.79769e+308 | 1.16E-35 |
| NM_001110305 | Keap1 | 5.53E+00 | 0.00E+00 | -1.79769e+308 | 1.19E-03 |
| NM_001122953 | Nfia | 9.36E-01 | 0.00E+00 | -1.79769e+308 | 3.70E-03 |
| NM_001146122 | Psap | 7.05E+01 | 0.00E+00 | -1.79769e+308 | 2.10E-04 |
| NM_001164079 | Tia1 | 5.04E-03 | 0.00E+00 | -1.79769e+308 | 4.82E-07 |
| NM_001164671 | Dnaja1 | 1.67E+00 | 0.00E+00 | -1.79769e+308 | 3.45E-03 |
| NM_001168621 | Znrf1 | 6.22E-03 | 0.00E+00 | -1.79769e+308 | 2.38E-27 |
| NM_001177850 | Asph | 3.16E+00 | 0.00E+00 | -1.79769e+308 | 3.79E-03 |
| NM_001177853 | Asph | 9.29E-02 | 0.00E+00 | -1.79769e+308 | 1.97E-21 |
| NM_001177854 | Asph | 5.20E-02 | 0.00E+00 | -1.79769e+308 | 1.97E-21 |
| NM_001177973 | Irak1 | 5.11E+00 | 0.00E+00 | -1.79769e+308 | 9.67E-04 |
| NM_001190857 | Pdlim5 | 8.38E+00 | 0.00E+00 | -1.79769e+308 | 1.50E-03 |
| NM_001195205 | Trmt2a | 3.64E+00 | 0.00E+00 | -1.79769e+308 | 2.17E-03 |
| NM_001198570 | Abi2 | 4.44E-03 | 0.00E+00 | -1.79769e+308 | 4.82E-07 |
| NM_001198877 | Dync1i2 | 3.39E+00 | 0.00E+00 | -1.79769e+308 | 4.02E-03 |
| NM_001198878 | Dync1i2 | 3.45E+00 | 0.00E+00 | -1.79769e+308 | 3.94E-03 |
| NM_001242605 | Acin1 | 6.13E+00 | 0.00E+00 | -1.79769e+308 | 1.82E-03 |
| NM_001242606 | Acin1 | 3.92E+00 | 0.00E+00 | -1.79769e+308 | 3.68E-03 |
| NM_007615 | Ctnnd1 | 9.69E+00 | 0.00E+00 | -1.79769e+308 | 1.72E-04 |
| NM_023336 | Brd3 | 1.66E+00 | 0.00E+00 | -1.79769e+308 | 3.65E-03 |
| NM_025802 | Pnpla2 | 2.51E-02 | 0.00E+00 | -1.79769e+308 | 9.29E-30 |
| NM_030723 | Pum2 | 1.45E+00 | 0.00E+00 | -1.79769e+308 | 3.62E-03 |
| NM_133951 | Rrp8 | 1.51E+01 | 0.00E+00 | -1.79769e+308 | 2.39E-04 |
| NM_183312 | Synm | 1.17E-03 | 0.00E+00 | -1.79769e+308 | 1.35E-03 |
| NM_211357 | Eya3 | 7.06E-03 | 0.00E+00 | -1.79769e+308 | 1.28E-12 |
| NR_037579 | Angel2 | 3.27E-03 | 0.00E+00 | -1.79769e+308 | 1.00E-13 |
| NR_038349 | AV039307 | 8.19E+00 | 0.00E+00 | -1.79769e+308 | 4.18E-03 |
| NR_040257 | 2210408F21Rik | 2.91E-01 | 0.00E+00 | -1.79769e+308 | 1.36E-06 |
| NM_001025379 | Sema3g | 0.00E+00 | 4.91E-01 | 1.79769e+308 | 4.09E-03 |
| NM_001033301 | Fhdc1 | 0.00E+00 | 5.72E-01 | 1.79769e+308 | 1.47E-03 |
| NM_001033382 | Cacna2d4 | 0.00E+00 | 1.45E-02 | 1.79769e+308 | 5.38E-05 |
| NM_001033460 | Ccdc164 | 0.00E+00 | 9.10E+00 | 1.79769e+308 | 1.34E-04 |
| NM_001034876 | Ccdc24 | 0.00E+00 | 1.99E+00 | 1.79769e+308 | 3.03E-03 |
| NM_001039129 | Hnrnpa1 | 0.00E+00 | 2.29E+00 | 1.79769e+308 | 1.10E-03 |
| NM_001039243 | Gm7092 | 0.00E+00 | 2.79E+01 | 1.79769e+308 | 1.38E-04 |
| NM_001039562 | Ankrd37 | 0.00E+00 | 4.89E+00 | 1.79769e+308 | 1.43E-03 |
| NM_001039700 | Pkd1l3 | 0.00E+00 | 5.31E-01 | 1.79769e+308 | 1.45E-03 |
| NM_001040654 | Cdkn2a | 0.00E+00 | 2.63E+00 | 1.79769e+308 | 4.09E-03 |
| NM_001042523 | Txnrd1 | 0.00E+00 | 1.13E+00 | 1.79769e+308 | 1.15E-03 |
| NM_001042528 | Cacna1b | 0.00E+00 | 1.03E+00 | 1.79769e+308 | 3.50E-04 |
| NM_001042614 | Sepp1 | 0.00E+00 | 3.83E+00 | 1.79769e+308 | 3.26E-04 |
| NM_001048057 | Rpl38 | 0.00E+00 | 1.26E+01 | 1.79769e+308 | 3.08E-03 |
| NM_001081468 | Gtf2ird1 | 0.00E+00 | 1.64E-03 | 1.79769e+308 | 1.35E-39 |
| NM_001104614 | Vmn2r3 | 0.00E+00 | 2.43E+00 | 1.79769e+308 | 4.78E-04 |
| NM_001105252 | Tmc5 | 0.00E+00 | 5.58E+00 | 1.79769e+308 | 1.31E-04 |
| NM_001111291 | Caprin1 | 0.00E+00 | 1.09E+01 | 1.79769e+308 | 2.11E-04 |
| NM_001113351 | Synj2 | 0.00E+00 | 2.13E+00 | 1.79769e+308 | 2.69E-04 |
| NM_001114098 | 1110012J17Rik | 0.00E+00 | 2.89E-01 | 1.79769e+308 | 3.14E-03 |
| NM_001122640 | Arhgap17 | 0.00E+00 | 1.66E+00 | 1.79769e+308 | 5.68E-04 |
| NM_001136222 | Acsl3 | 0.00E+00 | 2.15E+00 | 1.79769e+308 | 3.53E-04 |
| NM_001142631 | Spdya | 0.00E+00 | 6.71E-02 | 1.79769e+308 | 2.30E-06 |
| NM_001144987 | Spg20 | 0.00E+00 | 5.50E-01 | 1.79769e+308 | 3.10E-03 |
| NM_001145015 | Arhgap40 | 0.00E+00 | 1.25E+00 | 1.79769e+308 | 2.81E-03 |
| NM_001145899 | Slc15a2 | 0.00E+00 | 1.02E-01 | 1.79769e+308 | 1.35E-03 |
| NM_001146049 | Htatip2 | 0.00E+00 | 1.96E+00 | 1.79769e+308 | 2.35E-03 |
| NM_001159577 | Lnx1 | 0.00E+00 | 3.93E-04 | 1.79769e+308 | 5.52E-66 |
| NM_001159617 | Pigp | 0.00E+00 | 1.01E-01 | 1.79769e+308 | 6.52E-133 |
| NM_001159620 | Pigp | 0.00E+00 | 3.12E-02 | 1.79769e+308 | 6.52E-133 |
| NM_001160180 | Tor1aip2 | 0.00E+00 | 1.37E+00 | 1.79769e+308 | 1.43E-03 |
| NM_001160711 | Cd300lg | 0.00E+00 | 8.84E-01 | 1.79769e+308 | 2.77E-03 |
| NM_001162417 | Myef2 | 0.00E+00 | 1.07E+00 | 1.79769e+308 | 2.64E-03 |
| NM_001163540 | Plec | 0.00E+00 | 4.81E+00 | 1.79769e+308 | 9.66E-04 |
| NM_001163578 | Prom1 | 0.00E+00 | 5.93E+00 | 1.79769e+308 | 1.35E-04 |
| NM_001163582 | Prom1 | 0.00E+00 | 1.79E+00 | 1.79769e+308 | 8.48E-04 |
| NM_001163643 | Map3k12 | 0.00E+00 | 3.42E-01 | 1.79769e+308 | 3.79E-03 |
| NM_001163704 | Fbxo6 | 0.00E+00 | 1.69E+00 | 1.79769e+308 | 3.14E-03 |
| NM_001164041 | Smad5 | 0.00E+00 | 2.89E-01 | 1.79769e+308 | 3.44E-03 |
| NM_001164045 | Ccl27a | 0.00E+00 | 4.85E-02 | 1.79769e+308 | 1.05E-36 |
| NM_001164075 | Tgif1 | 0.00E+00 | 1.27E+00 | 1.79769e+308 | 3.46E-03 |
| NM_001164117 | Serpinb6a | 0.00E+00 | 3.45E+00 | 1.79769e+308 | 7.40E-04 |
| NM_001164118 | Serpinb6a | 0.00E+00 | 4.75E+00 | 1.79769e+308 | 3.50E-04 |
| NM_001164193 | Mtm1 | 0.00E+00 | 9.72E-03 | 1.79769e+308 | 5.67E-83 |
| NM_001164248 | Tpm1 | 0.00E+00 | 1.55E+01 | 1.79769e+308 | 1.32E-04 |
| NM_001164251 | Tpm1 | 0.00E+00 | 1.57E+01 | 1.79769e+308 | 1.34E-04 |
| NM_001164557 | Pdzk1ip1 | 0.00E+00 | 1.77E+00 | 1.79769e+308 | 4.49E-03 |
| NM_001164573 | Myo1h | 0.00E+00 | 2.04E-03 | 1.79769e+308 | 9.67E-56 |
| NM_001164621 | Rnf14 | 0.00E+00 | 2.02E+00 | 1.79769e+308 | 4.27E-04 |
| NM_001166380 | Armcx1 | 0.00E+00 | 3.53E-03 | 1.79769e+308 | 3.55E-12 |
| NM_001166537 | Hmga1 | 0.00E+00 | 2.86E+00 | 1.79769e+308 | 6.67E-04 |
| NM_001166592 | Eif5a | 0.00E+00 | 2.84E+01 | 1.79769e+308 | 1.90E-04 |
| NM_001167784 | Tcirg1 | 0.00E+00 | 1.05E+00 | 1.79769e+308 | 1.81E-03 |
| NM_001168476 | Ttc23 | 0.00E+00 | 9.82E-01 | 1.79769e+308 | 3.93E-03 |
| NM_001170975 | Dbndd1 | 0.00E+00 | 7.01E-02 | 1.79769e+308 | 2.34E-03 |
| NM_001172093 | Depdc1a | 0.00E+00 | 1.01E-03 | 1.79769e+308 | 2.66E-04 |
| NM_001172481 | Aspn | 0.00E+00 | 8.10E-01 | 1.79769e+308 | 4.22E-03 |
| NM_001177395 | Gm13476 | 0.00E+00 | 1.80E+00 | 1.79769e+308 | 3.74E-03 |
| NM_001177770 | Clcc1 | 0.00E+00 | 6.88E-01 | 1.79769e+308 | 3.40E-03 |
| NM_001177836 | Smox | 0.00E+00 | 1.19E+00 | 1.79769e+308 | 3.63E-03 |
| NM_001177837 | Smox | 0.00E+00 | 1.34E+00 | 1.79769e+308 | 3.45E-03 |
| NM_001177974 | Irak1 | 0.00E+00 | 3.32E+00 | 1.79769e+308 | 2.80E-04 |
| NM_001177976 | Irak1 | 0.00E+00 | 1.08E+00 | 1.79769e+308 | 2.41E-03 |
| NM_001190406 | Gas2l1 | 0.00E+00 | 9.99E-01 | 1.79769e+308 | 1.45E-03 |
| NM_001190856 | Pdlim5 | 0.00E+00 | 4.97E+00 | 1.79769e+308 | 1.44E-04 |
| NM_001198823 | App | 0.00E+00 | 2.15E+01 | 1.79769e+308 | 7.63E-04 |
| NM_001198969 | Itsn2 | 0.00E+00 | 2.11E+00 | 1.79769e+308 | 8.32E-04 |
| NM_001204233 | Spp1 | 0.00E+00 | 6.71E+00 | 1.79769e+308 | 2.28E-04 |
| NM_001204973 | Brd2 | 0.00E+00 | 6.68E-01 | 1.79769e+308 | 1.78E-03 |
| NM_001205355 | Fhdc1 | 0.00E+00 | 5.73E-01 | 1.79769e+308 | 1.41E-03 |
| NM_007579 | Cacna1b | 0.00E+00 | 7.51E-01 | 1.79769e+308 | 6.39E-04 |
| NM_007786 | Csn3 | 0.00E+00 | 1.27E+01 | 1.79769e+308 | 1.44E-04 |
| NM_007867 | Dlx4 | 0.00E+00 | 1.68E+00 | 1.79769e+308 | 1.93E-03 |
| NM_007974 | F2rl1 | 0.00E+00 | 1.01E+01 | 1.79769e+308 | 1.62E-04 |
| NM_008226 | Hcn2 | 0.00E+00 | 7.08E-01 | 1.79769e+308 | 3.07E-03 |
| NM_008405 | Itgb2l | 0.00E+00 | 1.32E+00 | 1.79769e+308 | 1.55E-03 |
| NM_008585 | Mep1a | 0.00E+00 | 1.05E+00 | 1.79769e+308 | 1.98E-03 |
| NM_008586 | Mep1b | 0.00E+00 | 4.77E+00 | 1.79769e+308 | 2.00E-04 |
| NM_008815 | Etv4 | 0.00E+00 | 1.41E+00 | 1.79769e+308 | 1.79E-03 |
| NM_009052 | Bex1 | 0.00E+00 | 8.24E+00 | 1.79769e+308 | 5.79E-04 |
| NM_009074 | Mst1r | 0.00E+00 | 1.41E+00 | 1.79769e+308 | 4.24E-04 |
| NM_009083 | Rpl30 | 0.00E+00 | 1.21E+01 | 1.79769e+308 | 8.47E-04 |
| NM_009375 | Tg | 0.00E+00 | 1.00E-02 | 1.79769e+308 | 4.74E-15 |
| NM_009509 | Vil1 | 0.00E+00 | 7.17E-01 | 1.79769e+308 | 3.11E-03 |
| NM_009601 | Chrnb1 | 0.00E+00 | 1.51E+00 | 1.79769e+308 | 1.83E-03 |
| NM_009610 | Actg2 | 0.00E+00 | 2.20E+00 | 1.79769e+308 | 2.41E-03 |
| NM_009731 | Akr1b7 | 0.00E+00 | 4.17E+00 | 1.79769e+308 | 8.79E-04 |
| NM_009749 | Bex2 | 0.00E+00 | 3.33E+00 | 1.79769e+308 | 2.78E-03 |
| NM_009877 | Cdkn2a | 0.00E+00 | 6.31E+00 | 1.79769e+308 | 8.12E-04 |
| NM_010308 | Gnao1 | 0.00E+00 | 2.86E-02 | 1.79769e+308 | 4.08E-03 |
| NM_010404 | Hap1 | 0.00E+00 | 6.81E-01 | 1.79769e+308 | 2.50E-03 |
| NM_010727 | Lnx1 | 0.00E+00 | 2.79E-04 | 1.79769e+308 | 5.52E-66 |
| NM_011121 | Plk1 | 0.00E+00 | 1.10E+00 | 1.79769e+308 | 2.82E-03 |
| NM_011347 | Selp | 0.00E+00 | 8.62E-01 | 1.79769e+308 | 1.96E-03 |
| NM_011352 | Sema7a | 0.00E+00 | 9.29E-01 | 1.79769e+308 | 1.98E-03 |
| NM_011523 | Synj2 | 0.00E+00 | 3.09E+00 | 1.79769e+308 | 1.60E-04 |
| NM_013538 | Cdca3 | 0.00E+00 | 2.79E+00 | 1.79769e+308 | 1.31E-03 |
| NM_013873 | Sult4a1 | 0.00E+00 | 2.80E+00 | 1.79769e+308 | 4.56E-04 |
| NM_013900 | Mfi2 | 0.00E+00 | 1.06E+00 | 1.79769e+308 | 9.73E-04 |
| NM_013914 | Snai3 | 0.00E+00 | 2.82E+00 | 1.79769e+308 | 1.12E-03 |
| NM_016660 | Hmga1 | 0.00E+00 | 2.86E+00 | 1.79769e+308 | 6.47E-04 |
| NM_016865 | Htatip2 | 0.00E+00 | 2.06E+00 | 1.79769e+308 | 2.33E-03 |
| NM_019487 | Hebp2 | 0.00E+00 | 2.20E+00 | 1.79769e+308 | 1.16E-03 |
| NM_019670 | Diap3 | 0.00E+00 | 5.41E-01 | 1.79769e+308 | 3.95E-03 |
| NM_019753 | Cdh17 | 0.00E+00 | 3.10E+01 | 1.79769e+308 | 2.50E-03 |
| NM_019968 | Arl10 | 0.00E+00 | 2.90E+00 | 1.79769e+308 | 2.41E-03 |
| NM_019977 | Miox | 0.00E+00 | 2.17E+01 | 1.79769e+308 | 1.34E-04 |
| NM_020013 | Fgf21 | 0.00E+00 | 1.23E+01 | 1.79769e+308 | 2.24E-04 |
| NM_021304 | Abhd1 | 0.00E+00 | 4.50E+00 | 1.79769e+308 | 6.62E-04 |
| NM_021544 | Scn5a | 0.00E+00 | 1.86E+00 | 1.79769e+308 | 1.47E-04 |
| NM_021719 | Cldn15 | 0.00E+00 | 1.53E+00 | 1.79769e+308 | 2.34E-03 |
| NM_022886 | Scel | 0.00E+00 | 7.72E-01 | 1.79769e+308 | 2.82E-03 |
| NM_023256 | Krt20 | 0.00E+00 | 1.51E+00 | 1.79769e+308 | 2.15E-03 |
| NM_025464 | Tmem218 | 0.00E+00 | 2.50E+00 | 1.79769e+308 | 3.20E-03 |
| NM_025711 | Aspn | 0.00E+00 | 8.07E-01 | 1.79769e+308 | 4.20E-03 |
| NM_026785 | Ube2c | 0.00E+00 | 2.80E+00 | 1.79769e+308 | 3.20E-03 |
| NM_027011 | Krt5 | 0.00E+00 | 2.83E+00 | 1.79769e+308 | 5.58E-04 |
| NM_027582 | Akr1cl | 0.00E+00 | 1.70E+00 | 1.79769e+308 | 3.42E-03 |
| NM_027924 | Pdgfd | 0.00E+00 | 1.64E+00 | 1.79769e+308 | 2.15E-03 |
| NM_028444 | Prkcdbp | 0.00E+00 | 2.58E+00 | 1.79769e+308 | 3.07E-03 |
| NM_028475 | Ubap2l | 0.00E+00 | 5.20E-03 | 1.79769e+308 | 1.60E-135 |
| NM_028930 | Tmc5 | 0.00E+00 | 6.77E-01 | 1.79769e+308 | 3.69E-03 |
| NM_029182 | Rasd2 | 0.00E+00 | 1.25E+00 | 1.79769e+308 | 1.48E-03 |
| NM_029248 | Taf1d | 0.00E+00 | 2.14E+00 | 1.79769e+308 | 3.25E-03 |
| NM_029434 | Lca5 | 0.00E+00 | 5.18E-02 | 1.79769e+308 | 4.08E-03 |
| NM_029458 | Hormad2 | 0.00E+00 | 1.86E+00 | 1.79769e+308 | 3.07E-03 |
| NM_029617 | Casc5 | 0.00E+00 | 2.71E-01 | 1.79769e+308 | 4.09E-03 |
| NM_029627 | Ly6k | 0.00E+00 | 7.18E+01 | 1.79769e+308 | 2.60E-04 |
| NM_029682 | Stambpl1 | 0.00E+00 | 4.68E+00 | 1.79769e+308 | 2.50E-04 |
| NM_030228 | Gas2l1 | 0.00E+00 | 9.46E-01 | 1.79769e+308 | 1.33E-03 |
| NM_030599 | Klrb1b | 0.00E+00 | 5.81E+01 | 1.79769e+308 | 2.95E-03 |
| NM_033582 | Pcdhgc4 | 0.00E+00 | 5.76E-01 | 1.79769e+308 | 1.97E-03 |
| NM_053015 | Mlph | 0.00E+00 | 7.42E-01 | 1.79769e+308 | 1.55E-03 |
| NM_053128 | Pcdhb3 | 0.00E+00 | 1.03E-02 | 1.79769e+308 | 7.55E-136 |
| NM_053246 | Dok4 | 0.00E+00 | 8.03E-01 | 1.79769e+308 | 3.49E-03 |
| NM_080850 | Pask | 0.00E+00 | 3.48E-01 | 1.79769e+308 | 4.09E-03 |
| NM_133888 | Smpdl3b | 0.00E+00 | 2.22E+00 | 1.79769e+308 | 1.19E-03 |
| NM_134050 | Rab15 | 0.00E+00 | 1.06E+00 | 1.79769e+308 | 1.59E-03 |
| NM_138670 | Mpst | 0.00E+00 | 2.57E+00 | 1.79769e+308 | 1.56E-03 |
| NM_138682 | Lrrc4 | 0.00E+00 | 6.51E-01 | 1.79769e+308 | 2.77E-03 |
| NM_138750 | Prom2 | 0.00E+00 | 4.90E-01 | 1.79769e+308 | 3.41E-03 |
| NM_144529 | Arhgap17 | 0.00E+00 | 1.67E+00 | 1.79769e+308 | 5.75E-04 |
| NM_144557 | Myrip | 0.00E+00 | 2.77E+00 | 1.79769e+308 | 1.51E-04 |
| NM_144852 | Slc7a4 | 0.00E+00 | 5.65E-01 | 1.79769e+308 | 4.09E-03 |
| NM_144946 | Neto1 | 0.00E+00 | 1.47E+00 | 1.79769e+308 | 7.26E-04 |
| NM_145419 | Hkdc1 | 0.00E+00 | 5.96E-01 | 1.79769e+308 | 3.42E-03 |
| NM_145450 | BC022687 | 0.00E+00 | 1.38E+00 | 1.79769e+308 | 2.55E-03 |
| NM_146109 | Als2 | 0.00E+00 | 9.91E-01 | 1.79769e+308 | 1.92E-03 |
| NM_146182 | Klc3 | 0.00E+00 | 2.31E+00 | 1.79769e+308 | 1.26E-03 |
| NM_146248 | Cchcr1 | 0.00E+00 | 9.96E-01 | 1.79769e+308 | 2.41E-03 |
| NM_153104 | Phospho1 | 0.00E+00 | 1.08E+00 | 1.79769e+308 | 3.68E-03 |
| NM_153489 | Ubap2l | 0.00E+00 | 7.08E-03 | 1.79769e+308 | 1.60E-135 |
| NM_153543 | Aldh1l2 | 0.00E+00 | 3.16E+00 | 1.79769e+308 | 1.31E-04 |
| NM_172287 | Spire2 | 0.00E+00 | 1.19E+00 | 1.79769e+308 | 2.34E-03 |
| NM_172294 | Sulf1 | 0.00E+00 | 7.70E-04 | 1.79769e+308 | 9.14E-05 |
| NM_172301 | Ccnb1 | 0.00E+00 | 1.47E+00 | 1.79769e+308 | 1.59E-03 |
| NM_172610 | Mpped1 | 0.00E+00 | 2.91E+00 | 1.79769e+308 | 2.32E-04 |
| NM_172768 | Gramd1b | 0.00E+00 | 1.37E+00 | 1.79769e+308 | 1.31E-03 |
| NM_172930 | Fam70a | 0.00E+00 | 6.57E-01 | 1.79769e+308 | 3.07E-03 |
| NM_172963 | 1110012J17Rik | 0.00E+00 | 2.89E-01 | 1.79769e+308 | 3.24E-03 |
| NM_174851 | Il28ra | 0.00E+00 | 1.39E+00 | 1.79769e+308 | 5.93E-04 |
| NM_175350 | Tmem146 | 0.00E+00 | 1.10E+00 | 1.79769e+308 | 2.15E-03 |
| NM_175459 | Glis3 | 0.00E+00 | 1.47E+00 | 1.79769e+308 | 5.79E-04 |
| NM_175501 | Adamts12 | 0.00E+00 | 5.13E-01 | 1.79769e+308 | 1.59E-03 |
| NM_175628 | A2m | 0.00E+00 | 3.92E-01 | 1.79769e+308 | 4.18E-03 |
| NM_175833 | Cdv3 | 0.00E+00 | 6.24E+00 | 1.79769e+308 | 1.34E-04 |
| NM_177353 | Slc9a7 | 0.00E+00 | 2.91E+00 | 1.79769e+308 | 3.98E-04 |
| NM_177981 | Hap1 | 0.00E+00 | 1.37E+00 | 1.79769e+308 | 9.97E-04 |
| NM_178362 | Sorbs1 | 0.00E+00 | 2.07E+00 | 1.79769e+308 | 1.51E-04 |
| NM_178444 | Egfl7 | 0.00E+00 | 1.83E+00 | 1.79769e+308 | 2.94E-03 |
| NM_178789 | Tmem117 | 0.00E+00 | 9.80E-01 | 1.79769e+308 | 2.34E-03 |
| NM_181444 | Gprc5a | 0.00E+00 | 1.27E+00 | 1.79769e+308 | 2.55E-03 |
| NM_181544 | Pkd1l3 | 0.00E+00 | 5.53E-01 | 1.79769e+308 | 1.36E-03 |
| NM_181748 | O3far1 | 0.00E+00 | 1.51E+00 | 1.79769e+308 | 3.95E-03 |
| NM_181859 | Oxct2b | 0.00E+00 | 1.43E+00 | 1.79769e+308 | 2.80E-03 |
| NM_182745 | 1700028K03Rik | 0.00E+00 | 3.49E-02 | 1.79769e+308 | 1.91E-28 |
| NM_182841 | Tmem150c | 0.00E+00 | 1.16E+00 | 1.79769e+308 | 1.42E-03 |
| NM_182928 | Adm2 | 0.00E+00 | 4.36E+00 | 1.79769e+308 | 1.22E-03 |
| NM_199036 | Fbxw15 | 0.00E+00 | 1.89E+00 | 1.79769e+308 | 2.41E-03 |
| NM_201389 | Plec | 0.00E+00 | 4.81E+00 | 1.79769e+308 | 9.58E-04 |
| NR_027670 | Pum2 | 0.00E+00 | 8.52E-01 | 1.79769e+308 | 6.50E-04 |
| NR_028095 | Rabggtb | 0.00E+00 | 4.59E+00 | 1.79769e+308 | 4.40E-04 |
| NR_028097 | Rabggtb | 0.00E+00 | 9.76E+00 | 1.79769e+308 | 1.75E-04 |
| NR_028426 | Gm5129 | 0.00E+00 | 1.70E-02 | 1.79769e+308 | 8.15E-117 |
| NR_028432 | 2210013O21Rik | 0.00E+00 | 4.75E+00 | 1.79769e+308 | 1.59E-03 |
| NR_030443 | Mir678 | 0.00E+00 | 2.81E+00 | 1.79769e+308 | 3.62E-08 |
| NR_030710 | Rtel1 | 0.00E+00 | 1.05E-03 | 1.79769e+308 | 2.09E-23 |
| NR_033261 | Gm14492 | 0.00E+00 | 4.01E-02 | 1.79769e+308 | 7.71E-09 |
| NR_033561 | 2900002K06Rik | 0.00E+00 | 9.00E-02 | 1.79769e+308 | 3.78E-101 |
| NR_037679 | 5330439B14Rik | 0.00E+00 | 1.99E-02 | 1.79769e+308 | 3.49E-115 |
| NR_040259 | 2210408F21Rik | 0.00E+00 | 3.34E+00 | 1.79769e+308 | 4.08E-03 |
| NR_040279 | A730090H04Rik | 0.00E+00 | 1.82E+00 | 1.79769e+308 | 2.12E-03 |
| NR_040309 | 2310069G16Rik | 0.00E+00 | 9.63E-02 | 1.79769e+308 | 5.91E-10 |
| NR_040487 | 1810053B23Rik | 0.00E+00 | 1.87E-02 | 1.79769e+308 | 4.08E-03 |
| NR_040528 | Gm9926 | 0.00E+00 | 3.82E-01 | 1.79769e+308 | 3.95E-03 |
| NR_040679 | 2310034O05Rik | 0.00E+00 | 5.00E+01 | 1.79769e+308 | 3.34E-04 |
| NR_040767 | NA | 0.00E+00 | 1.99E+00 | 1.79769e+308 | 1.98E-03 |
| NM_001177852 | Asph | 5.79E-03 | 1.11E+01 | 7.56E+00 | 0.00E+00 |
| NM_001080390 | Mark2 | 1.22E-03 | 2.26E+00 | 7.52E+00 | 0.00E+00 |
| NM_001164039 | Ly6e | 4.88E-02 | 8.54E+01 | 7.47E+00 | 0.00E+00 |
| NM_001160222 | Pum2 | 3.42E-03 | 5.80E+00 | 7.44E+00 | 0.00E+00 |
| NM_016697 | Gpc3 | 1.84E-01 | 1.81E+02 | 6.89E+00 | 4.63E-09 |
| NM_001136227 | Rtkn | 1.06E-02 | 9.89E+00 | 6.84E+00 | 0.00E+00 |
| NM_001177630 | Etl4 | 7.12E-04 | 4.70E-01 | 6.49E+00 | 0.00E+00 |
| NM_001164360 | Erlin1 | 5.32E-02 | 2.81E+01 | 6.27E+00 | 0.00E+00 |
| NM_001160017 | Gnb1 | 4.60E-02 | 2.43E+01 | 6.27E+00 | 0.00E+00 |
| NM_011106 | Pkig | 1.18E-02 | 6.10E+00 | 6.25E+00 | 0.00E+00 |
| NM_001085453 | Ctnnd1 | 2.91E-02 | 1.50E+01 | 6.25E+00 | 0.00E+00 |
| NM_001025562 | Pus1 | 1.55E-02 | 7.71E+00 | 6.21E+00 | 0.00E+00 |
| NM_001168513 | Mapk14 | 4.53E-02 | 2.04E+01 | 6.11E+00 | 0.00E+00 |
| NM_015742 | Myo9b | 1.16E-02 | 5.20E+00 | 6.11E+00 | 0.00E+00 |
| NM_021437 | 1700123O20Rik | 2.63E-02 | 1.13E+01 | 6.06E+00 | 0.00E+00 |
| NM_008449 | Kif5c | 5.73E-02 | 2.36E+01 | 6.02E+00 | 2.52E-08 |
| NM_001085449 | Ctnnd1 | 5.01E-02 | 2.05E+01 | 6.02E+00 | 0.00E+00 |
| NM_001198824 | App | 5.55E-02 | 2.15E+01 | 5.96E+00 | 0.00E+00 |
| NR_038053 | 4930579G18Rik | 8.90E-04 | 3.08E-01 | 5.85E+00 | 0.00E+00 |
| NM_001160304 | Gm4788 | 4.38E-02 | 1.52E+01 | 5.85E+00 | 0.00E+00 |
| NR_027373 | Arhgap1 | 5.18E-03 | 1.67E+00 | 5.78E+00 | 0.00E+00 |
| NM_007423 | Afp | 4.04E-01 | 1.14E+02 | 5.64E+00 | 4.37E-11 |
| NM_001045964 | Mink1 | 5.14E-03 | 1.35E+00 | 5.57E+00 | 0.00E+00 |
| NM_001146124 | Psap | 8.11E-02 | 1.96E+01 | 5.49E+00 | 0.00E+00 |
| NM_001164733 | Mpp6 | 6.13E-02 | 1.41E+01 | 5.44E+00 | 0.00E+00 |
| NM_001159606 | Pum1 | 2.05E-03 | 4.46E-01 | 5.38E+00 | 0.00E+00 |
| NM_025452 | Tmem54 | 2.13E-01 | 3.99E+01 | 5.24E+00 | 7.03E-07 |
| NM_001205025 | Jagn1 | 4.66E-02 | 8.75E+00 | 5.23E+00 | 0.00E+00 |
| NM_010716 | Lig3 | 3.33E-02 | 6.04E+00 | 5.20E+00 | 0.00E+00 |
| NM_001114125 | Dab2ip | 2.65E-03 | 4.65E-01 | 5.17E+00 | 0.00E+00 |
| NM_001083808 | Rusc1 | 6.95E-03 | 1.10E+00 | 5.07E+00 | 0.00E+00 |
| NM_011323 | Scn8a | 1.72E-02 | 2.71E+00 | 5.06E+00 | 1.59E-05 |
| NM_001077499 | Scn8a | 1.72E-02 | 2.71E+00 | 5.06E+00 | 1.55E-05 |
| NR_037707 | Adamts10 | 4.45E-03 | 6.41E-01 | 4.97E+00 | 0.00E+00 |
| NM_001197322 | Foxp1 | 5.14E-03 | 7.35E-01 | 4.96E+00 | 0.00E+00 |
| NM_172498 | Ptk2b | 1.32E-02 | 1.88E+00 | 4.96E+00 | 0.00E+00 |
| NM_011614 | Tnfsf12 | 4.14E-02 | 5.88E+00 | 4.96E+00 | 0.00E+00 |
| NM_001177793 | Snap23 | 3.54E-02 | 5.01E+00 | 4.95E+00 | 0.00E+00 |
| NM_001166459 | Brcc3 | 6.29E-02 | 8.72E+00 | 4.93E+00 | 0.00E+00 |
| NM_001025393 | Bclaf1 | 2.24E-02 | 3.10E+00 | 4.93E+00 | 0.00E+00 |
| NM_001039086 | Rapgef1 | 2.78E-02 | 3.84E+00 | 4.93E+00 | 0.00E+00 |
| NM_029021 | 4833422F24Rik | 2.17E-01 | 2.97E+01 | 4.92E+00 | 2.41E-06 |
| NM_011938 | Grk6 | 4.51E-03 | 5.96E-01 | 4.88E+00 | 0.00E+00 |
| NR_033185 | Rcbtb2 | 1.15E-02 | 1.50E+00 | 4.87E+00 | 4.21E-05 |
| NM_001085408 | Sdccag3 | 1.19E-01 | 1.54E+01 | 4.86E+00 | 0.00E+00 |
| NM_177420 | Psat1 | 1.87E-01 | 2.38E+01 | 4.85E+00 | 1.14E-03 |
| NM_029427 | Pnpla5 | 5.10E-01 | 6.13E+01 | 4.79E+00 | 8.76E-10 |
| NM_001168508 | Mapk14 | 2.44E-02 | 2.90E+00 | 4.78E+00 | 0.00E+00 |
| NM_001159556 | Cd36 | 1.52E-01 | 1.75E+01 | 4.74E+00 | 3.81E-07 |
| NM_009258 | Spink3 | 1.74E+00 | 1.94E+02 | 4.71E+00 | 5.99E-06 |
| NM_008687 | Nfib | 2.75E-02 | 2.94E+00 | 4.67E+00 | 0.00E+00 |
| NM_001168680 | Tspan8 | 1.04E+00 | 1.10E+02 | 4.66E+00 | 8.66E-04 |
| NM_001085472 | Acin1 | 2.18E-02 | 2.16E+00 | 4.59E+00 | 0.00E+00 |
| NM_001166596 | Eif5a | 3.75E-01 | 3.61E+01 | 4.57E+00 | 6.15E-06 |
| NM_001159418 | Irf9 | 3.64E-02 | 3.34E+00 | 4.52E+00 | 0.00E+00 |
| NR_028296 | Fance | 5.56E-03 | 5.06E-01 | 4.51E+00 | 0.00E+00 |
| NM_001159574 | Fip1l1 | 2.39E-02 | 2.06E+00 | 4.46E+00 | 0.00E+00 |
| NM_001177654 | Nelf | 1.94E-02 | 1.62E+00 | 4.42E+00 | 0.00E+00 |
| NM_007559 | Bmp8b | 1.39E-01 | 1.10E+01 | 4.38E+00 | 2.52E-05 |
| NM_001177856 | Asph | 1.17E-02 | 9.13E-01 | 4.36E+00 | 0.00E+00 |
| NM_001177655 | Nelf | 1.81E-02 | 1.33E+00 | 4.30E+00 | 0.00E+00 |
| NM_023223 | Cdc20 | 2.58E-02 | 1.84E+00 | 4.26E+00 | 0.00E+00 |
| NM_020276 | Nelf | 2.31E-02 | 1.55E+00 | 4.21E+00 | 0.00E+00 |
| NM_194342 | Sun2 | 2.37E-02 | 1.58E+00 | 4.20E+00 | 0.00E+00 |
| NM_001146123 | Psap | 3.62E-02 | 2.26E+00 | 4.13E+00 | 0.00E+00 |
| NM_001163820 | Fance | 8.67E-03 | 5.29E-01 | 4.11E+00 | 3.11E-15 |
| NM_029811 | St5 | 5.31E-03 | 3.11E-01 | 4.07E+00 | 0.00E+00 |
| NM_175454 | C630004H02Rik | 2.51E-01 | 1.47E+01 | 4.07E+00 | 1.07E-07 |
| NM_001136260 | Slc4a4 | 9.28E-03 | 5.40E-01 | 4.06E+00 | 0.00E+00 |
| NM_009662 | Alox5 | 1.45E-01 | 8.26E+00 | 4.04E+00 | 9.68E-05 |
| NM_001177667 | Spna2 | 6.96E-01 | 3.86E+01 | 4.02E+00 | 4.96E-08 |
| NM_199011 | Dgkq | 3.55E-02 | 1.84E+00 | 3.95E+00 | 0.00E+00 |
| NM_008396 | Itga2 | 9.47E-02 | 4.89E+00 | 3.94E+00 | 1.42E-04 |
| NM_001161457 | Cbfb | 1.26E-02 | 6.40E-01 | 3.93E+00 | 0.00E+00 |
| NM_001161456 | Cbfb | 1.68E-02 | 8.47E-01 | 3.92E+00 | 0.00E+00 |
| NM_172308 | Mthfd1l | 1.63E-01 | 7.80E+00 | 3.86E+00 | 2.08E-03 |
| NM_001171005 | Prkd3 | 1.48E-01 | 6.99E+00 | 3.86E+00 | 7.32E-07 |
| NM_001159605 | Pum1 | 3.26E-03 | 1.47E-01 | 3.81E+00 | 4.44E-16 |
| NM_007670 | Cdkn2b | 6.39E-01 | 2.88E+01 | 3.81E+00 | 5.58E-07 |
| NM_178591 | Nrg1 | 2.00E-01 | 9.01E+00 | 3.81E+00 | 2.42E-04 |
| NM_030236 | Fbxo34 | 2.29E-02 | 1.01E+00 | 3.79E+00 | 0.00E+00 |
| NM_001048227 | Dbndd2 | 2.42E-02 | 1.06E+00 | 3.78E+00 | 2.22E-16 |
| NM_033324 | Dgcr8 | 6.84E-02 | 2.99E+00 | 3.78E+00 | 0.00E+00 |
| NM_001164608 | Maf1 | 1.50E-02 | 6.52E-01 | 3.77E+00 | 1.33E-15 |
| NM_001163422 | Tatdn3 | 1.47E-02 | 6.16E-01 | 3.74E+00 | 7.31E-04 |
| NM_001033304 | 5330417C22Rik | 6.09E-01 | 2.50E+01 | 3.72E+00 | 2.43E-09 |
| NM_001024539 | Shc2 | 2.03E-01 | 8.29E+00 | 3.71E+00 | 1.04E-06 |
| NM_010213 | Fhl3 | 5.13E-01 | 2.05E+01 | 3.69E+00 | 1.28E-06 |
| NM_176921 | 6030419C18Rik | 1.96E-01 | 7.75E+00 | 3.67E+00 | 3.95E-04 |
| NM_001112698 | Ngf | 1.03E+00 | 4.08E+01 | 3.67E+00 | 1.84E-03 |
| NM_009400 | Tnfrsf18 | 4.73E-01 | 1.85E+01 | 3.67E+00 | 4.05E-04 |
| NM_020043 | Igdcc4 | 3.16E-01 | 1.22E+01 | 3.66E+00 | 4.52E-09 |
| NM_138631 | Tssc4 | 1.59E-02 | 6.04E-01 | 3.64E+00 | 4.60E-10 |
| NM_009622 | Adcy1 | 9.46E-02 | 3.59E+00 | 3.64E+00 | 1.83E-08 |
| NM_001079695 | Srsf5 | 3.56E-01 | 1.32E+01 | 3.61E+00 | 6.22E-05 |
| NM_001146121 | Psap | 2.08E+00 | 7.67E+01 | 3.61E+00 | 2.08E-09 |
| NM_001160235 | Fam168b | 1.56E-02 | 5.73E-01 | 3.61E+00 | 0.00E+00 |
| NM_001109990 | D0H4S114 | 1.87E-02 | 6.73E-01 | 3.58E+00 | 4.32E-04 |
| NM_011179 | Psap | 2.09E+00 | 7.46E+01 | 3.58E+00 | 2.39E-09 |
| NM_001159590 | Sirt1 | 4.50E-02 | 1.59E+00 | 3.57E+00 | 0.00E+00 |
| NM_021042 | Abcc9 | 2.76E-03 | 9.76E-02 | 3.56E+00 | 4.62E-14 |
| NM_016782 | Cntnap1 | 1.47E-01 | 5.07E+00 | 3.54E+00 | 3.17E-06 |
| NM_025629 | Adamtsl5 | 1.27E-01 | 4.34E+00 | 3.53E+00 | 8.22E-04 |
| NM_011373 | St6galnac4 | 3.21E-02 | 1.09E+00 | 3.52E+00 | 0.00E+00 |
| NM_013658 | Sema4a | 7.64E-01 | 2.59E+01 | 3.52E+00 | 3.79E-09 |
| NM_011486 | Stat3 | 3.86E-02 | 1.30E+00 | 3.52E+00 | 0.00E+00 |
| NM_001163522 | Emcn | 3.07E-01 | 1.02E+01 | 3.51E+00 | 6.70E-04 |
| NM_030682 | Tlr1 | 1.29E+00 | 4.10E+01 | 3.46E+00 | 2.03E-09 |
| NM_001113548 | Adamtsl5 | 1.27E-01 | 3.94E+00 | 3.43E+00 | 9.73E-04 |
| NM_175093 | Trib3 | 4.12E-01 | 1.26E+01 | 3.42E+00 | 6.60E-06 |
| NM_001164677 | Pdcd6ip | 2.74E-02 | 8.31E-01 | 3.41E+00 | 0.00E+00 |
| NM_008750 | Nxn | 4.52E-01 | 1.35E+01 | 3.40E+00 | 1.21E-07 |
| NM_026422 | Mrrf | 2.11E-01 | 6.23E+00 | 3.39E+00 | 5.40E-04 |
| NM_001164525 | Tstd1 | 1.31E+01 | 3.89E+02 | 3.39E+00 | 5.64E-09 |
| NM_023647 | Nipa2 | 1.19E-01 | 3.50E+00 | 3.38E+00 | 7.68E-04 |
| NM_025638 | Gdpd1 | 3.33E-01 | 9.68E+00 | 3.37E+00 | 8.95E-06 |
| NM_023612 | Esm1 | 8.01E-01 | 2.30E+01 | 3.36E+00 | 1.64E-07 |
| NM_010704 | Lepr | 2.15E-01 | 6.15E+00 | 3.35E+00 | 2.30E-05 |
| NM_138303 | Yipf2 | 1.83E-01 | 5.14E+00 | 3.33E+00 | 0.00E+00 |
| NM_001165954 | Phc3 | 7.01E-02 | 1.96E+00 | 3.33E+00 | 2.69E-04 |
| NM_054088 | Pnpla3 | 5.13E-01 | 1.40E+01 | 3.31E+00 | 2.04E-08 |
| NM_008872 | Plat | 1.63E-01 | 4.44E+00 | 3.30E+00 | 1.47E-03 |
| NM_148958 | Osbpl10 | 2.28E-01 | 6.15E+00 | 3.30E+00 | 1.51E-03 |
| NM_001033364 | Cdhr2 | 9.69E-02 | 2.60E+00 | 3.29E+00 | 1.55E-03 |
| NM_028137 | 5730528L13Rik | 8.77E-02 | 2.32E+00 | 3.28E+00 | 1.91E-03 |
| NM_001037493 | Slc41a3 | 8.33E-01 | 2.18E+01 | 3.27E+00 | 1.41E-07 |
| NM_009636 | Aebp1 | 5.75E-01 | 1.51E+01 | 3.27E+00 | 4.15E-08 |
| NM_153800 | Arhgap22 | 4.76E-01 | 1.25E+01 | 3.26E+00 | 3.66E-07 |
| NM_001145925 | 5730528L13Rik | 9.15E-02 | 2.39E+00 | 3.26E+00 | 1.98E-03 |
| NM_011212 | Ptpre | 2.47E-01 | 6.32E+00 | 3.24E+00 | 7.67E-07 |
| NM_001243132 | Tspan2 | 2.40E-01 | 6.08E+00 | 3.23E+00 | 4.21E-03 |
| NM_001164567 | Vill | 2.94E-01 | 7.43E+00 | 3.23E+00 | 4.12E-03 |
| NM_001035122 | Golm1 | 1.28E+00 | 3.23E+01 | 3.23E+00 | 8.48E-09 |
| NM_025439 | Tmem9 | 2.26E-01 | 5.68E+00 | 3.22E+00 | 0.00E+00 |
| NM_028805 | Katnb1 | 2.88E-02 | 7.24E-01 | 3.22E+00 | 6.66E-15 |
| NM_001159482 | Rab34 | 3.31E-01 | 8.32E+00 | 3.22E+00 | 5.35E-07 |
| NM_001205339 | Psat1 | 1.88E-01 | 4.70E+00 | 3.22E+00 | 1.67E-03 |
| NM_010101 | S1pr3 | 3.55E-01 | 8.81E+00 | 3.21E+00 | 5.02E-07 |
| NM_001085448 | Ctnnd1 | 2.62E-02 | 6.46E-01 | 3.21E+00 | 0.00E+00 |
| NM_183142 | Alg11 | 1.77E-02 | 4.35E-01 | 3.20E+00 | 0.00E+00 |
| NM_029770 | Unc5b | 8.06E-01 | 1.94E+01 | 3.18E+00 | 1.20E-08 |
| NM_029315 | Pou5f2 | 5.46E-03 | 1.31E-01 | 3.18E+00 | 1.83E-05 |
| NM_145134 | Spsb4 | 1.87E-01 | 4.34E+00 | 3.14E+00 | 2.52E-03 |
| NM_207624 | Ace | 3.85E-01 | 8.89E+00 | 3.14E+00 | 2.50E-07 |
| NM_153164 | Cnot1 | 1.67E-01 | 3.82E+00 | 3.13E+00 | 1.45E-06 |
| NM_019468 | G6pd2 | 2.66E-01 | 6.06E+00 | 3.13E+00 | 2.63E-03 |
| NM_010742 | Ly6d | 3.35E+01 | 7.58E+02 | 3.12E+00 | 9.27E-06 |
| NM_007468 | Apoa4 | 2.67E+02 | 6.01E+03 | 3.11E+00 | 0.00E+00 |
| NM_016859 | Bysl | 1.10E-01 | 2.44E+00 | 3.10E+00 | 2.23E-03 |
| NM_007653 | Cd63 | 3.71E+00 | 8.23E+01 | 3.10E+00 | 7.87E-08 |
| NR_033564 | Gm16897 | 1.93E-02 | 4.16E-01 | 3.07E+00 | 1.82E-09 |
| NM_011575 | Tff3 | 1.94E+01 | 4.17E+02 | 3.07E+00 | 3.31E-08 |
| NM_010917 | Nid1 | 3.25E+00 | 6.89E+01 | 3.05E+00 | 2.54E-05 |
| NM_194069 | Ifi27l1 | 4.84E-01 | 1.01E+01 | 3.03E+00 | 0.00E+00 |
| NM_134090 | Kdelr3 | 4.66E-01 | 9.60E+00 | 3.03E+00 | 4.85E-04 |
| NM_001040426 | Thsd4 | 1.46E-01 | 3.00E+00 | 3.02E+00 | 4.33E-06 |
| NM_053195 | Slc24a3 | 2.36E-01 | 4.82E+00 | 3.01E+00 | 7.07E-05 |
| NM_011178 | Prtn3 | 4.96E-01 | 1.01E+01 | 3.01E+00 | 3.79E-03 |
| NM_016773 | Nucb2 | 8.03E-01 | 1.63E+01 | 3.01E+00 | 8.34E-06 |
| NM_001130164 | Oxr1 | 1.61E-01 | 3.26E+00 | 3.01E+00 | 0.00E+00 |
| NM_177259 | Dab1 | 7.47E-02 | 1.50E+00 | 3.00E+00 | 3.94E-03 |
| NM_172945 | Ankrd13b | 2.43E-01 | 4.84E+00 | 2.99E+00 | 8.04E-05 |
| NM_001146026 | Rnf44 | 2.02E-01 | 3.99E+00 | 2.98E+00 | 9.06E-05 |
| NM_178699 | B930041F14Rik | 1.96E-01 | 3.83E+00 | 2.97E+00 | 4.35E-03 |
| NM_029922 | Parp6 | 9.73E-02 | 1.89E+00 | 2.97E+00 | 6.66E-16 |
| NM_001001179 | BC048546 | 2.03E+00 | 3.92E+01 | 2.96E+00 | 1.48E-07 |
| NM_001163553 | Ap4b1 | 2.35E-02 | 4.50E-01 | 2.95E+00 | 1.11E-11 |
| NM_011604 | Tlr6 | 3.17E-01 | 5.92E+00 | 2.93E+00 | 1.16E-04 |
| NR_038074 | Parp6 | 1.01E-01 | 1.88E+00 | 2.92E+00 | 2.44E-15 |
| NM_172471 | Itih5 | 3.54E+00 | 6.57E+01 | 2.92E+00 | 2.76E-03 |
| NM_019926 | Mtm1 | 2.25E-01 | 4.09E+00 | 2.90E+00 | 2.05E-04 |
| NM_028710 | Arsg | 8.36E-01 | 1.51E+01 | 2.89E+00 | 4.07E-07 |
| NM_175130 | Trpm4 | 1.88E-01 | 3.37E+00 | 2.88E+00 | 1.45E-04 |
| NM_021486 | Bcmo1 | 2.20E+00 | 3.88E+01 | 2.87E+00 | 8.29E-08 |
| NM_182782 | Klhl25 | 1.54E-01 | 2.72E+00 | 2.87E+00 | 0.00E+00 |
| NM_010634 | Fabp5 | 1.79E+01 | 3.10E+02 | 2.85E+00 | 1.82E-06 |
| NM_010492 | Ica1 | 4.06E-01 | 6.97E+00 | 2.84E+00 | 1.82E-04 |
| NM_145760 | Arfgap1 | 4.12E-01 | 7.00E+00 | 2.83E+00 | 3.03E-05 |
| NM_001081171 | Lama5 | 3.74E-01 | 6.29E+00 | 2.82E+00 | 1.52E-07 |
| NR_003522 | Abhd1 | 5.38E-01 | 8.96E+00 | 2.81E+00 | 7.24E-04 |
| NM_011035 | Pak1 | 5.32E-01 | 8.87E+00 | 2.81E+00 | 9.50E-06 |
| NM_019737 | B4galt6 | 9.50E-01 | 1.57E+01 | 2.80E+00 | 1.34E-07 |
| NM_134064 | Rnf44 | 2.43E-01 | 3.88E+00 | 2.77E+00 | 9.55E-05 |
| NM_001130479 | Nucb2 | 7.90E-01 | 1.26E+01 | 2.77E+00 | 3.72E-05 |
| NR_028094 | Rabggtb | 4.09E-01 | 6.48E+00 | 2.76E+00 | 2.70E-03 |
| NM_001035525 | AU040320 | 4.46E-01 | 6.76E+00 | 2.72E+00 | 1.50E-05 |
| NR_015574 | 4930513N10Rik | 1.47E-02 | 2.17E-01 | 2.69E+00 | 3.88E-08 |
| NM_025284 | Tmsb10 | 1.89E+00 | 2.74E+01 | 2.67E+00 | 8.00E-04 |
| NM_178618 | Fam83g | 1.73E-01 | 2.51E+00 | 2.67E+00 | 3.40E-04 |
| NM_001130170 | Zfp207 | 6.31E-01 | 9.10E+00 | 2.67E+00 | 3.95E-05 |
| NM_001034964 | Sorbs1 | 9.46E-01 | 1.35E+01 | 2.66E+00 | 4.51E-07 |
| NM_001035526 | AU040320 | 4.41E-01 | 6.16E+00 | 2.64E+00 | 2.20E-05 |
| NM_001190161 | Psrc1 | 3.38E-01 | 4.72E+00 | 2.64E+00 | 5.48E-06 |
| NM_029001 | Elovl7 | 2.18E-01 | 3.04E+00 | 2.63E+00 | 3.77E-05 |
| NM_001242365 | Fam160a2 | 5.20E-02 | 7.24E-01 | 2.63E+00 | 2.80E-09 |
| NM_019501 | Pdss1 | 6.41E-02 | 8.88E-01 | 2.63E+00 | 1.96E-10 |
| NM_001043322 | Fmn1 | 5.54E-01 | 7.62E+00 | 2.62E+00 | 6.96E-04 |
| NM_001163420 | Fam172a | 9.43E-03 | 1.28E-01 | 2.61E+00 | 1.63E-06 |
| NM_001174073 | Pcbp2 | 5.80E-01 | 7.85E+00 | 2.60E+00 | 5.19E-05 |
| NM_172887 | Fry | 1.08E-01 | 1.43E+00 | 2.58E+00 | 5.37E-05 |
| NM_007585 | Anxa2 | 1.36E+01 | 1.80E+02 | 2.58E+00 | 8.43E-06 |
| NM_133754 | Fblim1 | 4.65E-01 | 6.07E+00 | 2.57E+00 | 7.13E-05 |
| NM_001081396 | Wdr67 | 1.93E+00 | 2.51E+01 | 2.56E+00 | 7.12E-07 |
| NM_007614 | Ctnnb1 | 2.26E+00 | 2.93E+01 | 2.56E+00 | 7.84E-07 |
| NM_007633 | Ccne1 | 8.68E-01 | 1.12E+01 | 2.56E+00 | 5.53E-05 |
| NM_009741 | Bcl2 | 9.44E-02 | 1.18E+00 | 2.53E+00 | 1.81E-03 |
| NM_001043228 | Dntt | 3.01E-01 | 3.74E+00 | 2.52E+00 | 1.02E-04 |
| NM_029502 | Cant1 | 3.19E-01 | 3.91E+00 | 2.50E+00 | 4.63E-04 |
| NM_001024720 | Hmcn1 | 4.20E-02 | 5.08E-01 | 2.49E+00 | 1.06E-03 |
| NM_016693 | Map3k6 | 4.60E-01 | 5.55E+00 | 2.49E+00 | 3.39E-05 |
| NM_080419 | Igsf8 | 1.69E+00 | 2.03E+01 | 2.49E+00 | 4.27E-06 |
| NM_027196 | Pold4 | 1.39E+01 | 1.67E+02 | 2.49E+00 | 1.42E-06 |
| NM_001029985 | Kcp | 6.49E-01 | 7.79E+00 | 2.49E+00 | 5.42E-06 |
| NM_001163155 | Col4a5 | 7.40E-02 | 8.83E-01 | 2.48E+00 | 1.31E-03 |
| NM_030249 | Cttnbp2nl | 2.90E-02 | 3.46E-01 | 2.48E+00 | 8.37E-09 |
| NM_033269 | Chrm3 | 2.56E-01 | 3.05E+00 | 2.48E+00 | 1.17E-03 |
| NM_001243050 | Atp6v0a1 | 2.97E+00 | 3.52E+01 | 2.47E+00 | 3.20E-06 |
| NM_133834 | Hnrnpf | 1.31E+00 | 1.55E+01 | 2.47E+00 | 1.24E-05 |
| NM_001110337 | Gprc5c | 2.63E+00 | 3.09E+01 | 2.46E+00 | 1.70E-06 |
| NM_001081066 | Dennd3 | 1.49E-01 | 1.74E+00 | 2.46E+00 | 1.27E-03 |
| NM_001045489 | Mfge8 | 6.65E+00 | 7.66E+01 | 2.44E+00 | 3.39E-06 |
| NM_133871 | Ifi44 | 4.22E-01 | 4.85E+00 | 2.44E+00 | 1.36E-04 |
| NM_001081127 | Adamts14 | 1.53E-01 | 1.75E+00 | 2.44E+00 | 1.38E-03 |
| NR_002860 | A130040M12Rik | 1.02E+01 | 1.16E+02 | 2.44E+00 | 3.77E-04 |
| NM_001029895 | Ate1 | 1.06E+00 | 1.21E+01 | 2.43E+00 | 1.57E-04 |
| NM_009997 | Cyp2a4 | 3.20E+02 | 3.63E+03 | 2.43E+00 | 1.87E-11 |
| NM_013565 | Itga3 | 7.36E-01 | 8.34E+00 | 2.43E+00 | 6.72E-06 |
| NM_009285 | Stc1 | 3.22E-01 | 3.64E+00 | 2.42E+00 | 1.53E-04 |
| NM_001146025 | Rnf44 | 7.47E-02 | 8.39E-01 | 2.42E+00 | 6.92E-12 |
| NM_001146350 | Eng | 1.35E+00 | 1.51E+01 | 2.41E+00 | 3.40E-06 |
| NM_172916 | Hydin | 4.88E-02 | 5.39E-01 | 2.40E+00 | 1.65E-03 |
| NM_001202500 | Armcx4 | 2.44E-01 | 2.69E+00 | 2.40E+00 | 1.65E-03 |
| NM_178743 | Slc26a11 | 3.06E-01 | 3.37E+00 | 2.40E+00 | 1.65E-03 |
| NM_013790 | Abcc5 | 6.11E-01 | 6.73E+00 | 2.40E+00 | 8.46E-06 |
| NM_026681 | Ccdc88c | 3.12E-01 | 3.40E+00 | 2.39E+00 | 6.91E-05 |
| NM_011990 | Slc7a11 | 6.37E-01 | 6.92E+00 | 2.39E+00 | 2.57E-06 |
| NM_008605 | Mmp12 | 3.35E-01 | 3.62E+00 | 2.38E+00 | 2.07E-04 |
| NM_001168220 | 2310008H09Rik | 5.84E-01 | 6.29E+00 | 2.38E+00 | 2.02E-05 |
| NR_037965 | Gm6644 | 1.12E+00 | 1.20E+01 | 2.37E+00 | 2.82E-04 |
| NM_001242363 | Fam160a2 | 1.93E-01 | 2.06E+00 | 2.37E+00 | 4.08E-03 |
| NM_182807 | Fam19a2 | 1.93E-01 | 2.03E+00 | 2.35E+00 | 2.08E-03 |
| NM_025565 | Spc25 | 1.18E-01 | 1.24E+00 | 2.35E+00 | 6.60E-07 |
| NM_007426 | Angpt2 | 6.31E-01 | 6.56E+00 | 2.34E+00 | 2.79E-04 |
| NM_020028 | Lpar2 | 2.07E-01 | 2.10E+00 | 2.32E+00 | 3.02E-04 |
| NM_134448 | Dst | 8.16E-01 | 8.28E+00 | 2.32E+00 | 2.43E-05 |
| NM_008381 | Inhbb | 7.51E-01 | 7.58E+00 | 2.31E+00 | 2.19E-05 |
| NM_019702 | Hbs1l | 1.35E+00 | 1.36E+01 | 2.31E+00 | 1.67E-05 |
| NM_001190449 | Ddah2 | 3.35E-01 | 3.36E+00 | 2.31E+00 | 3.11E-03 |
| NM_011994 | Abcd2 | 4.21E+00 | 4.22E+01 | 2.30E+00 | 6.59E-05 |
| NM_133733 | 9030425E11Rik | 6.97E-01 | 6.94E+00 | 2.30E+00 | 1.31E-04 |
| NM_019741 | Slc2a5 | 9.20E-01 | 8.99E+00 | 2.28E+00 | 4.36E-05 |
| NM_001164252 | Tpm1 | 1.83E+00 | 1.79E+01 | 2.28E+00 | 4.00E-05 |
| NR_035432 | Mir1902 | 5.62E+01 | 5.49E+02 | 2.28E+00 | 2.45E-05 |
| NM_001025600 | Cadm1 | 4.36E-02 | 4.24E-01 | 2.27E+00 | 1.64E-08 |
| NM_001164253 | Tpm1 | 1.84E+00 | 1.78E+01 | 2.27E+00 | 4.14E-05 |
| NM_133772 | Ssbp4 | 8.97E-01 | 8.72E+00 | 2.27E+00 | 3.95E-04 |
| NM_025980 | Nrarp | 3.21E-01 | 3.09E+00 | 2.27E+00 | 3.06E-03 |
| NM_009166 | Sorbs1 | 2.53E-01 | 2.43E+00 | 2.26E+00 | 4.89E-04 |
| NM_011322 | Scn1b | 3.48E+00 | 3.33E+01 | 2.26E+00 | 1.23E-05 |
| NM_015730 | Chrna4 | 8.08E-01 | 7.70E+00 | 2.25E+00 | 2.72E-05 |
| NM_001166594 | Eif5a | 3.15E+00 | 3.00E+01 | 2.25E+00 | 2.65E-05 |
| NM_001113517 | Arhgef7 | 5.17E-02 | 4.92E-01 | 2.25E+00 | 1.57E-08 |
| NM_001243051 | Atp6v0a1 | 1.14E+00 | 1.08E+01 | 2.25E+00 | 1.46E-05 |
| NM_175751 | Zfp608 | 1.93E-01 | 1.82E+00 | 2.24E+00 | 4.68E-04 |
| NM_001199967 | Gm11127 | 3.09E-01 | 2.90E+00 | 2.24E+00 | 3.16E-06 |
| NM_134038 | Slc16a6 | 6.58E-01 | 6.16E+00 | 2.24E+00 | 6.60E-05 |
| NM_019782 | Lepre1 | 5.00E-01 | 4.66E+00 | 2.23E+00 | 1.00E-03 |
| NM_001168623 | Znrf1 | 1.92E-02 | 1.79E-01 | 2.23E+00 | 3.98E-06 |
| NM_025519 | Chmp4c | 7.19E-01 | 6.68E+00 | 2.23E+00 | 5.11E-04 |
| NM_178929 | Kazald1 | 9.70E-01 | 9.00E+00 | 2.23E+00 | 5.11E-04 |
| NM_173182 | Fndc3b | 4.22E+00 | 3.90E+01 | 2.23E+00 | 2.24E-04 |
| NM_027881 | Osbpl3 | 1.09E+00 | 1.01E+01 | 2.22E+00 | 3.68E-03 |
| NM_011838 | Lynx1 | 2.03E-01 | 1.87E+00 | 2.22E+00 | 3.75E-03 |
| NM_022420 | Gprc5b | 7.49E-01 | 6.90E+00 | 2.22E+00 | 3.88E-05 |
| NM_027928 | Chst13 | 5.25E-01 | 4.84E+00 | 2.22E+00 | 3.75E-03 |
| NM_146115 | A830007P12Rik | 1.06E+00 | 9.70E+00 | 2.21E+00 | 3.66E-05 |
| NR_024509 | Tmem80 | 2.73E-01 | 2.48E+00 | 2.20E+00 | 4.38E-07 |
| NM_001185020 | Wnk1 | 7.49E-01 | 6.74E+00 | 2.20E+00 | 9.32E-06 |
| NR_038062 | Yipf2 | 6.98E-01 | 6.26E+00 | 2.19E+00 | 1.08E-03 |
| NM_026167 | Klhl13 | 1.89E+00 | 1.69E+01 | 2.19E+00 | 1.38E-05 |
| NM_177013 | Tmem229a | 1.59E-01 | 1.41E+00 | 2.19E+00 | 4.37E-03 |
| NM_028950 | Nsun6 | 6.54E-02 | 5.77E-01 | 2.18E+00 | 5.62E-05 |
| NM_025446 | Aig1 | 4.92E+00 | 4.32E+01 | 2.17E+00 | 2.86E-05 |
| NM_031874 | Rab3d | 2.08E+00 | 1.82E+01 | 2.17E+00 | 1.18E-05 |
| NM_011571 | Tesk1 | 8.09E-02 | 7.09E-01 | 2.17E+00 | 1.12E-08 |
| NM_172856 | Lass6 | 2.96E+00 | 2.59E+01 | 2.17E+00 | 1.00E-05 |
| NM_008594 | Mfge8 | 2.04E+00 | 1.78E+01 | 2.17E+00 | 3.37E-05 |
| NM_001190445 | 2610002J02Rik | 1.22E+00 | 1.05E+01 | 2.16E+00 | 1.53E-03 |
| NM_008397 | Itga6 | 1.05E+00 | 8.94E+00 | 2.15E+00 | 3.66E-05 |
| NM_177466 | Rab11fip5 | 9.95E-02 | 8.50E-01 | 2.15E+00 | 1.31E-03 |
| NM_174996 | D4Ertd22e | 1.54E+00 | 1.31E+01 | 2.14E+00 | 3.02E-05 |
| NM_178589 | Tnfrsf21 | 2.00E+00 | 1.69E+01 | 2.13E+00 | 1.75E-05 |
| NM_009594 | Abl1 | 5.02E-01 | 4.22E+00 | 2.13E+00 | 1.08E-04 |
| NM_001167679 | Wdr67 | 7.13E-01 | 5.98E+00 | 2.13E+00 | 1.90E-04 |
| NM_001164167 | Pias2 | 5.62E-03 | 4.67E-02 | 2.12E+00 | 8.06E-04 |
| NM_011607 | Tnc | 2.20E-01 | 1.83E+00 | 2.12E+00 | 8.66E-04 |
| NM_007657 | Cd9 | 9.62E+00 | 7.98E+01 | 2.12E+00 | 1.51E-05 |
| NM_011242 | Rasgrp2 | 4.01E+00 | 3.32E+01 | 2.11E+00 | 1.71E-05 |
| NM_008797 | Pcx | 6.31E+00 | 5.19E+01 | 2.11E+00 | 1.20E-04 |
| NM_001162996 | Eml2 | 7.32E-01 | 6.01E+00 | 2.11E+00 | 1.00E-03 |
| NM_001039160 | Gvin1 | 4.96E-01 | 4.06E+00 | 2.10E+00 | 9.31E-07 |
| NM_023061 | Mcam | 1.13E+00 | 9.12E+00 | 2.09E+00 | 1.22E-04 |
| NM_024406 | Fabp4 | 1.30E+01 | 1.05E+02 | 2.08E+00 | 3.34E-05 |
| NM_010590 | Jub | 8.07E-01 | 6.42E+00 | 2.07E+00 | 1.91E-04 |
| NM_001122899 | Lepr | 2.15E+00 | 1.71E+01 | 2.07E+00 | 1.95E-05 |
| NM_013912 | Apln | 6.46E-01 | 5.12E+00 | 2.07E+00 | 5.59E-04 |
| NM_010705 | Lgals3 | 2.45E+00 | 1.93E+01 | 2.06E+00 | 1.72E-04 |
| NM_001077192 | Abi1 | 3.57E-01 | 2.81E+00 | 2.06E+00 | 2.49E-03 |
| NM_007796 | Ctla2a | 1.33E+00 | 1.03E+01 | 2.05E+00 | 1.14E-03 |
| NM_010800 | Bhlha15 | 3.47E-01 | 2.67E+00 | 2.04E+00 | 1.51E-03 |
| NM_028657 | F630110N24Rik | 4.54E-01 | 3.50E+00 | 2.04E+00 | 1.84E-03 |
| NM_009260 | Spnb2 | 3.50E-01 | 2.69E+00 | 2.04E+00 | 1.60E-04 |
| NM_008343 | Igfbp3 | 6.97E+00 | 5.35E+01 | 2.04E+00 | 3.87E-05 |
| NM_001163473 | 1810010H24Rik | 2.97E+00 | 2.28E+01 | 2.04E+00 | 2.54E-04 |
| NM_008380 | Inhba | 2.29E+00 | 1.75E+01 | 2.03E+00 | 1.83E-04 |
| NM_011896 | Spry1 | 1.33E+00 | 1.01E+01 | 2.03E+00 | 1.87E-04 |
| NM_001048179 | Ccl27a | 2.61E-02 | 1.96E-01 | 2.02E+00 | 0.00E+00 |
| NM_138656 | Mvd | 3.43E+00 | 2.57E+01 | 2.01E+00 | 6.45E-05 |
| NM_001205330 | Mtap4 | 1.25E+00 | 9.36E+00 | 2.01E+00 | 4.04E-05 |
| NM_008013 | Fgl2 | 2.77E+00 | 2.07E+01 | 2.01E+00 | 3.66E-05 |
| NM_024442 | Cyp4f16 | 8.71E-01 | 6.49E+00 | 2.01E+00 | 1.04E-03 |
| NM_011710 | Wars | 9.10E-01 | 6.76E+00 | 2.01E+00 | 1.81E-03 |
| NM_001081155 | Rap1gap | 1.39E+00 | 1.03E+01 | 2.00E+00 | 1.10E-04 |
| NM_011847 | Dnajb6 | 1.65E+00 | 1.22E+01 | 2.00E+00 | 4.28E-04 |
| NM_177704 | Sytl5 | 7.25E-01 | 5.36E+00 | 2.00E+00 | 1.68E-03 |
| NM_018811 | Abhd2 | 1.92E+01 | 1.41E+02 | 2.00E+00 | 3.46E-03 |
| NM_011492 | Stk11 | 2.69E+00 | 1.98E+01 | 2.00E+00 | 1.38E-04 |
| NM_031167 | Il1rn | 2.81E+00 | 2.06E+01 | 1.99E+00 | 5.45E-05 |
| NM_023190 | Acin1 | 8.12E-01 | 5.94E+00 | 1.99E+00 | 1.65E-04 |
| NM_019976 | Psrc1 | 6.51E-01 | 4.72E+00 | 1.98E+00 | 4.35E-03 |
| NM_007993 | Fbn1 | 2.35E-01 | 1.69E+00 | 1.97E+00 | 6.02E-04 |
| NM_001127338 | Aldh7a1 | 3.67E-01 | 2.63E+00 | 1.97E+00 | 3.98E-03 |
| NM_010562 | Ilk | 3.35E+00 | 2.38E+01 | 1.96E+00 | 1.02E-04 |
| NM_001164724 | Il33 | 6.49E-01 | 4.58E+00 | 1.95E+00 | 2.14E-03 |
| NM_011267 | Rgs16 | 2.60E+01 | 1.81E+02 | 1.94E+00 | 4.20E-03 |
| NM_080553 | Itpr3 | 3.89E-01 | 2.70E+00 | 1.94E+00 | 2.88E-04 |
| NM_001098837 | Atxn7l3 | 8.94E-01 | 6.19E+00 | 1.94E+00 | 3.31E-04 |
| NM_173047 | Cbr3 | 4.74E+00 | 3.27E+01 | 1.93E+00 | 1.52E-04 |
| NM_172787 | L3mbtl3 | 8.30E-01 | 5.72E+00 | 1.93E+00 | 5.24E-04 |
| NM_019477 | Acsl4 | 7.41E+00 | 5.07E+01 | 1.92E+00 | 6.63E-04 |
| NM_053110 | Gpnmb | 3.17E-01 | 2.15E+00 | 1.92E+00 | 3.03E-03 |
| NR_038015 | Gm19395 | 1.30E-01 | 8.77E-01 | 1.91E+00 | 4.46E-04 |
| NM_001080749 | Gtf2i | 1.15E+00 | 7.79E+00 | 1.91E+00 | 1.71E-04 |
| NM_010345 | Grb10 | 4.42E-01 | 2.97E+00 | 1.90E+00 | 1.07E-03 |
| NM_001080940 | Gm6484 | 2.24E+01 | 1.49E+02 | 1.89E+00 | 1.04E-04 |
| NM_028732 | 4632428N05Rik | 3.69E-01 | 2.44E+00 | 1.89E+00 | 2.06E-03 |
| NM_011223 | Pxn | 2.81E+00 | 1.85E+01 | 1.89E+00 | 8.18E-05 |
| NM_028060 | Slc35f2 | 6.43E-01 | 4.24E+00 | 1.89E+00 | 3.12E-03 |
| NM_144927 | BC019943 | 1.02E-01 | 6.71E-01 | 1.88E+00 | 8.96E-06 |
| NM_010316 | Gng3 | 2.43E-02 | 1.59E-01 | 1.88E+00 | 2.94E-03 |
| NM_008721 | Npdc1 | 3.00E+00 | 1.96E+01 | 1.88E+00 | 3.35E-04 |
| NM_028304 | Pus10 | 8.87E-02 | 5.79E-01 | 1.88E+00 | 6.09E-07 |
| NM_133979 | Ano10 | 3.81E+00 | 2.48E+01 | 1.87E+00 | 9.19E-05 |
| NM_172867 | Zfp462 | 1.48E-01 | 9.58E-01 | 1.87E+00 | 3.40E-03 |
| NM_017405 | Lsr | 4.85E+00 | 3.13E+01 | 1.86E+00 | 9.24E-05 |
| NM_007734 | Col4a3 | 1.09E+00 | 6.95E+00 | 1.86E+00 | 1.07E-04 |
| NM_001162970 | Aim1l | 4.17E-01 | 2.65E+00 | 1.85E+00 | 2.14E-03 |
| NM_133900 | Psph | 1.71E+00 | 1.08E+01 | 1.84E+00 | 1.33E-03 |
| NM_001166548 | Eif4enif1 | 2.32E-02 | 1.45E-01 | 1.83E+00 | 4.23E-04 |
| NM_001139512 | Raly | 2.62E+00 | 1.64E+01 | 1.83E+00 | 5.85E-04 |
| NM_001164366 | Atp13a2 | 5.64E-01 | 3.52E+00 | 1.83E+00 | 1.46E-03 |
| NM_001111102 | Lmna | 2.90E+00 | 1.80E+01 | 1.82E+00 | 2.68E-04 |
| NM_012055 | Asns | 2.21E+00 | 1.36E+01 | 1.82E+00 | 4.98E-04 |
| NM_133983 | Cd276 | 1.28E+00 | 7.84E+00 | 1.82E+00 | 5.10E-04 |
| NM_010128 | Emp1 | 9.06E-01 | 5.57E+00 | 1.82E+00 | 1.61E-03 |
| NM_015797 | Fbxo6 | 2.12E-01 | 1.30E+00 | 1.82E+00 | 3.40E-06 |
| NM_001033380 | Itpripl2 | 1.37E+00 | 8.39E+00 | 1.81E+00 | 1.44E-04 |
| NM_021606 | Nek6 | 4.27E+00 | 2.61E+01 | 1.81E+00 | 1.23E-04 |
| NM_013749 | Tnfrsf12a | 1.63E+01 | 9.97E+01 | 1.81E+00 | 1.32E-04 |
| NM_009864 | Cdh1 | 7.59E+00 | 4.63E+01 | 1.81E+00 | 7.94E-04 |
| NM_001243009 | Col6a3 | 3.24E-01 | 1.97E+00 | 1.81E+00 | 1.04E-03 |
| NM_001177849 | Asph | 1.25E+00 | 7.62E+00 | 1.81E+00 | 1.71E-04 |
| NM_176933 | Dusp4 | 1.20E+00 | 7.26E+00 | 1.80E+00 | 1.21E-03 |
| NM_018861 | Slc1a4 | 1.12E+00 | 6.79E+00 | 1.80E+00 | 4.83E-04 |
| NM_001081328 | Chsy3 | 1.14E+00 | 6.82E+00 | 1.79E+00 | 5.05E-04 |
| NM_177078 | Adrbk2 | 6.60E-01 | 3.95E+00 | 1.79E+00 | 5.16E-04 |
| NM_008080 | B4galnt1 | 4.74E+00 | 2.84E+01 | 1.79E+00 | 1.47E-04 |
| NM_009412 | Tpd52 | 1.19E+00 | 7.14E+00 | 1.79E+00 | 1.55E-03 |
| NM_001048177 | Jak2 | 8.36E-01 | 4.99E+00 | 1.79E+00 | 5.85E-04 |
| NR_040299 | 4931408D14Rik | 4.02E+00 | 2.39E+01 | 1.78E+00 | 1.74E-04 |
| NM_146006 | Lss | 1.18E+01 | 6.96E+01 | 1.77E+00 | 1.02E-03 |
| NM_024440 | Derl3 | 1.68E+00 | 9.86E+00 | 1.77E+00 | 3.31E-03 |
| NM_019542 | Nagk | 2.09E+00 | 1.22E+01 | 1.76E+00 | 1.98E-03 |
| NM_016956 | Hbb-b2 | 4.42E+01 | 2.58E+02 | 1.76E+00 | 3.00E-04 |
| NM_172814 | Lrp12 | 5.91E-01 | 3.44E+00 | 1.76E+00 | 2.20E-03 |
| NM_001130450 | Nfe2l1 | 1.81E+00 | 1.05E+01 | 1.76E+00 | 2.37E-04 |
| NM_010741 | Ly6c1 | 2.08E+00 | 1.20E+01 | 1.75E+00 | 4.03E-03 |
| NM_177611 | Psd4 | 8.49E-01 | 4.86E+00 | 1.74E+00 | 8.50E-04 |
| NM_001163707 | Fbxo6 | 3.71E+00 | 2.12E+01 | 1.74E+00 | 8.25E-04 |
| NM_001205392 | Atg16l1 | 1.05E+00 | 5.96E+00 | 1.74E+00 | 1.35E-03 |
| NM_019454 | Dll4 | 6.94E-01 | 3.94E+00 | 1.74E+00 | 2.68E-03 |
| NM_010929 | Notch4 | 3.56E-01 | 2.01E+00 | 1.73E+00 | 2.64E-03 |
| NM_009849 | Entpd2 | 2.63E+00 | 1.49E+01 | 1.73E+00 | 6.85E-04 |
| NM_010915 | Klk1b4 | 5.57E+00 | 3.14E+01 | 1.73E+00 | 1.07E-03 |
| NM_001145821 | Ggta1 | 6.22E-01 | 3.50E+00 | 1.73E+00 | 3.51E-03 |
| NM_010283 | Ggta1 | 6.21E-01 | 3.50E+00 | 1.73E+00 | 3.29E-03 |
| NM_172710 | Sel1l3 | 2.12E+00 | 1.19E+01 | 1.73E+00 | 2.80E-04 |
| NM_198170 | Szt2 | 2.79E-01 | 1.56E+00 | 1.72E+00 | 1.51E-03 |
| NM_001243040 | Gm4070 | 5.02E-01 | 2.81E+00 | 1.72E+00 | 5.62E-05 |
| NM_177350 | Gldn | 4.28E+00 | 2.39E+01 | 1.72E+00 | 3.92E-04 |
| NM_021320 | Ntn4 | 1.11E+00 | 6.21E+00 | 1.72E+00 | 2.36E-03 |
| NM_010111 | Efnb2 | 5.54E-01 | 3.09E+00 | 1.72E+00 | 2.84E-03 |
| NM_001025264 | Tpd52 | 1.16E+00 | 6.44E+00 | 1.71E+00 | 2.43E-03 |
| NM_133654 | Cd34 | 1.10E+00 | 6.13E+00 | 1.71E+00 | 2.34E-03 |
| NM_033581 | Pcdhgc3 | 7.05E-01 | 3.90E+00 | 1.71E+00 | 1.55E-03 |
| NM_030258 | Gpr146 | 4.84E+00 | 2.67E+01 | 1.71E+00 | 4.49E-04 |
| NM_001136054 | Ate1 | 1.90E+00 | 1.04E+01 | 1.70E+00 | 3.48E-04 |
| NM_001079844 | Gpc6 | 4.06E-01 | 2.21E+00 | 1.70E+00 | 2.30E-03 |
| NM_011821 | Gpc6 | 4.07E-01 | 2.22E+00 | 1.70E+00 | 2.32E-03 |
| NM_001243201 | Gimap4 | 1.80E-01 | 9.83E-01 | 1.70E+00 | 4.64E-04 |
| NM_001081117 | Mki67 | 5.01E-01 | 2.71E+00 | 1.69E+00 | 7.79E-04 |
| NM_144878 | Fmo4 | 2.64E+00 | 1.43E+01 | 1.69E+00 | 1.06E-03 |
| NM_001033293 | Uap1l1 | 4.86E+00 | 2.62E+01 | 1.68E+00 | 4.52E-04 |
| NM_172410 | Nup93 | 1.12E+00 | 5.98E+00 | 1.68E+00 | 1.96E-03 |
| NM_007392 | Acta2 | 2.63E+00 | 1.41E+01 | 1.68E+00 | 1.08E-03 |
| NM_024169 | Fkbp11 | 1.50E+01 | 8.02E+01 | 1.68E+00 | 4.32E-04 |
| NM_145128 | Mgat5 | 1.18E+00 | 6.29E+00 | 1.67E+00 | 1.69E-03 |
| NM_001162926 | Fam84b | 1.78E+00 | 9.48E+00 | 1.67E+00 | 3.92E-04 |
| NM_001198839 | Bcar1 | 2.37E+00 | 1.26E+01 | 1.67E+00 | 5.40E-04 |
| NM_001103162 | Scap | 6.45E+00 | 3.41E+01 | 1.67E+00 | 8.96E-04 |
| NM_001081205 | Nipal1 | 1.71E+00 | 8.94E+00 | 1.65E+00 | 6.22E-04 |
| NM_001130151 | Arhgef1 | 7.45E-01 | 3.88E+00 | 1.65E+00 | 3.61E-03 |
| NM_008509 | Lpl | 5.19E+00 | 2.70E+01 | 1.65E+00 | 6.40E-04 |
| NM_001204983 | Gm9766 | 1.58E+00 | 8.20E+00 | 1.65E+00 | 1.49E-03 |
| NM_023517 | Tnfsf13 | 6.33E+00 | 3.28E+01 | 1.65E+00 | 5.01E-04 |
| NM_172514 | Tmem71 | 1.39E+00 | 7.17E+00 | 1.64E+00 | 1.72E-03 |
| NM_007742 | Col1a1 | 1.10E+00 | 5.64E+00 | 1.63E+00 | 1.16E-03 |
| NM_001003913 | Mars | 3.61E+00 | 1.84E+01 | 1.63E+00 | 5.17E-04 |
| NM_133770 | Adck4 | 7.08E+00 | 3.61E+01 | 1.63E+00 | 4.99E-04 |
| NM_027760 | Rassf8 | 3.48E+00 | 1.77E+01 | 1.63E+00 | 1.07E-03 |
| NM_001130150 | Arhgef1 | 7.59E-01 | 3.87E+00 | 1.63E+00 | 3.99E-03 |
| NM_028127 | Frmd6 | 7.25E-01 | 3.68E+00 | 1.63E+00 | 2.48E-03 |
| NM_021491 | Smpd3 | 1.08E+00 | 5.42E+00 | 1.62E+00 | 1.15E-03 |
| NM_016969 | Myadm | 2.37E+00 | 1.20E+01 | 1.62E+00 | 9.86E-04 |
| NM_001111311 | Lrrfip1 | 2.62E+00 | 1.32E+01 | 1.61E+00 | 6.47E-04 |
| NM_008686 | Nfe2l1 | 9.13E-01 | 4.56E+00 | 1.61E+00 | 1.83E-03 |
| NM_028864 | Zc3hav1 | 9.30E-01 | 4.63E+00 | 1.60E+00 | 3.26E-03 |
| NM_001164173 | Cpsf1 | 3.04E+00 | 1.51E+01 | 1.60E+00 | 5.68E-04 |
| NM_018770 | Cadm1 | 1.69E+00 | 8.41E+00 | 1.60E+00 | 8.65E-04 |
| NM_175414 | Tspan9 | 4.49E+00 | 2.23E+01 | 1.60E+00 | 6.10E-04 |
| NM_007484 | Rhoc | 1.83E+01 | 9.01E+01 | 1.60E+00 | 5.78E-04 |
| NM_177646 | Dgkd | 3.25E+00 | 1.60E+01 | 1.59E+00 | 6.76E-04 |
| NM_001205158 | Yipf2 | 2.13E+00 | 1.04E+01 | 1.59E+00 | 2.49E-03 |
| NM_001013753 | Pcdh17 | 4.09E-01 | 1.99E+00 | 1.58E+00 | 2.47E-03 |
| NM_001145830 | Plcb1 | 6.20E-01 | 3.01E+00 | 1.58E+00 | 2.09E-03 |
| NM_146173 | Tspan33 | 2.38E+00 | 1.16E+01 | 1.58E+00 | 2.16E-03 |
| NM_001109045 | Aqp8 | 1.79E+01 | 8.70E+01 | 1.58E+00 | 9.23E-04 |
| NM_001160379 | Fam46a | 1.70E+00 | 8.22E+00 | 1.58E+00 | 8.15E-04 |
| NM_172759 | Ces2e | 4.29E+00 | 2.07E+01 | 1.58E+00 | 7.71E-04 |
| NM_011716 | Wfs1 | 1.02E+00 | 4.90E+00 | 1.57E+00 | 3.24E-03 |
| NM_015731 | Atp9a | 1.03E+01 | 4.95E+01 | 1.57E+00 | 2.53E-03 |
| NM_146078 | Ubr2 | 1.69E+00 | 8.14E+00 | 1.57E+00 | 6.84E-04 |
| NM_133188 | Dazap1 | 2.64E+00 | 1.27E+01 | 1.57E+00 | 1.80E-03 |
| NM_016900 | Cav2 | 2.85E+00 | 1.36E+01 | 1.57E+00 | 1.23E-03 |
| NM_027677 | Gpr39 | 2.12E+00 | 1.02E+01 | 1.57E+00 | 1.62E-03 |
| NM_001168492 | Pdcd4 | 2.05E+00 | 9.79E+00 | 1.56E+00 | 3.54E-03 |
| NM_001001602 | Dab2ip | 7.28E-01 | 3.47E+00 | 1.56E+00 | 2.12E-03 |
| NM_001163441 | Mov10 | 1.30E+00 | 6.16E+00 | 1.56E+00 | 2.42E-03 |
| NM_183161 | Slc17a9 | 2.29E+00 | 1.08E+01 | 1.55E+00 | 2.21E-03 |
| NM_146155 | Ahdc1 | 5.94E-01 | 2.79E+00 | 1.55E+00 | 3.14E-03 |
| NM_001040111 | Arap1 | 1.30E+00 | 6.08E+00 | 1.54E+00 | 1.21E-03 |
| NM_009499 | Vasp | 4.64E+00 | 2.17E+01 | 1.54E+00 | 1.03E-03 |
| NM_001204202 | Spp1 | 1.25E+01 | 5.81E+01 | 1.54E+00 | 9.58E-04 |
| NM_001159551 | H13 | 8.85E-01 | 4.10E+00 | 1.53E+00 | 2.56E-03 |
| NM_145449 | Ifi27l2b | 2.89E+01 | 1.33E+02 | 1.53E+00 | 1.31E-03 |
| NM_153178 | Eif2c2 | 1.85E+00 | 8.50E+00 | 1.53E+00 | 9.70E-04 |
| NM_001141931 | Rbms1 | 1.88E+00 | 8.66E+00 | 1.53E+00 | 2.81E-03 |
| NM_001162939 | Aen | 2.44E+00 | 1.12E+01 | 1.53E+00 | 2.07E-03 |
| NM_001077709 | Slc39a7 | 7.16E+00 | 3.27E+01 | 1.52E+00 | 9.64E-04 |
| NM_009932 | Col4a2 | 4.09E+00 | 1.86E+01 | 1.52E+00 | 1.44E-03 |
| NM_009097 | Rps6ka1 | 3.24E+00 | 1.48E+01 | 1.52E+00 | 1.19E-03 |
| NM_016884 | Hnrnpc | 1.87E+00 | 8.50E+00 | 1.52E+00 | 2.56E-03 |
| NM_009841 | Cd14 | 4.12E+00 | 1.86E+01 | 1.51E+00 | 2.37E-03 |
| NM_001033606 | Acsl3 | 1.64E+00 | 7.43E+00 | 1.51E+00 | 2.07E-03 |
| NM_172752 | Sorbs2 | 5.45E+00 | 2.46E+01 | 1.51E+00 | 2.20E-03 |
| NM_145832 | Slc35c1 | 2.88E+00 | 1.29E+01 | 1.50E+00 | 1.70E-03 |
| NM_181409 | Mtmr11 | 2.48E+00 | 1.11E+01 | 1.50E+00 | 1.90E-03 |
| NM_015817 | Ppap2c | 8.70E+00 | 3.89E+01 | 1.50E+00 | 1.15E-03 |
| NM_008696 | Map4k4 | 1.82E+00 | 8.15E+00 | 1.50E+00 | 1.34E-03 |
| NM_028994 | Pck2 | 1.19E+00 | 5.30E+00 | 1.49E+00 | 4.28E-03 |
| NM_001077264 | Ap2a1 | 3.16E+00 | 1.41E+01 | 1.49E+00 | 1.38E-03 |
| NM_144541 | Bre | 3.67E+00 | 1.63E+01 | 1.49E+00 | 4.09E-03 |
| NM_018887 | Cyp39a1 | 6.67E+00 | 2.94E+01 | 1.48E+00 | 1.32E-03 |
| NM_001040131 | Eif4g2 | 2.33E+00 | 1.02E+01 | 1.48E+00 | 1.23E-03 |
| NM_198294 | Tanc1 | 1.34E+00 | 5.86E+00 | 1.48E+00 | 1.51E-03 |
| NM_010019 | Dapk2 | 3.64E+00 | 1.59E+01 | 1.48E+00 | 2.70E-03 |
| NM_001122739 | Inppl1 | 1.34E+00 | 5.78E+00 | 1.46E+00 | 2.85E-03 |
| NR_028440 | Ccdc85b | 5.83E-02 | 2.49E-01 | 1.45E+00 | 2.31E-03 |
| NM_133185 | Rogdi | 8.64E+00 | 3.68E+01 | 1.45E+00 | 1.76E-03 |
| NM_026473 | Tubb6 | 8.39E+00 | 3.57E+01 | 1.45E+00 | 1.58E-03 |
| NM_001142681 | Chid1 | 2.55E+00 | 1.08E+01 | 1.45E+00 | 1.78E-03 |
| NM_008135 | Slc6a9 | 6.57E+00 | 2.79E+01 | 1.44E+00 | 1.80E-03 |
| NM_133218 | Zfp704 | 9.75E-01 | 4.13E+00 | 1.44E+00 | 1.78E-03 |
| NM_020270 | Scamp5 | 5.85E+00 | 2.47E+01 | 1.44E+00 | 1.74E-03 |
| NM_010683 | Lamc1 | 1.69E+00 | 7.10E+00 | 1.43E+00 | 1.78E-03 |
| NM_007395 | Acvr1b | 1.86E+00 | 7.77E+00 | 1.43E+00 | 3.59E-03 |
| NM_001025246 | Trp53i11 | 2.32E+00 | 9.71E+00 | 1.43E+00 | 3.59E-03 |
| NM_019581 | Gtpbp2 | 2.91E+00 | 1.22E+01 | 1.43E+00 | 2.44E-03 |
| NM_011676 | Unc119 | 6.70E+00 | 2.81E+01 | 1.43E+00 | 2.74E-03 |
| NM_019422 | Elovl1 | 5.67E+00 | 2.37E+01 | 1.43E+00 | 2.26E-03 |
| NM_008305 | Hspg2 | 1.22E+00 | 5.10E+00 | 1.43E+00 | 1.76E-03 |
| NM_001033445 | Fam59a | 5.40E+00 | 2.23E+01 | 1.42E+00 | 2.75E-03 |
| NM_008608 | Mmp14 | 6.51E+00 | 2.69E+01 | 1.42E+00 | 1.90E-03 |
| NM_175357 | Crbn | 2.53E+00 | 1.04E+01 | 1.41E+00 | 2.35E-03 |
| NM_001130458 | Tcn2 | 9.88E+00 | 4.03E+01 | 1.40E+00 | 2.15E-03 |
| NM_016674 | Cldn1 | 6.88E+00 | 2.80E+01 | 1.40E+00 | 2.33E-03 |
| NM_172132 | Kdm4b | 1.55E+00 | 6.25E+00 | 1.39E+00 | 3.68E-03 |
| NM_001199948 | Dynlt1e | 6.94E-02 | 2.78E-01 | 1.39E+00 | 5.83E-05 |
| NM_001033988 | Ncoa4 | 6.52E+00 | 2.57E+01 | 1.37E+00 | 2.82E-03 |
| NM_145511 | BC003331 | 6.50E+00 | 2.56E+01 | 1.37E+00 | 2.52E-03 |
| NM_008641 | Mast2 | 1.86E+00 | 7.32E+00 | 1.37E+00 | 3.04E-03 |
| NM_001038492 | Ctsa | 4.74E+00 | 1.85E+01 | 1.36E+00 | 2.65E-03 |
| NM_001166503 | Slc39a11 | 3.16E+00 | 1.23E+01 | 1.36E+00 | 3.81E-03 |
| NM_026995 | Carkd | 6.85E+00 | 2.66E+01 | 1.36E+00 | 3.94E-03 |
| NM_019413 | Robo1 | 1.60E+00 | 6.16E+00 | 1.35E+00 | 3.36E-03 |
| NM_001081030 | Sbf1 | 1.75E+00 | 6.72E+00 | 1.34E+00 | 3.54E-03 |
| NM_001136077 | Enpp2 | 1.05E+01 | 4.01E+01 | 1.34E+00 | 4.35E-03 |
| NM_013515 | Stom | 1.09E+01 | 4.18E+01 | 1.34E+00 | 4.19E-03 |
| NM_172409 | Fmnl2 | 1.68E+00 | 6.42E+00 | 1.34E+00 | 3.90E-03 |
| NM_201640 | Cyp4a31 | 7.35E+00 | 2.79E+01 | 1.33E+00 | 3.48E-03 |
| NM_030680 | Upf1 | 3.93E+00 | 1.49E+01 | 1.33E+00 | 3.29E-03 |
| NM_013742 | Cars | 3.96E+00 | 1.50E+01 | 1.33E+00 | 3.97E-03 |
| NM_001170849 | Luc7l2 | 2.47E+00 | 9.36E+00 | 1.33E+00 | 3.64E-03 |
| NM_001098227 | Sdcbp | 1.02E+01 | 3.86E+01 | 1.33E+00 | 3.46E-03 |
| NM_025836 | Plin3 | 1.11E+01 | 4.19E+01 | 1.33E+00 | 3.45E-03 |
| NM_001042491 | Anapc5 | 3.82E+00 | 1.44E+01 | 1.32E+00 | 4.22E-03 |
| NM_201601 | Fgfr2 | 4.22E+00 | 1.57E+01 | 1.31E+00 | 3.85E-03 |
| NM_031869 | Prkab1 | 7.82E+00 | 2.90E+01 | 1.31E+00 | 3.80E-03 |
| NM_175285 | Tmem62 | 4.43E+00 | 1.63E+01 | 1.30E+00 | 4.41E-03 |
| NM_178373 | Cidec | 9.90E+00 | 3.62E+01 | 1.30E+00 | 4.16E-03 |
| NM_134156 | Actn1 | 7.04E+00 | 2.56E+01 | 1.29E+00 | 4.38E-03 |
| NM_026169 | Frmd8 | 7.57E+00 | 2.75E+01 | 1.29E+00 | 4.50E-03 |
| NM_133977 | Trf | 6.93E+03 | 2.49E+04 | 1.28E+00 | 0.00E+00 |
| NM_011479 | Sptlc2 | 5.57E+00 | 2.00E+01 | 1.28E+00 | 4.51E-03 |
| NM_001039175 | Elovl1 | 2.57E-01 | 9.10E-01 | 1.26E+00 | 3.30E-03 |
| NM_027487 | Ccdc53 | 1.81E-02 | 4.60E-02 | 9.34E-01 | 3.39E-10 |
| NR_033457 | Gm6225 | 8.95E-03 | 1.71E-02 | 6.50E-01 | 6.02E-06 |
| NM_001042557 | Map2k7 | 7.01E-03 | 1.30E-02 | 6.20E-01 | 1.14E-06 |
| NM_009654 | Alb | 5.06E+04 | 8.83E+04 | 5.56E-01 | 0.00E+00 |
| NM_009127 | Scd1 | 2.07E+03 | 2.02E+03 | -2.37E-02 | 6.35E-04 |
| NM_153168 | Lars2 | 9.55E+03 | 8.48E+03 | -1.19E-01 | 0.00E+00 |
| NM_009696 | Apoe | 1.06E+04 | 7.63E+03 | -3.31E-01 | 0.00E+00 |
| NM_026679 | D14Ertd449e | 5.47E+01 | 2.69E+01 | -7.12E-01 | 2.09E-03 |
| NR_028111 | 4930523C07Rik | 5.94E-02 | 2.71E-02 | -7.86E-01 | 1.78E-04 |
| NM_009247 | Serpina1e | 8.75E+03 | 3.82E+03 | -8.28E-01 | 0.00E+00 |
| NM_001109687 | Ehmt1 | 8.83E-03 | 3.66E-03 | -8.81E-01 | 5.67E-09 |
| NM_001164499 | Papd5 | 1.48E-02 | 5.98E-03 | -9.06E-01 | 0.00E+00 |
| NM_001110855 | Crem | 9.88E-02 | 3.64E-02 | -9.98E-01 | 3.50E-12 |
| NM_001159751 | Tcea1 | 4.30E-02 | 1.46E-02 | -1.08E+00 | 0.00E+00 |
| NM_025790 | Acot13 | 1.31E+02 | 4.32E+01 | -1.11E+00 | 4.47E-03 |
| NM_001033225 | Pnrc1 | 4.46E+01 | 1.47E+01 | -1.11E+00 | 4.25E-03 |
| NM_054080 | Akr1c20 | 8.45E+01 | 2.74E+01 | -1.13E+00 | 4.05E-03 |
| NM_001081408 | Agmat | 7.44E+01 | 2.40E+01 | -1.13E+00 | 3.77E-03 |
| NR_001460 | Rmrp | 8.86E+02 | 2.86E+02 | -1.13E+00 | 3.64E-03 |
| NM_001104525 | Cyp2c69 | 6.44E+01 | 2.07E+01 | -1.13E+00 | 3.96E-03 |
| NM_145443 | L2hgdh | 1.93E+01 | 6.19E+00 | -1.14E+00 | 3.54E-03 |
| NM_001164745 | Ptp4a2 | 2.82E+01 | 9.02E+00 | -1.14E+00 | 3.55E-03 |
| NM_007996 | Fdx1 | 1.15E+02 | 3.67E+01 | -1.14E+00 | 3.33E-03 |
| NM_011344 | Sel1l | 1.32E+01 | 4.19E+00 | -1.15E+00 | 3.28E-03 |
| NM_007757 | Cpox | 3.94E+01 | 1.25E+01 | -1.15E+00 | 3.76E-03 |
| NM_030114 | Herc4 | 9.92E+00 | 3.11E+00 | -1.16E+00 | 3.78E-03 |
| NM_001243063 | Nr1i3 | 3.63E+01 | 1.14E+01 | -1.16E+00 | 3.35E-03 |
| NM_025387 | Tmem14c | 1.53E+02 | 4.78E+01 | -1.17E+00 | 3.32E-03 |
| NM_026701 | Pbld1 | 7.86E+01 | 2.44E+01 | -1.17E+00 | 3.57E-03 |
| NM_027552 | Kynu | 5.74E+01 | 1.78E+01 | -1.17E+00 | 3.74E-03 |
| NM_028388 | Ndufv2 | 1.15E+02 | 3.56E+01 | -1.18E+00 | 3.71E-03 |
| NM_144836 | Slc17a2 | 5.27E+01 | 1.61E+01 | -1.18E+00 | 3.49E-03 |
| NM_010591 | Jun | 2.23E+01 | 6.83E+00 | -1.18E+00 | 2.35E-03 |
| NM_001110208 | Akt2 | 1.18E+01 | 3.61E+00 | -1.19E+00 | 3.28E-03 |
| NM_013863 | Bag3 | 4.22E+01 | 1.29E+01 | -1.19E+00 | 2.49E-03 |
| NM_001037726 | Creb1 | 9.24E-03 | 2.82E-03 | -1.19E+00 | 0.00E+00 |
| NM_029556 | Clybl | 3.99E+01 | 1.21E+01 | -1.19E+00 | 2.74E-03 |
| NM_027570 | Ldhd | 5.30E+01 | 1.60E+01 | -1.19E+00 | 2.33E-03 |
| NM_172961 | Abat | 3.77E+01 | 1.14E+01 | -1.19E+00 | 3.64E-03 |
| NM_030717 | Lactb | 2.48E+01 | 7.49E+00 | -1.20E+00 | 2.51E-03 |
| NM_013642 | Dusp1 | 2.67E+01 | 8.06E+00 | -1.20E+00 | 2.48E-03 |
| NM_001039678 | Prhoxnb | 4.53E+01 | 1.35E+01 | -1.21E+00 | 4.18E-03 |
| NM_027696 | Mier1 | 8.29E+00 | 2.44E+00 | -1.22E+00 | 2.37E-03 |
| NM_025897 | Rrp8 | 1.53E+01 | 4.52E+00 | -1.22E+00 | 2.15E-03 |
| NM_008303 | Hspe1 | 2.92E+02 | 8.59E+01 | -1.22E+00 | 2.83E-03 |
| NM_009040 | Rdh16 | 2.16E+01 | 6.33E+00 | -1.23E+00 | 1.63E-03 |
| NM_013546 | Hebp1 | 2.16E+02 | 6.32E+01 | -1.23E+00 | 3.13E-03 |
| NM_001160038 | Ndufs1 | 1.33E+01 | 3.88E+00 | -1.23E+00 | 2.27E-03 |
| NM_008993 | Pxmp2 | 1.51E+02 | 4.37E+01 | -1.24E+00 | 1.76E-03 |
| NM_025348 | Ndufa3 | 4.71E+02 | 1.36E+02 | -1.24E+00 | 1.39E-03 |
| NM_001081394 | 0610007L01Rik | 1.17E+01 | 3.35E+00 | -1.25E+00 | 1.84E-03 |
| NM_021704 | Cxcl12 | 5.50E+01 | 1.58E+01 | -1.25E+00 | 1.36E-03 |
| NM_028785 | Dock8 | 5.65E+00 | 1.62E+00 | -1.25E+00 | 1.67E-03 |
| NM_023422 | Hist1h2bc | 2.72E+02 | 7.79E+01 | -1.25E+00 | 1.86E-03 |
| NM_008853 | Pja1 | 1.57E+01 | 4.50E+00 | -1.25E+00 | 2.42E-03 |
| NM_010286 | Tsc22d3 | 5.85E+01 | 1.67E+01 | -1.26E+00 | 1.32E-03 |
| NM_025926 | Dnajb4 | 4.39E+01 | 1.25E+01 | -1.26E+00 | 1.29E-03 |
| NM_024446 | Nudt7 | 8.67E+01 | 2.46E+01 | -1.26E+00 | 1.20E-03 |
| NM_009139 | Ccl6 | 1.64E+01 | 4.63E+00 | -1.26E+00 | 4.39E-03 |
| NM_026003 | Smarca2 | 1.39E+01 | 3.94E+00 | -1.26E+00 | 3.18E-03 |
| NM_025690 | Sltm | 6.88E+00 | 1.94E+00 | -1.26E+00 | 3.28E-03 |
| NM_011703 | Vipr1 | 4.85E+00 | 1.37E+00 | -1.27E+00 | 3.53E-03 |
| NM_009653 | Alas2 | 4.06E+01 | 1.14E+01 | -1.27E+00 | 1.10E-03 |
| NM_013671 | Sod2 | 6.61E+01 | 1.86E+01 | -1.27E+00 | 3.38E-03 |
| NM_001162883 | Apol9a | 4.52E+01 | 1.27E+01 | -1.27E+00 | 1.19E-03 |
| NM_026931 | 1810011O10Rik | 5.83E+01 | 1.63E+01 | -1.27E+00 | 1.08E-03 |
| NM_174846 | Glyctk | 2.79E+01 | 7.80E+00 | -1.27E+00 | 1.16E-03 |
| NM_001042634 | Clk1 | 2.27E+01 | 6.36E+00 | -1.27E+00 | 1.57E-03 |
| NM_172267 | Phyhd1 | 3.87E+01 | 1.08E+01 | -1.27E+00 | 1.34E-03 |
| NM_025374 | Glo1 | 1.47E+02 | 4.10E+01 | -1.28E+00 | 3.39E-03 |
| NM_001044744 | Gcdh | 1.26E+02 | 3.50E+01 | -1.28E+00 | 3.16E-03 |
| NM_153787 | Bclaf1 | 7.52E+00 | 2.10E+00 | -1.28E+00 | 1.43E-03 |
| NM_001033305 | Ndufb6 | 1.20E+02 | 3.33E+01 | -1.28E+00 | 1.40E-03 |
| NM_001161744 | Hsd3b3 | 3.34E+01 | 9.28E+00 | -1.28E+00 | 1.10E-03 |
| NM_001168295 | Serpina3f | 9.85E+00 | 2.72E+00 | -1.29E+00 | 4.38E-03 |
| NM_001159717 | 2-Sep | 1.24E+01 | 3.41E+00 | -1.29E+00 | 1.36E-03 |
| NM_026614 | Ndufa5 | 1.28E+02 | 3.52E+01 | -1.29E+00 | 1.11E-03 |
| NR_036450 | Gm14403 | 1.41E+01 | 3.85E+00 | -1.30E+00 | 9.93E-04 |
| NM_001100180 | Cyp3a57 | 1.26E+01 | 3.44E+00 | -1.30E+00 | 4.38E-03 |
| NM_008952 | Pipox | 1.88E+02 | 5.13E+01 | -1.30E+00 | 3.65E-03 |
| NM_011016 | Orm2 | 3.34E+02 | 9.10E+01 | -1.30E+00 | 1.71E-03 |
| NM_144516 | Zmynd11 | 4.75E+00 | 1.29E+00 | -1.30E+00 | 4.20E-03 |
| NM_008188 | Thumpd3 | 1.09E+01 | 2.95E+00 | -1.30E+00 | 3.31E-03 |
| NM_022026 | Aqp9 | 1.32E+02 | 3.57E+01 | -1.31E+00 | 4.31E-03 |
| NM_001161745 | Hsd3b3 | 3.28E+01 | 8.84E+00 | -1.31E+00 | 9.06E-04 |
| NM_010886 | Ndufa4 | 1.02E+03 | 2.74E+02 | -1.31E+00 | 3.23E-03 |
| NM_001100181 | Cyp4a32 | 2.38E+01 | 6.42E+00 | -1.31E+00 | 1.03E-03 |
| NM_001114096 | Smarcc2 | 4.58E+00 | 1.23E+00 | -1.31E+00 | 3.04E-03 |
| NM_001161742 | Hsd3b3 | 3.25E+01 | 8.74E+00 | -1.31E+00 | 8.37E-04 |
| NM_001204333 | Cyp4f14 | 2.14E+01 | 5.75E+00 | -1.31E+00 | 1.04E-03 |
| NM_008458 | Serpina3c | 5.21E+01 | 1.39E+01 | -1.32E+00 | 6.63E-04 |
| NM_007608 | Car5a | 4.27E+01 | 1.13E+01 | -1.33E+00 | 8.61E-04 |
| NM_010775 | Mbl1 | 1.65E+02 | 4.37E+01 | -1.33E+00 | 7.12E-04 |
| NM_033601 | Bcl3 | 2.40E+01 | 6.34E+00 | -1.33E+00 | 9.12E-04 |
| NM_194321 | Fxyd1 | 4.62E+01 | 1.22E+01 | -1.33E+00 | 3.24E-03 |
| NR_027853 | Clk1 | 1.39E+01 | 3.67E+00 | -1.33E+00 | 2.55E-03 |
| NR_028042 | Gm10012 | 1.75E+02 | 4.61E+01 | -1.33E+00 | 1.40E-03 |
| NM_153807 | Acsf2 | 3.62E+01 | 9.55E+00 | -1.33E+00 | 6.24E-04 |
| NM_019878 | Sult1b1 | 1.46E+01 | 3.84E+00 | -1.33E+00 | 1.03E-03 |
| NM_175316 | Slco2b1 | 2.66E+01 | 6.94E+00 | -1.34E+00 | 6.02E-04 |
| NM_020030 | Raet1d | 3.08E+01 | 8.03E+00 | -1.34E+00 | 1.10E-03 |
| NM_013559 | Hsph1 | 7.84E+01 | 2.04E+01 | -1.35E+00 | 1.95E-03 |
| NR_028420 | Zfp809 | 9.98E+00 | 2.59E+00 | -1.35E+00 | 3.85E-03 |
| NM_013930 | Aass | 1.03E+02 | 2.64E+01 | -1.36E+00 | 3.39E-03 |
| NM_001038703 | Gpr146 | 8.37E+00 | 2.15E+00 | -1.36E+00 | 9.62E-04 |
| NM_029550 | Keg1 | 1.02E+02 | 2.61E+01 | -1.36E+00 | 6.67E-04 |
| NM_001161743 | Hsd3b3 | 3.31E+01 | 8.49E+00 | -1.36E+00 | 5.45E-04 |
| NM_053122 | Immp2l | 2.50E+01 | 6.42E+00 | -1.36E+00 | 1.72E-03 |
| NM_007899 | Ecm1 | 4.08E+01 | 1.04E+01 | -1.36E+00 | 4.64E-04 |
| NM_175329 | Chchd10 | 4.12E+02 | 1.05E+02 | -1.36E+00 | 2.40E-03 |
| NM_026185 | Abhd15 | 9.11E+00 | 2.33E+00 | -1.36E+00 | 1.22E-03 |
| NM_010231 | Fmo1 | 1.54E+02 | 3.91E+01 | -1.37E+00 | 2.63E-03 |
| NM_025626 | Fam107b | 3.31E+01 | 8.41E+00 | -1.37E+00 | 4.40E-04 |
| NM_053071 | Cox6c | 5.43E+02 | 1.38E+02 | -1.37E+00 | 7.10E-04 |
| NM_172771 | Dmxl2 | 4.42E+00 | 1.12E+00 | -1.37E+00 | 5.50E-04 |
| NM_053109 | Clec2d | 1.32E+02 | 3.33E+01 | -1.37E+00 | 5.09E-04 |
| NM_173786 | Apol9a | 2.23E+01 | 5.65E+00 | -1.37E+00 | 1.03E-03 |
| NM_177789 | Vsig4 | 3.78E+01 | 9.55E+00 | -1.37E+00 | 5.42E-04 |
| NM_001039201 | Hdhd2 | 8.99E+00 | 2.27E+00 | -1.38E+00 | 2.19E-03 |
| NM_001159631 | Nek6 | 9.56E+00 | 2.41E+00 | -1.38E+00 | 1.05E-03 |
| NM_026085 | Pbld2 | 5.21E+01 | 1.31E+01 | -1.38E+00 | 3.85E-04 |
| NM_027827 | Afmid | 4.94E+01 | 1.23E+01 | -1.39E+00 | 3.75E-04 |
| NM_001160236 | Fam168b | 3.97E+00 | 9.90E-01 | -1.39E+00 | 2.05E-03 |
| NM_175369 | Ccdc122 | 1.25E+01 | 3.11E+00 | -1.39E+00 | 2.08E-03 |
| NM_001166556 | Abca6 | 2.01E+01 | 4.99E+00 | -1.39E+00 | 1.11E-03 |
| NM_028626 | Mcee | 1.08E+02 | 2.68E+01 | -1.39E+00 | 3.84E-04 |
| NM_008748 | Dusp8 | 2.64E+00 | 6.53E-01 | -1.39E+00 | 4.45E-03 |
| NM_001039646 | Gbp10 | 6.38E+00 | 1.58E+00 | -1.40E+00 | 1.64E-03 |
| NM_001190732 | Mtrnr2l | 3.38E+03 | 8.36E+02 | -1.40E+00 | 4.30E-03 |
| NM_028056 | 1600002H07Rik | 3.86E+01 | 9.54E+00 | -1.40E+00 | 3.18E-04 |
| NM_019580 | Gde1 | 4.45E+01 | 1.09E+01 | -1.40E+00 | 3.26E-04 |
| NM_028720 | Glyr1 | 1.56E+01 | 3.83E+00 | -1.40E+00 | 3.81E-04 |
| NM_001164620 | Ccdc157 | 2.56E+00 | 6.29E-01 | -1.40E+00 | 4.12E-03 |
| NM_198193 | Raet1e | 2.61E+01 | 6.40E+00 | -1.41E+00 | 8.82E-04 |
| NM_001177354 | Myc | 9.32E+00 | 2.28E+00 | -1.41E+00 | 1.59E-03 |
| NM_001037863 | Atp11c | 4.43E+01 | 1.08E+01 | -1.41E+00 | 1.00E-03 |
| NM_145567 | Hibadh | 1.81E+02 | 4.42E+01 | -1.41E+00 | 1.31E-03 |
| NM_027290 | Mcm10 | 1.68E+01 | 4.09E+00 | -1.41E+00 | 3.20E-04 |
| NM_028035 | Snx10 | 1.48E+01 | 3.61E+00 | -1.41E+00 | 5.55E-04 |
| NM_175277 | Bola3 | 1.00E+02 | 2.44E+01 | -1.41E+00 | 6.29E-04 |
| NM_172763 | Zfp809 | 1.37E+01 | 3.35E+00 | -1.41E+00 | 2.12E-03 |
| NM_013590 | Lyz1 | 2.36E+01 | 5.76E+00 | -1.41E+00 | 1.05E-03 |
| NM_007700 | Chuk | 2.03E+01 | 4.94E+00 | -1.41E+00 | 2.77E-04 |
| NM_010702 | Lect2 | 2.08E+02 | 5.02E+01 | -1.42E+00 | 3.54E-04 |
| NM_134103 | Il1rap | 6.51E+01 | 1.57E+01 | -1.42E+00 | 3.54E-04 |
| NM_023440 | Tmem86b | 3.55E+01 | 8.56E+00 | -1.42E+00 | 4.79E-04 |
| NM_013464 | Ahr | 1.78E+01 | 4.30E+00 | -1.42E+00 | 2.52E-04 |
| NR_027396 | Rnf44 | 4.52E+00 | 1.09E+00 | -1.42E+00 | 2.28E-03 |
| NM_025404 | Arl4d | 2.10E+01 | 5.05E+00 | -1.42E+00 | 9.77E-04 |
| NM_008558 | Max | 1.42E+01 | 3.42E+00 | -1.43E+00 | 9.09E-04 |
| NM_009786 | Cacybp | 4.83E+01 | 1.16E+01 | -1.43E+00 | 2.32E-04 |
| NR_027477 | Chpt1 | 3.72E+01 | 8.91E+00 | -1.43E+00 | 2.84E-04 |
| NM_001081131 | Dhtkd1 | 7.99E+00 | 1.91E+00 | -1.43E+00 | 8.22E-04 |
| NM_008690 | Nfkbie | 6.00E+00 | 1.43E+00 | -1.43E+00 | 3.13E-03 |
| NR_027395 | Rnf44 | 4.61E+00 | 1.10E+00 | -1.43E+00 | 2.01E-03 |
| NM_173422 | Colec10 | 8.59E+00 | 2.04E+00 | -1.43E+00 | 4.20E-04 |
| NR_002445 | BC002163 | 1.55E+02 | 3.68E+01 | -1.44E+00 | 7.91E-05 |
| NM_134021 | Pnpo | 6.81E+01 | 1.61E+01 | -1.44E+00 | 2.48E-04 |
| NM_001038641 | 2210009G21Rik | 6.48E+00 | 1.54E+00 | -1.44E+00 | 1.84E-03 |
| NM_027348 | Pmvk | 1.22E+01 | 2.90E+00 | -1.44E+00 | 3.64E-03 |
| NM_001199350 | Rpl34-ps1 | 5.68E+01 | 1.34E+01 | -1.44E+00 | 3.57E-03 |
| NM_016771 | Sult1d1 | 1.18E+02 | 2.79E+01 | -1.44E+00 | 7.45E-04 |
| NM_181821 | Hcfc1r1 | 2.91E+01 | 6.86E+00 | -1.44E+00 | 5.81E-04 |
| NM_010907 | Nfkbia | 5.58E+01 | 1.32E+01 | -1.45E+00 | 1.95E-04 |
| NM_025551 | Ndufa12 | 9.36E+01 | 2.20E+01 | -1.45E+00 | 4.42E-04 |
| NM_001163729 | Klhdc3 | 6.32E+00 | 1.49E+00 | -1.45E+00 | 3.73E-03 |
| NM_153193 | Hsd3b2 | 3.60E+01 | 8.45E+00 | -1.45E+00 | 2.22E-04 |
| NM_024427 | Tpm1 | 9.45E+00 | 2.22E+00 | -1.45E+00 | 2.44E-03 |
| NM_013821 | Hsd3b6 | 8.94E+00 | 2.10E+00 | -1.45E+00 | 2.40E-03 |
| NM_025826 | Acadsb | 5.38E+01 | 1.26E+01 | -1.45E+00 | 3.13E-04 |
| NM_016847 | Avpr1a | 5.24E+00 | 1.22E+00 | -1.46E+00 | 2.86E-03 |
| NM_001190357 | Carkd | 2.14E+01 | 5.00E+00 | -1.46E+00 | 8.60E-04 |
| NM_007717 | Cmah | 2.30E+00 | 5.37E-01 | -1.46E+00 | 1.09E-03 |
| NM_001160219 | Pum2 | 6.47E+00 | 1.50E+00 | -1.46E+00 | 2.97E-04 |
| NM_001145826 | Specc1l | 2.13E+00 | 4.94E-01 | -1.46E+00 | 2.75E-03 |
| NM_015760 | Nox4 | 1.27E+01 | 2.94E+00 | -1.46E+00 | 2.83E-04 |
| NM_015829 | Slc25a13 | 4.13E+01 | 9.57E+00 | -1.46E+00 | 1.94E-04 |
| NM_019503 | Fxyd1 | 2.39E+02 | 5.50E+01 | -1.47E+00 | 1.55E-04 |
| NM_023160 | Cml1 | 1.42E+02 | 3.26E+01 | -1.47E+00 | 1.75E-04 |
| NM_007652 | Cd59a | 4.65E+01 | 1.07E+01 | -1.47E+00 | 1.58E-04 |
| NM_001033291 | Usp40 | 2.99E+00 | 6.87E-01 | -1.47E+00 | 2.02E-03 |
| NR_002142 | Rpph1 | 4.38E+02 | 1.01E+02 | -1.47E+00 | 1.88E-04 |
| NM_019792 | Cyp3a25 | 2.22E+02 | 5.08E+01 | -1.47E+00 | 1.73E-03 |
| NM_011518 | Sykb | 2.19E+00 | 5.02E-01 | -1.48E+00 | 3.49E-03 |
| NM_133894 | Ugt2b38 | 1.44E+02 | 3.30E+01 | -1.48E+00 | 4.77E-04 |
| NM_027186 | Rpain | 1.36E+01 | 3.10E+00 | -1.48E+00 | 2.89E-03 |
| NM_199314 | Serpina11 | 1.27E+01 | 2.88E+00 | -1.48E+00 | 1.58E-03 |
| NM_145956 | Brcc3 | 3.28E+00 | 7.43E-01 | -1.48E+00 | 2.28E-03 |
| NM_010512 | Igf1 | 4.31E+00 | 9.77E-01 | -1.48E+00 | 4.27E-04 |
| NM_001042408 | Txnl4a | 2.00E+01 | 4.52E+00 | -1.49E+00 | 4.38E-04 |
| NM_001039386 | Nelf | 1.11E+01 | 2.49E+00 | -1.50E+00 | 3.73E-04 |
| NM_170778 | Dpyd | 1.30E+02 | 2.91E+01 | -1.50E+00 | 3.64E-03 |
| NM_001001798 | Atp11c | 1.08E+01 | 2.40E+00 | -1.50E+00 | 1.22E-04 |
| NM_008668 | Nab2 | 5.76E+00 | 1.29E+00 | -1.50E+00 | 1.95E-03 |
| NM_029331 | 1700019G17Rik | 3.21E+01 | 7.15E+00 | -1.50E+00 | 1.09E-04 |
| NM_008819 | Pemt | 3.32E+02 | 7.33E+01 | -1.51E+00 | 2.74E-04 |
| NM_001098404 | Nr1i2 | 7.36E+00 | 1.62E+00 | -1.51E+00 | 1.35E-03 |
| NM_146015 | Efemp1 | 7.26E+00 | 1.60E+00 | -1.51E+00 | 1.98E-03 |
| NM_146157 | C230096C10Rik | 2.62E+00 | 5.77E-01 | -1.51E+00 | 1.43E-03 |
| NM_009729 | Atp6v0c | 9.69E+01 | 2.13E+01 | -1.51E+00 | 9.58E-05 |
| NR_027485 | Psmd2 | 4.07E+00 | 8.96E-01 | -1.51E+00 | 2.39E-03 |
| NM_001177558 | Gng12 | 3.25E+00 | 7.16E-01 | -1.51E+00 | 2.06E-03 |
| NM_008298 | Dnaja1 | 8.82E+01 | 1.94E+01 | -1.51E+00 | 2.92E-03 |
| NM_029821 | 1190003J15Rik | 4.02E+02 | 8.83E+01 | -1.51E+00 | 2.08E-04 |
| NM_016751 | Clec4f | 3.39E+01 | 7.46E+00 | -1.51E+00 | 9.39E-05 |
| NM_001177568 | Gm14420 | 1.18E+01 | 2.60E+00 | -1.52E+00 | 1.20E-04 |
| NM_028094 | Ugt2a3 | 2.32E+02 | 5.08E+01 | -1.52E+00 | 1.52E-03 |
| NM_001013785 | Akr1c19 | 8.97E+01 | 1.96E+01 | -1.52E+00 | 8.77E-05 |
| NM_007519 | Baat | 1.44E+02 | 3.16E+01 | -1.52E+00 | 3.42E-04 |
| NM_010888 | Ndufs6 | 1.40E+02 | 3.05E+01 | -1.53E+00 | 1.49E-04 |
| NM_001128606 | Epb4.1 | 3.54E+00 | 7.67E-01 | -1.53E+00 | 1.00E-03 |
| NM_198122 | Arid4b | 2.27E+00 | 4.91E-01 | -1.53E+00 | 1.76E-03 |
| NM_001243041 | Adk | 3.68E+02 | 7.93E+01 | -1.53E+00 | 3.53E-03 |
| NM_007471 | App | 4.86E-02 | 1.05E-02 | -1.53E+00 | 0.00E+00 |
| NM_133768 | Asl | 3.78E+02 | 8.14E+01 | -1.54E+00 | 2.53E-03 |
| NM_001159525 | Pex19 | 3.50E+00 | 7.52E-01 | -1.54E+00 | 3.29E-03 |
| NM_010889 | Neb | 1.78E+00 | 3.84E-01 | -1.54E+00 | 1.52E-04 |
| NM_028744 | Pi4k2b | 2.76E+00 | 5.94E-01 | -1.54E+00 | 4.05E-03 |
| NM_172612 | Rnd1 | 5.32E+00 | 1.14E+00 | -1.54E+00 | 2.56E-03 |
| NM_144943 | Cd207 | 1.12E+01 | 2.40E+00 | -1.54E+00 | 1.28E-03 |
| NM_177005 | Glt1d1 | 1.49E+01 | 3.19E+00 | -1.54E+00 | 6.87E-04 |
| NM_001044740 | Slc7a2 | 7.01E+00 | 1.50E+00 | -1.54E+00 | 9.65E-05 |
| NM_009397 | Tnfaip3 | 6.21E+00 | 1.32E+00 | -1.55E+00 | 3.63E-04 |
| NM_001160303 | Gm4788 | 8.63E+01 | 1.84E+01 | -1.55E+00 | 1.97E-04 |
| NM_145502 | Erlin1 | 1.15E+01 | 2.43E+00 | -1.55E+00 | 1.66E-04 |
| NM_001177561 | Slc10a1 | 3.94E+02 | 8.36E+01 | -1.55E+00 | 2.67E-03 |
| NM_001076676 | Usp33 | 4.26E+00 | 9.04E-01 | -1.55E+00 | 9.67E-04 |
| NM_001114140 | Tcf20 | 4.51E+00 | 9.56E-01 | -1.55E+00 | 2.02E-04 |
| NM_139292 | Reep6 | 1.50E+01 | 3.16E+00 | -1.55E+00 | 3.04E-04 |
| NM_001141948 | Nmi | 6.64E+00 | 1.40E+00 | -1.55E+00 | 4.43E-03 |
| NM_001159317 | Il1rap | 2.62E+00 | 5.52E-01 | -1.56E+00 | 2.53E-03 |
| NM_001190451 | Dcn | 1.44E+02 | 3.04E+01 | -1.56E+00 | 2.21E-04 |
| NM_001190444 | Aamp | 1.87E+01 | 3.94E+00 | -1.56E+00 | 2.40E-04 |
| NM_001145877 | Slc25a44 | 6.27E+00 | 1.32E+00 | -1.56E+00 | 5.61E-04 |
| NM_152816 | Dnm1l | 7.23E+00 | 1.52E+00 | -1.56E+00 | 2.66E-04 |
| NM_007807 | Cybb | 3.70E+01 | 7.76E+00 | -1.56E+00 | 9.98E-05 |
| NM_016973 | St6galnac6 | 5.79E+00 | 1.21E+00 | -1.57E+00 | 1.40E-03 |
| NM_175236 | Adhfe1 | 2.10E+01 | 4.39E+00 | -1.57E+00 | 6.19E-05 |
| NM_001122766 | Sirt2 | 1.93E+01 | 4.04E+00 | -1.57E+00 | 2.57E-04 |
| NM_053215 | Ugt2b37 | 6.89E+01 | 1.44E+01 | -1.57E+00 | 5.79E-05 |
| NM_008524 | Lum | 7.11E+00 | 1.48E+00 | -1.57E+00 | 1.39E-03 |
| NR_037974 | Jagn1 | 1.02E+01 | 2.13E+00 | -1.57E+00 | 2.22E-03 |
| NM_175417 | 9530008L14Rik | 5.71E+01 | 1.19E+01 | -1.57E+00 | 5.81E-05 |
| NM_001083955 | Hba-a2 | 1.37E+02 | 2.85E+01 | -1.57E+00 | 3.88E-07 |
| NM_010284 | Ghr | 1.23E+02 | 2.56E+01 | -1.57E+00 | 1.98E-03 |
| NM_001145164 | Tgtp2 | 2.30E+01 | 4.76E+00 | -1.57E+00 | 2.19E-07 |
| NM_001037741 | Gpx4 | 1.08E+01 | 2.21E+00 | -1.58E+00 | 3.02E-03 |
| NM_011458 | Serpina3k | 8.02E+03 | 1.65E+03 | -1.58E+00 | 0.00E+00 |
| NM_001081050 | Pard3b | 1.07E+00 | 2.19E-01 | -1.58E+00 | 2.77E-03 |
| NM_001166031 | Mrps2 | 8.00E+00 | 1.64E+00 | -1.59E+00 | 9.76E-04 |
| NM_010324 | Got1 | 1.32E+02 | 2.69E+01 | -1.59E+00 | 1.44E-04 |
| NM_001039512 | Ivns1abp | 4.46E+00 | 9.11E-01 | -1.59E+00 | 1.07E-03 |
| NM_008176 | Cxcl1 | 1.03E+02 | 2.09E+01 | -1.59E+00 | 4.16E-05 |
| NM_001172096 | 2700078E11Rik | 2.60E+00 | 5.28E-01 | -1.59E+00 | 1.04E-03 |
| NM_027491 | Rragd | 1.40E+01 | 2.86E+00 | -1.59E+00 | 4.41E-05 |
| NM_199195 | Bckdhb | 7.89E+01 | 1.60E+01 | -1.59E+00 | 4.15E-05 |
| NM_026219 | Uqcrb | 3.69E+02 | 7.49E+01 | -1.59E+00 | 4.11E-05 |
| NM_008094 | Gba | 3.88E+00 | 7.87E-01 | -1.59E+00 | 4.25E-03 |
| NR_040298 | 4931408D14Rik | 3.99E+00 | 8.10E-01 | -1.60E+00 | 1.36E-03 |
| NM_001081079 | Ogfrl1 | 4.42E+00 | 8.96E-01 | -1.60E+00 | 4.28E-04 |
| NM_031159 | Apobec1 | 9.92E+00 | 2.00E+00 | -1.60E+00 | 4.24E-04 |
| NM_001159417 | Irf9 | 6.52E+00 | 1.31E+00 | -1.60E+00 | 8.02E-04 |
| NM_001164359 | Erlin1 | 1.15E+01 | 2.30E+00 | -1.61E+00 | 1.11E-04 |
| NM_001163457 | Mttp | 3.78E+00 | 7.57E-01 | -1.61E+00 | 7.65E-04 |
| NM_001009949 | Mcart1 | 2.31E+01 | 4.62E+00 | -1.61E+00 | 3.34E-05 |
| NM_001193660 | Cldn12 | 1.08E+01 | 2.16E+00 | -1.61E+00 | 8.57E-05 |
| NM_133853 | Magi3 | 3.94E+00 | 7.85E-01 | -1.61E+00 | 2.30E-04 |
| NM_010720 | Lipg | 4.56E+00 | 9.10E-01 | -1.61E+00 | 6.66E-04 |
| NM_001080386 | Clta | 2.85E+01 | 5.67E+00 | -1.61E+00 | 2.39E-04 |
| NM_001039354 | Lin7a | 3.58E+00 | 7.14E-01 | -1.61E+00 | 3.91E-04 |
| NM_001099674 | 1810022K09Rik | 1.49E+02 | 2.97E+01 | -1.61E+00 | 1.52E-04 |
| NM_017373 | Nfil3 | 1.79E+01 | 3.55E+00 | -1.61E+00 | 1.25E-04 |
| NM_001163160 | Pcyt1a | 3.32E+00 | 6.59E-01 | -1.62E+00 | 7.07E-04 |
| NM_025806 | Plbd1 | 2.56E+01 | 5.06E+00 | -1.62E+00 | 5.79E-05 |
| NM_173788 | Npr2 | 1.01E+01 | 2.01E+00 | -1.62E+00 | 9.40E-05 |
| NM_153069 | Leap2 | 9.98E+01 | 1.97E+01 | -1.62E+00 | 1.51E-04 |
| NM_001177804 | Sirt3 | 2.00E+01 | 3.94E+00 | -1.62E+00 | 2.46E-04 |
| NM_001076554 | Spna2 | 7.45E+00 | 1.47E+00 | -1.62E+00 | 3.67E-05 |
| NM_016704 | C6 | 8.37E+01 | 1.65E+01 | -1.62E+00 | 8.71E-05 |
| NM_001168539 | Tsku | 1.16E+01 | 2.28E+00 | -1.63E+00 | 1.81E-04 |
| NM_052991 | Fxyd1 | 6.23E+01 | 1.23E+01 | -1.63E+00 | 3.33E-04 |
| NM_011579 | Tgtp1 | 9.33E+00 | 1.82E+00 | -1.63E+00 | 2.33E-04 |
| NM_008035 | Folr2 | 1.29E+01 | 2.52E+00 | -1.63E+00 | 9.82E-04 |
| NR_015583 | BC024386 | 8.69E+01 | 1.70E+01 | -1.63E+00 | 2.80E-05 |
| NM_001193659 | Cldn12 | 1.17E+01 | 2.28E+00 | -1.63E+00 | 5.84E-05 |
| NM_198645 | Ccdc58 | 9.41E+00 | 1.83E+00 | -1.64E+00 | 2.28E-03 |
| NM_013655 | Cxcl12 | 3.97E+01 | 7.72E+00 | -1.64E+00 | 2.60E-05 |
| NM_001164528 | Ildr2 | 3.54E+00 | 6.88E-01 | -1.64E+00 | 1.42E-04 |
| NM_009929 | Col18a1 | 4.91E+00 | 9.53E-01 | -1.64E+00 | 2.26E-04 |
| NR_003634 | Rps4y2 | 1.13E+01 | 2.18E+00 | -1.65E+00 | 2.27E-03 |
| NM_001164640 | Apol7a | 1.65E+01 | 3.17E+00 | -1.65E+00 | 7.70E-05 |
| NM_029628 | Ppp1r3g | 3.43E+00 | 6.58E-01 | -1.65E+00 | 2.85E-03 |
| NM_001025608 | D4Ertd22e | 1.49E+01 | 2.86E+00 | -1.65E+00 | 4.02E-05 |
| NM_145559 | Slc2a9 | 2.99E+00 | 5.73E-01 | -1.65E+00 | 1.80E-03 |
| NM_054094 | Acsm1 | 2.09E+02 | 4.00E+01 | -1.65E+00 | 3.13E-04 |
| NM_172826 | Dact2 | 2.63E+00 | 5.00E-01 | -1.66E+00 | 3.22E-03 |
| NM_008330 | Ifi47 | 2.76E+01 | 5.26E+00 | -1.66E+00 | 4.69E-05 |
| NM_172755 | Sugp2 | 2.31E+00 | 4.39E-01 | -1.66E+00 | 2.02E-03 |
| NM_013631 | Pklr | 4.07E+00 | 7.75E-01 | -1.66E+00 | 1.27E-03 |
| NM_028802 | Gpcpd1 | 2.15E+01 | 4.08E+00 | -1.66E+00 | 2.00E-05 |
| NM_028223 | Tmem175 | 2.32E+00 | 4.39E-01 | -1.66E+00 | 3.17E-03 |
| NM_019414 | Selenbp2 | 3.72E+02 | 7.04E+01 | -1.67E+00 | 1.14E-03 |
| NR_028405 | Marcksl1-ps4 | 1.72E+01 | 3.25E+00 | -1.67E+00 | 5.85E-04 |
| NM_001204334 | Cyp4f14 | 2.31E+01 | 4.36E+00 | -1.67E+00 | 4.03E-05 |
| NM_146083 | Srsf7 | 3.50E+00 | 6.61E-01 | -1.67E+00 | 2.57E-03 |
| NM_001110201 | Yif1b | 1.30E+01 | 2.46E+00 | -1.67E+00 | 9.03E-04 |
| NM_178041 | Eif5 | 6.58E+00 | 1.24E+00 | -1.67E+00 | 1.74E-04 |
| NM_009048 | Reps1 | 3.95E+00 | 7.42E-01 | -1.67E+00 | 1.45E-03 |
| NM_009286 | Sult2a2 | 5.90E+01 | 1.11E+01 | -1.67E+00 | 7.75E-05 |
| NM_001042671 | Gpcpd1 | 3.41E+01 | 6.39E+00 | -1.68E+00 | 1.76E-05 |
| NM_001164838 | Lrrfip2 | 5.13E+00 | 9.58E-01 | -1.68E+00 | 4.85E-04 |
| NM_144855 | Cbs | 4.60E+01 | 8.58E+00 | -1.68E+00 | 1.58E-05 |
| NM_001199568 | Xylb | 4.68E+00 | 8.68E-01 | -1.68E+00 | 3.51E-04 |
| NM_001190490 | Dmpk | 2.91E+00 | 5.39E-01 | -1.68E+00 | 2.55E-03 |
| NM_001081308 | Taok3 | 4.33E+00 | 8.03E-01 | -1.68E+00 | 3.42E-04 |
| NM_001177779 | Dlg3 | 1.88E+00 | 3.48E-01 | -1.69E+00 | 2.68E-03 |
| NM_001080948 | Larp4 | 4.69E+00 | 8.68E-01 | -1.69E+00 | 8.46E-05 |
| NM_029419 | Apol7a | 2.81E+01 | 5.20E+00 | -1.69E+00 | 1.72E-05 |
| NM_170598 | Rbm12 | 1.61E+00 | 2.97E-01 | -1.69E+00 | 3.87E-03 |
| NM_028933 | Fam134c | 2.67E+00 | 4.89E-01 | -1.70E+00 | 1.82E-03 |
| NM_181588 | Cmbl | 1.62E+02 | 2.96E+01 | -1.70E+00 | 1.42E-05 |
| NR_003559 | Mrpl48 | 3.28E+01 | 5.97E+00 | -1.70E+00 | 1.42E-04 |
| NM_007833 | Dcn | 3.89E+01 | 7.07E+00 | -1.71E+00 | 1.46E-05 |
| NM_007498 | Atf3 | 2.25E+01 | 4.09E+00 | -1.71E+00 | 3.18E-05 |
| NM_008278 | Hpgd | 8.48E+01 | 1.54E+01 | -1.71E+00 | 1.24E-05 |
| NM_026610 | Ndufb4 | 1.17E+02 | 2.12E+01 | -1.71E+00 | 7.23E-05 |
| NM_025543 | Mcts2 | 1.29E+01 | 2.34E+00 | -1.71E+00 | 1.91E-03 |
| NM_009534 | Yap1 | 8.01E+00 | 1.45E+00 | -1.71E+00 | 6.17E-05 |
| NM_001164071 | Tank | 4.16E+00 | 7.50E-01 | -1.71E+00 | 1.51E-03 |
| NM_001037098 | Nacc2 | 1.21E+00 | 2.17E-01 | -1.72E+00 | 1.65E-03 |
| NM_009114 | S100a9 | 2.41E+01 | 4.30E+00 | -1.72E+00 | 2.27E-03 |
| NM_001130451 | Nfe2l1 | 7.73E+00 | 1.37E+00 | -1.73E+00 | 9.07E-05 |
| NM_001122873 | Ubl7 | 1.35E+01 | 2.40E+00 | -1.73E+00 | 3.44E-04 |
| NR_040650 | Alg11 | 5.87E+00 | 1.04E+00 | -1.73E+00 | 6.75E-05 |
| NM_008131 | Glul | 2.36E+02 | 4.17E+01 | -1.73E+00 | 9.01E-04 |
| NM_001177552 | Bfar | 6.56E+00 | 1.16E+00 | -1.74E+00 | 3.30E-04 |
| NM_023129 | Pln | 6.66E+00 | 1.17E+00 | -1.74E+00 | 3.93E-04 |
| NM_001161356 | Timd2 | 2.94E+00 | 5.17E-01 | -1.74E+00 | 1.45E-03 |
| NM_001168219 | 2310008H09Rik | 2.41E+00 | 4.22E-01 | -1.74E+00 | 7.04E-04 |
| NM_023624 | Lrat | 7.31E+00 | 1.28E+00 | -1.74E+00 | 3.01E-05 |
| NM_001164086 | Homer2 | 2.70E+00 | 4.71E-01 | -1.75E+00 | 5.39E-05 |
| NM_010195 | Lgr5 | 1.35E+00 | 2.35E-01 | -1.75E+00 | 2.75E-03 |
| NM_007555 | Bmp5 | 5.23E+00 | 9.11E-01 | -1.75E+00 | 5.50E-04 |
| NM_001080812 | Cib3 | 1.38E+01 | 2.39E+00 | -1.75E+00 | 2.75E-03 |
| NM_133752 | Opa1 | 8.11E+00 | 1.41E+00 | -1.75E+00 | 1.49E-05 |
| NM_177645 | 1110028C15Rik | 4.83E+00 | 8.39E-01 | -1.75E+00 | 1.30E-04 |
| NM_023523 | Pecr | 1.49E+02 | 2.57E+01 | -1.75E+00 | 7.77E-06 |
| NM_010074 | Dpp4 | 3.22E+01 | 5.56E+00 | -1.75E+00 | 1.01E-05 |
| NM_001159718 | 2-Sep | 1.14E+01 | 1.97E+00 | -1.76E+00 | 3.01E-05 |
| NM_028316 | Zfp444 | 1.18E+00 | 2.04E-01 | -1.76E+00 | 4.26E-03 |
| NM_029555 | Gstk1 | 1.07E+02 | 1.84E+01 | -1.76E+00 | 6.22E-06 |
| NM_001195268 | Dos | 5.26E+00 | 9.05E-01 | -1.76E+00 | 3.26E-04 |
| NM_001159329 | Gtpbp8 | 6.26E+00 | 1.08E+00 | -1.76E+00 | 2.92E-03 |
| NM_001171582 | Mars | 7.45E+00 | 1.27E+00 | -1.77E+00 | 1.28E-04 |
| NM_001038589 | Usp14 | 2.41E+00 | 4.11E-01 | -1.77E+00 | 9.26E-04 |
| NM_008744 | Ntn1 | 1.99E+00 | 3.39E-01 | -1.77E+00 | 5.95E-04 |
| NM_001085450 | Ctnnd1 | 9.69E+00 | 1.65E+00 | -1.77E+00 | 9.61E-06 |
| NR_040402 | Gm19522 | 3.92E+00 | 6.66E-01 | -1.77E+00 | 9.17E-04 |
| NM_026062 | Fam69a | 4.59E+00 | 7.77E-01 | -1.78E+00 | 5.23E-04 |
| NM_008280 | Lipc | 6.49E+01 | 1.10E+01 | -1.78E+00 | 5.01E-06 |
| NM_001113355 | Vps26a | 3.20E+00 | 5.40E-01 | -1.78E+00 | 8.65E-04 |
| NM_007995 | Fcna | 3.04E+01 | 5.12E+00 | -1.78E+00 | 2.63E-05 |
| NM_145158 | Emilin2 | 1.44E+00 | 2.42E-01 | -1.78E+00 | 3.02E-03 |
| NM_178758 | Acsm5 | 4.70E+01 | 7.91E+00 | -1.78E+00 | 4.51E-06 |
| NM_001042672 | Gpcpd1 | 5.27E+00 | 8.84E-01 | -1.78E+00 | 2.06E-04 |
| NM_001168620 | Enpp5 | 6.56E+00 | 1.10E+00 | -1.79E+00 | 2.52E-04 |
| NR_027515 | Rbm18 | 7.91E+00 | 1.32E+00 | -1.79E+00 | 1.60E-04 |
| NM_008028 | Flot2 | 9.20E+00 | 1.52E+00 | -1.80E+00 | 7.68E-05 |
| NM_007860 | Dio1 | 2.43E+02 | 4.01E+01 | -1.80E+00 | 4.45E-05 |
| NR_030782 | Cyp3a25 | 9.54E+01 | 1.58E+01 | -1.80E+00 | 5.32E-06 |
| NM_001161355 | Timd2 | 4.10E+00 | 6.77E-01 | -1.80E+00 | 4.04E-04 |
| NM_008086 | Gas1 | 1.69E+00 | 2.78E-01 | -1.80E+00 | 3.23E-03 |
| NM_001085409 | Steap3 | 2.26E+00 | 3.72E-01 | -1.80E+00 | 1.69E-03 |
| NM_011921 | Aldh1a7 | 2.20E+02 | 3.62E+01 | -1.81E+00 | 7.82E-05 |
| NM_001199115 | Slc29a1 | 5.65E+00 | 9.23E-01 | -1.81E+00 | 5.47E-04 |
| NM_181582 | Eif5a | 1.01E+02 | 1.64E+01 | -1.82E+00 | 3.02E-06 |
| NM_011336 | Ccl27a | 1.55E+00 | 2.52E-01 | -1.82E+00 | 0.00E+00 |
| NM_001206382 | Mavs | 8.25E+00 | 1.34E+00 | -1.82E+00 | 6.18E-05 |
| NM_001162425 | Efna1 | 4.47E+00 | 7.20E-01 | -1.82E+00 | 2.29E-03 |
| NM_175392 | Fam73b | 4.29E+00 | 6.91E-01 | -1.82E+00 | 2.31E-04 |
| NR_028403 | Ufd1l | 6.44E+00 | 1.04E+00 | -1.83E+00 | 3.98E-04 |
| NM_019759 | Dpt | 4.25E+00 | 6.83E-01 | -1.83E+00 | 1.90E-03 |
| NR_037572 | Dcaf11 | 2.51E+01 | 4.03E+00 | -1.83E+00 | 3.74E-06 |
| NM_144942 | Csad | 3.54E+01 | 5.65E+00 | -1.83E+00 | 2.71E-06 |
| NM_001199122 | Ghitm | 5.05E+00 | 8.06E-01 | -1.84E+00 | 2.26E-04 |
| NR_033745 | 2310028O11Rik | 2.67E+00 | 4.25E-01 | -1.84E+00 | 1.57E-03 |
| NM_001081285 | Mup6 | 1.42E+02 | 2.26E+01 | -1.84E+00 | 5.69E-06 |
| NM_001048147 | Ghr | 1.86E+02 | 2.96E+01 | -1.84E+00 | 3.46E-06 |
| NM_001122642 | Arhgap17 | 1.41E+00 | 2.24E-01 | -1.84E+00 | 3.54E-03 |
| NM_001139513 | Raly | 7.70E+00 | 1.22E+00 | -1.84E+00 | 3.21E-04 |
| NM_133660 | Ces1e | 9.36E+01 | 1.48E+01 | -1.85E+00 | 3.33E-06 |
| NM_147176 | Homer1 | 1.80E+00 | 2.83E-01 | -1.85E+00 | 8.55E-04 |
| NM_010658 | Mafb | 1.38E+01 | 2.17E+00 | -1.85E+00 | 5.94E-06 |
| NM_001102407 | Rbm8a | 5.41E+00 | 8.48E-01 | -1.85E+00 | 2.41E-04 |
| NM_001164678 | Pdcd6ip | 1.16E+01 | 1.81E+00 | -1.85E+00 | 2.48E-06 |
| NR_024093 | U05342 | 9.47E+00 | 1.48E+00 | -1.85E+00 | 2.15E-05 |
| NM_025429 | Serpinb1a | 2.88E+00 | 4.52E-01 | -1.85E+00 | 2.64E-03 |
| NM_001083905 | Fetub | 8.42E+00 | 1.32E+00 | -1.86E+00 | 3.73E-04 |
| NM_011206 | Ptpn18 | 3.05E+00 | 4.76E-01 | -1.86E+00 | 3.61E-03 |
| NM_026731 | Ppp1r14a | 1.28E+01 | 1.99E+00 | -1.86E+00 | 3.60E-03 |
| NM_001103178 | Ablim1 | 1.19E+00 | 1.86E-01 | -1.86E+00 | 1.21E-03 |
| NM_001159502 | Ddx24 | 3.55E+00 | 5.51E-01 | -1.86E+00 | 4.40E-04 |
| NM_001037999 | Dbi | 1.05E+01 | 1.63E+00 | -1.86E+00 | 3.67E-03 |
| NM_011996 | Adh4 | 9.02E+01 | 1.40E+01 | -1.87E+00 | 1.58E-06 |
| NM_001034963 | Sorbs1 | 6.08E+00 | 9.41E-01 | -1.87E+00 | 1.63E-05 |
| NM_019506 | Gdf2 | 7.78E+00 | 1.20E+00 | -1.87E+00 | 4.98E-05 |
| NM_175391 | Apol7c | 3.18E+00 | 4.91E-01 | -1.87E+00 | 1.63E-03 |
| NM_172765 | Zbtb44 | 2.24E+00 | 3.44E-01 | -1.87E+00 | 7.20E-05 |
| NM_001012306 | Hsd3b3 | 6.87E+00 | 1.06E+00 | -1.87E+00 | 5.85E-04 |
| NM_001113283 | BC031353 | 1.21E+01 | 1.85E+00 | -1.88E+00 | 3.16E-06 |
| NM_001166376 | Ms4a6c | 7.73E+00 | 1.18E+00 | -1.88E+00 | 4.11E-03 |
| NM_007494 | Ass1 | 6.55E+02 | 9.96E+01 | -1.88E+00 | 1.74E-03 |
| NM_001145824 | Hipk3 | 9.22E+00 | 1.39E+00 | -1.89E+00 | 1.61E-06 |
| NM_007792 | Csrp2 | 4.66E+01 | 7.01E+00 | -1.89E+00 | 1.13E-05 |
| NM_001114119 | Qrich1 | 7.41E+00 | 1.11E+00 | -1.89E+00 | 3.24E-05 |
| NM_019996 | Phax | 3.30E+01 | 4.96E+00 | -1.90E+00 | 2.01E-06 |
| NM_019466 | Rcan1 | 6.64E+01 | 9.93E+00 | -1.90E+00 | 1.17E-06 |
| NM_021878 | Jarid2 | 9.71E-01 | 1.45E-01 | -1.90E+00 | 1.81E-03 |
| NM_001163311 | Srr | 5.01E+00 | 7.44E-01 | -1.91E+00 | 9.01E-05 |
| NM_010158 | Khdrbs3 | 8.78E+00 | 1.30E+00 | -1.91E+00 | 1.15E-04 |
| NM_019819 | Dusp14 | 3.99E+00 | 5.92E-01 | -1.91E+00 | 2.15E-03 |
| NM_001122641 | Arhgap17 | 1.56E+00 | 2.31E-01 | -1.91E+00 | 2.30E-03 |
| NM_010003 | Cyp2c39 | 3.16E+00 | 4.68E-01 | -1.91E+00 | 1.84E-03 |
| NM_001164038 | Ly6e | 1.27E+02 | 1.88E+01 | -1.91E+00 | 3.21E-06 |
| NM_013485 | C9 | 6.03E+02 | 8.93E+01 | -1.91E+00 | 1.47E-03 |
| NM_019812 | Sirt1 | 2.53E+00 | 3.75E-01 | -1.91E+00 | 4.09E-04 |
| NM_021375 | Rhbg | 6.45E+00 | 9.54E-01 | -1.91E+00 | 2.60E-04 |
| NM_001039515 | Arl4a | 3.80E+00 | 5.61E-01 | -1.91E+00 | 1.63E-04 |
| NM_001177351 | AW112010 | 3.08E+02 | 4.53E+01 | -1.92E+00 | 1.41E-06 |
| NM_001122952 | Nfia | 3.78E+00 | 5.56E-01 | -1.92E+00 | 7.22E-06 |
| NM_029662 | Mfsd2a | 3.14E+01 | 4.61E+00 | -1.92E+00 | 1.41E-06 |
| NM_177562 | Clec16a | 1.42E+00 | 2.07E-01 | -1.92E+00 | 4.92E-04 |
| NM_201256 | Eif4ebp3 | 4.90E+01 | 7.14E+00 | -1.93E+00 | 2.60E-06 |
| NM_001170486 | Arpc4 | 2.64E+00 | 3.85E-01 | -1.93E+00 | 1.81E-03 |
| NM_026282 | Spc24 | 9.29E+00 | 1.35E+00 | -1.93E+00 | 2.69E-04 |
| NM_001111316 | Ptprc | 3.73E+00 | 5.43E-01 | -1.93E+00 | 3.86E-05 |
| NM_008220 | Hbb-b1 | 1.83E+02 | 2.65E+01 | -1.93E+00 | 8.00E-07 |
| NM_010874 | Nat2 | 4.35E+00 | 6.26E-01 | -1.94E+00 | 1.48E-03 |
| NM_001109745 | Cnbp | 8.38E+00 | 1.20E+00 | -1.94E+00 | 7.32E-05 |
| NM_001159964 | Eps15 | 4.81E+00 | 6.88E-01 | -1.94E+00 | 4.07E-05 |
| NM_177606 | Plekhh2 | 5.12E-01 | 7.32E-02 | -1.95E+00 | 4.30E-03 |
| NM_175263 | Notum | 8.80E+00 | 1.26E+00 | -1.95E+00 | 7.86E-05 |
| NM_145392 | Bag2 | 2.83E+00 | 4.05E-01 | -1.95E+00 | 2.12E-03 |
| NM_173375 | Fam180a | 2.58E+00 | 3.69E-01 | -1.95E+00 | 4.30E-03 |
| NM_025386 | Fbxo36 | 8.19E+00 | 1.17E+00 | -1.95E+00 | 1.25E-03 |
| NR_028525 | Snord45c | 1.04E+03 | 1.48E+02 | -1.95E+00 | 0.00E+00 |
| NM_001085522 | Gm13251 | 2.95E+00 | 4.21E-01 | -1.95E+00 | 2.53E-04 |
| NM_001195244 | 2900053A13Rik | 3.77E+01 | 5.35E+00 | -1.95E+00 | 3.89E-06 |
| NM_001114332 | Slc16a10 | 1.53E+01 | 2.17E+00 | -1.95E+00 | 6.55E-07 |
| NM_011802 | Clpx | 1.21E+01 | 1.71E+00 | -1.96E+00 | 6.36E-06 |
| NM_001168386 | Ccdc125 | 5.37E+00 | 7.57E-01 | -1.96E+00 | 1.45E-04 |
| NM_001160107 | Zc3h14 | 3.62E+00 | 5.09E-01 | -1.96E+00 | 2.30E-04 |
| NM_001168541 | Tsku | 1.61E+01 | 2.26E+00 | -1.96E+00 | 2.53E-06 |
| NM_001080132 | Tmpo | 5.77E+00 | 8.10E-01 | -1.96E+00 | 4.94E-05 |
| NM_001184980 | Sult2a5 | 8.05E+00 | 1.13E+00 | -1.96E+00 | 7.25E-04 |
| NM_008769 | Otc | 2.69E+02 | 3.77E+01 | -1.97E+00 | 5.00E-05 |
| NM_008753 | Oaz1 | 9.05E+01 | 1.26E+01 | -1.97E+00 | 5.20E-07 |
| NM_001037877 | Tcf25 | 2.07E+00 | 2.88E-01 | -1.98E+00 | 1.13E-03 |
| NM_001038699 | Fn3k | 9.28E-01 | 1.28E-01 | -1.98E+00 | 3.84E-03 |
| NM_013807 | Plk3 | 5.33E+01 | 7.34E+00 | -1.98E+00 | 3.60E-07 |
| NM_001166410 | Rbm3 | 1.86E+01 | 2.55E+00 | -1.98E+00 | 2.74E-05 |
| NM_024264 | Cyp27a1 | 1.82E+02 | 2.49E+01 | -1.99E+00 | 3.36E-06 |
| NM_001113560 | Glo1 | 1.42E+02 | 1.95E+01 | -1.99E+00 | 3.38E-07 |
| NM_177002 | Slc22a30 | 5.36E+01 | 7.35E+00 | -1.99E+00 | 3.32E-07 |
| NM_001013820 | Slc22a28 | 2.56E+01 | 3.51E+00 | -1.99E+00 | 1.40E-06 |
| NM_011314 | Saa2 | 2.14E+02 | 2.93E+01 | -1.99E+00 | 3.58E-07 |
| NM_001127367 | Dnajb6 | 5.02E+01 | 6.84E+00 | -1.99E+00 | 4.44E-07 |
| NM_008392 | Irg1 | 3.03E+00 | 4.10E-01 | -2.00E+00 | 5.20E-04 |
| NM_007474 | Aqp8 | 1.80E+01 | 2.43E+00 | -2.00E+00 | 1.40E-05 |
| NM_001168226 | Morf4l2 | 2.29E+01 | 3.09E+00 | -2.00E+00 | 2.45E-06 |
| NM_001122685 | Rhbdd1 | 2.78E+00 | 3.76E-01 | -2.00E+00 | 2.38E-04 |
| NM_001001327 | Vkorc1l1 | 1.40E+01 | 1.89E+00 | -2.00E+00 | 1.48E-04 |
| NM_001102405 | Acp5 | 8.34E+00 | 1.12E+00 | -2.01E+00 | 1.98E-04 |
| NM_010479 | Hspa1a | 3.43E+01 | 4.60E+00 | -2.01E+00 | 2.69E-07 |
| NM_001205226 | Cnot1 | 6.07E+00 | 8.10E-01 | -2.01E+00 | 8.13E-07 |
| NM_001111274 | Igf1 | 1.75E+01 | 2.32E+00 | -2.02E+00 | 2.34E-07 |
| NM_009017 | Raet1b | 7.39E+00 | 9.74E-01 | -2.03E+00 | 3.87E-04 |
| NM_015780 | Cfhr1 | 1.37E+02 | 1.80E+01 | -2.03E+00 | 5.01E-07 |
| NM_173743 | Apol9b | 6.51E+01 | 8.56E+00 | -2.03E+00 | 2.19E-07 |
| NM_177380 | Cyp3a44 | 7.94E+01 | 1.04E+01 | -2.03E+00 | 2.30E-07 |
| NM_001110327 | Dmtf1 | 6.88E+00 | 9.03E-01 | -2.03E+00 | 1.23E-05 |
| NM_022434 | Cyp4f14 | 2.52E+01 | 3.31E+00 | -2.03E+00 | 6.38E-07 |
| NM_173733 | Suox | 3.10E+01 | 4.06E+00 | -2.03E+00 | 3.19E-07 |
| NM_001130454 | Nfe2l1 | 9.23E+00 | 1.20E+00 | -2.04E+00 | 4.00E-06 |
| NM_134127 | Cyp4f15 | 6.67E+01 | 8.69E+00 | -2.04E+00 | 1.76E-07 |
| NM_028772 | Dmgdh | 2.08E+02 | 2.70E+01 | -2.04E+00 | 2.72E-05 |
| NM_011309 | S100a1 | 3.45E+02 | 4.46E+01 | -2.05E+00 | 1.88E-07 |
| NM_144807 | Chpt1 | 4.15E+01 | 5.36E+00 | -2.05E+00 | 1.54E-07 |
| NM_025829 | Eif4e3 | 1.12E+01 | 1.45E+00 | -2.05E+00 | 6.79E-06 |
| NM_001025582 | Dram2 | 9.20E+00 | 1.19E+00 | -2.05E+00 | 3.70E-05 |
| NM_001168622 | Znrf1 | 4.14E+00 | 5.35E-01 | -2.05E+00 | 1.36E-05 |
| NR_038075 | Parp6 | 2.17E+00 | 2.79E-01 | -2.05E+00 | 1.04E-03 |
| NM_001083904 | Fetub | 2.08E+02 | 2.66E+01 | -2.06E+00 | 4.74E-07 |
| NM_009080 | Rpl26 | 1.10E+02 | 1.40E+01 | -2.06E+00 | 1.52E-06 |
| NR_037622 | Cep57 | 3.02E+00 | 3.84E-01 | -2.06E+00 | 3.02E-04 |
| NR_003145 | Snhg10 | 1.08E+01 | 1.37E+00 | -2.06E+00 | 2.82E-03 |
| NM_001164466 | Dpys | 7.85E+01 | 9.94E+00 | -2.07E+00 | 1.39E-07 |
| NM_172460 | Nphp3 | 1.83E+00 | 2.31E-01 | -2.07E+00 | 2.85E-03 |
| NM_027147 | Enho | 6.26E+00 | 7.88E-01 | -2.07E+00 | 4.65E-04 |
| NM_133960 | Ces2a | 1.36E+02 | 1.70E+01 | -2.08E+00 | 4.18E-07 |
| NR_027977 | Rai12 | 6.34E+00 | 7.93E-01 | -2.08E+00 | 5.10E-04 |
| NM_001242358 | Trnt1 | 1.45E+01 | 1.81E+00 | -2.08E+00 | 2.92E-06 |
| NM_201641 | Ugt1a10 | 9.46E-01 | 1.18E-01 | -2.08E+00 | 4.25E-03 |
| NM_001134791 | Osbpl9 | 4.59E+00 | 5.67E-01 | -2.09E+00 | 5.14E-05 |
| NM_001014288 | Ptprd | 1.27E+01 | 1.57E+00 | -2.09E+00 | 8.63E-08 |
| NM_194052 | Rtn4 | 1.55E+01 | 1.90E+00 | -2.10E+00 | 1.37E-03 |
| NM_134147 | Macrod1 | 1.73E+01 | 2.11E+00 | -2.10E+00 | 1.46E-05 |
| NM_001077353 | Gsta3 | 2.24E+02 | 2.72E+01 | -2.11E+00 | 3.56E-07 |
| NM_001205239 | Parp6 | 2.15E+00 | 2.62E-01 | -2.11E+00 | 7.05E-04 |
| NM_001190448 | Ddc | 7.34E+00 | 8.90E-01 | -2.11E+00 | 4.75E-05 |
| NM_021301 | Slc15a2 | 1.40E+00 | 1.69E-01 | -2.11E+00 | 7.22E-04 |
| NM_178759 | Timd4 | 3.09E+00 | 3.72E-01 | -2.12E+00 | 5.36E-04 |
| NM_145953 | Cth | 2.41E+02 | 2.90E+01 | -2.12E+00 | 1.44E-06 |
| NR_003270 | Snhg3 | 4.07E+01 | 4.89E+00 | -2.12E+00 | 1.26E-04 |
| NM_008288 | Hsd11b1 | 1.34E+01 | 1.60E+00 | -2.13E+00 | 2.25E-05 |
| NM_001162989 | Phax | 4.10E+01 | 4.87E+00 | -2.13E+00 | 1.08E-07 |
| NM_018808 | Dnajb1 | 1.18E+02 | 1.40E+01 | -2.13E+00 | 1.50E-07 |
| NM_213660 | Stat3 | 1.15E+01 | 1.36E+00 | -2.14E+00 | 2.29E-07 |
| NM_019483 | Smad9 | 1.62E+00 | 1.89E-01 | -2.15E+00 | 1.63E-04 |
| NR_028309 | Serpinb6a | 2.43E+01 | 2.84E+00 | -2.15E+00 | 1.47E-06 |
| NM_001025376 | Wdr61 | 3.36E+01 | 3.91E+00 | -2.15E+00 | 1.16E-06 |
| NM_019975 | Hacl1 | 7.84E+01 | 9.11E+00 | -2.15E+00 | 6.32E-08 |
| NM_145078 | 2610305D13Rik | 2.23E+00 | 2.59E-01 | -2.15E+00 | 6.50E-04 |
| NM_023113 | Aspa | 1.06E+01 | 1.23E+00 | -2.16E+00 | 2.77E-05 |
| NM_008295 | Hsd3b5 | 4.83E+01 | 5.57E+00 | -2.16E+00 | 6.51E-08 |
| NM_001159565 | Dctn3 | 1.35E+01 | 1.56E+00 | -2.16E+00 | 1.15E-04 |
| NM_175283 | Srd5a1 | 3.34E+01 | 3.84E+00 | -2.16E+00 | 4.31E-08 |
| NM_011324 | Scnn1a | 3.23E+00 | 3.71E-01 | -2.16E+00 | 6.97E-05 |
| NM_030069 | Fam55b | 9.01E+00 | 1.03E+00 | -2.17E+00 | 1.59E-06 |
| NM_007447 | Ang | 4.01E+01 | 4.57E+00 | -2.17E+00 | 5.94E-07 |
| NM_009349 | Inmt | 6.58E+02 | 7.50E+01 | -2.17E+00 | 4.48E-06 |
| NM_027626 | Psd3 | 6.44E-01 | 7.27E-02 | -2.18E+00 | 4.12E-04 |
| NM_001170571 | Fam47e | 2.50E+01 | 2.81E+00 | -2.19E+00 | 1.47E-06 |
| NM_008087 | Gas2 | 2.06E+01 | 2.32E+00 | -2.19E+00 | 3.34E-07 |
| NM_009117 | Saa1 | 6.03E+02 | 6.73E+01 | -2.19E+00 | 6.79E-08 |
| NM_008340 | Igfals | 2.57E+01 | 2.86E+00 | -2.20E+00 | 1.35E-07 |
| NM_024201 | Ccdc127 | 7.64E+00 | 8.49E-01 | -2.20E+00 | 2.12E-06 |
| NM_001102446 | Alas2 | 1.64E+01 | 1.82E+00 | -2.20E+00 | 1.20E-06 |
| NM_016672 | Ddc | 2.59E+00 | 2.88E-01 | -2.20E+00 | 8.23E-04 |
| NM_010007 | Cyp2j5 | 1.89E+02 | 2.10E+01 | -2.20E+00 | 1.43E-05 |
| NM_001166249 | Mgll | 9.28E+00 | 1.02E+00 | -2.20E+00 | 8.90E-07 |
| NM_009686 | Apbb2 | 1.56E-02 | 1.72E-03 | -2.20E+00 | 0.00E+00 |
| NM_001185164 | Vegfb | 5.52E+00 | 6.09E-01 | -2.20E+00 | 4.74E-04 |
| NM_001204166 | Rapgef4 | 1.33E+01 | 1.47E+00 | -2.20E+00 | 1.03E-07 |
| NM_028596 | Fbrsl1 | 1.76E+00 | 1.94E-01 | -2.21E+00 | 3.06E-04 |
| NM_001081029 | 4930420K17Rik | 1.47E+02 | 1.62E+01 | -2.21E+00 | 1.67E-08 |
| NM_138654 | 5033411D12Rik | 1.23E+01 | 1.34E+00 | -2.21E+00 | 6.39E-06 |
| NM_001163290 | Adck3 | 2.50E+01 | 2.73E+00 | -2.21E+00 | 1.81E-08 |
| NM_183167 | AI987944 | 7.64E+00 | 8.35E-01 | -2.21E+00 | 5.96E-06 |
| NM_152811 | Ugt2b1 | 3.02E+02 | 3.30E+01 | -2.21E+00 | 1.51E-05 |
| NM_008777 | Pah | 6.95E+02 | 7.58E+01 | -2.22E+00 | 2.47E-03 |
| NM_001111275 | Igf1 | 7.72E+01 | 8.40E+00 | -2.22E+00 | 2.95E-06 |
| NM_029846 | Atg16l1 | 4.55E+00 | 4.94E-01 | -2.22E+00 | 2.24E-05 |
| NM_001168470 | Tmem93 | 9.97E+00 | 1.08E+00 | -2.22E+00 | 3.43E-05 |
| NM_153141 | Carm1 | 1.12E+01 | 1.21E+00 | -2.23E+00 | 5.11E-07 |
| NM_153508 | Clstn3 | 1.20E+00 | 1.29E-01 | -2.23E+00 | 7.32E-04 |
| NM_177828 | Arhgef37 | 7.53E-01 | 8.07E-02 | -2.23E+00 | 3.59E-03 |
| NM_133882 | C8b | 2.69E+02 | 2.88E+01 | -2.23E+00 | 2.58E-06 |
| NM_011315 | Saa3 | 4.65E+01 | 4.98E+00 | -2.23E+00 | 1.18E-05 |
| NM_023211 | Usmg5 | 5.62E+02 | 5.96E+01 | -2.24E+00 | 1.68E-08 |
| NM_001159638 | 0610012H03Rik | 1.59E+01 | 1.69E+00 | -2.24E+00 | 3.20E-06 |
| NR_040404 | Gm15417 | 4.51E+00 | 4.77E-01 | -2.25E+00 | 1.87E-03 |
| NM_001128605 | Psen2 | 2.68E+01 | 2.83E+00 | -2.25E+00 | 8.59E-08 |
| NM_001198635 | Mtap7 | 7.39E+00 | 7.79E-01 | -2.25E+00 | 1.05E-06 |
| NM_001009550 | Mup21 | 1.01E+03 | 1.07E+02 | -2.25E+00 | 2.11E-06 |
| NM_022027 | Syne1 | 8.55E-01 | 8.99E-02 | -2.25E+00 | 1.06E-03 |
| NM_001195338 | Bbip1 | 8.58E+00 | 9.00E-01 | -2.25E+00 | 3.57E-06 |
| NM_023595 | Dut | 3.16E+00 | 3.31E-01 | -2.26E+00 | 1.78E-04 |
| NM_175523 | Ppm1k | 2.02E+01 | 2.11E+00 | -2.26E+00 | 8.27E-09 |
| NM_183249 | 1100001G20Rik | 1.74E+03 | 1.82E+02 | -2.26E+00 | 3.05E-07 |
| NM_001159497 | Ppm1b | 1.82E+01 | 1.90E+00 | -2.26E+00 | 1.23E-06 |
| NR_033214 | B430212C06Rik | 1.50E+00 | 1.56E-01 | -2.26E+00 | 1.30E-03 |
| NR_040277 | B230378P21Rik | 1.49E+00 | 1.55E-01 | -2.27E+00 | 3.59E-03 |
| NM_053096 | Cml2 | 3.42E+02 | 3.55E+01 | -2.27E+00 | 1.75E-04 |
| NM_184052 | Igf1 | 1.10E+02 | 1.14E+01 | -2.27E+00 | 8.06E-09 |
| NM_022722 | Dpys | 6.13E+00 | 6.29E-01 | -2.28E+00 | 1.23E-05 |
| NM_001083918 | Gm13139 | 2.67E+00 | 2.74E-01 | -2.28E+00 | 1.64E-04 |
| NM_001161824 | Mtx1 | 6.79E+00 | 6.94E-01 | -2.28E+00 | 6.37E-05 |
| NM_027496 | Ankrd33b | 1.62E+00 | 1.64E-01 | -2.28E+00 | 3.18E-03 |
| NM_001001446 | Cyp2c44 | 1.64E+02 | 1.67E+01 | -2.29E+00 | 3.15E-08 |
| NM_008361 | Il1b | 5.75E+00 | 5.80E-01 | -2.29E+00 | 1.80E-04 |
| NM_201245 | Mprip | 3.76E+00 | 3.79E-01 | -2.29E+00 | 3.07E-07 |
| NM_001170984 | Hnrnpc | 1.41E+01 | 1.42E+00 | -2.30E+00 | 1.69E-07 |
| NM_026563 | Sdccag3 | 8.39E+00 | 8.44E-01 | -2.30E+00 | 5.38E-06 |
| NM_024175 | Rps23 | 5.07E+01 | 5.09E+00 | -2.30E+00 | 4.40E-06 |
| NM_027943 | Pdilt | 2.95E+00 | 2.95E-01 | -2.30E+00 | 2.73E-04 |
| NM_146232 | Slc22a26 | 5.91E+00 | 5.88E-01 | -2.31E+00 | 3.15E-05 |
| NM_001161825 | Pex7 | 9.21E+00 | 9.14E-01 | -2.31E+00 | 7.86E-06 |
| NR_030704 | Snord55 | 3.98E+03 | 3.94E+02 | -2.31E+00 | 3.74E-03 |
| NM_011834 | Aadat | 6.80E+01 | 6.72E+00 | -2.31E+00 | 3.59E-09 |
| NM_020282 | Nqo2 | 6.08E+00 | 5.96E-01 | -2.32E+00 | 1.14E-06 |
| NM_001199338 | Apoo | 1.05E+01 | 1.03E+00 | -2.33E+00 | 5.37E-05 |
| NM_029620 | Pcolce2 | 2.52E+00 | 2.45E-01 | -2.33E+00 | 7.23E-04 |
| NM_177406 | Cyp4a12a | 3.63E+01 | 3.51E+00 | -2.34E+00 | 4.88E-09 |
| NM_001164274 | Mga | 2.07E+00 | 1.99E-01 | -2.34E+00 | 4.56E-07 |
| NM_001111296 | Sult2a1 | 4.82E+01 | 4.64E+00 | -2.34E+00 | 8.00E-08 |
| NM_001159589 | Sirt1 | 2.54E+00 | 2.44E-01 | -2.34E+00 | 4.23E-05 |
| NM_010478 | Hspa1b | 7.08E+01 | 6.78E+00 | -2.35E+00 | 3.55E-09 |
| NR_027492 | Hspa13 | 7.72E+00 | 7.37E-01 | -2.35E+00 | 2.54E-07 |
| NM_207216 | Ugt3a1 | 4.63E+01 | 4.40E+00 | -2.35E+00 | 3.06E-09 |
| NR_028096 | Rabggtb | 1.27E+01 | 1.19E+00 | -2.36E+00 | 2.86E-06 |
| NM_001204915 | Reep3 | 7.66E+00 | 7.20E-01 | -2.36E+00 | 5.51E-08 |
| NM_007647 | Entpd5 | 2.33E+01 | 2.19E+00 | -2.37E+00 | 1.84E-09 |
| NM_027211 | Anxa13 | 5.88E+00 | 5.51E-01 | -2.37E+00 | 1.76E-04 |
| NM_008160 | Gpx1 | 1.38E+03 | 1.28E+02 | -2.37E+00 | 1.35E-04 |
| NM_001163476 | Gins1 | 2.24E+00 | 2.07E-01 | -2.38E+00 | 1.53E-03 |
| NM_001190436 | Fau | 8.22E+00 | 7.57E-01 | -2.39E+00 | 5.08E-04 |
| NM_172306 | Cyp4a12b | 2.32E+01 | 2.14E+00 | -2.39E+00 | 1.60E-08 |
| NM_011395 | Slc22a3 | 1.62E+00 | 1.48E-01 | -2.39E+00 | 2.55E-04 |
| NM_016928 | Tlr5 | 8.88E-01 | 8.16E-02 | -2.39E+00 | 1.53E-03 |
| NM_001170395 | Cd163 | 2.87E+00 | 2.59E-01 | -2.40E+00 | 2.51E-03 |
| NM_145629 | Pls3 | 5.83E+00 | 5.27E-01 | -2.40E+00 | 2.15E-06 |
| NM_001159610 | Lrrc57 | 5.15E+00 | 4.63E-01 | -2.41E+00 | 3.58E-03 |
| NR_028142 | Pnpla2 | 9.74E+00 | 8.75E-01 | -2.41E+00 | 4.12E-07 |
| NR_038350 | AV039307 | 8.12E+00 | 7.29E-01 | -2.41E+00 | 4.50E-04 |
| NM_146245 | Lrit1 | 3.33E+00 | 2.97E-01 | -2.42E+00 | 5.85E-06 |
| NM_001159351 | Ube2v2 | 3.75E+00 | 3.28E-01 | -2.43E+00 | 7.30E-07 |
| NR_002899 | Snora70 | 4.95E+02 | 4.33E+01 | -2.43E+00 | 1.92E-03 |
| NM_001042514 | Txnrd1 | 1.05E+01 | 9.21E-01 | -2.44E+00 | 5.94E-08 |
| NR_036582 | Dos | 5.15E+00 | 4.49E-01 | -2.44E+00 | 2.54E-06 |
| NM_001166635 | Mid1ip1 | 1.81E+01 | 1.56E+00 | -2.45E+00 | 6.59E-08 |
| NM_007820 | Cyp3a16 | 9.96E+01 | 8.57E+00 | -2.45E+00 | 5.10E-10 |
| NM_001081957 | Gm11428 | 2.46E+02 | 2.12E+01 | -2.45E+00 | 2.87E-09 |
| NM_010118 | Egr2 | 1.38E+00 | 1.18E-01 | -2.46E+00 | 7.00E-04 |
| NM_145364 | Akr1d1 | 1.13E+02 | 9.67E+00 | -2.46E+00 | 2.21E-09 |
| NM_008097 | Gcdh | 9.83E+01 | 8.31E+00 | -2.47E+00 | 6.29E-10 |
| NM_001042593 | Hbs1l | 2.17E+01 | 1.81E+00 | -2.48E+00 | 3.95E-09 |
| NM_001168514 | Mapk14 | 1.13E+01 | 9.41E-01 | -2.49E+00 | 2.06E-08 |
| NM_013797 | Slco1a1 | 9.50E+01 | 7.85E+00 | -2.49E+00 | 4.34E-09 |
| NR_040368 | Gm13944 | 4.30E+00 | 3.55E-01 | -2.49E+00 | 2.48E-03 |
| NM_001167907 | Gm4952 | 2.46E+01 | 2.02E+00 | -2.50E+00 | 4.81E-08 |
| NM_194066 | Ifi27l1 | 6.47E+01 | 5.32E+00 | -2.50E+00 | 3.79E-09 |
| NM_001159555 | Cd36 | 1.33E+01 | 1.09E+00 | -2.50E+00 | 9.42E-09 |
| NM_029609 | Lhpp | 3.14E+01 | 2.55E+00 | -2.51E+00 | 9.03E-09 |
| NM_001145806 | Capn8 | 1.27E+00 | 1.03E-01 | -2.51E+00 | 1.26E-03 |
| NM_010010 | Cyp46a1 | 1.59E+00 | 1.28E-01 | -2.52E+00 | 6.88E-04 |
| NM_201644 | Ugt1a9 | 1.18E+01 | 9.37E-01 | -2.54E+00 | 2.22E-08 |
| NM_001167750 | Ccdc132 | 1.09E+00 | 8.57E-02 | -2.54E+00 | 3.21E-03 |
| NM_010223 | Fkbp8 | 2.48E+01 | 1.96E+00 | -2.54E+00 | 1.51E-08 |
| NM_001034909 | Gm6034 | 2.00E+00 | 1.58E-01 | -2.54E+00 | 1.18E-03 |
| NM_001085407 | Sdccag3 | 8.47E+00 | 6.63E-01 | -2.55E+00 | 1.38E-06 |
| NM_001163472 | Cyp2d22 | 3.13E+01 | 2.44E+00 | -2.55E+00 | 3.63E-10 |
| NR_033614 | Cyp2c53-ps | 3.37E+00 | 2.63E-01 | -2.55E+00 | 3.86E-04 |
| NM_028201 | 2210009G21Rik | 7.03E+00 | 5.43E-01 | -2.56E+00 | 1.08E-06 |
| NM_001003899 | Tardbp | 7.58E+00 | 5.84E-01 | -2.56E+00 | 3.41E-09 |
| NM_011535 | Tbx3 | 1.18E+00 | 9.04E-02 | -2.57E+00 | 1.03E-04 |
| NM_001163624 | Prepl | 5.59E+00 | 4.29E-01 | -2.57E+00 | 9.89E-07 |
| NM_144845 | Ugt3a2 | 1.98E+02 | 1.52E+01 | -2.57E+00 | 3.35E-09 |
| NM_001163709 | Bri3 | 4.60E+01 | 3.51E+00 | -2.57E+00 | 4.00E-08 |
| NM_197986 | Tmem140 | 7.14E+00 | 5.44E-01 | -2.58E+00 | 7.65E-07 |
| NM_010305 | Gnai1 | 2.16E+00 | 1.64E-01 | -2.58E+00 | 5.72E-05 |
| NM_026808 | Fitm1 | 2.74E+01 | 2.07E+00 | -2.58E+00 | 3.53E-07 |
| NR_027845 | Pex11b | 4.56E+00 | 3.42E-01 | -2.59E+00 | 3.58E-03 |
| NM_130885 | Oxr1 | 7.40E+00 | 5.55E-01 | -2.59E+00 | 3.25E-08 |
| NM_146214 | Tat | 8.84E+02 | 6.62E+01 | -2.59E+00 | 7.30E-04 |
| NM_011044 | Pck1 | 8.77E+02 | 6.55E+01 | -2.59E+00 | 2.19E-03 |
| NM_001163623 | Prepl | 6.35E+00 | 4.74E-01 | -2.60E+00 | 3.05E-07 |
| NM_001111276 | Igf1 | 7.74E+00 | 5.71E-01 | -2.61E+00 | 1.46E-09 |
| NR_034048 | Snora81 | 5.34E+02 | 3.94E+01 | -2.61E+00 | 3.09E-05 |
| NM_008061 | G6pc | 1.99E+02 | 1.47E+01 | -2.61E+00 | 2.28E-09 |
| NM_016857 | Exoc7 | 3.64E+00 | 2.68E-01 | -2.61E+00 | 3.95E-06 |
| NM_001164477 | Ifih1 | 7.44E+00 | 5.47E-01 | -2.61E+00 | 7.30E-09 |
| NR_040766 | NA | 3.20E+00 | 2.33E-01 | -2.62E+00 | 8.98E-06 |
| NM_030611 | Akr1c6 | 1.47E+03 | 1.06E+02 | -2.63E+00 | 2.77E-04 |
| NR_036616 | Srsf9 | 7.48E+00 | 5.37E-01 | -2.63E+00 | 1.94E-05 |
| NM_033564 | Mpv17l | 1.21E+01 | 8.65E-01 | -2.64E+00 | 9.96E-09 |
| NM_001110500 | Canx | 6.90E+01 | 4.93E+00 | -2.64E+00 | 1.11E-10 |
| NM_144511 | Ces3b | 2.55E+02 | 1.81E+01 | -2.65E+00 | 6.42E-09 |
| NM_130890 | Capn8 | 5.82E-01 | 4.12E-02 | -2.65E+00 | 6.43E-04 |
| NM_001166549 | Eif4enif1 | 2.05E+00 | 1.45E-01 | -2.65E+00 | 2.64E-05 |
| NM_001146690 | Chpt1 | 4.36E+01 | 3.06E+00 | -2.66E+00 | 7.45E-10 |
| NM_001077515 | Slc1a2 | 1.04E+01 | 7.30E-01 | -2.66E+00 | 2.85E-11 |
| NM_001024955 | Pik3r1 | 7.08E+00 | 4.89E-01 | -2.67E+00 | 2.73E-09 |
| NM_028351 | Rspo3 | 3.81E+00 | 2.60E-01 | -2.69E+00 | 1.19E-05 |
| NM_028679 | Irak3 | 7.01E+00 | 4.71E-01 | -2.70E+00 | 1.85E-07 |
| NM_182959 | Slc17a8 | 6.35E+00 | 4.22E-01 | -2.71E+00 | 2.14E-08 |
| NM_011042 | Pcbp2 | 3.19E+01 | 2.09E+00 | -2.72E+00 | 3.95E-11 |
| NM_017396 | Cyp3a41a | 1.27E+02 | 8.30E+00 | -2.73E+00 | 1.61E-11 |
| NM_001130153 | Arhgef1 | 1.10E+00 | 7.19E-02 | -2.73E+00 | 4.36E-04 |
| NM_030687 | Slco1a4 | 1.97E+01 | 1.28E+00 | -2.73E+00 | 7.95E-11 |
| NM_009834 | Ccrn4l | 4.35E+01 | 2.82E+00 | -2.74E+00 | 6.93E-12 |
| NM_026348 | Itgb3bp | 1.50E+00 | 9.65E-02 | -2.74E+00 | 1.66E-04 |
| NM_175217 | Mmd2 | 1.96E+00 | 1.26E-01 | -2.74E+00 | 1.66E-04 |
| NM_177787 | Slc15a5 | 1.68E+00 | 1.08E-01 | -2.74E+00 | 1.66E-04 |
| NM_020495 | Slco1b2 | 4.79E+02 | 3.07E+01 | -2.75E+00 | 8.85E-05 |
| NR_029412 | Snora16a | 4.19E+02 | 2.67E+01 | -2.75E+00 | 8.09E-04 |
| NM_001243062 | Nr1i3 | 5.31E+00 | 3.35E-01 | -2.76E+00 | 4.50E-05 |
| NR_002172 | Snord15a | 4.12E+02 | 2.59E+01 | -2.77E+00 | 2.78E-04 |
| NM_011134 | Pon1 | 4.57E+02 | 2.86E+01 | -2.77E+00 | 3.32E-09 |
| NR_028260 | Afg3l1 | 5.51E+00 | 3.41E-01 | -2.78E+00 | 5.04E-08 |
| NM_172952 | Gphn | 4.38E+00 | 2.71E-01 | -2.78E+00 | 6.85E-07 |
| NM_001105159 | Cyp3a41b | 1.24E+02 | 7.68E+00 | -2.78E+00 | 4.88E-12 |
| NM_010011 | Cyp4a10 | 6.93E+01 | 4.27E+00 | -2.79E+00 | 3.48E-12 |
| NM_001159415 | Ces3b | 1.79E+01 | 1.08E+00 | -2.81E+00 | 4.08E-09 |
| NM_001164491 | Ablim3 | 4.88E+00 | 2.93E-01 | -2.81E+00 | 7.63E-08 |
| NM_013808 | Csrp3 | 1.06E+01 | 6.37E-01 | -2.81E+00 | 1.25E-05 |
| NM_001170488 | Tprkb | 2.27E+01 | 1.36E+00 | -2.82E+00 | 3.15E-10 |
| NM_194268 | Onecut2 | 8.39E+00 | 5.01E-01 | -2.82E+00 | 3.89E-12 |
| NM_145741 | Gdf10 | 1.81E+00 | 1.06E-01 | -2.84E+00 | 8.61E-05 |
| NM_009255 | Serpine2 | 9.04E+00 | 5.28E-01 | -2.84E+00 | 1.55E-07 |
| NM_010701 | Lect1 | 6.79E+00 | 3.97E-01 | -2.84E+00 | 6.50E-06 |
| NM_023455 | Nat8 | 3.42E+01 | 1.98E+00 | -2.85E+00 | 1.07E-08 |
| NM_011172 | Prodh | 7.16E+01 | 4.13E+00 | -2.85E+00 | 1.03E-12 |
| NM_028089 | Cyp2c55 | 5.54E+00 | 3.19E-01 | -2.85E+00 | 2.54E-06 |
| NM_001204336 | Cyp4f14 | 1.26E+01 | 7.24E-01 | -2.86E+00 | 1.02E-08 |
| NM_001039209 | Gm13152 | 9.90E+00 | 5.69E-01 | -2.86E+00 | 1.88E-06 |
| NR_038011 | C730036E19Rik | 1.48E+01 | 8.36E-01 | -2.88E+00 | 7.56E-10 |
| NM_001013762 | Gm4952 | 1.21E+01 | 6.82E-01 | -2.88E+00 | 1.19E-07 |
| NR_027648 | Zc3h14 | 5.49E+00 | 3.09E-01 | -2.88E+00 | 2.11E-07 |
| NM_010321 | Gnmt | 1.44E+03 | 8.06E+01 | -2.88E+00 | 2.01E-06 |
| NR_028546 | Snora7a | 1.33E+03 | 7.41E+01 | -2.89E+00 | 1.07E-05 |
| NM_001164681 | Ces3a | 9.16E+01 | 5.03E+00 | -2.90E+00 | 5.02E-13 |
| NM_028300 | Pih1d2 | 3.92E-01 | 2.12E-02 | -2.92E+00 | 2.36E-03 |
| NM_008488 | Arhgef1 | 1.14E+00 | 6.14E-02 | -2.92E+00 | 2.63E-04 |
| NM_001177607 | 0610010K14Rik | 3.63E-01 | 1.94E-02 | -2.93E+00 | 0.00E+00 |
| NM_010154 | Erbb4 | 7.50E-01 | 4.02E-02 | -2.93E+00 | 5.54E-04 |
| NM_030701 | Niacr1 | 2.62E+00 | 1.40E-01 | -2.93E+00 | 4.60E-05 |
| NM_028275 | 1700112E06Rik | 4.30E+00 | 2.30E-01 | -2.93E+00 | 5.54E-04 |
| NM_153076 | Crygn | 7.60E+00 | 4.07E-01 | -2.93E+00 | 5.54E-04 |
| NM_145941 | Eif4g1 | 3.62E+01 | 1.94E+00 | -2.93E+00 | 3.61E-13 |
| NM_011393 | Slc1a2 | 1.50E+01 | 8.00E-01 | -2.93E+00 | 2.31E-09 |
| NM_009060 | Rgn | 8.68E+02 | 4.58E+01 | -2.94E+00 | 1.07E-06 |
| NM_001163505 | Atl3 | 1.12E+01 | 5.90E-01 | -2.95E+00 | 6.08E-12 |
| NM_007825 | Cyp7b1 | 1.74E+02 | 9.14E+00 | -2.95E+00 | 3.37E-12 |
| NM_008277 | Hpd | 1.33E+03 | 7.01E+01 | -2.95E+00 | 2.03E-05 |
| NM_008745 | Ntrk2 | 6.29E+00 | 3.30E-01 | -2.95E+00 | 1.28E-10 |
| NM_024437 | Nudt7 | 3.85E+02 | 2.01E+01 | -2.95E+00 | 2.37E-12 |
| NR_027664 | Adhfe1 | 2.06E+01 | 1.07E+00 | -2.96E+00 | 1.42E-11 |
| NM_009993 | Cyp1a2 | 3.24E+02 | 1.68E+01 | -2.96E+00 | 1.12E-10 |
| NM_009078 | Rpl19 | 1.24E+02 | 6.35E+00 | -2.97E+00 | 6.45E-12 |
| NM_001163587 | Agxt2l1 | 2.64E+00 | 1.32E-01 | -2.99E+00 | 3.37E-03 |
| NM_001199218 | Slc38a3 | 7.83E+01 | 3.92E+00 | -2.99E+00 | 1.12E-13 |
| NM_001159595 | Ints8 | 1.21E+00 | 6.06E-02 | -2.99E+00 | 3.68E-05 |
| NM_001204167 | Rapgef4 | 1.82E-01 | 9.09E-03 | -3.00E+00 | 6.99E-05 |
| NM_011082 | Pigr | 4.02E+02 | 2.01E+01 | -3.00E+00 | 1.61E-05 |
| NM_001024853 | Timm9 | 9.18E+00 | 4.43E-01 | -3.03E+00 | 7.76E-06 |
| NM_007824 | Cyp7a1 | 6.62E+01 | 3.18E+00 | -3.03E+00 | 1.64E-13 |
| NR_040768 | NA | 3.24E+00 | 1.54E-01 | -3.04E+00 | 3.01E-04 |
| NM_023493 | Cml5 | 8.75E+00 | 4.17E-01 | -3.04E+00 | 1.12E-05 |
| NM_144856 | Slc22a7 | 1.24E+01 | 5.87E-01 | -3.05E+00 | 6.10E-09 |
| NM_001044703 | Zscan21 | 1.70E+00 | 7.98E-02 | -3.06E+00 | 2.93E-04 |
| NM_175250 | 2810007J24Rik | 8.44E+01 | 3.90E+00 | -3.08E+00 | 3.44E-14 |
| NM_001044705 | Zscan21 | 1.74E+00 | 7.92E-02 | -3.09E+00 | 2.74E-04 |
| NM_011817 | Gadd45g | 2.52E+01 | 1.12E+00 | -3.12E+00 | 4.47E-09 |
| NM_008648 | Mup4 | 1.99E+02 | 8.71E+00 | -3.13E+00 | 2.80E-14 |
| NM_001111100 | Lipa | 4.10E+01 | 1.80E+00 | -3.13E+00 | 6.97E-14 |
| NM_198672 | Ces3a | 9.16E+02 | 3.99E+01 | -3.13E+00 | 7.09E-06 |
| NM_007606 | Car3 | 1.87E+03 | 8.07E+01 | -3.14E+00 | 1.28E-03 |
| NM_174857 | Mamdc2 | 1.21E+00 | 5.20E-02 | -3.15E+00 | 1.63E-04 |
| NR_028445 | F930015N05Rik | 9.81E-01 | 4.15E-02 | -3.16E+00 | 4.08E-04 |
| NM_001168471 | Dynll2 | 3.15E+01 | 1.32E+00 | -3.17E+00 | 8.03E-13 |
| NM_010002 | Cyp2c38 | 1.52E+01 | 6.27E-01 | -3.19E+00 | 2.11E-11 |
| NM_008137 | Gna14 | 3.11E+00 | 1.28E-01 | -3.19E+00 | 8.16E-07 |
| NM_001161731 | Ang | 2.75E+02 | 1.13E+01 | -3.19E+00 | 7.99E-15 |
| NM_201239 | Rnase4 | 1.87E+02 | 7.71E+00 | -3.19E+00 | 1.75E-14 |
| NM_011797 | Car14 | 1.40E+01 | 5.66E-01 | -3.21E+00 | 5.72E-09 |
| NM_134246 | Acot3 | 1.56E+01 | 6.33E-01 | -3.21E+00 | 3.31E-11 |
| NM_133664 | Lad1 | 4.36E+00 | 1.75E-01 | -3.21E+00 | 1.68E-07 |
| NM_007815 | Cyp2c29 | 5.63E+02 | 2.26E+01 | -3.22E+00 | 9.95E-10 |
| NM_008760 | Ogn | 1.11E+00 | 4.30E-02 | -3.25E+00 | 1.50E-03 |
| NM_010766 | Marco | 1.30E+01 | 5.03E-01 | -3.25E+00 | 1.25E-09 |
| NM_172950 | Lpin1 | 3.36E+01 | 1.30E+00 | -3.25E+00 | 2.22E-15 |
| NM_016978 | Oat | 2.89E+02 | 1.11E+01 | -3.26E+00 | 1.10E-12 |
| NM_016668 | Bhmt | 1.57E+03 | 5.90E+01 | -3.28E+00 | 1.29E-03 |
| NM_021456 | Ces1g | 1.46E+02 | 5.44E+00 | -3.29E+00 | 4.44E-15 |
| NM_001164441 | Ankrd33b | 3.52E+00 | 1.29E-01 | -3.31E+00 | 3.72E-04 |
| NR_030416 | Mir675 | 9.10E+03 | 3.32E+02 | -3.31E+00 | 1.88E-06 |
| NM_183278 | Fam25c | 1.09E+02 | 3.98E+00 | -3.31E+00 | 6.88E-09 |
| NM_001170848 | Luc7l2 | 1.73E+01 | 6.29E-01 | -3.31E+00 | 4.46E-12 |
| NM_010001 | Cyp2c37 | 2.75E+02 | 9.96E+00 | -3.32E+00 | 1.82E-14 |
| NM_001033819 | 9130409I23Rik | 1.62E+01 | 5.77E-01 | -3.33E+00 | 1.94E-10 |
| NM_007817 | Cyp2f2 | 6.87E+02 | 2.42E+01 | -3.34E+00 | 2.80E-09 |
| NM_026522 | Chid1 | 4.58E+00 | 1.58E-01 | -3.37E+00 | 6.44E-09 |
| NM_001170978 | Abat | 2.68E+01 | 9.15E-01 | -3.38E+00 | 3.33E-15 |
| NM_021472 | Rnase4 | 9.93E+02 | 3.28E+01 | -3.41E+00 | 2.70E-07 |
| NM_001166402 | Tnfaip3 | 1.52E+01 | 4.85E-01 | -3.44E+00 | 7.51E-14 |
| NM_018884 | Pdzrn3 | 2.63E+00 | 8.35E-02 | -3.45E+00 | 3.43E-07 |
| NM_008649 | Mup5 | 2.27E+02 | 7.17E+00 | -3.45E+00 | 2.22E-16 |
| NM_001166654 | Cdkl3 | 4.44E-02 | 1.40E-03 | -3.46E+00 | 8.69E-04 |
| NR_002173 | Snord15b | 3.59E+03 | 1.13E+02 | -3.46E+00 | 3.26E-11 |
| NM_032541 | Hamp | 4.32E+03 | 1.36E+02 | -3.46E+00 | 9.45E-12 |
| NM_011333 | Ccl2 | 6.06E+01 | 1.89E+00 | -3.47E+00 | 6.93E-12 |
| NM_001201416 | Apbb2 | 1.91E-02 | 5.84E-04 | -3.49E+00 | 0.00E+00 |
| NM_029692 | Upp2 | 5.16E+02 | 1.56E+01 | -3.50E+00 | 1.44E-12 |
| NM_001167875 | Cyp2c50 | 2.12E+02 | 6.07E+00 | -3.55E+00 | 0.00E+00 |
| NM_001080797 | G3bp2 | 1.03E+01 | 2.82E-01 | -3.60E+00 | 9.08E-13 |
| NM_001163700 | Nr1h4 | 3.67E+01 | 9.91E-01 | -3.61E+00 | 1.31E-14 |
| NM_013476 | Ar | 1.09E+00 | 2.92E-02 | -3.62E+00 | 1.04E-03 |
| NM_008030 | Fmo3 | 5.05E+00 | 1.35E-01 | -3.62E+00 | 1.30E-07 |
| NM_009398 | Tnfaip6 | 2.18E+00 | 5.83E-02 | -3.62E+00 | 1.04E-03 |
| NM_001165983 | Ubap2l | 1.98E+00 | 5.23E-02 | -3.63E+00 | 1.12E-06 |
| NM_001101586 | Sult2a3 | 3.99E+00 | 1.04E-01 | -3.64E+00 | 2.37E-03 |
| NM_183257 | Hamp2 | 6.05E+02 | 1.52E+01 | -3.68E+00 | 0.00E+00 |
| NM_009429 | Tpt1 | 2.94E+02 | 7.37E+00 | -3.69E+00 | 0.00E+00 |
| NM_001110321 | Cd72 | 4.72E+00 | 1.14E-01 | -3.72E+00 | 1.62E-05 |
| NM_001042613 | Sepp1 | 2.05E+02 | 4.90E+00 | -3.73E+00 | 0.00E+00 |
| NM_175692 | Snhg11 | 1.21E+00 | 2.88E-02 | -3.74E+00 | 3.73E-06 |
| NM_001085509 | Myom3 | 1.27E+00 | 3.03E-02 | -3.74E+00 | 3.73E-06 |
| NM_007811 | Cyp26a1 | 2.17E+01 | 5.17E-01 | -3.74E+00 | 2.26E-12 |
| NM_146118 | Slc25a25 | 3.30E+01 | 7.84E-01 | -3.74E+00 | 0.00E+00 |
| NR_004445 | Snord22 | 4.16E+03 | 9.86E+01 | -3.74E+00 | 3.31E-10 |
| NM_206537 | Cyp2c54 | 2.12E+02 | 4.91E+00 | -3.77E+00 | 0.00E+00 |
| NR_030703 | Snord104 | 4.12E+04 | 9.50E+02 | -3.77E+00 | 2.05E-09 |
| NM_001166656 | Cdkl3 | 5.37E-02 | 1.22E-03 | -3.79E+00 | 2.63E-04 |
| NM_001164192 | Mtm1 | 1.39E+00 | 3.15E-02 | -3.79E+00 | 1.83E-04 |
| NM_198636 | Acss3 | 8.68E-01 | 1.93E-02 | -3.81E+00 | 7.68E-04 |
| NM_001166428 | Hnrnpf | 2.82E+00 | 6.20E-02 | -3.82E+00 | 2.42E-05 |
| NM_001164662 | Cyfip1 | 7.06E+00 | 1.55E-01 | -3.82E+00 | 9.08E-14 |
| NR_015570 | LOC624853 | 8.51E+02 | 1.84E+01 | -3.83E+00 | 0.00E+00 |
| NR_002861 | Serpina4-ps1 | 6.05E+01 | 1.28E+00 | -3.86E+00 | 6.66E-16 |
| NM_172577 | Slc25a21 | 3.55E+00 | 7.45E-02 | -3.86E+00 | 4.75E-04 |
| NM_010012 | Cyp8b1 | 6.44E+01 | 1.31E+00 | -3.90E+00 | 0.00E+00 |
| NR_003555 | Vmn2r29 | 1.09E+00 | 2.18E-02 | -3.91E+00 | 6.85E-04 |
| NM_144796 | Susd4 | 1.12E+01 | 2.22E-01 | -3.92E+00 | 2.86E-10 |
| NR_033587 | Otub2 | 4.46E-01 | 8.58E-03 | -3.95E+00 | 2.88E-03 |
| NM_001199306 | 2810007J24Rik | 4.51E+02 | 8.48E+00 | -3.97E+00 | 6.22E-15 |
| NM_001166655 | Cdkl3 | 5.63E-02 | 1.05E-03 | -3.99E+00 | 1.23E-04 |
| NM_023137 | Ubd | 3.31E+02 | 6.11E+00 | -3.99E+00 | 0.00E+00 |
| NM_012050 | Omd | 3.17E+00 | 5.67E-02 | -4.02E+00 | 1.31E-04 |
| NR_027866 | 5730408K05Rik | 2.63E+01 | 4.51E-01 | -4.07E+00 | 1.82E-04 |
| NR_034050 | Snora44 | 2.65E+03 | 4.37E+01 | -4.10E+00 | 2.29E-07 |
| NM_134144 | Cyp2c50 | 3.51E+02 | 5.48E+00 | -4.16E+00 | 0.00E+00 |
| NR_002858 | Gm4956 | 1.77E+01 | 2.59E-01 | -4.23E+00 | 2.24E-10 |
| NM_001110784 | Xlr3a | 6.80E+00 | 9.41E-02 | -4.28E+00 | 5.76E-07 |
| NM_001008545 | Tardbp | 9.04E+00 | 1.23E-01 | -4.30E+00 | 4.44E-16 |
| NM_001167877 | Cyp2c50 | 3.02E+01 | 4.06E-01 | -4.31E+00 | 1.78E-15 |
| NM_001166657 | Cdkl3 | 5.87E-02 | 7.82E-04 | -4.32E+00 | 3.17E-05 |
| NM_001159573 | Fip1l1 | 1.21E+00 | 1.50E-02 | -4.39E+00 | 0.00E+00 |
| NR_000002 | Snord32a | 1.78E+04 | 2.11E+02 | -4.44E+00 | 3.25E-08 |
| NM_001130152 | Arhgef1 | 1.14E+00 | 1.32E-02 | -4.46E+00 | 0.00E+00 |
| NM_001081067 | A1bg | 4.46E+00 | 5.03E-02 | -4.48E+00 | 2.88E-05 |
| NM_198091 | Usp2 | 7.84E+00 | 8.47E-02 | -4.53E+00 | 3.47E-11 |
| NM_020565 | Sult3a1 | 8.52E+00 | 9.13E-02 | -4.54E+00 | 2.27E-05 |
| NM_001184981 | Sult2a7 | 1.10E+01 | 1.18E-01 | -4.54E+00 | 2.27E-05 |
| NM_013910 | Kdm2b | 4.26E-01 | 4.45E-03 | -4.56E+00 | 4.14E-04 |
| NM_001122647 | Mup10 | 6.74E+03 | 6.96E+01 | -4.57E+00 | 1.65E-03 |
| NM_001190854 | Pdlim5 | 3.00E+00 | 3.03E-02 | -4.60E+00 | 5.89E-08 |
| NM_023135 | Sult1e1 | 6.15E+01 | 6.17E-01 | -4.60E+00 | 0.00E+00 |
| NM_001045550 | Mup2 | 1.17E+03 | 1.18E+01 | -4.60E+00 | 0.00E+00 |
| NR_040321 | 1300015D01Rik | 5.00E+00 | 4.98E-02 | -4.61E+00 | 3.77E-09 |
| NM_001122643 | Arhgap17 | 1.47E+00 | 1.45E-02 | -4.62E+00 | 0.00E+00 |
| NM_021509 | Moxd1 | 1.16E+01 | 1.13E-01 | -4.63E+00 | 5.89E-13 |
| NM_001165984 | Ubap2l | 1.98E+00 | 1.91E-02 | -4.64E+00 | 0.00E+00 |
| NR_027885 | Vaultrc5 | 1.26E+02 | 1.14E+00 | -4.70E+00 | 1.71E-09 |
| NM_008647 | Mup2 | 6.50E+03 | 5.76E+01 | -4.73E+00 | 1.30E-03 |
| NM_001126319 | Mup9 | 6.31E+03 | 5.58E+01 | -4.73E+00 | 7.62E-05 |
| NM_010236 | Fpgs | 1.84E+01 | 1.61E-01 | -4.74E+00 | 8.68E-14 |
| NM_001110193 | Inpp5d | 3.49E-01 | 2.93E-03 | -4.78E+00 | 0.00E+00 |
| NM_001110350 | Sin3a | 1.78E+00 | 1.49E-02 | -4.78E+00 | 9.97E-08 |
| NM_001167873 | Zfp568 | 9.79E-01 | 7.77E-03 | -4.84E+00 | 0.00E+00 |
| NM_010924 | Nnmt | 1.51E+02 | 1.19E+00 | -4.84E+00 | 0.00E+00 |
| NM_001134676 | Mup8 | 5.63E+03 | 4.41E+01 | -4.85E+00 | 1.06E-03 |
| NR_001592 | H19 | 1.14E+02 | 8.92E-01 | -4.85E+00 | 0.00E+00 |
| NM_001164526 | Mup11 | 6.38E+03 | 4.96E+01 | -4.86E+00 | 8.50E-04 |
| NM_001142804 | Acss3 | 2.52E+00 | 1.94E-02 | -4.87E+00 | 7.23E-06 |
| NM_001199020 | Angel2 | 2.12E+00 | 1.58E-02 | -4.90E+00 | 0.00E+00 |
| NM_019809 | Pdlim5 | 9.21E+00 | 6.76E-02 | -4.91E+00 | 1.00E-09 |
| NM_001200006 | Mup17 | 7.05E+03 | 5.08E+01 | -4.93E+00 | 1.95E-03 |
| NM_001199936 | Mup16 | 6.82E+03 | 4.90E+01 | -4.94E+00 | 6.58E-04 |
| NM_144783 | Wt1 | 7.90E+00 | 5.64E-02 | -4.94E+00 | 1.70E-10 |
| NM_001134674 | Mup13 | 6.29E+03 | 4.28E+01 | -4.99E+00 | 1.91E-03 |
| NM_001199995 | Mup12 | 6.36E+03 | 4.31E+01 | -4.99E+00 | 8.17E-04 |
| NM_031188 | Mup1 | 6.33E+03 | 3.96E+01 | -5.07E+00 | 4.69E-04 |
| NM_001001882 | Rtel1 | 3.94E-01 | 2.34E-03 | -5.13E+00 | 2.22E-03 |
| NM_027907 | Agxt2l1 | 2.29E+01 | 1.32E-01 | -5.16E+00 | 1.02E-07 |
| NM_001159395 | Nfkbiz | 1.85E+00 | 1.00E-02 | -5.22E+00 | 1.11E-09 |
| NM_001134644 | LOC100048885 | 1.94E+03 | 1.04E+01 | -5.23E+00 | 3.77E-15 |
| NM_001177407 | Gm8898 | 2.71E+00 | 1.44E-02 | -5.24E+00 | 3.23E-03 |
| NM_007822 | Cyp4a14 | 5.66E+01 | 2.84E-01 | -5.29E+00 | 0.00E+00 |
| NM_176920 | Lrtm1 | 4.65E+00 | 2.29E-02 | -5.31E+00 | 4.03E-12 |
| NM_001190786 | Ddx19b | 1.29E+00 | 6.20E-03 | -5.34E+00 | 8.34E-09 |
| NM_023617 | Aox3 | 1.27E+02 | 6.08E-01 | -5.34E+00 | 0.00E+00 |
| NM_007703 | Elovl3 | 2.06E+01 | 9.73E-02 | -5.36E+00 | 2.54E-12 |
| NR_028088 | Cox18 | 1.60E+00 | 7.23E-03 | -5.40E+00 | 0.00E+00 |
| NM_001166583 | Fam122b | 1.93E-01 | 8.16E-04 | -5.46E+00 | 9.63E-04 |
| NM_001167864 | Hps5 | 8.82E-01 | 3.65E-03 | -5.49E+00 | 0.00E+00 |
| NM_011589 | Timeless | 2.01E-01 | 8.11E-04 | -5.51E+00 | 3.00E-03 |
| NR_029418 | Tmem161b | 1.49E+00 | 5.23E-03 | -5.65E+00 | 2.10E-08 |
| NM_001170332 | Clec4a2 | 7.60E-01 | 2.65E-03 | -5.66E+00 | 4.00E-08 |
| NR_004432 | Rnu12 | 2.52E+03 | 8.20E+00 | -5.73E+00 | 4.35E-08 |
| NM_001163010 | Mup1 | 4.01E+02 | 1.06E+00 | -5.93E+00 | 0.00E+00 |
| NM_001079905 | Repin1 | 1.35E+00 | 3.47E-03 | -5.97E+00 | 1.21E-03 |
| NM_001111290 | Caprin1 | 5.28E+00 | 1.24E-02 | -6.05E+00 | 0.00E+00 |
| NM_001205364 | Clec9a | 2.58E-01 | 5.91E-04 | -6.08E+00 | 2.67E-04 |
| NM_001164482 | Sipa1 | 6.78E-01 | 1.24E-03 | -6.30E+00 | 0.00E+00 |
| NM_001164249 | Tpm1 | 9.48E+00 | 1.49E-03 | -8.76E+00 | 0.00E+00 |
| NM_001001489 | BC021785 | 8.35E-01 | 0.00E+00 | -1.79769e+308 | 3.10E-02 |
| NM_001025261 | Tpd52 | 1.14E+00 | 0.00E+00 | -1.79769e+308 | 2.10E-02 |
| NM_001025262 | Tpd52 | 1.14E+00 | 0.00E+00 | -1.79769e+308 | 2.12E-02 |
| NM_001033227 | Slc5a10 | 1.09E+00 | 0.00E+00 | -1.79769e+308 | 2.48E-02 |
| NM_001034097 | BC096441 | 1.44E+00 | 0.00E+00 | -1.79769e+308 | 1.73E-02 |
| NM_001034866 | Pnldc1 | 7.68E-01 | 0.00E+00 | -1.79769e+308 | 4.16E-02 |
| NM_001039157 | Ube2j2 | 1.14E+00 | 0.00E+00 | -1.79769e+308 | 1.33E-02 |
| NM_001039159 | Ube2j2 | 1.14E+00 | 0.00E+00 | -1.79769e+308 | 1.43E-02 |
| NM_001039390 | Pkig | 2.04E+00 | 0.00E+00 | -1.79769e+308 | 3.07E-02 |
| NM_001080385 | Clta | 2.43E+00 | 0.00E+00 | -1.79769e+308 | 2.27E-02 |
| NM_001110322 | Cd72 | 4.76E+00 | 0.00E+00 | -1.79769e+308 | 7.86E-03 |
| NM_001113530 | Csf1 | 7.59E-01 | 0.00E+00 | -1.79769e+308 | 3.60E-02 |
| NM_001122604 | Dazap1 | 2.72E+00 | 0.00E+00 | -1.79769e+308 | 8.48E-03 |
| NM_001129886 | Gm10731 | 1.27E+00 | 0.00E+00 | -1.79769e+308 | 2.79E-02 |
| NM_001131069 | Rfesd | 9.48E-01 | 0.00E+00 | -1.79769e+308 | 3.98E-02 |
| NM_001146298 | Wac | 7.35E-01 | 0.00E+00 | -1.79769e+308 | 1.39E-02 |
| NM_001159275 | Slc25a2 | 1.09E+00 | 0.00E+00 | -1.79769e+308 | 4.74E-02 |
| NM_001159503 | BC096441 | 1.44E+00 | 0.00E+00 | -1.79769e+308 | 1.74E-02 |
| NM_001160145 | Tmem9 | 3.09E+00 | 0.00E+00 | -1.79769e+308 | 1.02E-02 |
| NM_001163310 | Rit1 | 9.45E-01 | 0.00E+00 | -1.79769e+308 | 2.81E-02 |
| NM_001164215 | Romo1 | 1.43E+01 | 0.00E+00 | -1.79769e+308 | 8.70E-03 |
| NM_001164643 | Trappc9 | 4.77E-01 | 0.00E+00 | -1.79769e+308 | 4.67E-02 |
| NM_001166557 | Abca6 | 1.39E+00 | 0.00E+00 | -1.79769e+308 | 3.29E-02 |
| NM_001170485 | Arpc4 | 1.63E+00 | 0.00E+00 | -1.79769e+308 | 1.64E-02 |
| NM_001204253 | Clec1b | 5.32E+00 | 0.00E+00 | -1.79769e+308 | 1.21E-02 |
| NM_001205287 | Tmem39a | 2.46E+00 | 0.00E+00 | -1.79769e+308 | 6.82E-03 |
| NM_007919 | Cela2a | 2.76E+00 | 0.00E+00 | -1.79769e+308 | 2.93E-02 |
| NM_008191 | Guca2b | 8.63E+00 | 0.00E+00 | -1.79769e+308 | 1.45E-02 |
| NM_008852 | Pitx3 | 1.33E+00 | 0.00E+00 | -1.79769e+308 | 3.83E-02 |
| NM_009582 | Map3k12 | 2.93E-01 | 0.00E+00 | -1.79769e+308 | 4.10E-02 |
| NM_010024 | Dct | 2.40E+00 | 0.00E+00 | -1.79769e+308 | 8.71E-03 |
| NM_010381 | H2-Ea-ps | 8.53E+00 | 0.00E+00 | -1.79769e+308 | 8.71E-03 |
| NM_020564 | Sult5a1 | 6.36E+00 | 0.00E+00 | -1.79769e+308 | 6.75E-03 |
| NM_021338 | Rpl35a | 1.03E+01 | 0.00E+00 | -1.79769e+308 | 1.46E-02 |
| NM_021883 | Tmod1 | 4.76E-01 | 0.00E+00 | -1.79769e+308 | 4.63E-02 |
| NM_026056 | Cap2 | 8.80E-01 | 0.00E+00 | -1.79769e+308 | 1.62E-02 |
| NM_026253 | Lrrc18 | 7.17E-01 | 0.00E+00 | -1.79769e+308 | 3.37E-02 |
| NM_028188 | Rusc1 | 4.49E-01 | 0.00E+00 | -1.79769e+308 | 3.61E-02 |
| NM_028494 | 1700034I23Rik | 6.37E-01 | 0.00E+00 | -1.79769e+308 | 4.31E-02 |
| NM_029588 | 1700012L04Rik | 7.91E+00 | 0.00E+00 | -1.79769e+308 | 2.11E-02 |
| NM_029920 | Mtus2 | 1.55E-01 | 0.00E+00 | -1.79769e+308 | 4.16E-02 |
| NM_146241 | Trhde | 2.07E-01 | 0.00E+00 | -1.79769e+308 | 4.16E-02 |
| NM_173418 | Lrit2 | 1.62E+00 | 0.00E+00 | -1.79769e+308 | 1.08E-02 |
| NM_175305 | Lrrc19 | 1.07E+00 | 0.00E+00 | -1.79769e+308 | 1.82E-02 |
| NM_181279 | Bre | 1.78E+00 | 0.00E+00 | -1.79769e+308 | 2.61E-02 |
| NM_181280 | Bre | 2.91E+00 | 0.00E+00 | -1.79769e+308 | 1.54E-02 |
| NM_194053 | Rtn4 | 1.55E+01 | 0.00E+00 | -1.79769e+308 | 4.04E-02 |
| NM_207676 | Cadm1 | 1.78E+00 | 0.00E+00 | -1.79769e+308 | 4.78E-03 |
| NR_000003 | Snord35a | 1.77E+03 | 0.00E+00 | -1.79769e+308 | 2.92E-02 |
| NR_001277 | Snord33 | 2.44E+03 | 0.00E+00 | -1.79769e+308 | 3.12E-02 |
| NR_004410 | Snord87 | 2.85E+03 | 0.00E+00 | -1.79769e+308 | 4.70E-02 |
| NR_024198 | Rprl3 | 1.40E+02 | 0.00E+00 | -1.79769e+308 | 5.10E-03 |
| NR_028098 | Rabggtb | 2.52E+00 | 0.00E+00 | -1.79769e+308 | 1.94E-02 |
| NR_028282 | Snord83b | 2.81E+03 | 0.00E+00 | -1.79769e+308 | 3.97E-02 |
| NR_028543 | Snord47 | 2.38E+04 | 0.00E+00 | -1.79769e+308 | 9.12E-03 |
| NR_028548 | Snord16a | 1.24E+03 | 0.00E+00 | -1.79769e+308 | 3.90E-02 |
| NR_028550 | Snord49a | 1.61E+04 | 0.00E+00 | -1.79769e+308 | 2.45E-02 |
| NR_028573 | Snora24 | 2.77E+02 | 0.00E+00 | -1.79769e+308 | 3.77E-02 |
| NR_028577 | D030025P21Rik | 9.91E-02 | 0.00E+00 | -1.79769e+308 | 2.28E-02 |
| NR_033563 | Gpx2-ps1 | 1.64E+00 | 0.00E+00 | -1.79769e+308 | 4.45E-02 |
| NR_034043 | Snora47 | 3.59E+02 | 0.00E+00 | -1.79769e+308 | 3.10E-02 |
| NR_035461 | Mir1940 | 1.55E+03 | 0.00E+00 | -1.79769e+308 | 1.39E-02 |
| NR_037681 | Snord100 | 9.98E+03 | 0.00E+00 | -1.79769e+308 | 1.95E-02 |
| NR_040580 | 1700094J05Rik | 2.62E+00 | 0.00E+00 | -1.79769e+308 | 2.78E-02 |
| NR_040673 | LOC622167 | 6.29E+00 | 0.00E+00 | -1.79769e+308 | 1.23E-02 |
| NR_040704 | 4930563F08Rik | 1.48E+00 | 0.00E+00 | -1.79769e+308 | 4.16E-02 |
| NM_001001650 | Prss48 | 0.00E+00 | 7.65E-01 | 1.79769e+308 | 1.94E-02 |
| NM_001003672 | Pcdhac2 | 0.00E+00 | 1.03E-01 | 1.79769e+308 | 2.09E-02 |
| NM_001003972 | Senp7 | 0.00E+00 | 9.26E-01 | 1.79769e+308 | 1.04E-02 |
| NM_001003973 | Senp7 | 0.00E+00 | 9.71E-01 | 1.79769e+308 | 1.10E-02 |
| NM_001004155 | 9930012K11Rik | 0.00E+00 | 1.83E-01 | 1.79769e+308 | 4.03E-02 |
| NM_001005342 | Ypel4 | 0.00E+00 | 1.34E+00 | 1.79769e+308 | 1.45E-02 |
| NM_001005511 | Lmtk3 | 0.00E+00 | 3.35E-01 | 1.79769e+308 | 4.86E-03 |
| NM_001005787 | Inadl | 0.00E+00 | 3.45E-01 | 1.79769e+308 | 1.06E-02 |
| NM_001005854 | Gm609 | 0.00E+00 | 3.82E-01 | 1.79769e+308 | 1.45E-02 |
| NM_001008233 | Plekhn1 | 0.00E+00 | 3.66E-01 | 1.79769e+308 | 1.45E-02 |
| NM_001008502 | Bbs12 | 0.00E+00 | 2.92E-01 | 1.79769e+308 | 1.62E-02 |
| NM_001013778 | Fam18a | 0.00E+00 | 3.27E-01 | 1.79769e+308 | 9.29E-03 |
| NM_001024142 | Fbxo10 | 0.00E+00 | 3.31E-01 | 1.79769e+308 | 5.26E-03 |
| NM_001024617 | Inpp4b | 0.00E+00 | 1.04E-01 | 1.79769e+308 | 3.05E-02 |
| NM_001024920 | Trp53i13 | 0.00E+00 | 9.11E-01 | 1.79769e+308 | 8.71E-03 |
| NM_001033254 | Pak6 | 0.00E+00 | 3.20E-01 | 1.79769e+308 | 6.73E-03 |
| NM_001033711 | Evi2a | 0.00E+00 | 2.20E-01 | 1.79769e+308 | 2.62E-02 |
| NM_001033775 | 4933422H20Rik | 0.00E+00 | 6.60E-01 | 1.79769e+308 | 7.84E-03 |
| NM_001033779 | Gm5544 | 0.00E+00 | 3.56E-01 | 1.79769e+308 | 4.16E-02 |
| NM_001033789 | Gm5820 | 0.00E+00 | 1.66E-01 | 1.79769e+308 | 3.96E-02 |
| NM_001033874 | Ak8 | 0.00E+00 | 1.79E-01 | 1.79769e+308 | 4.16E-02 |
| NM_001034895 | Zfr2 | 0.00E+00 | 1.56E-01 | 1.79769e+308 | 2.38E-02 |
| NM_001035531 | Adrbk2 | 0.00E+00 | 9.40E-01 | 1.79769e+308 | 6.78E-03 |
| NM_001039000 | Kif5a | 0.00E+00 | 8.68E-02 | 1.79769e+308 | 2.11E-02 |
| NM_001039202 | Hdhd2 | 0.00E+00 | 2.05E-01 | 1.79769e+308 | 4.28E-02 |
| NM_001042418 | Cabyr | 0.00E+00 | 1.98E-01 | 1.79769e+308 | 4.46E-02 |
| NM_001042451 | Snca | 0.00E+00 | 6.21E-01 | 1.79769e+308 | 1.56E-02 |
| NM_001044380 | Hhipl1 | 0.00E+00 | 4.75E-01 | 1.79769e+308 | 7.84E-03 |
| NM_001080926 | Lrp8 | 0.00E+00 | 2.02E-01 | 1.79769e+308 | 5.32E-03 |
| NM_001081249 | Vcan | 0.00E+00 | 8.81E-02 | 1.79769e+308 | 8.64E-03 |
| NM_001081258 | Kif14 | 0.00E+00 | 7.61E-02 | 1.79769e+308 | 1.62E-02 |
| NM_001081280 | Nlrc3 | 0.00E+00 | 1.05E-01 | 1.79769e+308 | 3.05E-02 |
| NM_001081330 | Dnahc2 | 0.00E+00 | 3.02E-02 | 1.79769e+308 | 3.05E-02 |
| NM_001081435 | Fbxo47 | 0.00E+00 | 4.96E-01 | 1.79769e+308 | 1.45E-02 |
| NM_001081446 | Iqck | 0.00E+00 | 1.76E-01 | 1.79769e+308 | 4.18E-02 |
| NM_001081557 | Camta1 | 0.00E+00 | 1.05E-01 | 1.79769e+308 | 1.13E-02 |
| NM_001085497 | Slc25a43 | 0.00E+00 | 5.39E-01 | 1.79769e+308 | 2.38E-02 |
| NM_001085549 | Gm12824 | 0.00E+00 | 1.27E-01 | 1.79769e+308 | 1.23E-02 |
| NM_001097623 | Trerf1 | 0.00E+00 | 8.68E-02 | 1.79769e+308 | 3.21E-02 |
| NM_001098528 | Kcnb2 | 0.00E+00 | 2.39E-01 | 1.79769e+308 | 7.02E-03 |
| NM_001098799 | Tox2 | 0.00E+00 | 7.20E-01 | 1.79769e+308 | 8.71E-03 |
| NM_001099296 | Grrp1 | 0.00E+00 | 7.97E-01 | 1.79769e+308 | 1.23E-02 |
| NM_001100606 | Adat3 | 0.00E+00 | 7.21E-01 | 1.79769e+308 | 1.11E-02 |
| NM_001101535 | Ccdc8 | 0.00E+00 | 7.91E-01 | 1.79769e+308 | 5.26E-03 |
| NM_001103367 | Rai2 | 0.00E+00 | 1.40E-01 | 1.79769e+308 | 4.48E-02 |
| NM_001104617 | Rdx | 0.00E+00 | 7.55E-01 | 1.79769e+308 | 6.82E-03 |
| NM_001110265 | Ttk | 0.00E+00 | 1.45E-01 | 1.79769e+308 | 3.41E-02 |
| NM_001111317 | BC048609 | 0.00E+00 | 9.00E-01 | 1.79769e+308 | 4.28E-02 |
| NM_001112735 | 9930012K11Rik | 0.00E+00 | 1.83E-01 | 1.79769e+308 | 4.02E-02 |
| NM_001112738 | Atp5c1 | 0.00E+00 | 5.76E-01 | 1.79769e+308 | 1.02E-02 |
| NM_001113384 | Gnao1 | 0.00E+00 | 5.79E-02 | 1.79769e+308 | 3.78E-02 |
| NM_001114174 | Fam189a2 | 0.00E+00 | 3.48E-01 | 1.79769e+308 | 1.23E-02 |
| NM_001114662 | Mfsd4 | 0.00E+00 | 9.19E-02 | 1.79769e+308 | 3.83E-02 |
| NM_001127318 | Gucy2c | 0.00E+00 | 1.54E-01 | 1.79769e+308 | 1.92E-02 |
| NM_001127376 | Wisp3 | 0.00E+00 | 2.28E-01 | 1.79769e+308 | 4.56E-02 |
| NM_001128103 | Ano3 | 0.00E+00 | 1.01E-01 | 1.79769e+308 | 4.57E-02 |
| NM_001136062 | Eno3 | 0.00E+00 | 2.61E-01 | 1.79769e+308 | 4.09E-02 |
| NM_001142728 | Lrrc9 | 0.00E+00 | 8.46E-02 | 1.79769e+308 | 3.10E-02 |
| NM_001142732 | Ttll3 | 0.00E+00 | 2.91E-01 | 1.79769e+308 | 3.43E-02 |
| NM_001145854 | Pak6 | 0.00E+00 | 1.78E-01 | 1.79769e+308 | 1.79E-02 |
| NM_001145931 | Yeats2 | 0.00E+00 | 5.96E-01 | 1.79769e+308 | 6.78E-03 |
| NM_001145949 | Dlgap5 | 0.00E+00 | 1.26E-01 | 1.79769e+308 | 2.15E-02 |
| NM_001145958 | Crocc | 0.00E+00 | 7.07E-02 | 1.79769e+308 | 2.90E-02 |
| NM_001146002 | Txndc2 | 0.00E+00 | 3.14E-01 | 1.79769e+308 | 2.11E-02 |
| NM_001146330 | Gpr52 | 0.00E+00 | 6.53E-01 | 1.79769e+308 | 1.91E-02 |
| NM_001159344 | Casz1 | 0.00E+00 | 8.16E-02 | 1.79769e+308 | 1.70E-02 |
| NM_001159393 | Irf1 | 0.00E+00 | 6.30E-01 | 1.79769e+308 | 7.17E-03 |
| NM_001159618 | Pigp | 0.00E+00 | 1.20E+00 | 1.79769e+308 | 1.42E-02 |
| NM_001159650 | Crybb3 | 0.00E+00 | 1.25E+00 | 1.79769e+308 | 1.50E-02 |
| NM_001160420 | Optc | 0.00E+00 | 3.39E-01 | 1.79769e+308 | 2.49E-02 |
| NM_001160421 | Optc | 0.00E+00 | 2.69E-01 | 1.79769e+308 | 3.01E-02 |
| NM_001161516 | Dctd | 0.00E+00 | 2.33E-01 | 1.79769e+308 | 3.08E-02 |
| NM_001161620 | Mpp7 | 0.00E+00 | 5.71E-01 | 1.79769e+308 | 1.06E-02 |
| NM_001161850 | Sgk1 | 0.00E+00 | 5.30E-01 | 1.79769e+308 | 6.81E-03 |
| NM_001162864 | Ttbk1 | 0.00E+00 | 2.17E-01 | 1.79769e+308 | 5.26E-03 |
| NM_001162909 | Ncrna00085 | 0.00E+00 | 9.98E-01 | 1.79769e+308 | 1.08E-02 |
| NM_001163394 | Evl | 0.00E+00 | 1.98E-01 | 1.79769e+308 | 3.05E-02 |
| NM_001163566 | Crb2 | 0.00E+00 | 4.73E-02 | 1.79769e+308 | 4.31E-02 |
| NM_001163583 | Prom1 | 0.00E+00 | 3.48E-01 | 1.79769e+308 | 9.97E-03 |
| NM_001163728 | 4930563E22Rik | 0.00E+00 | 1.16E-01 | 1.79769e+308 | 3.44E-02 |
| NM_001163833 | Msl3l2 | 0.00E+00 | 5.01E-01 | 1.79769e+308 | 9.62E-03 |
| NM_001164437 | Tmem212 | 0.00E+00 | 9.52E-01 | 1.79769e+308 | 2.38E-02 |
| NM_001164641 | Trappc9 | 0.00E+00 | 1.82E-01 | 1.79769e+308 | 1.22E-02 |
| NM_001164642 | Trappc9 | 0.00E+00 | 2.37E-01 | 1.79769e+308 | 1.53E-02 |
| NM_001164813 | Pet117 | 0.00E+00 | 3.28E+00 | 1.79769e+308 | 3.11E-02 |
| NM_001165929 | Ccdc78 | 0.00E+00 | 2.88E-01 | 1.79769e+308 | 2.92E-02 |
| NM_001166250 | Mgll | 0.00E+00 | 8.62E-01 | 1.79769e+308 | 1.13E-02 |
| NM_001166378 | Armcx1 | 0.00E+00 | 4.52E-01 | 1.79769e+308 | 1.08E-02 |
| NM_001166535 | Hmga1 | 0.00E+00 | 7.58E-01 | 1.79769e+308 | 7.83E-03 |
| NM_001166543 | Hmga1 | 0.00E+00 | 2.69E-01 | 1.79769e+308 | 3.06E-02 |
| NM_001166604 | Chn1 | 0.00E+00 | 9.93E-02 | 1.79769e+308 | 3.59E-02 |
| NM_001166653 | Cdkl3 | 0.00E+00 | 2.06E-01 | 1.79769e+308 | 2.06E-02 |
| NM_001167746 | Dnahc17 | 0.00E+00 | 4.55E-02 | 1.79769e+308 | 1.55E-02 |
| NM_001167768 | 1110057K04Rik | 0.00E+00 | 8.78E-01 | 1.79769e+308 | 1.12E-02 |
| NM_001167818 | Rnls | 0.00E+00 | 3.78E-01 | 1.79769e+308 | 2.14E-02 |
| NM_001167831 | AI846148 | 0.00E+00 | 7.16E-01 | 1.79769e+308 | 9.45E-03 |
| NM_001167888 | Suv420h1 | 0.00E+00 | 9.24E-02 | 1.79769e+308 | 4.88E-02 |
| NM_001167923 | Col6a5 | 0.00E+00 | 1.30E-01 | 1.79769e+308 | 7.54E-03 |
| NM_001167946 | Zfp821 | 0.00E+00 | 5.52E-01 | 1.79769e+308 | 8.74E-03 |
| NM_001168250 | Mtbp | 0.00E+00 | 1.84E-01 | 1.79769e+308 | 1.86E-02 |
| NM_001168318 | Scara5 | 0.00E+00 | 9.81E-02 | 1.79769e+308 | 4.33E-02 |
| NM_001168624 | C2cd4c | 0.00E+00 | 1.89E-01 | 1.79769e+308 | 7.19E-03 |
| NM_001170572 | Fam47e | 0.00E+00 | 2.58E-01 | 1.79769e+308 | 4.23E-02 |
| NM_001170705 | Gm17384 | 0.00E+00 | 2.19E-01 | 1.79769e+308 | 2.38E-02 |
| NM_001172092 | Depdc1a | 0.00E+00 | 1.57E-01 | 1.79769e+308 | 2.38E-02 |
| NM_001177349 | Pydc4 | 0.00E+00 | 1.66E-01 | 1.79769e+308 | 2.99E-02 |
| NM_001177350 | Pydc4 | 0.00E+00 | 1.66E-01 | 1.79769e+308 | 3.09E-02 |
| NM_001177536 | Prdm11 | 0.00E+00 | 2.45E-01 | 1.79769e+308 | 3.05E-02 |
| NM_001177732 | Plch1 | 0.00E+00 | 1.43E-01 | 1.79769e+308 | 1.12E-02 |
| NM_001177733 | Plch1 | 0.00E+00 | 1.50E-01 | 1.79769e+308 | 1.05E-02 |
| NM_001177950 | Rpgr | 0.00E+00 | 8.17E-02 | 1.79769e+308 | 4.52E-02 |
| NM_001177951 | Rpgr | 0.00E+00 | 9.15E-02 | 1.79769e+308 | 4.62E-02 |
| NM_001177988 | Zmym3 | 0.00E+00 | 9.18E-01 | 1.79769e+308 | 7.23E-03 |
| NM_001195258 | Gm14378 | 0.00E+00 | 1.12E+00 | 1.79769e+308 | 9.61E-03 |
| NM_001195537 | Gm10345 | 0.00E+00 | 2.36E-01 | 1.79769e+308 | 3.10E-02 |
| NM_001198565 | Sulf1 | 0.00E+00 | 1.30E-01 | 1.79769e+308 | 1.92E-02 |
| NM_001198566 | Sulf1 | 0.00E+00 | 1.30E-01 | 1.79769e+308 | 1.99E-02 |
| NM_001199433 | Dnmt1 | 0.00E+00 | 5.62E-02 | 1.79769e+308 | 4.22E-02 |
| NM_001205070 | Josd2 | 0.00E+00 | 4.31E-01 | 1.79769e+308 | 3.40E-02 |
| NM_001205331 | Mtap4 | 0.00E+00 | 1.41E-01 | 1.79769e+308 | 2.50E-02 |
| NM_001242360 | Trnt1 | 0.00E+00 | 3.69E-01 | 1.79769e+308 | 1.71E-02 |
| NM_007378 | Abca4 | 0.00E+00 | 9.25E-02 | 1.79769e+308 | 1.62E-02 |
| NM_007490 | Art2a-ps | 0.00E+00 | 4.79E-01 | 1.79769e+308 | 4.28E-02 |
| NM_007504 | Atp2a1 | 0.00E+00 | 1.04E-01 | 1.79769e+308 | 3.82E-02 |
| NM_007558 | Bmp8a | 0.00E+00 | 6.41E-01 | 1.79769e+308 | 8.49E-03 |
| NM_007567 | Bsn | 0.00E+00 | 1.55E-02 | 1.79769e+308 | 4.16E-02 |
| NM_007731 | Col13a1 | 0.00E+00 | 2.72E-01 | 1.79769e+308 | 1.45E-02 |
| NM_007857 | Dhh | 0.00E+00 | 3.01E-01 | 1.79769e+308 | 1.62E-02 |
| NM_007863 | Mpp3 | 0.00E+00 | 1.23E-01 | 1.79769e+308 | 4.03E-02 |
| NM_007933 | Eno3 | 0.00E+00 | 2.61E-01 | 1.79769e+308 | 3.97E-02 |
| NM_007938 | Epha6 | 0.00E+00 | 8.18E-02 | 1.79769e+308 | 3.99E-02 |
| NM_008051 | Fut1 | 0.00E+00 | 4.12E-01 | 1.79769e+308 | 9.61E-03 |
| NM_008447 | Kif5a | 0.00E+00 | 8.99E-02 | 1.79769e+308 | 3.37E-02 |
| NM_008479 | Lag3 | 0.00E+00 | 3.17E-01 | 1.79769e+308 | 1.94E-02 |
| NM_008655 | Gadd45b | 0.00E+00 | 8.44E-01 | 1.79769e+308 | 1.08E-02 |
| NM_008719 | Npas2 | 0.00E+00 | 2.46E-01 | 1.79769e+308 | 9.61E-03 |
| NM_008729 | Ctnnd2 | 0.00E+00 | 5.68E-02 | 1.79769e+308 | 4.03E-02 |
| NM_008858 | Prkd1 | 0.00E+00 | 3.88E-01 | 1.79769e+308 | 5.78E-03 |
| NM_009016 | Raet1a | 0.00E+00 | 5.73E-01 | 1.79769e+308 | 1.34E-02 |
| NM_009185 | Stil | 0.00E+00 | 3.18E-01 | 1.79769e+308 | 4.50E-03 |
| NM_009199 | Slc1a1 | 0.00E+00 | 6.98E-02 | 1.79769e+308 | 4.16E-02 |
| NM_009434 | Phlda2 | 0.00E+00 | 1.99E+00 | 1.79769e+308 | 8.71E-03 |
| NM_009445 | Ttk | 0.00E+00 | 2.58E-01 | 1.79769e+308 | 1.50E-02 |
| NM_009675 | Aoc3 | 0.00E+00 | 2.43E-01 | 1.79769e+308 | 9.28E-03 |
| NM_009704 | Areg | 0.00E+00 | 1.31E+00 | 1.79769e+308 | 6.75E-03 |
| NM_009711 | Artn | 0.00E+00 | 3.30E-01 | 1.79769e+308 | 1.62E-02 |
| NM_009759 | Bmx | 0.00E+00 | 1.75E-01 | 1.79769e+308 | 2.38E-02 |
| NM_009839 | Cct6b | 0.00E+00 | 2.08E-01 | 1.79769e+308 | 4.03E-02 |
| NM_009855 | Cd80 | 0.00E+00 | 3.27E-01 | 1.79769e+308 | 2.38E-02 |
| NM_009863 | Cdc7 | 0.00E+00 | 2.37E-01 | 1.79769e+308 | 1.62E-02 |
| NM_009867 | Cdh4 | 0.00E+00 | 1.86E-01 | 1.79769e+308 | 7.84E-03 |
| NM_009897 | Ckmt1 | 0.00E+00 | 7.65E-01 | 1.79769e+308 | 8.71E-03 |
| NM_009925 | Col10a1 | 0.00E+00 | 1.42E-01 | 1.79769e+308 | 2.93E-02 |
| NM_009943 | Cox6a2 | 0.00E+00 | 1.51E-01 | 1.79769e+308 | 9.51E-03 |
| NM_009978 | Cst8 | 0.00E+00 | 3.10E+00 | 1.79769e+308 | 4.86E-03 |
| NM_010014 | Dab1 | 0.00E+00 | 3.42E-01 | 1.79769e+308 | 3.98E-02 |
| NM_010055 | Dlx3 | 0.00E+00 | 6.11E-01 | 1.79769e+308 | 5.26E-03 |
| NM_010452 | Hoxa3 | 0.00E+00 | 1.78E-01 | 1.79769e+308 | 2.92E-02 |
| NM_010453 | Hoxa5 | 0.00E+00 | 4.17E-01 | 1.79769e+308 | 1.50E-02 |
| NM_010588 | Jag2 | 0.00E+00 | 2.12E-01 | 1.79769e+308 | 1.23E-02 |
| NM_010598 | Kcnab2 | 0.00E+00 | 1.22E-01 | 1.79769e+308 | 3.05E-02 |
| NM_010610 | Kcnma1 | 0.00E+00 | 1.18E-01 | 1.79769e+308 | 1.94E-02 |
| NM_010728 | Lox | 0.00E+00 | 2.64E-01 | 1.79769e+308 | 1.08E-02 |
| NM_010927 | Nos2 | 0.00E+00 | 3.87E-01 | 1.79769e+308 | 5.26E-03 |
| NM_010930 | Nov | 0.00E+00 | 4.15E-01 | 1.79769e+308 | 1.08E-02 |
| NM_011193 | Pstpip1 | 0.00E+00 | 2.47E-01 | 1.79769e+308 | 3.05E-02 |
| NM_011234 | Rad51 | 0.00E+00 | 7.16E-01 | 1.79769e+308 | 5.78E-03 |
| NM_011283 | Rp1 | 0.00E+00 | 1.18E-01 | 1.79769e+308 | 1.13E-02 |
| NM_011345 | Sele | 0.00E+00 | 5.42E-01 | 1.79769e+308 | 5.26E-03 |
| NM_011403 | Slc4a1 | 0.00E+00 | 1.87E-01 | 1.79769e+308 | 1.23E-02 |
| NM_011409 | Slfn3 | 0.00E+00 | 5.56E-01 | 1.79769e+308 | 1.08E-02 |
| NM_011538 | Tbx6 | 0.00E+00 | 3.77E-01 | 1.79769e+308 | 1.94E-02 |
| NM_011789 | Apc2 | 0.00E+00 | 7.00E-02 | 1.79769e+308 | 1.77E-02 |
| NM_011823 | Gpr34 | 0.00E+00 | 3.44E-01 | 1.79769e+308 | 1.87E-02 |
| NM_012026 | Rgnef | 0.00E+00 | 1.61E-01 | 1.79769e+308 | 9.61E-03 |
| NM_013468 | Ankrd1 | 0.00E+00 | 7.83E-01 | 1.79769e+308 | 7.02E-03 |
| NM_013564 | Insl3 | 0.00E+00 | 1.88E+00 | 1.79769e+308 | 1.45E-02 |
| NM_013611 | Nodal | 0.00E+00 | 6.03E-01 | 1.79769e+308 | 7.84E-03 |
| NM_013717 | B9d1 | 0.00E+00 | 5.61E-01 | 1.79769e+308 | 4.03E-02 |
| NM_013874 | Dpf1 | 0.00E+00 | 4.32E-01 | 1.79769e+308 | 1.08E-02 |
| NM_013920 | Hnf4g | 0.00E+00 | 3.28E-01 | 1.79769e+308 | 6.75E-03 |
| NM_013932 | Ddx25 | 0.00E+00 | 6.09E-01 | 1.79769e+308 | 1.08E-02 |
| NM_015764 | Greb1 | 0.00E+00 | 6.21E-02 | 1.79769e+308 | 2.38E-02 |
| NM_016758 | Rgs14 | 0.00E+00 | 2.27E-01 | 1.79769e+308 | 2.38E-02 |
| NM_016925 | Fanca | 0.00E+00 | 2.88E-01 | 1.79769e+308 | 6.86E-03 |
| NM_017407 | Spag5 | 0.00E+00 | 3.60E-01 | 1.79769e+308 | 6.75E-03 |
| NM_017465 | Sult2b1 | 0.00E+00 | 1.75E+00 | 1.79769e+308 | 4.50E-03 |
| NM_019389 | Vcan | 0.00E+00 | 4.98E-02 | 1.79769e+308 | 2.53E-02 |
| NM_019392 | Tyro3 | 0.00E+00 | 3.45E-01 | 1.79769e+308 | 6.75E-03 |
| NM_019461 | Usp27x | 0.00E+00 | 4.03E-01 | 1.79769e+308 | 7.02E-03 |
| NM_020518 | Vsig2 | 0.00E+00 | 6.75E-01 | 1.79769e+308 | 1.62E-02 |
| NM_021050 | Cftr | 0.00E+00 | 9.35E-02 | 1.79769e+308 | 1.94E-02 |
| NM_021307 | Zfp112 | 0.00E+00 | 1.97E-01 | 1.79769e+308 | 1.62E-02 |
| NM_021352 | Crybb3 | 0.00E+00 | 1.10E+00 | 1.79769e+308 | 1.54E-02 |
| NM_021439 | Chst11 | 0.00E+00 | 2.91E-01 | 1.79769e+308 | 4.86E-03 |
| NM_021985 | Tnfrsf18 | 0.00E+00 | 9.72E-01 | 1.79769e+308 | 1.86E-02 |
| NM_022033 | Oxct2a | 0.00E+00 | 2.39E-01 | 1.79769e+308 | 3.25E-02 |
| NM_022983 | Lpar3 | 0.00E+00 | 6.40E-01 | 1.79769e+308 | 5.26E-03 |
| NM_023116 | Cacnb2 | 0.00E+00 | 1.06E-01 | 1.79769e+308 | 4.31E-02 |
| NM_023249 | Ypel1 | 0.00E+00 | 3.23E-01 | 1.79769e+308 | 1.31E-02 |
| NM_025329 | Tctex1d2 | 0.00E+00 | 9.53E-01 | 1.79769e+308 | 3.05E-02 |
| NM_025619 | 1700019L03Rik | 0.00E+00 | 2.62E-01 | 1.79769e+308 | 3.07E-02 |
| NM_025891 | Smarcd3 | 0.00E+00 | 9.11E-01 | 1.79769e+308 | 5.78E-03 |
| NM_025944 | 2810432L12Rik | 0.00E+00 | 2.64E-01 | 1.79769e+308 | 1.45E-02 |
| NM_026012 | Nradd | 0.00E+00 | 8.48E-01 | 1.79769e+308 | 1.23E-02 |
| NM_026222 | Ccdc39 | 0.00E+00 | 9.01E-02 | 1.79769e+308 | 4.29E-02 |
| NM_026260 | Tctn3 | 0.00E+00 | 2.59E-01 | 1.79769e+308 | 1.62E-02 |
| NM_026380 | Rgs8 | 0.00E+00 | 1.46E-01 | 1.79769e+308 | 1.23E-02 |
| NM_026458 | Abca14 | 0.00E+00 | 4.72E-02 | 1.79769e+308 | 4.16E-02 |
| NM_026588 | Stx19 | 0.00E+00 | 2.69E-01 | 1.79769e+308 | 4.79E-02 |
| NM_026648 | Lrrc50 | 0.00E+00 | 1.17E-01 | 1.79769e+308 | 4.33E-02 |
| NM_026700 | Dopey2 | 0.00E+00 | 5.69E-01 | 1.79769e+308 | 3.58E-02 |
| NM_026739 | 9530077C05Rik | 0.00E+00 | 1.67E-01 | 1.79769e+308 | 4.03E-02 |
| NM_026967 | Rhebl1 | 0.00E+00 | 1.21E+00 | 1.79769e+308 | 7.02E-03 |
| NM_027032 | Pacrg | 0.00E+00 | 4.25E-01 | 1.79769e+308 | 2.38E-02 |
| NM_027077 | 1700016C15Rik | 0.00E+00 | 1.79E+00 | 1.79769e+308 | 9.61E-03 |
| NM_027227 | Glod5 | 0.00E+00 | 1.74E+00 | 1.79769e+308 | 1.23E-02 |
| NM_027363 | 2010110P09Rik | 0.00E+00 | 1.60E+00 | 1.79769e+308 | 4.86E-03 |
| NM_027531 | Ppp2r2b | 0.00E+00 | 8.85E-02 | 1.79769e+308 | 4.16E-02 |
| NM_027564 | 4921507P07Rik | 0.00E+00 | 2.94E-01 | 1.79769e+308 | 1.73E-02 |
| NM_027733 | Spata24 | 0.00E+00 | 2.53E+00 | 1.79769e+308 | 6.93E-03 |
| NM_027961 | Wfdc3 | 0.00E+00 | 1.98E+00 | 1.79769e+308 | 7.02E-03 |
| NM_027972 | Ccdc19 | 0.00E+00 | 2.01E-01 | 1.79769e+308 | 4.03E-02 |
| NM_027975 | Fam83d | 0.00E+00 | 7.13E-01 | 1.79769e+308 | 5.26E-03 |
| NM_027982 | Ppm1j | 0.00E+00 | 4.30E-01 | 1.79769e+308 | 1.62E-02 |
| NM_028034 | Tdrd12 | 0.00E+00 | 2.87E-01 | 1.79769e+308 | 3.05E-02 |
| NM_028116 | Pygo1 | 0.00E+00 | 8.99E-01 | 1.79769e+308 | 5.78E-03 |
| NM_028222 | Cdkn3 | 0.00E+00 | 2.43E+00 | 1.79769e+308 | 5.78E-03 |
| NM_028238 | Rab38 | 0.00E+00 | 8.90E-01 | 1.79769e+308 | 7.02E-03 |
| NM_028310 | 2810006K23Rik | 0.00E+00 | 2.06E-01 | 1.79769e+308 | 4.25E-02 |
| NM_028333 | Angptl1 | 0.00E+00 | 1.99E-01 | 1.79769e+308 | 3.11E-02 |
| NM_028392 | Ppp2r2b | 0.00E+00 | 4.84E-02 | 1.79769e+308 | 4.16E-02 |
| NM_028443 | Fam101a | 0.00E+00 | 2.82E-01 | 1.79769e+308 | 3.05E-02 |
| NM_028536 | 1700054N08Rik | 0.00E+00 | 3.63E-01 | 1.79769e+308 | 1.08E-02 |
| NM_028576 | 1700106N22Rik | 0.00E+00 | 7.92E-01 | 1.79769e+308 | 9.61E-03 |
| NM_028834 | 2210009G21Rik | 0.00E+00 | 4.97E-01 | 1.79769e+308 | 1.80E-02 |
| NM_028849 | Cldnd2 | 0.00E+00 | 2.57E+00 | 1.79769e+308 | 8.71E-03 |
| NM_028974 | Kbtbd13 | 0.00E+00 | 5.60E-01 | 1.79769e+308 | 4.86E-03 |
| NM_029195 | Ccdc146 | 0.00E+00 | 2.97E-01 | 1.79769e+308 | 9.75E-03 |
| NM_029249 | 4930547N16Rik | 0.00E+00 | 2.98E-01 | 1.79769e+308 | 9.30E-03 |
| NM_029361 | Wnk2 | 0.00E+00 | 1.78E-01 | 1.79769e+308 | 7.84E-03 |
| NM_029407 | 4933432B09Rik | 0.00E+00 | 4.91E-02 | 1.79769e+308 | 4.16E-02 |
| NM_029485 | Spata24 | 0.00E+00 | 2.66E+00 | 1.79769e+308 | 6.40E-03 |
| NM_029523 | Depdc1a | 0.00E+00 | 1.56E-01 | 1.79769e+308 | 2.40E-02 |
| NM_029599 | Sun5 | 0.00E+00 | 5.14E-01 | 1.79769e+308 | 2.38E-02 |
| NM_029809 | 2310014L17Rik | 0.00E+00 | 2.95E-01 | 1.79769e+308 | 1.08E-02 |
| NM_029835 | 5730590G19Rik | 0.00E+00 | 7.35E-02 | 1.79769e+308 | 2.14E-02 |
| NM_029901 | Akr1c21 | 0.00E+00 | 2.43E-01 | 1.79769e+308 | 4.16E-02 |
| NM_030066 | Armcx1 | 0.00E+00 | 4.52E-01 | 1.79769e+308 | 9.62E-03 |
| NM_030070 | Lrrc9 | 0.00E+00 | 8.82E-02 | 1.79769e+308 | 4.22E-02 |
| NM_030172 | 2610021K21Rik | 0.00E+00 | 1.75E-01 | 1.79769e+308 | 4.03E-02 |
| NM_030244 | Ier5l | 0.00E+00 | 7.21E-01 | 1.79769e+308 | 9.61E-03 |
| NM_031169 | Kcnmb1 | 0.00E+00 | 1.43E-01 | 1.79769e+308 | 1.94E-02 |
| NM_031396 | Cnnm1 | 0.00E+00 | 6.74E-02 | 1.79769e+308 | 4.03E-02 |
| NM_033574 | Pcdhgb1 | 0.00E+00 | 1.37E-01 | 1.79769e+308 | 1.86E-02 |
| NM_033576 | Pcdhgb4 | 0.00E+00 | 2.75E-01 | 1.79769e+308 | 7.60E-03 |
| NM_033577 | Pcdhgb5 | 0.00E+00 | 2.04E-01 | 1.79769e+308 | 1.09E-02 |
| NM_033578 | Pcdhgb6 | 0.00E+00 | 2.95E-01 | 1.79769e+308 | 6.44E-03 |
| NM_033579 | Pcdhgb7 | 0.00E+00 | 1.61E-01 | 1.79769e+308 | 1.49E-02 |
| NM_033584 | Pcdhga1 | 0.00E+00 | 9.00E-02 | 1.79769e+308 | 3.07E-02 |
| NM_033585 | Pcdhga2 | 0.00E+00 | 1.27E-01 | 1.79769e+308 | 2.08E-02 |
| NM_033586 | Pcdhga3 | 0.00E+00 | 2.76E-01 | 1.79769e+308 | 6.76E-03 |
| NM_033587 | Pcdhga4 | 0.00E+00 | 1.14E-01 | 1.79769e+308 | 2.40E-02 |
| NM_033592 | Pcdhga9 | 0.00E+00 | 1.88E-01 | 1.79769e+308 | 1.17E-02 |
| NM_033593 | Pcdhga10 | 0.00E+00 | 1.71E-01 | 1.79769e+308 | 1.39E-02 |
| NM_053134 | Pcdhb9 | 0.00E+00 | 1.41E-01 | 1.79769e+308 | 3.05E-02 |
| NM_053137 | Pcdhb12 | 0.00E+00 | 1.59E-01 | 1.79769e+308 | 2.67E-02 |
| NM_053143 | Pcdhb18 | 0.00E+00 | 1.37E-01 | 1.79769e+308 | 1.59E-02 |
| NM_053169 | Trim16 | 0.00E+00 | 3.09E-01 | 1.79769e+308 | 7.84E-03 |
| NM_053171 | Csmd1 | 0.00E+00 | 2.32E-02 | 1.79769e+308 | 4.03E-02 |
| NM_053173 | Kifc5b | 0.00E+00 | 3.47E-01 | 1.79769e+308 | 1.16E-02 |
| NM_058212 | Dpf3 | 0.00E+00 | 2.61E-01 | 1.79769e+308 | 3.07E-02 |
| NM_130448 | Pcdh18 | 0.00E+00 | 1.48E-01 | 1.79769e+308 | 1.45E-02 |
| NM_130455 | Grin3b | 0.00E+00 | 1.75E-01 | 1.79769e+308 | 2.14E-02 |
| NM_130886 | Card14 | 0.00E+00 | 2.59E-01 | 1.79769e+308 | 9.63E-03 |
| NM_133193 | Il1rl2 | 0.00E+00 | 2.33E-01 | 1.79769e+308 | 3.05E-02 |
| NM_133203 | Klra17 | 0.00E+00 | 3.35E-01 | 1.79769e+308 | 4.03E-02 |
| NM_133229 | Ripply3 | 0.00E+00 | 7.38E-01 | 1.79769e+308 | 9.61E-03 |
| NM_133723 | Asph | 0.00E+00 | 2.12E-01 | 1.79769e+308 | 3.95E-02 |
| NM_134066 | Akr1c18 | 0.00E+00 | 2.69E+02 | 1.79769e+308 | 2.76E-02 |
| NM_138628 | Txlnb | 0.00E+00 | 9.58E-02 | 1.79769e+308 | 3.05E-02 |
| NM_138649 | Syt17 | 0.00E+00 | 1.15E+00 | 1.79769e+308 | 4.50E-03 |
| NM_138674 | Pkhd1l1 | 0.00E+00 | 5.20E-02 | 1.79769e+308 | 1.62E-02 |
| NM_139001 | Cspg4 | 0.00E+00 | 1.45E-01 | 1.79769e+308 | 7.84E-03 |
| NM_144553 | Dlgap5 | 0.00E+00 | 4.04E-01 | 1.79769e+308 | 8.22E-03 |
| NM_144795 | Pycr1 | 0.00E+00 | 5.71E-01 | 1.79769e+308 | 4.86E-03 |
| NM_144912 | Rad9b | 0.00E+00 | 8.21E-01 | 1.79769e+308 | 7.84E-03 |
| NM_145067 | Gucy2c | 0.00E+00 | 1.54E-01 | 1.79769e+308 | 1.98E-02 |
| NM_145389 | BC016579 | 0.00E+00 | 1.64E-01 | 1.79769e+308 | 4.03E-02 |
| NM_145467 | Itgbl1 | 0.00E+00 | 1.14E-01 | 1.79769e+308 | 4.16E-02 |
| NM_145526 | P2rx3 | 0.00E+00 | 1.23E-01 | 1.79769e+308 | 2.38E-02 |
| NM_145548 | Cyp2j13 | 0.00E+00 | 7.82E-02 | 1.79769e+308 | 4.03E-02 |
| NM_145562 | Parm1 | 0.00E+00 | 6.50E-01 | 1.79769e+308 | 7.02E-03 |
| NM_145924 | Cenpi | 0.00E+00 | 2.21E-01 | 1.79769e+308 | 1.62E-02 |
| NM_145969 | Fam116a | 0.00E+00 | 3.15E-01 | 1.79769e+308 | 1.34E-02 |
| NM_146016 | Eml6 | 0.00E+00 | 1.27E-01 | 1.79769e+308 | 9.15E-03 |
| NM_146052 | Lrrc3b | 0.00E+00 | 2.24E-01 | 1.79769e+308 | 4.03E-02 |
| NM_146146 | Lepr | 0.00E+00 | 9.07E-02 | 1.79769e+308 | 3.47E-02 |
| NM_146208 | Neil3 | 0.00E+00 | 3.18E-01 | 1.79769e+308 | 1.62E-02 |
| NM_148942 | Serpinb6c | 0.00E+00 | 6.52E-01 | 1.79769e+308 | 1.45E-02 |
| NM_152813 | Plcd3 | 0.00E+00 | 4.61E-01 | 1.79769e+308 | 6.75E-03 |
| NM_153511 | Il1f9 | 0.00E+00 | 8.47E-01 | 1.79769e+308 | 7.02E-03 |
| NM_153512 | Kcng3 | 0.00E+00 | 2.58E-01 | 1.79769e+308 | 1.23E-02 |
| NM_153519 | Txndc2 | 0.00E+00 | 1.86E-01 | 1.79769e+308 | 4.56E-02 |
| NM_153534 | Adcy2 | 0.00E+00 | 3.05E-01 | 1.79769e+308 | 7.02E-03 |
| NM_153785 | Cdkl3 | 0.00E+00 | 2.06E-01 | 1.79769e+308 | 2.13E-02 |
| NM_172122 | Crocc | 0.00E+00 | 6.12E-02 | 1.79769e+308 | 3.05E-02 |
| NM_172506 | Boc | 0.00E+00 | 1.35E-01 | 1.79769e+308 | 1.94E-02 |
| NM_172522 | Megf11 | 0.00E+00 | 1.10E-01 | 1.79769e+308 | 3.18E-02 |
| NM_172622 | Trerf1 | 0.00E+00 | 8.66E-02 | 1.79769e+308 | 3.48E-02 |
| NM_172728 | Creb5 | 0.00E+00 | 1.22E-01 | 1.79769e+308 | 4.03E-02 |
| NM_172809 | Sacs | 0.00E+00 | 3.63E-01 | 1.79769e+308 | 7.84E-03 |
| NM_172868 | Palm2 | 0.00E+00 | 5.10E-02 | 1.79769e+308 | 3.05E-02 |
| NM_172891 | Styk1 | 0.00E+00 | 4.11E-01 | 1.79769e+308 | 6.75E-03 |
| NM_172913 | Tox3 | 0.00E+00 | 2.45E-01 | 1.79769e+308 | 1.45E-02 |
| NM_173024 | Serpina3b | 0.00E+00 | 7.86E-01 | 1.79769e+308 | 5.26E-03 |
| NM_173029 | Adcy10 | 0.00E+00 | 9.77E-02 | 1.79769e+308 | 2.38E-02 |
| NM_173426 | Fam110b | 0.00E+00 | 1.82E-01 | 1.79769e+308 | 4.03E-02 |
| NM_173449 | 4921523A10Rik | 0.00E+00 | 1.54E-01 | 1.79769e+308 | 3.54E-02 |
| NM_174849 | Agbl5 | 0.00E+00 | 3.00E-01 | 1.79769e+308 | 1.22E-02 |
| NM_174853 | Disc1 | 0.00E+00 | 5.04E-02 | 1.79769e+308 | 2.28E-02 |
| NM_175198 | Prox2 | 0.00E+00 | 4.27E-01 | 1.79769e+308 | 8.71E-03 |
| NM_175276 | Fhod3 | 0.00E+00 | 1.70E-01 | 1.79769e+308 | 1.07E-02 |
| NM_175347 | Srl | 0.00E+00 | 2.22E-01 | 1.79769e+308 | 8.71E-03 |
| NM_175448 | Clvs2 | 0.00E+00 | 2.84E-01 | 1.79769e+308 | 1.62E-02 |
| NM_175486 | 6430571L13Rik | 0.00E+00 | 3.47E-01 | 1.79769e+308 | 1.23E-02 |
| NM_175514 | Fam171b | 0.00E+00 | 1.79E-01 | 1.79769e+308 | 1.94E-02 |
| NM_175563 | Prr11 | 0.00E+00 | 4.27E-01 | 1.79769e+308 | 4.86E-03 |
| NM_175645 | Xylt1 | 0.00E+00 | 2.27E-01 | 1.79769e+308 | 1.62E-02 |
| NM_175752 | Chn1 | 0.00E+00 | 1.15E-01 | 1.79769e+308 | 2.93E-02 |
| NM_176966 | Fsd1l | 0.00E+00 | 2.28E-01 | 1.79769e+308 | 2.68E-02 |
| NM_176993 | Mpzl3 | 0.00E+00 | 4.38E-01 | 1.79769e+308 | 6.45E-03 |
| NM_177059 | Fstl4 | 0.00E+00 | 3.08E-01 | 1.79769e+308 | 1.08E-02 |
| NM_177115 | 3-Mar | 0.00E+00 | 6.31E-01 | 1.79769e+308 | 9.61E-03 |
| NM_177167 | Ppm1e | 0.00E+00 | 9.33E-02 | 1.79769e+308 | 1.94E-02 |
| NM_177222 | Casc1 | 0.00E+00 | 1.68E-01 | 1.79769e+308 | 3.05E-02 |
| NM_177265 | 6330512M04Rik | 0.00E+00 | 8.08E-02 | 1.79769e+308 | 4.16E-02 |
| NM_177384 | Ttc16 | 0.00E+00 | 1.33E-01 | 1.79769e+308 | 3.97E-02 |
| NM_177471 | Ccdc69 | 0.00E+00 | 6.70E-01 | 1.79769e+308 | 1.23E-02 |
| NM_177722 | 6030422M02Rik | 0.00E+00 | 1.39E-01 | 1.79769e+308 | 4.03E-02 |
| NM_177879 | Sdk1 | 0.00E+00 | 3.92E-02 | 1.79769e+308 | 4.03E-02 |
| NM_177889 | Zfp82 | 0.00E+00 | 3.48E-01 | 1.79769e+308 | 1.62E-02 |
| NM_177897 | B4galnt4 | 0.00E+00 | 2.53E-01 | 1.79769e+308 | 1.45E-02 |
| NM_178020 | Hyal3 | 0.00E+00 | 3.07E-01 | 1.79769e+308 | 2.33E-02 |
| NM_178047 | Prom2 | 0.00E+00 | 1.54E-01 | 1.79769e+308 | 1.75E-02 |
| NM_178084 | B230120H23Rik | 0.00E+00 | 4.55E-01 | 1.79769e+308 | 2.31E-02 |
| NM_178089 | Hnrnpul1 | 0.00E+00 | 4.24E-01 | 1.79769e+308 | 2.79E-02 |
| NM_178267 | Zfp827 | 0.00E+00 | 1.10E-01 | 1.79769e+308 | 1.23E-02 |
| NM_178609 | E2f7 | 0.00E+00 | 2.78E-01 | 1.79769e+308 | 5.26E-03 |
| NM_178624 | Fbxl2 | 0.00E+00 | 1.38E-01 | 1.79769e+308 | 2.96E-02 |
| NM_178683 | Depdc1b | 0.00E+00 | 1.75E-01 | 1.79769e+308 | 3.05E-02 |
| NM_178753 | Spin4 | 0.00E+00 | 2.87E-01 | 1.79769e+308 | 7.84E-03 |
| NM_178804 | Slit2 | 0.00E+00 | 6.28E-02 | 1.79769e+308 | 2.38E-02 |
| NM_178877 | Nhedc2 | 0.00E+00 | 4.40E-01 | 1.79769e+308 | 1.08E-02 |
| NM_178884 | Obsl1 | 0.00E+00 | 1.32E-01 | 1.79769e+308 | 1.45E-02 |
| NM_178934 | Slc2a12 | 0.00E+00 | 1.06E-01 | 1.79769e+308 | 3.21E-02 |
| NM_180958 | Ccdc79 | 0.00E+00 | 8.90E-02 | 1.79769e+308 | 4.16E-02 |
| NM_181075 | 2610524H06Rik | 0.00E+00 | 1.21E+00 | 1.79769e+308 | 1.08E-02 |
| NM_181419 | Zfp599 | 0.00E+00 | 1.93E-01 | 1.79769e+308 | 1.62E-02 |
| NM_182806 | Gpr18 | 0.00E+00 | 7.36E-01 | 1.79769e+308 | 1.24E-02 |
| NM_183014 | Zfp184 | 0.00E+00 | 3.59E-01 | 1.79769e+308 | 1.08E-02 |
| NM_183031 | Gpr183 | 0.00E+00 | 2.42E-01 | 1.79769e+308 | 1.58E-02 |
| NM_183141 | Elfn2 | 0.00E+00 | 2.97E-01 | 1.79769e+308 | 5.78E-03 |
| NM_183145 | Zfp748 | 0.00E+00 | 5.98E-01 | 1.79769e+308 | 4.33E-02 |
| NM_183160 | E030010A14Rik | 0.00E+00 | 3.60E-01 | 1.79769e+308 | 1.24E-02 |
| NM_183191 | Plch1 | 0.00E+00 | 1.51E-01 | 1.79769e+308 | 1.04E-02 |
| NM_183288 | Arhgap27 | 0.00E+00 | 3.12E-01 | 1.79769e+308 | 1.82E-02 |
| NM_183315 | Ctxn1 | 0.00E+00 | 9.56E-01 | 1.79769e+308 | 9.61E-03 |
| NM_197959 | Kif18b | 0.00E+00 | 2.34E-01 | 1.79769e+308 | 1.45E-02 |
| NM_198111 | Akap6 | 0.00E+00 | 8.91E-02 | 1.79769e+308 | 1.08E-02 |
| NM_198119 | Lrrc24 | 0.00E+00 | 2.16E-01 | 1.79769e+308 | 3.34E-02 |
| NM_198409 | Rai2 | 0.00E+00 | 1.40E-01 | 1.79769e+308 | 4.30E-02 |
| NM_198614 | C2cd4c | 0.00E+00 | 1.89E-01 | 1.79769e+308 | 6.82E-03 |
| NM_198620 | Rundc3b | 0.00E+00 | 3.52E-01 | 1.79769e+308 | 7.02E-03 |
| NM_198654 | Nsl1 | 0.00E+00 | 2.94E-01 | 1.79769e+308 | 1.23E-02 |
| NM_198664 | Tbc1d2 | 0.00E+00 | 3.97E-01 | 1.79769e+308 | 4.50E-03 |
| NM_199223 | Rtn4rl2 | 0.00E+00 | 1.31E+00 | 1.79769e+308 | 5.78E-03 |
| NM_199317 | Phf16 | 0.00E+00 | 5.39E-01 | 1.79769e+308 | 7.02E-03 |
| NM_201367 | Gpr176 | 0.00E+00 | 1.10E-01 | 1.79769e+308 | 3.05E-02 |
| NM_201610 | Neil2 | 0.00E+00 | 4.29E-01 | 1.79769e+308 | 1.45E-02 |
| NM_207204 | Ninl | 0.00E+00 | 1.50E-01 | 1.79769e+308 | 1.45E-02 |
| NM_207205 | Igsf3 | 0.00E+00 | 9.15E-02 | 1.79769e+308 | 1.62E-02 |
| NM_212457 | Bex4 | 0.00E+00 | 1.48E+00 | 1.79769e+308 | 1.15E-02 |
| NR_003630 | Gm6498 | 0.00E+00 | 2.97E-01 | 1.79769e+308 | 2.93E-02 |
| NR_004438 | Apoo-ps | 0.00E+00 | 3.61E+00 | 1.79769e+308 | 5.56E-03 |
| NR_015497 | 9330133O14Rik | 0.00E+00 | 6.88E-01 | 1.79769e+308 | 7.02E-03 |
| NR_015514 | 9330175E14Rik | 0.00E+00 | 6.72E-01 | 1.79769e+308 | 1.08E-02 |
| NR_015515 | 6430562O15Rik | 0.00E+00 | 2.58E-01 | 1.79769e+308 | 3.05E-02 |
| NR_015539 | 9630013A20Rik | 0.00E+00 | 1.77E-01 | 1.79769e+308 | 3.98E-02 |
| NR_015545 | 4930481A15Rik | 0.00E+00 | 5.67E-01 | 1.79769e+308 | 8.61E-03 |
| NR_015556 | 2610035D17Rik | 0.00E+00 | 1.98E-01 | 1.79769e+308 | 4.03E-02 |
| NR_015596 | 4930442L01Rik | 0.00E+00 | 3.62E-01 | 1.79769e+308 | 1.53E-02 |
| NR_015600 | A230072E10Rik | 0.00E+00 | 1.06E-01 | 1.79769e+308 | 4.03E-02 |
| NR_015602 | F730043M19Rik | 0.00E+00 | 1.81E-01 | 1.79769e+308 | 4.16E-02 |
| NR_015604 | E130112N10Rik | 0.00E+00 | 6.08E-01 | 1.79769e+308 | 1.49E-02 |
| NR_015606 | Ppp1r12b | 0.00E+00 | 1.50E-01 | 1.79769e+308 | 3.00E-02 |
| NR_015615 | 4732416N19Rik | 0.00E+00 | 7.39E-01 | 1.79769e+308 | 4.96E-02 |
| NR_024328 | 9230116N13Rik | 0.00E+00 | 2.11E-01 | 1.79769e+308 | 3.05E-02 |
| NR_024720 | 2700099C18Rik | 0.00E+00 | 2.28E-01 | 1.79769e+308 | 4.16E-02 |
| NR_027289 | Gm16119 | 0.00E+00 | 1.44E+00 | 1.79769e+308 | 3.76E-02 |
| NR_027414 | Fbxo34 | 0.00E+00 | 4.62E-01 | 1.79769e+308 | 4.89E-03 |
| NR_027827 | A030009H04Rik | 0.00E+00 | 5.17E-01 | 1.79769e+308 | 1.23E-02 |
| NR_027858 | Nlrp1c-ps | 0.00E+00 | 1.63E-01 | 1.79769e+308 | 2.11E-02 |
| NR_027963 | Zfp783 | 0.00E+00 | 1.34E-01 | 1.79769e+308 | 1.98E-02 |
| NR_027966 | 4930429B21Rik | 0.00E+00 | 1.94E-01 | 1.79769e+308 | 3.73E-02 |
| NR_028125 | 0610038B21Rik | 0.00E+00 | 2.62E-01 | 1.79769e+308 | 3.58E-02 |
| NR_028126 | 6330407A03Rik | 0.00E+00 | 2.25E-01 | 1.79769e+308 | 3.31E-02 |
| NR_028129 | LOC100302567 | 0.00E+00 | 2.22E+02 | 1.79769e+308 | 3.94E-02 |
| NR_028263 | B130006D01Rik | 0.00E+00 | 3.82E-01 | 1.79769e+308 | 7.84E-03 |
| NR_028304 | C4bp-ps1 | 0.00E+00 | 7.40E-01 | 1.79769e+308 | 3.05E-02 |
| NR_028329 | D630032N06Rik | 0.00E+00 | 5.60E-01 | 1.79769e+308 | 4.16E-02 |
| NR_028364 | AI450353 | 0.00E+00 | 3.63E-01 | 1.79769e+308 | 2.48E-02 |
| NR_028384 | 4930528A17Rik | 0.00E+00 | 1.61E-01 | 1.79769e+308 | 4.29E-02 |
| NR_028421 | D430020J02Rik | 0.00E+00 | 3.94E-01 | 1.79769e+308 | 8.71E-03 |
| NR_029456 | A330050B17Rik | 0.00E+00 | 9.90E-02 | 1.79769e+308 | 2.92E-02 |
| NR_029739 | Mir22 | 0.00E+00 | 2.50E+02 | 1.79769e+308 | 3.07E-02 |
| NR_030259 | Mir546 | 0.00E+00 | 2.53E+02 | 1.79769e+308 | 8.62E-03 |
| NR_030669 | 1700034H15Rik | 0.00E+00 | 7.47E-02 | 1.79769e+308 | 4.29E-02 |
| NR_030721 | 9130206I24Rik | 0.00E+00 | 1.83E-01 | 1.79769e+308 | 2.38E-02 |
| NR_030772 | Zfp821 | 0.00E+00 | 5.26E-01 | 1.79769e+308 | 9.30E-03 |
| NR_033147 | D830046C22Rik | 0.00E+00 | 1.90E-01 | 1.79769e+308 | 3.02E-02 |
| NR_033207 | 2010016I18Rik | 0.00E+00 | 2.48E-01 | 1.79769e+308 | 3.79E-02 |
| NR_033496 | Lrp8 | 0.00E+00 | 4.87E-02 | 1.79769e+308 | 4.51E-02 |
| NR_033532 | B230206H07Rik | 0.00E+00 | 2.15E-01 | 1.79769e+308 | 1.86E-02 |
| NR_033555 | Gm10400 | 0.00E+00 | 1.22E-01 | 1.79769e+308 | 2.77E-02 |
| NR_033628 | AA474331 | 0.00E+00 | 1.65E+00 | 1.79769e+308 | 9.86E-03 |
| NR_033637 | Gm4349 | 0.00E+00 | 9.35E-01 | 1.79769e+308 | 1.15E-02 |
| NR_035444 | Mir1907 | 0.00E+00 | 3.43E+02 | 1.79769e+308 | 2.93E-02 |
| NR_036469 | LOC100499420 | 0.00E+00 | 2.66E-01 | 1.79769e+308 | 1.45E-02 |
| NR_037224 | Mir3064 | 0.00E+00 | 4.50E+03 | 1.79769e+308 | 7.70E-03 |
| NR_037955 | 1190002F15Rik | 0.00E+00 | 4.17E-01 | 1.79769e+308 | 2.28E-02 |
| NR_037956 | 1190002F15Rik | 0.00E+00 | 4.16E-01 | 1.79769e+308 | 2.28E-02 |
| NR_038020 | Gm20125 | 0.00E+00 | 1.78E-01 | 1.79769e+308 | 1.94E-02 |
| NR_038058 | C030037D09Rik | 0.00E+00 | 6.69E-01 | 1.79769e+308 | 1.94E-02 |
| NR_038090 | AI506816 | 0.00E+00 | 9.80E-01 | 1.79769e+308 | 4.16E-02 |
| NR_038172 | 4931403G20Rik | 0.00E+00 | 2.77E-01 | 1.79769e+308 | 2.67E-02 |
| NR_039574 | Mir5114 | 0.00E+00 | 9.50E+03 | 1.79769e+308 | 6.82E-03 |
| NR_039582 | Mir5122 | 0.00E+00 | 2.82E+02 | 1.79769e+308 | 4.00E-02 |
| NR_039592 | Mir5130 | 0.00E+00 | 5.29E+02 | 1.79769e+308 | 2.50E-02 |
| NR_040300 | Gm13845 | 0.00E+00 | 7.50E-01 | 1.79769e+308 | 1.94E-02 |
| NR_040328 | 4833419F23Rik | 0.00E+00 | 9.62E-01 | 1.79769e+308 | 1.62E-02 |
| NR_040371 | Gm14023 | 0.00E+00 | 2.51E-01 | 1.79769e+308 | 3.12E-02 |
| NR_040389 | Gm10785 | 0.00E+00 | 3.05E-01 | 1.79769e+308 | 2.63E-02 |
| NR_040390 | A730090N16Rik | 0.00E+00 | 3.66E-01 | 1.79769e+308 | 2.84E-02 |
| NR_040409 | Gm15441 | 0.00E+00 | 3.00E-01 | 1.79769e+308 | 2.76E-02 |
| NR_040432 | Gm15708 | 0.00E+00 | 4.52E-01 | 1.79769e+308 | 1.47E-02 |
| NR_040463 | 9230112J17Rik | 0.00E+00 | 3.72E-01 | 1.79769e+308 | 1.17E-02 |
| NR_040464 | 9230112J17Rik | 0.00E+00 | 3.73E-01 | 1.79769e+308 | 1.31E-02 |
| NR_040505 | 4930405A21Rik | 0.00E+00 | 5.93E-01 | 1.79769e+308 | 2.45E-02 |
| NR_040508 | 1700024P03Rik | 0.00E+00 | 4.88E-01 | 1.79769e+308 | 3.18E-02 |
| NR_040613 | 4930471I20Rik | 0.00E+00 | 8.61E-01 | 1.79769e+308 | 3.37E-02 |
| NR_040614 | 4930533B01Rik | 0.00E+00 | 7.35E-01 | 1.79769e+308 | 1.81E-02 |
| NR_040674 | Gm12505 | 0.00E+00 | 1.03E+00 | 1.79769e+308 | 1.94E-02 |
| NR_040698 | AW046200 | 0.00E+00 | 2.19E-01 | 1.79769e+308 | 4.55E-02 |
| NM_008935 | Prom1 | 1.37E-01 | 1.28E+01 | 4.53E+00 | 3.29E-02 |
| NM_146010 | Tspan8 | 9.25E-01 | 5.24E+01 | 4.04E+00 | 4.51E-03 |
| NM_016966 | Phgdh | 1.14E-01 | 5.11E+00 | 3.80E+00 | 8.42E-03 |
| NM_001163480 | Neurl1a | 1.02E-01 | 4.51E+00 | 3.79E+00 | 1.38E-02 |
| NM_011611 | Cd40 | 5.15E-02 | 1.55E+00 | 3.40E+00 | 6.96E-03 |
| NM_170704 | Cd40 | 5.32E-02 | 1.58E+00 | 3.39E+00 | 6.92E-03 |
| NR_027925 | 2010204K13Rik | 1.22E-01 | 2.64E+00 | 3.08E+00 | 4.76E-03 |
| NM_178076 | Mcf2l | 2.55E-02 | 5.02E-01 | 2.98E+00 | 4.93E-03 |
| NM_001159485 | Mcf2l | 2.59E-02 | 5.03E-01 | 2.97E+00 | 5.15E-03 |
| NM_009554 | Zfp37 | 1.13E-01 | 2.05E+00 | 2.90E+00 | 5.37E-03 |
| NM_001168672 | Gtse1 | 7.60E-02 | 1.38E+00 | 2.90E+00 | 5.90E-03 |
| NM_175681 | Glp2r | 8.14E-02 | 1.45E+00 | 2.88E+00 | 5.78E-03 |
| NM_001082976 | Tc2n | 3.63E-01 | 6.39E+00 | 2.87E+00 | 2.60E-02 |
| NM_008121 | Gja5 | 1.31E-01 | 2.22E+00 | 2.83E+00 | 6.71E-03 |
| NM_013609 | Ngf | 1.03E+00 | 1.73E+01 | 2.82E+00 | 6.59E-03 |
| NM_029942 | Prelid2 | 7.31E-01 | 1.22E+01 | 2.82E+00 | 6.98E-03 |
| NM_031380 | Fstl3 | 8.99E-02 | 1.48E+00 | 2.80E+00 | 8.15E-03 |
| NM_030889 | Sorcs2 | 6.89E-02 | 1.09E+00 | 2.76E+00 | 8.15E-03 |
| NM_013662 | Sema6b | 3.18E-01 | 5.04E+00 | 2.76E+00 | 7.34E-03 |
| NM_016679 | Keap1 | 9.13E-02 | 1.41E+00 | 2.74E+00 | 8.07E-03 |
| NM_173769 | Zfp641 | 1.10E-01 | 1.64E+00 | 2.71E+00 | 9.59E-03 |
| NM_010737 | Klrb1a | 1.86E-01 | 2.78E+00 | 2.71E+00 | 1.05E-02 |
| NM_010818 | Cd200 | 1.77E-01 | 2.62E+00 | 2.69E+00 | 1.00E-02 |
| NM_030166 | Galntl2 | 1.78E-01 | 2.55E+00 | 2.66E+00 | 1.09E-02 |
| NM_008883 | Plxna3 | 5.64E-02 | 7.86E-01 | 2.63E+00 | 1.18E-02 |
| NM_033373 | Krt23 | 2.85E-01 | 3.84E+00 | 2.60E+00 | 1.29E-02 |
| NM_009640 | Angpt1 | 9.82E-02 | 1.18E+00 | 2.49E+00 | 1.79E-02 |
| NM_010892 | Nek2 | 1.27E-01 | 1.47E+00 | 2.45E+00 | 1.96E-02 |
| NM_026741 | Zfp579 | 1.94E-01 | 2.25E+00 | 2.45E+00 | 1.96E-02 |
| NM_001083628 | Greb1l | 4.63E-02 | 5.26E-01 | 2.43E+00 | 2.07E-02 |
| NM_001201413 | Apbb2 | 7.87E-02 | 8.90E-01 | 2.43E+00 | 8.15E-03 |
| NM_177068 | Olfml2b | 1.31E-01 | 1.47E+00 | 2.41E+00 | 2.17E-02 |
| NM_009402 | Pglyrp1 | 7.97E-01 | 8.88E+00 | 2.41E+00 | 2.17E-02 |
| NM_011751 | Zfp207 | 2.02E-01 | 2.24E+00 | 2.41E+00 | 1.70E-02 |
| NM_001080818 | Cdc14a | 4.51E-02 | 4.92E-01 | 2.39E+00 | 2.49E-02 |
| NM_138684 | Wfdc12 | 7.29E-01 | 7.81E+00 | 2.37E+00 | 2.40E-02 |
| NM_001145924 | 5730528L13Rik | 8.71E-02 | 9.10E-01 | 2.35E+00 | 2.91E-02 |
| NM_145927 | Fntb | 1.61E-01 | 1.63E+00 | 2.31E+00 | 2.82E-02 |
| NM_001162375 | Fam73a | 1.88E-01 | 1.89E+00 | 2.31E+00 | 4.01E-02 |
| NM_007400 | Adam12 | 5.09E-02 | 5.02E-01 | 2.29E+00 | 2.97E-02 |
| NM_008415 | Jrk | 7.14E-02 | 6.82E-01 | 2.26E+00 | 3.22E-02 |
| NM_028106 | Zbed3 | 2.42E-01 | 2.31E+00 | 2.26E+00 | 2.66E-02 |
| NM_028390 | Anln | 7.29E-02 | 6.87E-01 | 2.24E+00 | 3.32E-02 |
| NM_053188 | Srd5a2 | 5.85E-01 | 5.51E+00 | 2.24E+00 | 3.32E-02 |
| NM_001164560 | Trmt1 | 2.77E-01 | 2.53E+00 | 2.22E+00 | 1.51E-02 |
| NM_013882 | Gtse1 | 7.58E-02 | 6.94E-01 | 2.21E+00 | 3.84E-02 |
| NM_015821 | Fbxl8 | 2.24E-01 | 1.97E+00 | 2.17E+00 | 3.93E-02 |
| NM_001199296 | Acly | 1.43E+01 | 1.23E+02 | 2.15E+00 | 2.47E-02 |
| NM_018882 | Gpr56 | 5.06E-01 | 4.35E+00 | 2.15E+00 | 2.55E-02 |
| NM_178411 | Zfp334 | 1.57E-01 | 1.35E+00 | 2.15E+00 | 5.12E-03 |
| NM_001024136 | Anks6 | 1.14E-01 | 9.73E-01 | 2.15E+00 | 4.17E-02 |
| NM_145986 | Fam83f | 2.73E-01 | 2.34E+00 | 2.15E+00 | 5.12E-03 |
| NM_172676 | Samd10 | 2.01E-01 | 1.72E+00 | 2.15E+00 | 4.17E-02 |
| NM_028022 | Gatsl3 | 5.66E-01 | 4.79E+00 | 2.14E+00 | 5.40E-03 |
| NM_010762 | Mal | 7.62E-02 | 6.45E-01 | 2.14E+00 | 4.59E-02 |
| NM_001162974 | Lrrc51 | 6.63E-02 | 5.55E-01 | 2.13E+00 | 1.64E-02 |
| NM_013552 | Hmmr | 1.02E-01 | 8.57E-01 | 2.12E+00 | 4.43E-02 |
| NM_011441 | Sox17 | 1.30E-01 | 1.09E+00 | 2.12E+00 | 4.43E-02 |
| NM_017379 | Tuba8 | 3.09E-01 | 2.59E+00 | 2.12E+00 | 4.43E-02 |
| NM_173784 | Ubtd2 | 1.34E-01 | 1.09E+00 | 2.10E+00 | 4.70E-02 |
| NM_177618 | Wscd1 | 1.50E-01 | 1.22E+00 | 2.10E+00 | 4.70E-02 |
| NM_026345 | Mansc1 | 3.50E-01 | 2.85E+00 | 2.10E+00 | 6.35E-03 |
| NR_033525 | Wiz | 1.65E-01 | 1.30E+00 | 2.06E+00 | 1.07E-02 |
| NM_178751 | Orai2 | 2.09E-01 | 1.60E+00 | 2.04E+00 | 8.14E-03 |
| NM_001039537 | Lif | 2.51E-01 | 1.81E+00 | 1.98E+00 | 6.21E-03 |
| NM_001141950 | Tmem80 | 3.42E-01 | 2.46E+00 | 1.98E+00 | 2.76E-02 |
| NM_175684 | Fchsd1 | 1.26E-01 | 9.05E-01 | 1.97E+00 | 1.15E-02 |
| NM_007739 | Col8a1 | 1.55E-01 | 1.10E+00 | 1.96E+00 | 1.12E-02 |
| NM_029658 | Fam101b | 2.30E-01 | 1.63E+00 | 1.96E+00 | 1.12E-02 |
| NM_001177600 | Adam23 | 3.22E-01 | 2.25E+00 | 1.94E+00 | 7.78E-03 |
| NM_001081322 | Myo5c | 1.16E-01 | 8.11E-01 | 1.94E+00 | 1.19E-02 |
| NM_053261 | Impa2 | 6.43E-01 | 4.48E+00 | 1.94E+00 | 1.19E-02 |
| NM_029782 | Calr3 | 2.29E-01 | 1.57E+00 | 1.93E+00 | 1.61E-02 |
| NM_008481 | Lama2 | 8.00E-02 | 5.48E-01 | 1.93E+00 | 1.27E-02 |
| NM_027309 | Lysmd2 | 7.63E-01 | 5.23E+00 | 1.93E+00 | 1.27E-02 |
| NM_177592 | Tmem164 | 3.70E-01 | 2.52E+00 | 1.92E+00 | 4.09E-02 |
| NM_007735 | Col4a4 | 1.00E-01 | 6.75E-01 | 1.91E+00 | 1.34E-02 |
| NM_001159361 | Dip2b | 3.43E-01 | 2.31E+00 | 1.91E+00 | 2.05E-02 |
| NM_001159557 | Cd36 | 3.14E-01 | 2.10E+00 | 1.90E+00 | 5.99E-03 |
| NM_001160209 | Blzf1 | 2.18E-01 | 1.46E+00 | 1.90E+00 | 1.05E-02 |
| NM_007616 | Cav1 | 8.83E-01 | 5.89E+00 | 1.90E+00 | 4.12E-02 |
| NM_023132 | Renbp | 3.19E-01 | 2.12E+00 | 1.89E+00 | 1.64E-02 |
| NM_001001185 | BC048507 | 2.02E+00 | 1.33E+01 | 1.89E+00 | 1.46E-02 |
| NM_011567 | Tead4 | 1.35E-01 | 8.90E-01 | 1.88E+00 | 1.70E-02 |
| NM_009345 | Dntt | 3.00E-01 | 1.96E+00 | 1.88E+00 | 4.75E-03 |
| NM_007963 | Mecom | 1.80E-01 | 1.14E+00 | 1.84E+00 | 1.72E-02 |
| NM_009737 | Bcat2 | 7.43E-01 | 4.70E+00 | 1.84E+00 | 6.67E-03 |
| NM_007763 | Crip1 | 4.69E+00 | 2.95E+01 | 1.84E+00 | 4.53E-03 |
| NM_173189 | Mcph1 | 1.92E-01 | 1.20E+00 | 1.84E+00 | 1.27E-02 |
| NM_001102607 | Col6a6 | 1.92E-01 | 1.20E+00 | 1.84E+00 | 5.27E-03 |
| NM_178695 | Prrg4 | 4.32E-01 | 2.62E+00 | 1.80E+00 | 5.39E-03 |
| NM_001163030 | Cd97 | 5.25E-01 | 3.18E+00 | 1.80E+00 | 4.72E-03 |
| NM_177545 | Vangl1 | 6.70E-01 | 4.04E+00 | 1.80E+00 | 4.35E-02 |
| NM_028027 | Arhgef25 | 1.87E-01 | 1.13E+00 | 1.79E+00 | 2.39E-02 |
| NM_001166413 | Arhgef25 | 1.88E-01 | 1.13E+00 | 1.79E+00 | 2.40E-02 |
| NM_007692 | Chkb | 5.87E-01 | 3.50E+00 | 1.79E+00 | 1.35E-02 |
| NM_025958 | Cand2 | 1.22E-01 | 7.26E-01 | 1.78E+00 | 3.33E-02 |
| NM_175478 | Lrfn3 | 4.29E-01 | 2.55E+00 | 1.78E+00 | 6.10E-03 |
| NM_001024922 | Ddx49 | 6.37E-01 | 3.76E+00 | 1.77E+00 | 8.26E-03 |
| NM_017467 | Zfp316 | 1.14E-01 | 6.70E-01 | 1.77E+00 | 2.22E-02 |
| NM_001164056 | Pld1 | 8.40E-01 | 4.86E+00 | 1.75E+00 | 2.83E-02 |
| NM_008716 | Notch3 | 1.47E-01 | 8.42E-01 | 1.74E+00 | 7.32E-03 |
| NM_001163270 | Baiap3 | 9.83E-03 | 5.53E-02 | 1.73E+00 | 8.72E-03 |
| NM_001190870 | Kcne3 | 1.79E-01 | 9.99E-01 | 1.72E+00 | 3.59E-02 |
| NM_028176 | Cda | 2.51E+00 | 1.40E+01 | 1.72E+00 | 7.28E-03 |
| NM_029420 | Slx1b | 3.49E-01 | 1.93E+00 | 1.71E+00 | 1.08E-02 |
| NM_011979 | Vnn3 | 2.98E+01 | 1.64E+02 | 1.71E+00 | 4.87E-03 |
| NM_028833 | Iqce | 2.74E-01 | 1.51E+00 | 1.71E+00 | 8.81E-03 |
| NM_007736 | Col4a5 | 4.82E-02 | 2.65E-01 | 1.71E+00 | 3.41E-02 |
| NM_010000 | Cyp2b9 | 3.24E+01 | 1.74E+02 | 1.68E+00 | 1.25E-02 |
| NM_028902 | Hsf2bp | 4.28E-01 | 2.29E+00 | 1.68E+00 | 9.96E-03 |
| NM_020032 | Poll | 7.13E-01 | 3.82E+00 | 1.68E+00 | 8.80E-03 |
| NM_033475 | Rab34 | 4.96E-01 | 2.65E+00 | 1.67E+00 | 1.14E-02 |
| NM_001080949 | Ttc5 | 8.65E-01 | 4.59E+00 | 1.67E+00 | 1.05E-02 |
| NM_001163531 | Tmem175 | 5.94E-01 | 3.15E+00 | 1.67E+00 | 7.37E-03 |
| NM_025609 | Tab1 | 4.18E-01 | 2.21E+00 | 1.67E+00 | 1.06E-02 |
| NM_023824 | Paqr4 | 4.87E-01 | 2.56E+00 | 1.66E+00 | 1.62E-02 |
| NM_012044 | Pla2g2e | 1.17E+00 | 6.17E+00 | 1.66E+00 | 3.31E-02 |
| NM_172151 | Zdhhc8 | 2.45E-01 | 1.28E+00 | 1.65E+00 | 1.13E-02 |
| NM_027341 | Dzip3 | 7.99E-02 | 4.16E-01 | 1.65E+00 | 1.58E-02 |
| NM_001199927 | Fam198a | 5.08E-01 | 2.63E+00 | 1.65E+00 | 1.24E-02 |
| NM_001134383 | Iqsec1 | 3.69E-01 | 1.89E+00 | 1.63E+00 | 9.52E-03 |
| NM_183221 | Fat4 | 1.20E-01 | 6.10E-01 | 1.63E+00 | 6.98E-03 |
| NM_001033208 | Gcom1 | 7.31E-01 | 3.72E+00 | 1.63E+00 | 1.12E-02 |
| NM_001081367 | Kctd17 | 1.11E+00 | 5.65E+00 | 1.63E+00 | 1.12E-02 |
| NM_013641 | Ptger1 | 4.99E-01 | 2.52E+00 | 1.62E+00 | 2.37E-02 |
| NM_027828 | Fam110c | 6.17E-01 | 3.11E+00 | 1.62E+00 | 1.18E-02 |
| NM_021469 | Dysf | 2.03E-01 | 1.02E+00 | 1.62E+00 | 1.46E-02 |
| NM_001168526 | Sgms1 | 6.40E-01 | 3.20E+00 | 1.61E+00 | 7.28E-03 |
| NM_001167763 | Ift122 | 1.97E-01 | 9.80E-01 | 1.61E+00 | 7.28E-03 |
| NM_031177 | Ift122 | 1.97E-01 | 9.82E-01 | 1.61E+00 | 7.29E-03 |
| NM_134164 | Syt12 | 6.18E-01 | 3.08E+00 | 1.60E+00 | 7.94E-03 |
| NM_009616 | Adam19 | 1.84E-01 | 9.09E-01 | 1.60E+00 | 1.47E-02 |
| NM_001039878 | Strn4 | 6.22E-01 | 3.00E+00 | 1.57E+00 | 7.93E-03 |
| NM_053271 | Rims2 | 1.65E-01 | 7.98E-01 | 1.57E+00 | 4.38E-02 |
| NM_027985 | Mad2l2 | 2.24E+00 | 1.08E+01 | 1.57E+00 | 6.40E-03 |
| NM_054099 | 1110038F14Rik | 8.57E-01 | 4.13E+00 | 1.57E+00 | 4.38E-02 |
| NR_037773 | Tmem41a | 2.12E+00 | 1.02E+01 | 1.57E+00 | 7.78E-03 |
| NM_019703 | Pfkp | 5.83E-01 | 2.80E+00 | 1.57E+00 | 6.97E-03 |
| NM_138580 | Rdbp | 2.23E+00 | 1.07E+01 | 1.57E+00 | 4.87E-03 |
| NM_011349 | Sema3f | 8.34E-01 | 3.96E+00 | 1.56E+00 | 5.23E-03 |
| NM_027840 | Snx20 | 2.16E+00 | 1.03E+01 | 1.56E+00 | 5.23E-03 |
| NM_011925 | Cd97 | 6.62E-01 | 3.14E+00 | 1.56E+00 | 8.94E-03 |
| NM_001161775 | Myh11 | 2.37E-01 | 1.12E+00 | 1.55E+00 | 1.59E-02 |
| NM_007658 | Cdc25a | 4.44E-01 | 2.09E+00 | 1.55E+00 | 1.59E-02 |
| NM_173013 | Mtap1s | 3.71E-01 | 1.75E+00 | 1.55E+00 | 1.78E-02 |
| NM_009995 | Cyp21a1 | 7.74E-01 | 3.65E+00 | 1.55E+00 | 1.14E-02 |
| NM_028721 | Nphp3 | 1.90E-01 | 8.96E-01 | 1.55E+00 | 2.56E-02 |
| NM_001164195 | Pex3 | 1.85E+00 | 8.61E+00 | 1.54E+00 | 8.38E-03 |
| NM_030143 | Ddit4l | 6.04E-01 | 2.82E+00 | 1.54E+00 | 1.67E-02 |
| NM_207237 | Man1c1 | 2.53E-01 | 1.17E+00 | 1.54E+00 | 1.90E-02 |
| NM_026651 | Pomgnt1 | 2.08E+00 | 9.65E+00 | 1.53E+00 | 4.38E-02 |
| NM_010589 | Jak3 | 4.20E-01 | 1.94E+00 | 1.53E+00 | 1.58E-02 |
| NM_007554 | Bmp4 | 6.37E-01 | 2.93E+00 | 1.53E+00 | 1.99E-02 |
| NM_175638 | Wnk4 | 6.75E-01 | 3.10E+00 | 1.52E+00 | 6.35E-03 |
| NM_177588 | Thnsl1 | 2.94E-01 | 1.35E+00 | 1.52E+00 | 2.85E-02 |
| NM_153540 | C85492 | 5.27E-01 | 2.41E+00 | 1.52E+00 | 2.05E-02 |
| NM_011633 | Traf5 | 6.11E-01 | 2.79E+00 | 1.52E+00 | 2.05E-02 |
| NM_007730 | Col12a1 | 1.33E-01 | 6.05E-01 | 1.52E+00 | 1.85E-02 |
| NM_018763 | Chst2 | 2.52E-01 | 1.15E+00 | 1.52E+00 | 1.85E-02 |
| NM_001161433 | Eda2r | 5.16E-01 | 2.35E+00 | 1.52E+00 | 3.05E-02 |
| NM_009229 | Sntb2 | 8.11E-01 | 3.63E+00 | 1.50E+00 | 5.05E-03 |
| NM_011623 | Top2a | 4.55E-01 | 2.02E+00 | 1.49E+00 | 1.00E-02 |
| NM_022415 | Ptges | 3.31E-01 | 1.47E+00 | 1.49E+00 | 2.33E-02 |
| NM_026453 | Mak16 | 8.25E-01 | 3.63E+00 | 1.48E+00 | 2.40E-02 |
| NM_001136181 | Hsbp1l1 | 1.09E+00 | 4.74E+00 | 1.47E+00 | 2.49E-02 |
| NM_172574 | Pqlc3 | 1.08E+00 | 4.70E+00 | 1.47E+00 | 1.53E-02 |
| NM_198012 | Trim68 | 6.96E-01 | 3.02E+00 | 1.47E+00 | 2.29E-02 |
| NM_028112 | Seh1l | 5.84E-01 | 2.52E+00 | 1.46E+00 | 4.40E-02 |
| NM_001029936 | Specc1 | 2.94E-01 | 1.26E+00 | 1.46E+00 | 1.64E-02 |
| NM_019810 | Slc5a1 | 3.06E-01 | 1.31E+00 | 1.46E+00 | 2.68E-02 |
| NM_173429 | Zfp775 | 2.94E-01 | 1.26E+00 | 1.46E+00 | 2.68E-02 |
| NM_001177886 | Igsf5 | 1.72E+00 | 7.33E+00 | 1.45E+00 | 1.29E-02 |
| NM_177077 | Exoc6b | 7.99E-01 | 3.41E+00 | 1.45E+00 | 4.88E-03 |
| NM_011711 | Fmnl3 | 9.04E-01 | 3.86E+00 | 1.45E+00 | 5.55E-03 |
| NM_199009 | Fam160a2 | 8.26E-01 | 3.51E+00 | 1.45E+00 | 9.46E-03 |
| NM_010515 | Igf2r | 4.57E+00 | 1.94E+01 | 1.45E+00 | 5.24E-03 |
| NM_029100 | Sepn1 | 5.86E-01 | 2.49E+00 | 1.45E+00 | 1.72E-02 |
| NM_026179 | Abhd5 | 1.51E+01 | 6.40E+01 | 1.44E+00 | 5.14E-03 |
| NM_001038602 | Marveld2 | 1.49E+00 | 6.28E+00 | 1.44E+00 | 4.96E-03 |
| NM_028787 | Slc35f5 | 1.92E+01 | 8.13E+01 | 1.44E+00 | 6.82E-03 |
| NM_053254 | Tle6 | 6.30E-01 | 2.65E+00 | 1.44E+00 | 2.86E-02 |
| NM_028057 | Cyb5r1 | 2.14E+00 | 8.99E+00 | 1.44E+00 | 8.12E-03 |
| NM_001081419 | Dip2a | 3.09E-01 | 1.30E+00 | 1.44E+00 | 1.80E-02 |
| NM_175097 | Prickle3 | 2.34E-01 | 9.84E-01 | 1.43E+00 | 1.61E-02 |
| NM_020011 | Sphk2 | 7.86E-01 | 3.29E+00 | 1.43E+00 | 9.62E-03 |
| NM_013832 | Rasal1 | 7.59E-01 | 3.17E+00 | 1.43E+00 | 1.35E-02 |
| NM_011057 | Pdgfb | 5.55E-01 | 2.32E+00 | 1.43E+00 | 2.68E-02 |
| NM_019482 | Panx1 | 8.13E-01 | 3.40E+00 | 1.43E+00 | 2.68E-02 |
| NM_001170981 | Hnrnpc | 4.50E-01 | 1.88E+00 | 1.43E+00 | 3.80E-02 |
| NM_025294 | Gm16515 | 1.15E+00 | 4.80E+00 | 1.43E+00 | 5.00E-03 |
| NM_176996 | Smo | 1.21E+00 | 5.03E+00 | 1.43E+00 | 5.00E-03 |
| NM_027086 | Ubl7 | 3.66E+00 | 1.52E+01 | 1.42E+00 | 5.88E-03 |
| NM_175534 | Mrgpre | 3.21E-01 | 1.33E+00 | 1.42E+00 | 3.07E-02 |
| NM_019783 | Lepre1 | 9.67E-01 | 4.01E+00 | 1.42E+00 | 9.86E-03 |
| NM_023500 | Xk | 2.61E-01 | 1.08E+00 | 1.42E+00 | 3.43E-02 |
| NM_007737 | Col5a2 | 5.93E-01 | 2.44E+00 | 1.41E+00 | 6.76E-03 |
| NM_001099634 | Myof | 6.62E-01 | 2.71E+00 | 1.41E+00 | 5.55E-03 |
| NM_001083967 | Tcf4 | 7.81E-01 | 3.19E+00 | 1.41E+00 | 4.55E-03 |
| NM_009691 | Aplp2 | 1.54E+01 | 6.28E+01 | 1.41E+00 | 9.30E-03 |
| NM_001159883 | Dnajb2 | 1.47E+00 | 6.00E+00 | 1.40E+00 | 6.28E-03 |
| NM_172289 | Slc36a4 | 8.77E-01 | 3.57E+00 | 1.40E+00 | 2.98E-02 |
| NM_175930 | Rapgef5 | 1.31E+00 | 5.30E+00 | 1.40E+00 | 5.30E-03 |
| NM_153068 | Ehd2 | 1.20E+00 | 4.85E+00 | 1.40E+00 | 8.88E-03 |
| NM_009739 | Bckdk | 1.00E+00 | 4.05E+00 | 1.40E+00 | 1.25E-02 |
| NM_025968 | Ptgr1 | 1.20E+00 | 4.85E+00 | 1.40E+00 | 1.60E-02 |
| NM_001080968 | Golga2 | 3.35E+00 | 1.35E+01 | 1.39E+00 | 4.09E-02 |
| NM_001159633 | Slc44a1 | 1.31E+00 | 5.28E+00 | 1.39E+00 | 1.63E-02 |
| NM_172775 | Plxnb1 | 4.21E+00 | 1.70E+01 | 1.39E+00 | 4.55E-03 |
| NM_027891 | Lrwd1 | 9.62E-01 | 3.88E+00 | 1.39E+00 | 2.19E-02 |
| NM_015759 | Fgd3 | 3.92E-01 | 1.57E+00 | 1.39E+00 | 3.53E-02 |
| NM_177673 | BC046331 | 6.18E-01 | 2.47E+00 | 1.38E+00 | 2.29E-02 |
| NM_011829 | Impdh1 | 8.62E-01 | 3.44E+00 | 1.38E+00 | 2.29E-02 |
| NM_001177631 | Etl4 | 4.47E-01 | 1.77E+00 | 1.38E+00 | 1.06E-02 |
| NM_184053 | Calu | 1.39E+01 | 5.53E+01 | 1.38E+00 | 7.41E-03 |
| NM_008681 | Ndrg1 | 1.70E+00 | 6.73E+00 | 1.38E+00 | 6.66E-03 |
| NM_010097 | Sparcl1 | 7.83E-01 | 3.11E+00 | 1.38E+00 | 1.75E-02 |
| NM_173019 | Pfkfb4 | 4.82E-01 | 1.91E+00 | 1.38E+00 | 3.33E-02 |
| NR_029464 | 5033406O09Rik | 1.50E+00 | 5.93E+00 | 1.38E+00 | 1.75E-02 |
| NM_183086 | Mrps10 | 1.22E-01 | 4.81E-01 | 1.37E+00 | 1.20E-02 |
| NM_001168253 | Fam83h | 6.17E-01 | 2.44E+00 | 1.37E+00 | 1.38E-02 |
| NM_009655 | Alcam | 1.57E+01 | 6.22E+01 | 1.37E+00 | 4.23E-02 |
| NM_001146275 | Iigp1 | 2.12E+01 | 8.36E+01 | 1.37E+00 | 1.40E-02 |
| NM_175270 | Ankrd56 | 1.13E+00 | 4.45E+00 | 1.37E+00 | 7.75E-03 |
| NM_177364 | Sh3pxd2b | 8.94E-01 | 3.52E+00 | 1.37E+00 | 4.58E-03 |
| NM_011697 | Vegfb | 1.34E+00 | 5.27E+00 | 1.37E+00 | 3.78E-02 |
| NM_015763 | Lpin1 | 1.15E+01 | 4.46E+01 | 1.36E+00 | 1.57E-02 |
| NM_026400 | Dnajb11 | 5.71E-01 | 2.22E+00 | 1.36E+00 | 4.17E-02 |
| NM_011647 | Tsc2 | 6.35E-01 | 2.46E+00 | 1.35E+00 | 9.66E-03 |
| NM_023716 | Tubb2b | 2.00E+01 | 7.73E+01 | 1.35E+00 | 5.85E-03 |
| NM_001135611 | 3110062M04Rik | 4.95E-01 | 1.91E+00 | 1.35E+00 | 3.36E-02 |
| NM_028064 | Slc39a4 | 2.20E+01 | 8.50E+01 | 1.35E+00 | 8.46E-03 |
| NM_174850 | Micall2 | 4.84E-01 | 1.87E+00 | 1.35E+00 | 3.70E-02 |
| NR_027905 | LOC106740 | 9.07E-02 | 3.48E-01 | 1.35E+00 | 2.19E-02 |
| NM_133816 | Sh3bp4 | 8.77E-01 | 3.36E+00 | 1.34E+00 | 1.36E-02 |
| NM_001003955 | Rab11fip5 | 1.27E-01 | 4.86E-01 | 1.34E+00 | 4.65E-02 |
| NM_019424 | Hps1 | 7.45E-01 | 2.84E+00 | 1.34E+00 | 2.79E-02 |
| NM_172457 | Mobkl2a | 1.56E+00 | 5.94E+00 | 1.34E+00 | 1.04E-02 |
| NM_145933 | St6gal1 | 1.57E+01 | 6.00E+01 | 1.34E+00 | 2.24E-02 |
| NM_175111 | Hspbap1 | 3.45E-01 | 1.31E+00 | 1.34E+00 | 3.93E-02 |
| NM_027590 | Ints10 | 1.21E+00 | 4.58E+00 | 1.33E+00 | 1.72E-02 |
| NM_133715 | Arhgap27 | 4.96E-01 | 1.88E+00 | 1.33E+00 | 3.39E-02 |
| NM_011197 | Ptgfrn | 1.43E+00 | 5.39E+00 | 1.33E+00 | 4.87E-03 |
| NM_001142781 | Fam188b | 2.06E-01 | 7.71E-01 | 1.32E+00 | 2.85E-02 |
| NM_001042727 | Rarg | 1.76E+00 | 6.57E+00 | 1.32E+00 | 1.02E-02 |
| NM_001167918 | D830031N03Rik | 3.78E-01 | 1.41E+00 | 1.32E+00 | 1.51E-02 |
| NM_175451 | Ckap4 | 7.01E-01 | 2.61E+00 | 1.32E+00 | 3.08E-02 |
| NM_023735 | Actr3 | 1.44E+00 | 5.36E+00 | 1.32E+00 | 1.28E-02 |
| NM_008046 | Fst | 3.92E+00 | 1.46E+01 | 1.31E+00 | 5.04E-03 |
| NM_009154 | Sema5a | 1.08E-01 | 4.00E-01 | 1.31E+00 | 4.69E-02 |
| NM_172503 | Zswim4 | 8.26E-01 | 3.07E+00 | 1.31E+00 | 1.41E-02 |
| NM_170588 | Cpne1 | 1.62E+00 | 6.00E+00 | 1.31E+00 | 8.20E-03 |
| NM_172785 | Zc3h12d | 6.84E-01 | 2.54E+00 | 1.31E+00 | 1.95E-02 |
| NM_019738 | Nupr1 | 3.60E+00 | 1.33E+01 | 1.31E+00 | 4.40E-02 |
| NM_001166427 | Hnrnpf | 1.15E+00 | 4.24E+00 | 1.30E+00 | 2.23E-02 |
| NM_130859 | Card10 | 1.43E+00 | 5.26E+00 | 1.30E+00 | 6.90E-03 |
| NM_013659 | Sema4b | 7.11E-01 | 2.61E+00 | 1.30E+00 | 2.03E-02 |
| NM_009236 | Sox18 | 1.88E+00 | 6.91E+00 | 1.30E+00 | 2.03E-02 |
| NM_013719 | Eif2ak4 | 7.49E-01 | 2.75E+00 | 1.30E+00 | 1.33E-02 |
| NM_021539 | Wsb2 | 1.62E+01 | 5.94E+01 | 1.30E+00 | 6.71E-03 |
| NM_001135657 | Ptprj | 6.70E-01 | 2.45E+00 | 1.29E+00 | 2.08E-02 |
| NM_178653 | Sccpdh | 1.99E+00 | 7.26E+00 | 1.29E+00 | 1.58E-02 |
| NM_144936 | Tmem45b | 1.11E+00 | 4.04E+00 | 1.29E+00 | 4.65E-02 |
| NM_019654 | Socs5 | 1.36E+00 | 4.94E+00 | 1.29E+00 | 8.63E-03 |
| NM_012048 | Polk | 1.32E+00 | 4.80E+00 | 1.29E+00 | 9.46E-03 |
| NM_178389 | Gale | 3.42E+00 | 1.24E+01 | 1.29E+00 | 1.12E-02 |
| NM_145830 | Ehmt2 | 2.35E+00 | 8.50E+00 | 1.29E+00 | 5.72E-03 |
| NM_001164679 | Ano8 | 5.14E-01 | 1.86E+00 | 1.28E+00 | 3.57E-02 |
| NM_177715 | Kctd12 | 6.56E+00 | 2.37E+01 | 1.28E+00 | 8.27E-03 |
| NM_009014 | Rad51l1 | 2.07E+00 | 7.44E+00 | 1.28E+00 | 1.69E-02 |
| NM_011019 | Osmr | 1.40E+00 | 5.01E+00 | 1.28E+00 | 7.99E-03 |
| NM_010127 | Pou6f1 | 3.48E-01 | 1.25E+00 | 1.28E+00 | 4.91E-02 |
| NM_181541 | Caprin2 | 4.52E-01 | 1.62E+00 | 1.28E+00 | 4.91E-02 |
| NM_020486 | Bcam | 1.38E+00 | 4.96E+00 | 1.28E+00 | 1.89E-02 |
| NM_133904 | Acacb | 5.65E+00 | 2.03E+01 | 1.28E+00 | 1.27E-02 |
| NM_017376 | Tef | 3.82E+00 | 1.36E+01 | 1.27E+00 | 4.64E-03 |
| NM_017402 | Arhgef7 | 1.61E+00 | 5.75E+00 | 1.27E+00 | 8.01E-03 |
| NM_001042591 | Arrdc3 | 9.98E+00 | 3.56E+01 | 1.27E+00 | 9.02E-03 |
| NM_027870 | Armcx3 | 2.99E+00 | 1.06E+01 | 1.27E+00 | 6.00E-03 |
| NM_001039170 | Eif4e2 | 2.64E+00 | 9.34E+00 | 1.27E+00 | 1.37E-02 |
| NM_144832 | BC017643 | 1.43E+00 | 5.08E+00 | 1.26E+00 | 1.96E-02 |
| NM_007856 | Dhcr7 | 2.68E+01 | 9.47E+01 | 1.26E+00 | 2.58E-02 |
| NM_011175 | Lgmn | 3.21E+01 | 1.13E+02 | 1.26E+00 | 1.43E-02 |
| NM_011027 | P2rx7 | 5.40E-01 | 1.90E+00 | 1.26E+00 | 2.71E-02 |
| NR_033514 | 2310015A10Rik | 4.10E-01 | 1.44E+00 | 1.26E+00 | 3.97E-02 |
| NM_207649 | Rcan2 | 1.62E+00 | 5.70E+00 | 1.26E+00 | 1.21E-02 |
| NM_198683 | Celf1 | 5.74E+00 | 2.02E+01 | 1.26E+00 | 6.04E-03 |
| NM_001081454 | Furin | 1.81E+01 | 6.34E+01 | 1.25E+00 | 3.18E-02 |
| NM_201226 | Lrrc47 | 1.28E+00 | 4.49E+00 | 1.25E+00 | 1.50E-02 |
| NM_145439 | Tmc6 | 2.19E+00 | 7.68E+00 | 1.25E+00 | 1.04E-02 |
| NM_001085390 | Dusp5 | 3.60E+00 | 1.26E+01 | 1.25E+00 | 1.92E-02 |
| NM_145520 | Trub2 | 8.11E-01 | 2.83E+00 | 1.25E+00 | 3.95E-02 |
| NM_001077410 | Gimap8 | 7.73E-01 | 2.70E+00 | 1.25E+00 | 2.57E-02 |
| NM_001122830 | Klhl26 | 6.28E-01 | 2.19E+00 | 1.25E+00 | 4.69E-02 |
| NM_009931 | Col4a1 | 9.25E+00 | 3.22E+01 | 1.25E+00 | 2.08E-02 |
| NM_134189 | Galnt10 | 1.52E+00 | 5.29E+00 | 1.25E+00 | 9.18E-03 |
| NM_001110309 | Zfp426 | 7.34E-01 | 2.55E+00 | 1.25E+00 | 3.06E-02 |
| NM_001204979 | Sars | 5.82E+00 | 2.02E+01 | 1.25E+00 | 5.58E-03 |
| NM_145390 | Tnpo2 | 2.89E+00 | 1.00E+01 | 1.24E+00 | 6.08E-03 |
| NM_001113373 | Shank2 | 5.34E-01 | 1.85E+00 | 1.24E+00 | 2.07E-02 |
| NM_009842 | Cd151 | 4.85E+00 | 1.68E+01 | 1.24E+00 | 8.05E-03 |
| NM_153565 | Pcsk9 | 1.59E+01 | 5.49E+01 | 1.24E+00 | 1.51E-02 |
| NM_029777 | Rhbdd1 | 3.05E+00 | 1.06E+01 | 1.24E+00 | 6.97E-03 |
| NM_031397 | Bicc1 | 9.20E-01 | 3.18E+00 | 1.24E+00 | 2.69E-02 |
| NM_133626 | Rrbp1 | 8.53E+00 | 2.95E+01 | 1.24E+00 | 6.12E-03 |
| NM_177910 | Gmppb | 3.48E+00 | 1.20E+01 | 1.24E+00 | 1.22E-02 |
| NM_148929 | Slc9a8 | 2.18E+00 | 7.53E+00 | 1.24E+00 | 7.50E-03 |
| NM_001113554 | Nudcd1 | 1.78E+00 | 6.15E+00 | 1.24E+00 | 1.13E-02 |
| NM_009928 | Col15a1 | 8.17E-01 | 2.82E+00 | 1.24E+00 | 1.62E-02 |
| NM_023516 | 2310016C08Rik | 2.57E+00 | 8.84E+00 | 1.24E+00 | 4.07E-02 |
| NM_001171034 | Tmbim6 | 3.54E+00 | 1.22E+01 | 1.23E+00 | 8.15E-03 |
| NM_023130 | Raly | 2.19E+00 | 7.49E+00 | 1.23E+00 | 2.12E-02 |
| NM_016886 | Gria3 | 1.30E+00 | 4.43E+00 | 1.23E+00 | 1.08E-02 |
| NM_009386 | Tjp1 | 5.50E+00 | 1.88E+01 | 1.23E+00 | 8.98E-03 |
| NM_011656 | Tuft1 | 2.09E+00 | 7.15E+00 | 1.23E+00 | 1.46E-02 |
| NM_001163029 | Cd97 | 4.73E+00 | 1.61E+01 | 1.23E+00 | 7.10E-03 |
| NM_009395 | Tnfaip1 | 2.86E+00 | 9.76E+00 | 1.23E+00 | 7.60E-03 |
| NM_144808 | Slc39a14 | 2.00E+00 | 6.81E+00 | 1.22E+00 | 8.03E-03 |
| NM_008425 | Kcnj2 | 5.07E-01 | 1.72E+00 | 1.22E+00 | 2.95E-02 |
| NM_001111049 | Cd151 | 1.20E+01 | 4.09E+01 | 1.22E+00 | 6.33E-03 |
| NM_172743 | Plekha7 | 9.25E-01 | 3.13E+00 | 1.22E+00 | 1.99E-02 |
| NR_040429 | Gm15408 | 1.62E+00 | 5.48E+00 | 1.22E+00 | 4.62E-02 |
| NM_026436 | Tmem86a | 9.82E+00 | 3.32E+01 | 1.22E+00 | 6.67E-03 |
| NM_009195 | Slc12a4 | 3.54E+00 | 1.20E+01 | 1.22E+00 | 7.20E-03 |
| NM_007809 | Cyp17a1 | 2.21E+01 | 7.43E+01 | 1.21E+00 | 9.10E-03 |
| NM_022417 | Itm2c | 1.55E+01 | 5.20E+01 | 1.21E+00 | 7.12E-03 |
| NM_001162943 | Dchs1 | 3.64E-01 | 1.22E+00 | 1.21E+00 | 2.05E-02 |
| NM_008528 | Blnk | 2.01E+00 | 6.76E+00 | 1.21E+00 | 2.05E-02 |
| NM_010421 | Hexa | 1.95E+01 | 6.54E+01 | 1.21E+00 | 8.99E-03 |
| NM_001018063 | Cxx1b | 2.46E+00 | 8.28E+00 | 1.21E+00 | 3.34E-02 |
| NM_008428 | Kcnj8 | 4.60E+00 | 1.54E+01 | 1.21E+00 | 9.04E-03 |
| NM_001077695 | Ncoa2 | 1.05E+00 | 3.53E+00 | 1.21E+00 | 9.91E-03 |
| NR_003964 | Tubb2a-ps2 | 6.84E+00 | 2.30E+01 | 1.21E+00 | 2.38E-02 |
| NM_010148 | Epn2 | 1.23E+00 | 4.13E+00 | 1.21E+00 | 1.62E-02 |
| NM_016846 | Rgl1 | 3.10E+00 | 1.04E+01 | 1.21E+00 | 8.01E-03 |
| NM_001164062 | Stat5a | 8.96E-01 | 2.99E+00 | 1.21E+00 | 2.71E-02 |
| NM_001039059 | Klhl15 | 1.24E-01 | 4.14E-01 | 1.20E+00 | 4.67E-02 |
| NM_001165925 | Cldn14 | 3.21E+00 | 1.07E+01 | 1.20E+00 | 2.49E-02 |
| NM_145220 | Appl2 | 4.85E+00 | 1.62E+01 | 1.20E+00 | 8.01E-03 |
| NM_011249 | Rbl1 | 4.24E-01 | 1.41E+00 | 1.20E+00 | 4.74E-02 |
| NM_001081963 | 9430020K01Rik | 1.63E+00 | 5.43E+00 | 1.20E+00 | 8.81E-03 |
| NM_001168659 | Ccdc127 | 2.35E+00 | 7.82E+00 | 1.20E+00 | 1.09E-02 |
| NM_001085498 | Chmp6 | 5.60E+00 | 1.86E+01 | 1.20E+00 | 1.09E-02 |
| NM_001136078 | Pitpnm1 | 7.51E-01 | 2.49E+00 | 1.20E+00 | 2.96E-02 |
| NM_008695 | Nid2 | 8.63E-01 | 2.86E+00 | 1.20E+00 | 2.05E-02 |
| NM_144918 | Smyd5 | 2.82E+00 | 9.36E+00 | 1.20E+00 | 1.28E-02 |
| NM_007568 | Btc | 2.13E+00 | 7.07E+00 | 1.20E+00 | 1.47E-02 |
| NM_016722 | Galns | 1.54E+00 | 5.09E+00 | 1.20E+00 | 2.36E-02 |
| NM_178607 | Rnf24 | 2.23E+00 | 7.36E+00 | 1.20E+00 | 8.26E-03 |
| NM_174997 | Fam168b | 3.97E+00 | 1.31E+01 | 1.20E+00 | 7.18E-03 |
| NM_001146687 | Pip5k1c | 1.29E+00 | 4.25E+00 | 1.19E+00 | 1.70E-02 |
| NM_016739 | Caprin1 | 3.31E+00 | 1.09E+01 | 1.19E+00 | 7.55E-03 |
| NM_175518 | D730040F13Rik | 2.33E+00 | 7.68E+00 | 1.19E+00 | 7.82E-03 |
| NM_175429 | Kctd12b | 6.62E-01 | 2.18E+00 | 1.19E+00 | 2.89E-02 |
| NM_022993 | Lrp10 | 8.93E+00 | 2.94E+01 | 1.19E+00 | 7.97E-03 |
| NM_011580 | Thbs1 | 1.15E+00 | 3.80E+00 | 1.19E+00 | 1.34E-02 |
| NM_016696 | Gpc1 | 1.93E+00 | 6.35E+00 | 1.19E+00 | 1.34E-02 |
| NM_015774 | Ero1l | 7.13E+00 | 2.34E+01 | 1.19E+00 | 9.91E-03 |
| NM_145539 | Tm4sf4 | 4.11E+01 | 1.35E+02 | 1.19E+00 | 1.81E-02 |
| NM_153533 | Tenc1 | 5.15E+00 | 1.69E+01 | 1.19E+00 | 7.77E-03 |
| NM_021350 | Chml | 4.52E-01 | 1.48E+00 | 1.19E+00 | 3.45E-02 |
| NM_028427 | Tmem192 | 3.74E+00 | 1.22E+01 | 1.19E+00 | 2.65E-02 |
| NM_007988 | Fasn | 7.90E+01 | 2.58E+02 | 1.19E+00 | 5.74E-03 |
| NM_009930 | Col3a1 | 4.26E+00 | 1.39E+01 | 1.18E+00 | 8.32E-03 |
| NM_010687 | Large | 8.78E-01 | 2.87E+00 | 1.18E+00 | 3.00E-02 |
| NM_172650 | Kctd3 | 4.67E+00 | 1.53E+01 | 1.18E+00 | 8.38E-03 |
| NM_008884 | Pml | 5.97E-01 | 1.95E+00 | 1.18E+00 | 3.07E-02 |
| NM_007874 | Reep5 | 4.94E+00 | 1.61E+01 | 1.18E+00 | 9.05E-03 |
| NM_033523 | Spred2 | 2.14E+00 | 6.96E+00 | 1.18E+00 | 1.61E-02 |
| NM_026825 | Lrrc16a | 2.84E+00 | 9.21E+00 | 1.18E+00 | 9.16E-03 |
| NM_008826 | Pfkl | 4.00E+00 | 1.30E+01 | 1.18E+00 | 9.30E-03 |
| NM_001081286 | Fat1 | 4.38E+00 | 1.41E+01 | 1.17E+00 | 2.66E-02 |
| NM_172404 | Ccbl1 | 3.01E+01 | 9.71E+01 | 1.17E+00 | 2.20E-02 |
| NM_001172062 | Lman1 | 7.63E+00 | 2.46E+01 | 1.17E+00 | 9.79E-03 |
| NM_010472 | Agfg1 | 8.04E+00 | 2.59E+01 | 1.17E+00 | 8.70E-03 |
| NM_013855 | Abca3 | 5.19E+00 | 1.67E+01 | 1.17E+00 | 1.20E-02 |
| NM_023142 | Arpc1b | 2.14E+01 | 6.90E+01 | 1.17E+00 | 9.15E-03 |
| NM_001110307 | Keap1 | 4.10E+00 | 1.32E+01 | 1.17E+00 | 9.69E-03 |
| NM_173863 | Crtc3 | 7.95E-01 | 2.56E+00 | 1.17E+00 | 2.50E-02 |
| NM_008983 | Ptprk | 8.59E+00 | 2.76E+01 | 1.17E+00 | 2.25E-02 |
| NM_011101 | Prkca | 1.54E+00 | 4.93E+00 | 1.17E+00 | 9.83E-03 |
| NM_001014390 | Dyrk2 | 5.90E+00 | 1.89E+01 | 1.17E+00 | 1.03E-02 |
| NM_198724 | Egfl7 | 3.75E+00 | 1.20E+01 | 1.17E+00 | 2.24E-02 |
| NM_172261 | Ppp1r9b | 2.21E+00 | 7.07E+00 | 1.16E+00 | 1.20E-02 |
| NM_001080129 | Tmpo | 2.02E+00 | 6.46E+00 | 1.16E+00 | 1.52E-02 |
| NM_177354 | Vash1 | 6.99E-01 | 2.23E+00 | 1.16E+00 | 2.42E-02 |
| NM_144787 | Kdm4c | 8.97E-01 | 2.87E+00 | 1.16E+00 | 2.88E-02 |
| NM_181540 | Tm6sf2 | 1.03E+01 | 3.30E+01 | 1.16E+00 | 1.01E-02 |
| NM_153537 | Phldb1 | 6.51E-01 | 2.08E+00 | 1.16E+00 | 3.04E-02 |
| NM_001081081 | Gls | 4.72E-01 | 1.51E+00 | 1.16E+00 | 4.83E-02 |
| NM_020275 | Tnfrsf10b | 9.58E-01 | 3.05E+00 | 1.16E+00 | 3.69E-02 |
| NM_178732 | Zfp324 | 6.51E-01 | 2.07E+00 | 1.16E+00 | 3.96E-02 |
| NM_013755 | Gyg | 2.77E+00 | 8.79E+00 | 1.16E+00 | 2.50E-02 |
| NM_019924 | Rps6ka4 | 1.96E+00 | 6.23E+00 | 1.15E+00 | 1.87E-02 |
| NM_145384 | Pqlc2 | 4.12E+00 | 1.31E+01 | 1.15E+00 | 1.44E-02 |
| NM_010631 | Kifc3 | 1.22E+00 | 3.88E+00 | 1.15E+00 | 2.83E-02 |
| NM_145970 | Cc2d1a | 1.11E+00 | 3.51E+00 | 1.15E+00 | 3.15E-02 |
| NM_145564 | Fbxo21 | 6.36E+00 | 2.01E+01 | 1.15E+00 | 1.00E-02 |
| NM_001035533 | Akap2 | 1.06E+00 | 3.34E+00 | 1.15E+00 | 1.57E-02 |
| NM_153542 | Lrrc20 | 1.26E+00 | 3.98E+00 | 1.15E+00 | 3.51E-02 |
| NM_008398 | Itga7 | 6.58E-01 | 2.08E+00 | 1.15E+00 | 4.41E-02 |
| NM_145424 | BC089597 | 4.51E+01 | 1.43E+02 | 1.15E+00 | 1.85E-02 |
| NM_007511 | Atp7b | 3.54E+00 | 1.12E+01 | 1.15E+00 | 1.01E-02 |
| NM_139140 | Spats2 | 1.44E+00 | 4.53E+00 | 1.15E+00 | 4.12E-02 |
| NM_001077363 | Ptbp1 | 7.98E+00 | 2.52E+01 | 1.15E+00 | 1.01E-02 |
| NM_001024458 | Add1 | 4.66E+00 | 1.47E+01 | 1.15E+00 | 9.93E-03 |
| NM_030254 | Tusc3 | 5.23E+00 | 1.65E+01 | 1.15E+00 | 1.64E-02 |
| NM_031881 | Nedd4l | 1.70E+00 | 5.37E+00 | 1.15E+00 | 1.09E-02 |
| NM_197999 | Ces2g | 1.62E+01 | 5.09E+01 | 1.15E+00 | 1.83E-02 |
| NM_172884 | 2900026A02Rik | 6.81E+00 | 2.14E+01 | 1.15E+00 | 1.39E-02 |
| NM_033612 | Cela1 | 1.26E+01 | 3.97E+01 | 1.15E+00 | 1.18E-02 |
| NM_010207 | Fgfr2 | 4.67E+00 | 1.47E+01 | 1.14E+00 | 1.06E-02 |
| NM_198616 | Ccdc85b | 9.35E-01 | 2.94E+00 | 1.14E+00 | 2.81E-02 |
| NM_001164217 | Romo1 | 2.02E+01 | 6.33E+01 | 1.14E+00 | 3.08E-02 |
| NM_018771 | Gipc1 | 3.50E+00 | 1.10E+01 | 1.14E+00 | 2.31E-02 |
| NM_001128094 | Atp13a3 | 8.33E+00 | 2.60E+01 | 1.14E+00 | 2.59E-02 |
| NM_001122780 | Klhl25 | 2.67E+00 | 8.34E+00 | 1.14E+00 | 1.43E-02 |
| NM_025611 | Cul7 | 9.28E-01 | 2.90E+00 | 1.14E+00 | 2.34E-02 |
| NM_138754 | Cdipt | 6.53E+00 | 2.04E+01 | 1.14E+00 | 1.29E-02 |
| NM_001130457 | Tbrg4 | 1.41E+00 | 4.41E+00 | 1.14E+00 | 3.70E-02 |
| NM_133721 | Itga9 | 1.16E+00 | 3.62E+00 | 1.14E+00 | 1.55E-02 |
| NM_030074 | Zfp687 | 1.12E+00 | 3.49E+00 | 1.14E+00 | 2.36E-02 |
| NM_001110205 | Acvr1 | 1.51E+00 | 4.70E+00 | 1.14E+00 | 2.96E-02 |
| NM_011261 | Reln | 5.62E+00 | 1.75E+01 | 1.14E+00 | 3.22E-02 |
| NR_033136 | Atf5 | 4.01E+01 | 1.25E+02 | 1.14E+00 | 3.24E-02 |
| NM_172505 | A730008H23Rik | 5.02E+00 | 1.56E+01 | 1.13E+00 | 1.32E-02 |
| NM_016748 | Ctps | 1.82E+00 | 5.67E+00 | 1.13E+00 | 2.58E-02 |
| NM_145973 | Ell3 | 2.59E+00 | 8.04E+00 | 1.13E+00 | 3.07E-02 |
| NM_029575 | Tgfbr2 | 9.63E+00 | 2.99E+01 | 1.13E+00 | 1.97E-02 |
| NM_007805 | Cyb561 | 3.85E+00 | 1.20E+01 | 1.13E+00 | 1.47E-02 |
| NM_001165985 | Ubap2l | 1.54E+00 | 4.78E+00 | 1.13E+00 | 2.24E-02 |
| NM_001205133 | Yes1 | 1.07E+00 | 3.32E+00 | 1.13E+00 | 4.93E-02 |
| NM_001081161 | Fam171a1 | 8.68E-01 | 2.69E+00 | 1.13E+00 | 3.51E-02 |
| NM_172758 | Slc38a7 | 1.98E+00 | 6.11E+00 | 1.13E+00 | 2.10E-02 |
| NM_022327 | Ralb | 2.93E+00 | 9.08E+00 | 1.13E+00 | 2.10E-02 |
| NM_026355 | Ergic2 | 3.01E+00 | 9.32E+00 | 1.13E+00 | 3.19E-02 |
| NM_207255 | Zfp532 | 7.18E-01 | 2.22E+00 | 1.13E+00 | 3.19E-02 |
| NM_001163031 | Cd97 | 5.85E+00 | 1.80E+01 | 1.13E+00 | 1.13E-02 |
| NR_027943 | 1110038B12Rik | 9.11E+00 | 2.80E+01 | 1.12E+00 | 3.55E-02 |
| NM_134091 | Sgsm3 | 1.06E+00 | 3.27E+00 | 1.12E+00 | 4.12E-02 |
| NM_011626 | Tmem165 | 4.04E+00 | 1.24E+01 | 1.12E+00 | 1.88E-02 |
| NM_009637 | Aebp2 | 4.46E+00 | 1.37E+01 | 1.12E+00 | 3.94E-02 |
| NM_001199485 | 4931406C07Rik | 7.50E+00 | 2.30E+01 | 1.12E+00 | 1.22E-02 |
| NM_001113331 | Shc1 | 1.18E+00 | 3.62E+00 | 1.12E+00 | 3.18E-02 |
| NM_009988 | Cxadr | 2.56E+00 | 7.84E+00 | 1.12E+00 | 3.16E-02 |
| NM_033609 | Med15 | 2.86E+00 | 8.76E+00 | 1.12E+00 | 1.56E-02 |
| NM_001164191 | Mtm1 | 1.42E+00 | 4.35E+00 | 1.12E+00 | 2.79E-02 |
| NM_007557 | Bmp7 | 2.37E+00 | 7.26E+00 | 1.12E+00 | 3.01E-02 |
| NM_013664 | Sh3gl1 | 5.00E+00 | 1.53E+01 | 1.12E+00 | 1.59E-02 |
| NM_001177610 | Ube2i | 4.95E+00 | 1.51E+01 | 1.12E+00 | 1.38E-02 |
| NM_133351 | Prss8 | 4.40E+00 | 1.34E+01 | 1.12E+00 | 1.84E-02 |
| NM_001168591 | Lonp2 | 8.62E+00 | 2.63E+01 | 1.11E+00 | 1.40E-02 |
| NM_016677 | Hpcal1 | 5.60E+00 | 1.70E+01 | 1.11E+00 | 1.85E-02 |
| NM_153319 | Amot | 1.91E+00 | 5.81E+00 | 1.11E+00 | 1.44E-02 |
| NM_007743 | Col1a2 | 2.00E+00 | 6.05E+00 | 1.11E+00 | 1.62E-02 |
| NM_001081337 | Sipa1l2 | 6.17E-01 | 1.86E+00 | 1.11E+00 | 3.50E-02 |
| NM_198127 | Abi2 | 8.10E-01 | 2.45E+00 | 1.11E+00 | 3.00E-02 |
| NM_011906 | Tpra1 | 5.61E+00 | 1.69E+01 | 1.10E+00 | 1.69E-02 |
| NM_016752 | Slc35b1 | 2.28E+01 | 6.89E+01 | 1.10E+00 | 1.34E-02 |
| NM_001198571 | Abi2 | 8.10E-01 | 2.44E+00 | 1.10E+00 | 3.01E-02 |
| NM_021521 | Med12 | 1.89E+00 | 5.69E+00 | 1.10E+00 | 1.33E-02 |
| NM_178407 | Arap2 | 4.62E+00 | 1.39E+01 | 1.10E+00 | 1.63E-02 |
| NM_001033348 | Ralgapa2 | 5.58E+00 | 1.68E+01 | 1.10E+00 | 2.75E-02 |
| NM_013723 | Podxl | 2.37E+00 | 7.14E+00 | 1.10E+00 | 1.45E-02 |
| NM_030138 | Acap2 | 3.11E+00 | 9.34E+00 | 1.10E+00 | 1.31E-02 |
| NM_011587 | Tie1 | 2.07E+00 | 6.21E+00 | 1.10E+00 | 1.98E-02 |
| NM_133794 | Qars | 7.28E+00 | 2.18E+01 | 1.10E+00 | 1.34E-02 |
| NM_010228 | Flt1 | 1.25E+00 | 3.74E+00 | 1.10E+00 | 2.01E-02 |
| NM_001002011 | Lmna | 3.94E+00 | 1.18E+01 | 1.10E+00 | 1.56E-02 |
| NM_001163689 | Pnpla2 | 9.76E+00 | 2.91E+01 | 1.09E+00 | 1.38E-02 |
| NM_001025257 | Vegfa | 2.86E+00 | 8.50E+00 | 1.09E+00 | 1.85E-02 |
| NM_024181 | Dnajc10 | 9.68E+00 | 2.88E+01 | 1.09E+00 | 1.82E-02 |
| NM_015733 | Casp9 | 1.53E+00 | 4.55E+00 | 1.09E+00 | 2.70E-02 |
| NM_031199 | Tgfa | 4.72E+00 | 1.40E+01 | 1.09E+00 | 1.45E-02 |
| NM_153412 | Phldb2 | 6.40E+00 | 1.90E+01 | 1.09E+00 | 1.80E-02 |
| NM_013471 | Anxa4 | 2.26E+01 | 6.71E+01 | 1.09E+00 | 1.93E-02 |
| NM_145554 | Ldlrap1 | 6.47E+00 | 1.92E+01 | 1.09E+00 | 1.50E-02 |
| NM_008809 | Pdgfrb | 6.93E-01 | 2.05E+00 | 1.08E+00 | 4.06E-02 |
| NM_001122832 | Eps15l1 | 1.11E+00 | 3.28E+00 | 1.08E+00 | 4.16E-02 |
| NM_009510 | Ezr | 1.33E+00 | 3.92E+00 | 1.08E+00 | 3.91E-02 |
| NM_001190855 | Pdlim5 | 1.70E+00 | 5.01E+00 | 1.08E+00 | 2.09E-02 |
| NM_001110268 | Vegfa | 2.86E+00 | 8.44E+00 | 1.08E+00 | 2.29E-02 |
| NM_001195083 | Phc2 | 2.42E+00 | 7.15E+00 | 1.08E+00 | 2.76E-02 |
| NM_145394 | Slc44a3 | 3.55E+00 | 1.05E+01 | 1.08E+00 | 2.05E-02 |
| NM_009287 | Stim1 | 4.81E+00 | 1.42E+01 | 1.08E+00 | 1.53E-02 |
| NM_008235 | Hes1 | 3.66E+00 | 1.08E+01 | 1.08E+00 | 3.36E-02 |
| NM_033314 | Slco2a1 | 1.03E+01 | 3.04E+01 | 1.08E+00 | 1.95E-02 |
| NM_010586 | Itpr2 | 1.53E+00 | 4.50E+00 | 1.08E+00 | 1.66E-02 |
| NM_025815 | Cpne8 | 9.80E-01 | 2.88E+00 | 1.08E+00 | 4.84E-02 |
| NM_021517 | Pdzk1 | 7.91E+00 | 2.33E+01 | 1.08E+00 | 1.50E-02 |
| NR_027888 | Sqrdl | 9.20E+00 | 2.70E+01 | 1.08E+00 | 1.65E-02 |
| NM_027102 | Esam | 3.92E+00 | 1.15E+01 | 1.08E+00 | 2.54E-02 |
| NM_022324 | Sdf2l1 | 1.43E+01 | 4.19E+01 | 1.08E+00 | 1.66E-02 |
| NM_181395 | Pxdn | 1.07E+00 | 3.13E+00 | 1.07E+00 | 2.44E-02 |
| NM_207176 | Tes | 1.53E+00 | 4.48E+00 | 1.07E+00 | 4.56E-02 |
| NM_030706 | Trim2 | 1.79E+00 | 5.24E+00 | 1.07E+00 | 1.90E-02 |
| NM_153406 | Specc1l | 2.13E+00 | 6.25E+00 | 1.07E+00 | 1.68E-02 |
| NM_001161411 | Ttc15 | 1.33E-01 | 3.88E-01 | 1.07E+00 | 1.65E-02 |
| NM_009752 | Glb1 | 3.50E+00 | 1.02E+01 | 1.07E+00 | 2.39E-02 |
| NM_001103177 | Ablim1 | 2.72E+00 | 7.91E+00 | 1.07E+00 | 1.63E-02 |
| NM_001101561 | Gm6251 | 3.17E+01 | 9.23E+01 | 1.07E+00 | 1.76E-02 |
| NM_011816 | G3bp2 | 2.08E+00 | 6.05E+00 | 1.07E+00 | 2.16E-02 |
| NM_009500 | Vav2 | 3.59E+00 | 1.04E+01 | 1.07E+00 | 1.83E-02 |
| NM_133941 | Dhx32 | 8.25E+00 | 2.40E+01 | 1.07E+00 | 1.62E-02 |
| NM_144528 | Rnf126 | 3.45E+00 | 1.00E+01 | 1.07E+00 | 3.47E-02 |
| NM_172647 | F11r | 2.62E+01 | 7.59E+01 | 1.06E+00 | 3.65E-02 |
| NM_011774 | Slc30a4 | 3.32E+00 | 9.60E+00 | 1.06E+00 | 1.63E-02 |
| NM_021563 | Erbb2ip | 3.64E+00 | 1.05E+01 | 1.06E+00 | 1.61E-02 |
| NM_025739 | Rnf220 | 1.49E+00 | 4.29E+00 | 1.06E+00 | 4.15E-02 |
| NM_010117 | Rhbdf1 | 2.78E+00 | 8.01E+00 | 1.06E+00 | 2.47E-02 |
| NM_016974 | Dbp | 1.69E+01 | 4.86E+01 | 1.06E+00 | 1.67E-02 |
| NM_027129 | 2310035K24Rik | 4.33E+00 | 1.25E+01 | 1.06E+00 | 3.36E-02 |
| NM_177092 | Msrb3 | 4.84E+00 | 1.39E+01 | 1.06E+00 | 1.75E-02 |
| NM_026373 | Cdk2ap2 | 2.85E+01 | 8.19E+01 | 1.06E+00 | 1.73E-02 |
| NM_023124 | H2-Q8 | 6.79E+00 | 1.95E+01 | 1.05E+00 | 2.79E-02 |
| NM_001160378 | Fam46a | 1.70E+00 | 4.86E+00 | 1.05E+00 | 2.34E-02 |
| NM_146116 | Tubb2c | 4.51E+01 | 1.29E+02 | 1.05E+00 | 4.02E-02 |
| NM_199080 | Ddx17 | 1.93E+00 | 5.52E+00 | 1.05E+00 | 2.35E-02 |
| NM_011218 | Ptprs | 8.00E-01 | 2.28E+00 | 1.05E+00 | 3.53E-02 |
| NM_210071 | Eya3 | 1.87E+00 | 5.33E+00 | 1.05E+00 | 2.34E-02 |
| NM_026376 | Plxnd1 | 2.78E+00 | 7.90E+00 | 1.05E+00 | 1.81E-02 |
| NM_028783 | Robo4 | 2.07E+00 | 5.89E+00 | 1.04E+00 | 2.75E-02 |
| NM_010740 | Cd93 | 2.78E+00 | 7.89E+00 | 1.04E+00 | 1.84E-02 |
| NM_016667 | Sntb1 | 1.46E+01 | 4.15E+01 | 1.04E+00 | 1.87E-02 |
| NM_178619 | 1810026J23Rik | 2.96E+00 | 8.41E+00 | 1.04E+00 | 2.44E-02 |
| NM_001161853 | Smarcb1 | 3.82E+00 | 1.08E+01 | 1.04E+00 | 3.52E-02 |
| NM_009281 | Zfp143 | 1.76E+00 | 4.99E+00 | 1.04E+00 | 3.84E-02 |
| NM_031376 | Pik3ap1 | 1.47E+01 | 4.14E+01 | 1.04E+00 | 2.28E-02 |
| NM_011405 | Slc7a7 | 3.28E+00 | 9.28E+00 | 1.04E+00 | 3.01E-02 |
| NM_172731 | Fgd5 | 8.42E-01 | 2.38E+00 | 1.04E+00 | 4.15E-02 |
| NM_011400 | Slc2a1 | 1.93E+00 | 5.44E+00 | 1.04E+00 | 4.15E-02 |
| NM_053194 | Ric8 | 4.09E+00 | 1.15E+01 | 1.04E+00 | 2.17E-02 |
| NM_010681 | Lama4 | 8.46E-01 | 2.39E+00 | 1.04E+00 | 3.94E-02 |
| NM_001198876 | Dync1i2 | 3.74E+00 | 1.06E+01 | 1.04E+00 | 2.56E-02 |
| NM_020024 | Taf10 | 1.31E+01 | 3.69E+01 | 1.04E+00 | 2.88E-02 |
| NM_001159354 | Magi3 | 3.94E+00 | 1.11E+01 | 1.04E+00 | 1.90E-02 |
| NM_001160181 | Tor1aip2 | 3.86E+00 | 1.09E+01 | 1.04E+00 | 2.45E-02 |
| NM_016721 | Iqgap1 | 2.71E+00 | 7.62E+00 | 1.03E+00 | 1.89E-02 |
| NM_009242 | Sparc | 3.08E+01 | 8.67E+01 | 1.03E+00 | 4.26E-02 |
| NM_001039509 | Pnkd | 2.75E+00 | 7.71E+00 | 1.03E+00 | 2.89E-02 |
| NM_001113553 | Irak2 | 1.46E+00 | 4.10E+00 | 1.03E+00 | 4.55E-02 |
| NM_008363 | Irak1 | 3.93E+00 | 1.10E+01 | 1.03E+00 | 2.12E-02 |
| NM_010107 | Efna1 | 1.85E+01 | 5.18E+01 | 1.03E+00 | 2.03E-02 |
| NM_009146 | Frrs1 | 4.70E+00 | 1.31E+01 | 1.03E+00 | 2.45E-02 |
| NM_028633 | Haus7 | 6.72E+00 | 1.88E+01 | 1.03E+00 | 2.85E-02 |
| NM_009713 | Arsa | 7.17E+00 | 2.00E+01 | 1.03E+00 | 2.01E-02 |
| NM_011596 | Atp6v0a2 | 4.32E+00 | 1.21E+01 | 1.03E+00 | 2.22E-02 |
| NM_011704 | Vnn1 | 1.74E+01 | 4.85E+01 | 1.03E+00 | 2.58E-02 |
| NM_028078 | Igsf5 | 1.34E+01 | 3.74E+01 | 1.03E+00 | 2.02E-02 |
| NM_018822 | Sgsh | 1.01E+00 | 2.83E+00 | 1.03E+00 | 4.74E-02 |
| NM_016926 | Sart3 | 1.46E+00 | 4.07E+00 | 1.02E+00 | 4.16E-02 |
| NM_001008550 | Zfyve26 | 2.45E+00 | 6.81E+00 | 1.02E+00 | 1.94E-02 |
| NM_009722 | Atp2a2 | 2.19E+00 | 6.07E+00 | 1.02E+00 | 2.71E-02 |
| NM_013843 | Zfp53 | 1.59E+00 | 4.41E+00 | 1.02E+00 | 4.47E-02 |
| NM_001201460 | H2-Q9 | 1.86E+01 | 5.17E+01 | 1.02E+00 | 2.08E-02 |
| NM_001165986 | Ubap2l | 1.73E+00 | 4.79E+00 | 1.02E+00 | 3.72E-02 |
| NM_001160411 | Gstm4 | 3.90E+00 | 1.08E+01 | 1.02E+00 | 4.27E-02 |
| NM_001163489 | Sema4a | 2.50E+00 | 6.92E+00 | 1.02E+00 | 3.17E-02 |
| NM_011715 | Wdr1 | 1.59E+01 | 4.42E+01 | 1.02E+00 | 2.70E-02 |
| NM_001009818 | 11-Sep | 2.45E+00 | 6.80E+00 | 1.02E+00 | 2.40E-02 |
| NM_001045959 | Mink1 | 2.16E+00 | 5.97E+00 | 1.02E+00 | 2.55E-02 |
| NM_012027 | Mprip | 3.76E+00 | 1.04E+01 | 1.02E+00 | 2.25E-02 |
| NM_201373 | Trim56 | 2.21E+00 | 6.12E+00 | 1.02E+00 | 3.08E-02 |
| NR_037865 | Ankrd11 | 1.53E+00 | 4.23E+00 | 1.02E+00 | 2.35E-02 |
| NM_001081057 | Tecpr2 | 8.97E-01 | 2.48E+00 | 1.02E+00 | 3.35E-02 |
| NM_027556 | Cep192 | 6.75E-01 | 1.86E+00 | 1.01E+00 | 4.14E-02 |
| NM_008787 | Pcnt | 4.70E-01 | 1.29E+00 | 1.01E+00 | 4.77E-02 |
| NM_001083317 | Slc35a4 | 1.69E+00 | 4.65E+00 | 1.01E+00 | 4.90E-02 |
| NM_001145947 | Bace1 | 1.11E+00 | 3.05E+00 | 1.01E+00 | 4.86E-02 |
| NM_023913 | Ern1 | 1.09E+01 | 2.99E+01 | 1.01E+00 | 2.82E-02 |
| NM_026232 | Slc25a30 | 1.19E+01 | 3.26E+01 | 1.01E+00 | 2.25E-02 |
| NM_001160400 | Megf8 | 8.89E-01 | 2.44E+00 | 1.01E+00 | 2.96E-02 |
| NM_145475 | Cerk | 1.93E+00 | 5.27E+00 | 1.01E+00 | 3.07E-02 |
| NM_021390 | Sall1 | 5.12E+00 | 1.40E+01 | 1.01E+00 | 2.25E-02 |
| NM_175256 | Heg1 | 2.41E+00 | 6.59E+00 | 1.01E+00 | 2.68E-02 |
| NM_011962 | Plod3 | 3.82E+00 | 1.04E+01 | 1.01E+00 | 2.56E-02 |
| NM_001145884 | Itgb5 | 1.14E+01 | 3.11E+01 | 1.00E+00 | 2.41E-02 |
| NM_013646 | Rora | 1.18E+01 | 3.21E+01 | 1.00E+00 | 2.34E-02 |
| NM_027446 | Fam86 | 2.46E+00 | 6.69E+00 | 1.00E+00 | 4.82E-02 |
| NM_011099 | Pkm2 | 1.09E+01 | 2.96E+01 | 9.99E-01 | 2.30E-02 |
| NM_001177980 | Pde4b | 1.20E+00 | 3.26E+00 | 9.99E-01 | 4.80E-02 |
| NM_054081 | Mta1 | 5.62E+00 | 1.52E+01 | 9.98E-01 | 2.43E-02 |
| NM_001205043 | Jarid2 | 8.05E-01 | 2.18E+00 | 9.98E-01 | 4.90E-02 |
| NM_025445 | Arfgap3 | 4.09E+00 | 1.11E+01 | 9.98E-01 | 2.92E-02 |
| NM_173427 | Klhdc7a | 3.97E+00 | 1.08E+01 | 9.97E-01 | 2.30E-02 |
| NM_009868 | Cdh5 | 7.34E+00 | 1.99E+01 | 9.97E-01 | 2.35E-02 |
| NM_178716 | Tnpo1 | 3.96E+00 | 1.07E+01 | 9.96E-01 | 2.29E-02 |
| NM_001080796 | G3bp2 | 2.10E+00 | 5.67E+00 | 9.95E-01 | 3.23E-02 |
| NM_134076 | Abhd4 | 1.12E+01 | 3.01E+01 | 9.92E-01 | 2.47E-02 |
| NM_026269 | Get4 | 4.64E+00 | 1.25E+01 | 9.92E-01 | 3.23E-02 |
| NM_009419 | Tpst2 | 7.55E+00 | 2.03E+01 | 9.91E-01 | 2.74E-02 |
| NM_001033342 | Cdc42bpg | 1.77E+00 | 4.77E+00 | 9.90E-01 | 2.97E-02 |
| NM_198600 | Papd7 | 2.40E+00 | 6.47E+00 | 9.90E-01 | 3.23E-02 |
| NM_016769 | Smad3 | 2.96E+00 | 7.95E+00 | 9.90E-01 | 2.54E-02 |
| NM_009461 | Ubr1 | 1.83E+00 | 4.91E+00 | 9.88E-01 | 2.66E-02 |
| NM_029561 | Ndfip2 | 7.67E+00 | 2.06E+01 | 9.87E-01 | 2.53E-02 |
| NM_010511 | Ifngr1 | 1.62E+01 | 4.36E+01 | 9.86E-01 | 2.57E-02 |
| NM_178682 | 4933426M11Rik | 5.83E+00 | 1.56E+01 | 9.86E-01 | 2.54E-02 |
| NM_011436 | Sorl1 | 9.03E-01 | 2.42E+00 | 9.86E-01 | 4.40E-02 |
| NM_024223 | Crip2 | 1.57E+01 | 4.20E+01 | 9.86E-01 | 2.49E-02 |
| NM_019575 | Scamp4 | 5.71E+00 | 1.53E+01 | 9.83E-01 | 3.34E-02 |
| NM_001111279 | Wdfy1 | 2.35E+00 | 6.27E+00 | 9.81E-01 | 3.12E-02 |
| NM_183017 | Ttll12 | 1.71E+00 | 4.55E+00 | 9.81E-01 | 4.52E-02 |
| NM_001081413 | Unc13b | 1.26E+00 | 3.36E+00 | 9.80E-01 | 3.67E-02 |
| NM_007600 | Capn1 | 1.99E+00 | 5.29E+00 | 9.80E-01 | 4.57E-02 |
| NM_001162872 | Exoc7 | 3.65E+00 | 9.71E+00 | 9.79E-01 | 3.08E-02 |
| NM_007930 | Enc1 | 3.60E+00 | 9.57E+00 | 9.77E-01 | 2.71E-02 |
| NM_009284 | Stat6 | 8.46E+00 | 2.25E+01 | 9.77E-01 | 2.70E-02 |
| NM_001166630 | Dynlt1c | 6.72E+00 | 1.78E+01 | 9.76E-01 | 1.14E-02 |
| NM_147219 | Abca5 | 1.13E+00 | 3.00E+00 | 9.76E-01 | 3.46E-02 |
| NM_001199685 | Taok3 | 3.59E+00 | 9.51E+00 | 9.75E-01 | 2.76E-02 |
| NM_020009 | Mtor | 3.67E+00 | 9.74E+00 | 9.75E-01 | 2.75E-02 |
| NM_028352 | Pgm3 | 5.44E+00 | 1.44E+01 | 9.72E-01 | 2.59E-02 |
| NM_011779 | Coro1c | 5.76E+00 | 1.52E+01 | 9.72E-01 | 2.68E-02 |
| NM_009320 | Slc6a6 | 9.85E+00 | 2.60E+01 | 9.70E-01 | 4.78E-02 |
| NM_009743 | Bcl2l1 | 2.00E+01 | 5.27E+01 | 9.70E-01 | 3.57E-02 |
| NM_001081274 | Pgd | 2.31E+01 | 6.10E+01 | 9.69E-01 | 3.58E-02 |
| NM_010026 | Asap1 | 2.69E+00 | 7.09E+00 | 9.69E-01 | 3.16E-02 |
| NM_177725 | Lrrc8a | 8.26E+00 | 2.17E+01 | 9.68E-01 | 2.93E-02 |
| NM_001080118 | Med1 | 9.94E-01 | 2.62E+00 | 9.67E-01 | 4.62E-02 |
| NM_008706 | Nqo1 | 9.02E+00 | 2.37E+01 | 9.67E-01 | 3.10E-02 |
| NM_145987 | Tmem82 | 3.63E+00 | 9.53E+00 | 9.66E-01 | 4.40E-02 |
| NM_001099632 | Rnf39 | 9.18E+00 | 2.41E+01 | 9.64E-01 | 3.31E-02 |
| NM_001168218 | 2310008H09Rik | 2.21E+00 | 5.79E+00 | 9.63E-01 | 3.41E-02 |
| NM_153136 | Nudt18 | 2.49E+00 | 6.52E+00 | 9.63E-01 | 3.75E-02 |
| NM_020619 | Mogs | 7.55E+00 | 1.98E+01 | 9.63E-01 | 2.81E-02 |
| NM_012037 | Vat1 | 9.92E+00 | 2.60E+01 | 9.62E-01 | 2.87E-02 |
| NM_025999 | Rnf141 | 1.02E+01 | 2.66E+01 | 9.61E-01 | 4.12E-02 |
| NM_021890 | Fads3 | 3.10E+00 | 8.09E+00 | 9.60E-01 | 3.66E-02 |
| NM_001159521 | Plxnb2 | 6.59E+00 | 1.72E+01 | 9.59E-01 | 3.70E-02 |
| NM_134089 | Scrib | 1.78E+00 | 4.64E+00 | 9.58E-01 | 3.70E-02 |
| NM_178661 | Creb3l2 | 7.69E+00 | 2.00E+01 | 9.58E-01 | 2.78E-02 |
| NM_001033228 | Itga1 | 3.73E+00 | 9.73E+00 | 9.58E-01 | 2.93E-02 |
| NM_021287 | Spnb3 | 4.59E+00 | 1.19E+01 | 9.56E-01 | 3.13E-02 |
| NM_009886 | Celsr1 | 8.06E-01 | 2.10E+00 | 9.56E-01 | 3.90E-02 |
| NM_001167939 | Mau2 | 2.17E+00 | 5.65E+00 | 9.54E-01 | 3.47E-02 |
| NM_008199 | H2-Bl | 1.33E+01 | 3.45E+01 | 9.54E-01 | 3.23E-02 |
| NM_010227 | Flna | 4.34E+00 | 1.13E+01 | 9.54E-01 | 3.10E-02 |
| NM_015806 | Mapk6 | 2.42E+00 | 6.27E+00 | 9.54E-01 | 3.88E-02 |
| NM_008150 | Gpc4 | 1.47E+01 | 3.81E+01 | 9.52E-01 | 3.06E-02 |
| NM_001206335 | Itfg3 | 4.67E+00 | 1.21E+01 | 9.52E-01 | 3.36E-02 |
| NM_029337 | Ep400 | 2.17E+00 | 5.61E+00 | 9.52E-01 | 2.93E-02 |
| NM_194334 | Tbc1d2b | 7.10E+00 | 1.84E+01 | 9.51E-01 | 3.89E-02 |
| NM_001205082 | Scarb1 | 7.63E+00 | 1.98E+01 | 9.51E-01 | 3.12E-02 |
| NM_008909 | Ppl | 6.01E+00 | 1.55E+01 | 9.50E-01 | 3.20E-02 |
| NM_133796 | Arhgdia | 2.09E+01 | 5.40E+01 | 9.49E-01 | 4.95E-02 |
| NM_010495 | Id1 | 1.02E+01 | 2.64E+01 | 9.49E-01 | 4.55E-02 |
| NM_024267 | Ipo4 | 4.91E+00 | 1.27E+01 | 9.49E-01 | 3.15E-02 |
| NM_013630 | Pkd1 | 1.27E+00 | 3.27E+00 | 9.48E-01 | 3.14E-02 |
| NM_001033272 | Spata13 | 2.39E+00 | 6.17E+00 | 9.48E-01 | 3.13E-02 |
| NM_134082 | Farp1 | 4.00E+00 | 1.03E+01 | 9.48E-01 | 3.15E-02 |
| NM_001111044 | Serpinh1 | 3.27E+00 | 8.43E+00 | 9.48E-01 | 4.82E-02 |
| NM_007977 | F8 | 1.05E+00 | 2.71E+00 | 9.46E-01 | 4.44E-02 |
| NM_001199025 | Pla2g6 | 2.86E+00 | 7.36E+00 | 9.46E-01 | 4.32E-02 |
| NM_030201 | Hspa13 | 7.74E+00 | 1.99E+01 | 9.45E-01 | 3.12E-02 |
| NM_001114361 | Eml4 | 2.61E+00 | 6.72E+00 | 9.44E-01 | 3.41E-02 |
| NM_027216 | Slc39a11 | 3.16E+00 | 8.11E+00 | 9.44E-01 | 4.33E-02 |
| NM_008513 | Lrp5 | 6.73E+00 | 1.73E+01 | 9.42E-01 | 3.36E-02 |
| NM_026730 | Gpihbp1 | 1.86E+01 | 4.77E+01 | 9.42E-01 | 3.74E-02 |
| NM_033325 | Loxl2 | 1.68E+00 | 4.31E+00 | 9.41E-01 | 4.34E-02 |
| NM_019588 | Plce1 | 2.10E+00 | 5.39E+00 | 9.40E-01 | 3.15E-02 |
| NM_172977 | Gtf3c4 | 1.77E+00 | 4.54E+00 | 9.40E-01 | 3.63E-02 |
| NM_001242396 | Jmjd1c | 1.28E+00 | 3.26E+00 | 9.39E-01 | 3.92E-02 |
| NM_027835 | Ifih1 | 4.06E+00 | 1.04E+01 | 9.37E-01 | 3.17E-02 |
| NM_010928 | Notch2 | 4.61E+00 | 1.18E+01 | 9.36E-01 | 4.28E-02 |
| NM_008402 | Itgav | 4.05E+00 | 1.03E+01 | 9.34E-01 | 3.27E-02 |
| NM_016871 | Tomm40 | 5.66E+00 | 1.44E+01 | 9.34E-01 | 4.68E-02 |
| NM_019426 | Atf7ip | 2.37E+00 | 6.03E+00 | 9.32E-01 | 4.06E-02 |
| NM_010390 | H2-Q1 | 1.58E+01 | 4.02E+01 | 9.32E-01 | 3.44E-02 |
| NM_172579 | Sipa1l1 | 1.41E+00 | 3.57E+00 | 9.31E-01 | 4.08E-02 |
| NM_033218 | Srebf2 | 5.75E+00 | 1.46E+01 | 9.29E-01 | 3.28E-02 |
| NM_001081364 | Arhgap21 | 2.99E+00 | 7.55E+00 | 9.28E-01 | 3.38E-02 |
| NM_001099637 | Cep170 | 1.87E+00 | 4.71E+00 | 9.27E-01 | 3.79E-02 |
| NM_054040 | Tulp4 | 3.55E+00 | 8.95E+00 | 9.24E-01 | 3.75E-02 |
| NM_007803 | Cttn | 1.26E+01 | 3.18E+01 | 9.23E-01 | 3.67E-02 |
| NM_139306 | Acer2 | 5.51E+00 | 1.39E+01 | 9.23E-01 | 3.41E-02 |
| NM_172424 | Med13l | 1.34E+00 | 3.36E+00 | 9.21E-01 | 4.02E-02 |
| NM_001083334 | Bin1 | 4.55E+00 | 1.14E+01 | 9.21E-01 | 4.69E-02 |
| NM_153781 | Pygb | 5.13E+00 | 1.29E+01 | 9.20E-01 | 3.57E-02 |
| NM_153782 | Fam20a | 7.81E+00 | 1.96E+01 | 9.18E-01 | 3.66E-02 |
| NM_001039147 | Morf4l1 | 5.55E+00 | 1.39E+01 | 9.17E-01 | 4.52E-02 |
| NM_172924 | C230081A13Rik | 2.05E+00 | 5.12E+00 | 9.17E-01 | 3.50E-02 |
| NM_021557 | Rdh11 | 2.11E+01 | 5.26E+01 | 9.15E-01 | 3.67E-02 |
| NM_172903 | Man2a2 | 3.83E+00 | 9.55E+00 | 9.13E-01 | 3.62E-02 |
| NM_027706 | Nbas | 2.76E+00 | 6.86E+00 | 9.12E-01 | 3.75E-02 |
| NM_029653 | Dapk1 | 3.67E+00 | 9.12E+00 | 9.10E-01 | 3.72E-02 |
| NM_001171147 | Yap1 | 8.00E+00 | 1.99E+01 | 9.09E-01 | 3.80E-02 |
| NM_001077411 | Gba | 8.28E+00 | 2.05E+01 | 9.09E-01 | 4.16E-02 |
| NM_177016 | Slc17a4 | 1.17E+01 | 2.90E+01 | 9.08E-01 | 3.80E-02 |
| NM_033320 | Glce | 5.07E+00 | 1.26E+01 | 9.07E-01 | 3.68E-02 |
| NM_145556 | Tardbp | 5.06E+00 | 1.25E+01 | 9.07E-01 | 3.97E-02 |
| NM_172935 | Amdhd2 | 7.25E+00 | 1.79E+01 | 9.06E-01 | 4.94E-02 |
| NM_178696 | Slc25a44 | 6.08E+00 | 1.50E+01 | 9.05E-01 | 3.75E-02 |
| NM_176837 | Arhgap18 | 3.56E+00 | 8.81E+00 | 9.05E-01 | 4.38E-02 |
| NM_027910 | Klhdc3 | 6.25E+00 | 1.54E+01 | 9.01E-01 | 4.48E-02 |
| NM_145633 | Ankrd27 | 4.08E+00 | 1.00E+01 | 9.00E-01 | 4.09E-02 |
| NM_015762 | Txnrd1 | 1.44E+01 | 3.55E+01 | 8.99E-01 | 4.85E-02 |
| NM_013490 | Chka | 7.10E+00 | 1.74E+01 | 8.98E-01 | 4.13E-02 |
| NR_037854 | Atp6v0c-ps2 | 3.68E+01 | 9.02E+01 | 8.97E-01 | 4.12E-02 |
| NM_008064 | Gaa | 5.46E+00 | 1.34E+01 | 8.96E-01 | 4.03E-02 |
| NM_001042675 | Rbpms | 1.26E+01 | 3.09E+01 | 8.96E-01 | 4.84E-02 |
| NM_001080748 | Gtf2i | 3.38E+00 | 8.23E+00 | 8.91E-01 | 4.43E-02 |
| NM_028123 | Slc37a3 | 5.42E+00 | 1.32E+01 | 8.89E-01 | 4.13E-02 |
| NM_001146199 | Ptpn21 | 2.14E+00 | 5.18E+00 | 8.87E-01 | 4.86E-02 |
| NM_001166552 | Zbed6 | 7.80E+00 | 1.89E+01 | 8.86E-01 | 4.41E-02 |
| NM_134062 | Dapk1 | 3.37E+00 | 8.16E+00 | 8.85E-01 | 4.37E-02 |
| NM_173444 | Nbeal1 | 2.65E+00 | 6.42E+00 | 8.85E-01 | 4.25E-02 |
| NM_001164661 | Cyfip1 | 3.99E+00 | 9.65E+00 | 8.84E-01 | 4.17E-02 |
| NM_011597 | Tjp2 | 3.20E+00 | 7.74E+00 | 8.83E-01 | 4.63E-02 |
| NM_013747 | Golga5 | 6.49E+00 | 1.57E+01 | 8.82E-01 | 4.50E-02 |
| NM_009902 | Cldn3 | 2.26E+01 | 5.46E+01 | 8.82E-01 | 4.36E-02 |
| NM_001079686 | Syne1 | 1.63E+00 | 3.93E+00 | 8.82E-01 | 4.41E-02 |
| NM_001039394 | Rab43 | 8.96E+00 | 2.16E+01 | 8.82E-01 | 4.54E-02 |
| NM_011808 | Ets1 | 3.12E+00 | 7.52E+00 | 8.79E-01 | 4.58E-02 |
| NM_011609 | Tnfrsf1a | 1.99E+01 | 4.79E+01 | 8.79E-01 | 4.63E-02 |
| NM_021607 | Ncstn | 1.01E+01 | 2.43E+01 | 8.78E-01 | 4.35E-02 |
| NM_021500 | Maea | 1.13E+01 | 2.69E+01 | 8.72E-01 | 4.50E-02 |
| NM_138587 | Fam3c | 1.44E+01 | 3.44E+01 | 8.72E-01 | 4.84E-02 |
| NM_146110 | Aamp | 1.87E+01 | 4.47E+01 | 8.70E-01 | 4.66E-02 |
| NM_146064 | Soat2 | 9.29E+00 | 2.22E+01 | 8.69E-01 | 4.74E-02 |
| NM_001039939 | Asxl1 | 2.70E+00 | 6.44E+00 | 8.69E-01 | 4.73E-02 |
| NM_027106 | Avpi1 | 3.35E+01 | 7.97E+01 | 8.67E-01 | 4.52E-02 |
| NM_013720 | Mga | 1.60E+00 | 3.80E+00 | 8.67E-01 | 4.58E-02 |
| NM_001081362 | Trrap | 2.39E+00 | 5.66E+00 | 8.65E-01 | 4.70E-02 |
| NM_172608 | Tmem184b | 6.38E+00 | 1.51E+01 | 8.59E-01 | 4.87E-02 |
| NM_030886 | Ankrd17 | 2.95E+00 | 6.95E+00 | 8.58E-01 | 4.85E-02 |
| NM_080837 | D17Wsu104e | 3.86E+01 | 9.06E+01 | 8.54E-01 | 4.86E-02 |
| NM_133825 | D1Ertd622e | 8.40E+00 | 1.97E+01 | 8.53E-01 | 4.85E-02 |
| NM_153131 | Unc5a | 8.10E-03 | 1.71E-02 | 7.50E-01 | 1.02E-02 |
| NM_008218 | Hba-a1 | 3.83E+02 | 7.40E+02 | 6.58E-01 | 4.94E-02 |
| NM_001109686 | Ehmt1 | 6.03E-03 | 8.50E-03 | 3.43E-01 | 2.33E-02 |
| NM_001110499 | Canx | 1.72E+01 | 7.96E+00 | -7.71E-01 | 4.99E-02 |
| NM_001199043 | Lgals8 | 2.32E+01 | 1.07E+01 | -7.76E-01 | 4.84E-02 |
| NM_001113210 | Nfib | 8.16E+00 | 3.76E+00 | -7.76E-01 | 4.75E-02 |
| NM_025570 | Mrpl20 | 1.02E+02 | 4.68E+01 | -7.77E-01 | 4.85E-02 |
| NR_040453 | Gm17757 | 1.30E+01 | 5.96E+00 | -7.79E-01 | 7.52E-03 |
| NM_053108 | Glrx | 3.69E+01 | 1.69E+01 | -7.81E-01 | 4.90E-02 |
| NM_177124 | Tnrc6b | 2.48E+00 | 1.14E+00 | -7.81E-01 | 4.92E-02 |
| NM_001040399 | Larp1b | 4.38E+01 | 2.01E+01 | -7.81E-01 | 4.75E-02 |
| NM_001113209 | Nfib | 8.17E+00 | 3.73E+00 | -7.83E-01 | 4.58E-02 |
| NM_008183 | Gstm2 | 9.81E+01 | 4.47E+01 | -7.86E-01 | 4.72E-02 |
| NM_027293 | Dopey2 | 6.36E+00 | 2.90E+00 | -7.87E-01 | 4.57E-02 |
| NM_001160016 | Gnb1 | 1.29E+01 | 5.87E+00 | -7.87E-01 | 4.86E-02 |
| NM_134014 | Xpo1 | 8.07E+00 | 3.67E+00 | -7.88E-01 | 4.70E-02 |
| NM_025316 | Ndufb5 | 1.13E+02 | 5.14E+01 | -7.90E-01 | 4.72E-02 |
| NM_009460 | Sumo1 | 6.43E+01 | 2.92E+01 | -7.90E-01 | 4.41E-02 |
| NM_008210 | H3f3a | 9.86E+01 | 4.46E+01 | -7.92E-01 | 4.51E-02 |
| NM_001081295 | Arhgef26 | 1.25E+01 | 5.62E+00 | -7.96E-01 | 4.20E-02 |
| NM_019826 | Ivd | 5.12E+01 | 2.31E+01 | -7.97E-01 | 4.59E-02 |
| NM_172733 | Dera | 3.36E+01 | 1.51E+01 | -7.99E-01 | 4.14E-02 |
| NR_040456 | Gm18853 | 1.30E+01 | 5.83E+00 | -8.00E-01 | 6.12E-03 |
| NM_145541 | Rap1a | 2.88E+01 | 1.29E+01 | -8.04E-01 | 4.59E-02 |
| NM_025384 | Dnajc15 | 6.42E+01 | 2.86E+01 | -8.08E-01 | 4.83E-02 |
| NM_001159392 | Tnfaip1 | 1.04E+01 | 4.63E+00 | -8.11E-01 | 4.24E-02 |
| NM_027418 | Mapk6 | 8.01E+00 | 3.55E+00 | -8.12E-01 | 4.53E-02 |
| NM_001145952 | Lpp | 6.31E+00 | 2.80E+00 | -8.12E-01 | 4.15E-02 |
| NM_010270 | Mrps33 | 4.30E+01 | 1.91E+01 | -8.14E-01 | 4.17E-02 |
| NM_026178 | Mmd | 5.52E+01 | 2.45E+01 | -8.14E-01 | 4.87E-02 |
| NM_009648 | Akap1 | 1.20E+01 | 5.33E+00 | -8.14E-01 | 4.01E-02 |
| NM_026305 | Tceb2 | 1.01E+02 | 4.46E+01 | -8.16E-01 | 4.52E-02 |
| NM_026065 | Mrpl42 | 7.57E+01 | 3.34E+01 | -8.18E-01 | 4.13E-02 |
| NM_025624 | Pomp | 2.14E+02 | 9.45E+01 | -8.19E-01 | 4.29E-02 |
| NM_001128609 | Dedd | 1.13E+01 | 4.95E+00 | -8.21E-01 | 4.52E-02 |
| NM_001114322 | Cdhr5 | 1.23E+01 | 5.43E+00 | -8.21E-01 | 4.40E-02 |
| NM_001040187 | Ddx17 | 7.09E+00 | 3.12E+00 | -8.21E-01 | 4.20E-02 |
| NM_174847 | C2cd2 | 2.27E+01 | 9.99E+00 | -8.22E-01 | 4.80E-02 |
| NM_019773 | Rab9 | 4.61E+01 | 2.02E+01 | -8.24E-01 | 3.55E-02 |
| NM_009676 | Aox1 | 2.02E+01 | 8.85E+00 | -8.25E-01 | 3.66E-02 |
| NM_019480 | Ebag9 | 1.93E+01 | 8.45E+00 | -8.26E-01 | 4.63E-02 |
| NM_145418 | BC013529 | 4.61E+01 | 2.02E+01 | -8.26E-01 | 3.48E-02 |
| NM_172920 | Dpy19l1 | 1.43E+01 | 6.27E+00 | -8.27E-01 | 3.48E-02 |
| NM_175009 | Eny2 | 1.61E+01 | 7.05E+00 | -8.27E-01 | 3.86E-02 |
| NM_008537 | Amacr | 4.97E+01 | 2.17E+01 | -8.28E-01 | 4.15E-02 |
| NM_001166368 | Fkbp2 | 8.72E+01 | 3.81E+01 | -8.29E-01 | 3.77E-02 |
| NM_029573 | Idh3a | 2.07E+01 | 9.04E+00 | -8.30E-01 | 3.50E-02 |
| NM_009408 | Top1 | 3.55E+01 | 1.55E+01 | -8.31E-01 | 4.37E-02 |
| NM_027903 | Dhdh | 5.99E+01 | 2.61E+01 | -8.31E-01 | 4.84E-02 |
| NM_010516 | Cyr61 | 1.60E+01 | 6.97E+00 | -8.34E-01 | 4.12E-02 |
| NM_001081389 | Nlrp6 | 2.67E+01 | 1.16E+01 | -8.35E-01 | 3.61E-02 |
| NM_008390 | Irf1 | 1.65E+01 | 7.14E+00 | -8.36E-01 | 3.86E-02 |
| NM_026183 | Slc47a1 | 3.83E+01 | 1.66E+01 | -8.39E-01 | 3.47E-02 |
| NM_026352 | Ppid | 2.06E+01 | 8.89E+00 | -8.39E-01 | 3.88E-02 |
| NM_031195 | Msr1 | 7.29E+00 | 3.15E+00 | -8.39E-01 | 4.36E-02 |
| NM_010023 | Eci1 | 8.44E+01 | 3.65E+01 | -8.40E-01 | 3.24E-02 |
| NM_007611 | Casp7 | 1.20E+01 | 5.20E+00 | -8.40E-01 | 4.34E-02 |
| NM_021412 | Mmp19 | 1.08E+01 | 4.65E+00 | -8.45E-01 | 3.52E-02 |
| NM_172782 | Nxt2 | 1.16E+01 | 4.99E+00 | -8.45E-01 | 4.14E-02 |
| NM_029272 | Ndufs7 | 8.44E+01 | 3.62E+01 | -8.47E-01 | 3.14E-02 |
| NM_176963 | Galm | 5.85E+01 | 2.50E+01 | -8.49E-01 | 3.69E-02 |
| NR_002891 | Gm5512 | 1.29E+01 | 5.53E+00 | -8.50E-01 | 2.96E-02 |
| NM_010072 | Dpm1 | 2.62E+01 | 1.12E+01 | -8.51E-01 | 3.00E-02 |
| NM_025736 | Ttc35 | 2.67E+01 | 1.14E+01 | -8.51E-01 | 3.84E-02 |
| NM_028102 | Ddhd2 | 8.51E+00 | 3.63E+00 | -8.53E-01 | 3.38E-02 |
| NM_008831 | Phb | 3.16E+01 | 1.35E+01 | -8.53E-01 | 2.95E-02 |
| NM_026310 | Mrpl18 | 4.38E+01 | 1.87E+01 | -8.53E-01 | 3.48E-02 |
| NM_023203 | Dctpp1 | 5.87E+01 | 2.50E+01 | -8.55E-01 | 3.99E-02 |
| NM_025409 | Ier3ip1 | 1.98E+01 | 8.42E+00 | -8.55E-01 | 4.20E-02 |
| NM_001162980 | 1700024P16Rik | 7.00E+00 | 2.97E+00 | -8.56E-01 | 4.55E-02 |
| NM_144791 | Tor1aip1 | 6.94E+00 | 2.95E+00 | -8.57E-01 | 3.76E-02 |
| NM_010178 | Srsf10 | 1.10E+01 | 4.68E+00 | -8.58E-01 | 3.20E-02 |
| NM_026690 | Aspdh | 5.93E+01 | 2.51E+01 | -8.60E-01 | 2.94E-02 |
| NM_001112668 | Gm9790 | 1.13E+02 | 4.77E+01 | -8.60E-01 | 3.90E-02 |
| NM_025791 | Tmem223 | 3.63E+01 | 1.53E+01 | -8.62E-01 | 4.54E-02 |
| NM_007861 | Dld | 9.25E+01 | 3.90E+01 | -8.63E-01 | 4.69E-02 |
| NM_212449 | AU019823 | 7.82E+00 | 3.30E+00 | -8.64E-01 | 3.96E-02 |
| NM_007574 | C1qc | 6.39E+01 | 2.69E+01 | -8.65E-01 | 2.73E-02 |
| NM_146013 | Sec14l4 | 5.18E+01 | 2.18E+01 | -8.66E-01 | 3.63E-02 |
| NM_183250 | Ccdc72 | 6.49E+01 | 2.73E+01 | -8.67E-01 | 3.51E-02 |
| NM_001130165 | Oxr1 | 7.96E+00 | 3.34E+00 | -8.68E-01 | 3.18E-02 |
| NM_013640 | Psmb10 | 3.32E+01 | 1.39E+01 | -8.69E-01 | 3.00E-02 |
| NM_025819 | 1200016B10Rik | 1.01E+01 | 4.23E+00 | -8.69E-01 | 3.02E-02 |
| NM_016763 | Hsd17b10 | 1.87E+02 | 7.82E+01 | -8.69E-01 | 3.35E-02 |
| NM_172677 | Ythdf3 | 1.08E+01 | 4.52E+00 | -8.72E-01 | 2.56E-02 |
| NM_009427 | Tob1 | 1.04E+02 | 4.35E+01 | -8.72E-01 | 4.92E-02 |
| NM_025933 | Higd2a | 1.11E+02 | 4.66E+01 | -8.73E-01 | 2.70E-02 |
| NM_018819 | Brp44l | 2.10E+02 | 8.79E+01 | -8.73E-01 | 3.51E-02 |
| NM_010493 | Icam1 | 3.01E+01 | 1.26E+01 | -8.74E-01 | 2.52E-02 |
| NM_025461 | Cox16 | 1.74E+01 | 7.22E+00 | -8.78E-01 | 3.07E-02 |
| NM_025598 | Ppdpf | 6.02E+01 | 2.50E+01 | -8.80E-01 | 2.81E-02 |
| NM_025578 | Mrps25 | 4.95E+00 | 2.05E+00 | -8.80E-01 | 3.15E-02 |
| NM_029092 | Rg9mtd1 | 2.08E+01 | 8.63E+00 | -8.81E-01 | 2.87E-02 |
| NM_001007570 | Slc25a42 | 1.14E+01 | 4.72E+00 | -8.81E-01 | 2.89E-02 |
| NM_016710 | Hmgn5 | 2.48E+01 | 1.03E+01 | -8.81E-01 | 2.62E-02 |
| NR_027838 | Atp2a2 | 9.79E+00 | 4.05E+00 | -8.81E-01 | 2.43E-02 |
| NM_008698 | Nipsnap1 | 1.13E+02 | 4.66E+01 | -8.83E-01 | 4.02E-02 |
| NM_001081490 | Fbxo9 | 2.35E+01 | 9.72E+00 | -8.83E-01 | 2.63E-02 |
| NM_001077237 | BC003331 | 1.67E+01 | 6.90E+00 | -8.85E-01 | 2.41E-02 |
| NM_025498 | Psenen | 5.71E+01 | 2.35E+01 | -8.85E-01 | 3.35E-02 |
| NM_010197 | Fgf1 | 2.74E+01 | 1.13E+01 | -8.87E-01 | 2.63E-02 |
| NM_025822 | Rsrc1 | 7.53E+00 | 3.09E+00 | -8.90E-01 | 3.54E-02 |
| NM_009202 | Slc22a1 | 1.25E+02 | 5.14E+01 | -8.90E-01 | 4.69E-02 |
| NM_008059 | G0s2 | 7.02E+01 | 2.88E+01 | -8.90E-01 | 2.38E-02 |
| NM_053161 | Mrpl27 | 3.16E+01 | 1.30E+01 | -8.91E-01 | 4.52E-02 |
| NM_027346 | Taco1 | 1.54E+01 | 6.33E+00 | -8.92E-01 | 4.28E-02 |
| NM_013721 | Rpl7a | 2.59E+01 | 1.06E+01 | -8.94E-01 | 4.34E-02 |
| NM_001037938 | Dhrs4 | 8.32E+01 | 3.40E+01 | -8.94E-01 | 2.45E-02 |
| NM_025966 | 2310039H08Rik | 5.81E+01 | 2.38E+01 | -8.94E-01 | 2.69E-02 |
| NM_029963 | Mrps5 | 1.98E+01 | 8.08E+00 | -8.95E-01 | 3.31E-02 |
| NM_007749 | Cox7c | 1.91E+02 | 7.82E+01 | -8.95E-01 | 2.30E-02 |
| NM_001080130 | Tmpo | 4.75E+00 | 1.94E+00 | -8.96E-01 | 4.71E-02 |
| NM_001131054 | Pttg1 | 3.62E+01 | 1.48E+01 | -8.97E-01 | 3.84E-02 |
| NM_174987 | 1810063B05Rik | 3.47E+01 | 1.41E+01 | -8.97E-01 | 3.84E-02 |
| NM_198004 | 5133401N09Rik | 1.10E+01 | 4.47E+00 | -8.98E-01 | 4.86E-02 |
| NR_003552 | Cyp2d13 | 7.48E+01 | 3.04E+01 | -8.99E-01 | 2.48E-02 |
| NM_024461 | 1810037I17Rik | 1.92E+01 | 7.81E+00 | -9.00E-01 | 4.73E-02 |
| NM_001080934 | Slc16a5 | 5.13E+00 | 2.08E+00 | -9.01E-01 | 4.73E-02 |
| NM_025849 | 3110001D03Rik | 4.11E+01 | 1.67E+01 | -9.02E-01 | 3.48E-02 |
| NM_001168535 | Cdadc1 | 6.88E+00 | 2.79E+00 | -9.04E-01 | 3.52E-02 |
| NM_013562 | Ifrd1 | 3.94E+01 | 1.59E+01 | -9.06E-01 | 2.10E-02 |
| NM_023672 | Ssbp3 | 5.08E+00 | 2.05E+00 | -9.07E-01 | 4.45E-02 |
| NM_001081223 | Rbbp8 | 5.10E+00 | 2.06E+00 | -9.07E-01 | 4.26E-02 |
| NM_177186 | Slc35e2 | 1.46E+01 | 5.88E+00 | -9.07E-01 | 2.18E-02 |
| NM_001164763 | Rarres1 | 3.43E+01 | 1.38E+01 | -9.09E-01 | 2.18E-02 |
| NM_011660 | Txn1 | 1.85E+02 | 7.43E+01 | -9.11E-01 | 2.83E-02 |
| NM_026467 | Rps27l | 5.75E+02 | 2.31E+02 | -9.13E-01 | 2.74E-02 |
| NM_011184 | Psma3 | 6.78E+01 | 2.72E+01 | -9.14E-01 | 2.04E-02 |
| NM_009777 | C1qb | 1.01E+02 | 4.05E+01 | -9.15E-01 | 2.03E-02 |
| NM_133739 | Tmem123 | 2.68E+01 | 1.07E+01 | -9.16E-01 | 1.93E-02 |
| NM_198414 | Paqr9 | 5.50E+01 | 2.20E+01 | -9.17E-01 | 2.29E-02 |
| NM_007572 | C1qa | 5.45E+01 | 2.18E+01 | -9.17E-01 | 2.00E-02 |
| NM_001081150 | Lonrf1 | 3.77E+00 | 1.51E+00 | -9.17E-01 | 4.64E-02 |
| NM_001145804 | Nucks1 | 1.50E+01 | 5.98E+00 | -9.18E-01 | 2.03E-02 |
| NM_145079 | Ugt1a6a | 4.79E+01 | 1.91E+01 | -9.20E-01 | 2.60E-02 |
| NM_011506 | Sucla2 | 8.10E+01 | 3.23E+01 | -9.21E-01 | 2.64E-02 |
| NM_019745 | Pdcd10 | 1.76E+01 | 6.99E+00 | -9.21E-01 | 2.48E-02 |
| NM_011055 | Pde3b | 2.70E+01 | 1.07E+01 | -9.22E-01 | 2.42E-02 |
| NM_019766 | Ptges3 | 8.80E+01 | 3.50E+01 | -9.23E-01 | 2.61E-02 |
| NM_029271 | Mrpl32 | 4.14E+01 | 1.65E+01 | -9.23E-01 | 2.83E-02 |
| NM_011920 | Abcg2 | 4.99E+01 | 1.98E+01 | -9.23E-01 | 2.23E-02 |
| NM_025366 | Chchd1 | 8.26E+01 | 3.28E+01 | -9.24E-01 | 2.23E-02 |
| NM_025685 | Col27a1 | 2.76E+00 | 1.10E+00 | -9.24E-01 | 3.16E-02 |
| NM_145478 | Pim3 | 2.08E+01 | 8.24E+00 | -9.24E-01 | 1.89E-02 |
| NM_011157 | Srgn | 2.74E+01 | 1.09E+01 | -9.26E-01 | 3.20E-02 |
| NM_010576 | Itga4 | 1.68E+00 | 6.65E-01 | -9.26E-01 | 3.86E-02 |
| NM_001085500 | Cisd3 | 5.08E+01 | 2.01E+01 | -9.27E-01 | 2.44E-02 |
| NM_198003 | Zfp946 | 7.48E+00 | 2.96E+00 | -9.27E-01 | 3.13E-02 |
| NM_053262 | Hsd17b11 | 1.09E+02 | 4.29E+01 | -9.27E-01 | 2.73E-02 |
| NM_001122738 | 1110028C15Rik | 3.16E+00 | 1.25E+00 | -9.29E-01 | 4.37E-02 |
| NM_011294 | Sub1 | 1.89E+01 | 7.45E+00 | -9.31E-01 | 1.71E-02 |
| NM_178704 | Dpy19l3 | 2.19E+00 | 8.62E-01 | -9.32E-01 | 3.75E-02 |
| NM_026612 | Ndufb2 | 1.02E+02 | 4.02E+01 | -9.32E-01 | 2.53E-02 |
| NM_001081982 | Nfix | 1.06E+01 | 4.16E+00 | -9.33E-01 | 2.43E-02 |
| NM_031878 | Smarcd2 | 1.54E+01 | 6.04E+00 | -9.34E-01 | 2.06E-02 |
| NM_008796 | Pctp | 4.84E+01 | 1.90E+01 | -9.35E-01 | 1.74E-02 |
| NM_013560 | Hspb1 | 5.11E+01 | 2.00E+01 | -9.36E-01 | 1.97E-02 |
| NM_019778 | Zbtb20 | 8.83E+00 | 3.46E+00 | -9.36E-01 | 2.47E-02 |
| NM_146106 | Lyplal1 | 1.61E+01 | 6.29E+00 | -9.37E-01 | 2.78E-02 |
| NM_008321 | Id3 | 5.53E+01 | 2.16E+01 | -9.38E-01 | 1.80E-02 |
| NM_001168538 | Cdadc1 | 6.73E+00 | 2.63E+00 | -9.38E-01 | 2.98E-02 |
| NM_025345 | 0610037P05Rik | 1.75E+01 | 6.82E+00 | -9.41E-01 | 2.80E-02 |
| NM_011169 | Prlr | 5.03E+00 | 1.96E+00 | -9.41E-01 | 1.62E-02 |
| NM_008879 | Lcp1 | 3.07E+01 | 1.20E+01 | -9.41E-01 | 1.85E-02 |
| NM_009942 | Cox5b | 2.40E+02 | 9.36E+01 | -9.42E-01 | 1.66E-02 |
| NR_015608 | 1810058I24Rik | 5.82E+01 | 2.27E+01 | -9.43E-01 | 1.66E-02 |
| NM_024177 | Mrpl38 | 6.61E+01 | 2.57E+01 | -9.45E-01 | 1.60E-02 |
| NM_146108 | Hibch | 5.32E+01 | 2.06E+01 | -9.47E-01 | 1.57E-02 |
| NR_027854 | Clk1 | 1.13E+01 | 4.38E+00 | -9.48E-01 | 1.91E-02 |
| NM_001113474 | Lair1 | 3.36E+00 | 1.30E+00 | -9.50E-01 | 4.75E-02 |
| NM_133943 | Hsd3b7 | 7.31E+01 | 2.82E+01 | -9.54E-01 | 1.79E-02 |
| NM_009387 | Tk1 | 1.19E+01 | 4.60E+00 | -9.54E-01 | 3.88E-02 |
| NM_011662 | Tyrobp | 7.66E+01 | 2.95E+01 | -9.56E-01 | 2.03E-02 |
| NM_001110163 | Pde4dip | 1.30E+00 | 4.99E-01 | -9.56E-01 | 4.87E-02 |
| NM_177670 | Tmem69 | 1.00E+01 | 3.84E+00 | -9.56E-01 | 2.50E-02 |
| NM_026149 | Nudcd1 | 5.68E+00 | 2.18E+00 | -9.58E-01 | 2.96E-02 |
| NM_025444 | Taf13 | 2.00E+01 | 7.66E+00 | -9.58E-01 | 2.50E-02 |
| NM_013706 | Cd52 | 4.19E+01 | 1.61E+01 | -9.60E-01 | 4.07E-02 |
| NM_145133 | Tifa | 9.24E+00 | 3.54E+00 | -9.60E-01 | 3.00E-02 |
| NM_153151 | Acat3 | 6.98E+01 | 2.67E+01 | -9.60E-01 | 1.74E-02 |
| NM_008823 | Cfp | 2.02E+01 | 7.71E+00 | -9.60E-01 | 2.09E-02 |
| NM_019403 | Rnf5 | 5.93E+01 | 2.27E+01 | -9.60E-01 | 1.40E-02 |
| NM_001122843 | Tnpo2 | 2.89E+00 | 1.10E+00 | -9.61E-01 | 3.89E-02 |
| NM_144896 | Pet112l | 1.08E+01 | 4.15E+00 | -9.62E-01 | 2.36E-02 |
| NM_001161419 | Atp5g1 | 1.29E+02 | 4.93E+01 | -9.62E-01 | 1.42E-02 |
| NM_007750 | Cox8a | 4.23E+02 | 1.62E+02 | -9.63E-01 | 1.87E-02 |
| NM_025832 | Naa16 | 4.25E+00 | 1.62E+00 | -9.63E-01 | 3.28E-02 |
| NM_027301 | Sdr9c7 | 1.11E+01 | 4.22E+00 | -9.64E-01 | 1.89E-02 |
| NM_024220 | Ndufc2 | 1.15E+02 | 4.37E+01 | -9.65E-01 | 1.37E-02 |
| NM_019552 | Abcb10 | 6.21E+00 | 2.36E+00 | -9.66E-01 | 2.09E-02 |
| NM_029475 | Adal | 7.20E+00 | 2.74E+00 | -9.66E-01 | 3.33E-02 |
| NM_001177975 | Irak1 | 4.82E+00 | 1.83E+00 | -9.67E-01 | 2.91E-02 |
| NM_012029 | Ecsit | 1.54E+01 | 5.85E+00 | -9.68E-01 | 3.62E-02 |
| NM_013826 | Mocs2 | 3.99E+01 | 1.51E+01 | -9.68E-01 | 1.29E-02 |
| NM_026988 | Ptms | 2.74E+02 | 1.04E+02 | -9.69E-01 | 3.61E-02 |
| NM_009169 | Shfm1 | 2.83E+02 | 1.07E+02 | -9.69E-01 | 1.36E-02 |
| NM_013623 | Orm3 | 3.29E+01 | 1.25E+01 | -9.69E-01 | 2.70E-02 |
| NM_007987 | Fas | 1.60E+01 | 6.07E+00 | -9.70E-01 | 2.42E-02 |
| NM_027175 | Ndufaf1 | 2.10E+01 | 7.97E+00 | -9.70E-01 | 2.00E-02 |
| NM_001081040 | Coq10a | 6.93E+00 | 2.62E+00 | -9.74E-01 | 3.11E-02 |
| NM_001109972 | Sec61g | 1.93E+01 | 7.30E+00 | -9.74E-01 | 4.44E-02 |
| NM_001163159 | Pcyt1a | 4.97E+00 | 1.88E+00 | -9.75E-01 | 2.11E-02 |
| NM_025597 | Ndufb3 | 1.04E+02 | 3.93E+01 | -9.75E-01 | 1.55E-02 |
| NM_026871 | Hint2 | 1.22E+02 | 4.59E+01 | -9.76E-01 | 1.33E-02 |
| NM_001111099 | Cdkn1a | 7.59E+00 | 2.85E+00 | -9.79E-01 | 3.63E-02 |
| NM_183358 | Gadd45gip1 | 2.34E+01 | 8.78E+00 | -9.79E-01 | 1.46E-02 |
| NM_010394 | H2-Q7 | 1.82E+01 | 6.81E+00 | -9.80E-01 | 2.03E-02 |
| NM_001102438 | Acbd5 | 3.30E+01 | 1.24E+01 | -9.80E-01 | 1.44E-02 |
| NM_023480 | Fahd1 | 2.78E+01 | 1.04E+01 | -9.81E-01 | 1.52E-02 |
| NM_009369 | Tgfbi | 2.18E+01 | 8.17E+00 | -9.83E-01 | 1.21E-02 |
| NM_021516 | Mark3 | 4.93E+00 | 1.84E+00 | -9.83E-01 | 2.97E-02 |
| NM_025939 | Paics | 8.71E+01 | 3.26E+01 | -9.85E-01 | 2.13E-02 |
| NM_010820 | Mpdz | 2.81E+00 | 1.05E+00 | -9.86E-01 | 2.23E-02 |
| NM_026742 | Ndufaf4 | 1.41E+01 | 5.25E+00 | -9.86E-01 | 1.20E-02 |
| NM_028595 | Ms4a6c | 6.72E+00 | 2.50E+00 | -9.87E-01 | 4.03E-02 |
| NM_013847 | Gcat | 2.03E+01 | 7.54E+00 | -9.88E-01 | 1.22E-02 |
| NM_001134717 | 2810006K23Rik | 6.55E+00 | 2.44E+00 | -9.89E-01 | 4.47E-02 |
| NM_001242419 | 4933426M11Rik | 5.82E+00 | 2.16E+00 | -9.89E-01 | 1.63E-02 |
| NM_001198955 | Gm7694 | 3.56E+00 | 1.32E+00 | -9.89E-01 | 3.47E-02 |
| NM_019567 | Acin1 | 9.88E+00 | 3.67E+00 | -9.89E-01 | 1.99E-02 |
| NM_007934 | Enpep | 1.86E+01 | 6.92E+00 | -9.89E-01 | 1.12E-02 |
| NM_029239 | Prkd3 | 1.00E+01 | 3.73E+00 | -9.90E-01 | 1.12E-02 |
| NM_001033156 | Fbxo33 | 1.01E+01 | 3.74E+00 | -9.91E-01 | 1.45E-02 |
| NM_021022 | Abcb11 | 7.20E+01 | 2.67E+01 | -9.91E-01 | 4.06E-02 |
| NM_009883 | Cebpb | 5.17E+01 | 1.92E+01 | -9.91E-01 | 1.10E-02 |
| NM_025590 | Acot11 | 2.35E+00 | 8.70E-01 | -9.91E-01 | 3.41E-02 |
| NM_001102437 | Acbd5 | 3.35E+01 | 1.24E+01 | -9.94E-01 | 1.31E-02 |
| NM_023172 | Ndufb9 | 4.17E+02 | 1.54E+02 | -9.94E-01 | 1.99E-02 |
| NM_020568 | Plin4 | 1.79E+00 | 6.62E-01 | -9.94E-01 | 4.32E-02 |
| NM_011343 | Sec61g | 1.71E+01 | 6.31E+00 | -9.95E-01 | 4.57E-02 |
| NM_001037170 | Tomm40l | 5.75E+00 | 2.12E+00 | -9.97E-01 | 2.78E-02 |
| NM_019688 | Rapgef4 | 1.88E+01 | 6.92E+00 | -9.97E-01 | 1.06E-02 |
| NM_021536 | Rhot1 | 5.76E+00 | 2.12E+00 | -9.97E-01 | 1.91E-02 |
| NM_010448 | Hnrnpab | 5.10E+01 | 1.88E+01 | -9.98E-01 | 1.25E-02 |
| NM_001177658 | Mrpl15 | 1.10E+01 | 4.05E+00 | -9.98E-01 | 1.15E-02 |
| NM_175428 | Zfp295 | 1.62E+00 | 5.97E-01 | -9.99E-01 | 4.46E-02 |
| NM_001013405 | D9Ertd402e | 1.23E+01 | 4.54E+00 | -9.99E-01 | 1.29E-02 |
| NM_001040112 | Arap1 | 3.29E+00 | 1.21E+00 | -1.00E+00 | 2.57E-02 |
| NM_019419 | Arl6ip1 | 8.94E+01 | 3.29E+01 | -1.00E+00 | 1.68E-02 |
| NM_025646 | Crls1 | 4.00E+01 | 1.47E+01 | -1.00E+00 | 1.04E-02 |
| NR_030720 | Gm8989 | 4.16E+00 | 1.52E+00 | -1.00E+00 | 3.21E-02 |
| NM_025430 | Mrpl35 | 7.51E+00 | 2.75E+00 | -1.00E+00 | 1.62E-02 |
| NM_001101475 | F830016B08Rik | 1.10E+01 | 4.03E+00 | -1.00E+00 | 1.31E-02 |
| NM_001048267 | Tnpo1 | 3.96E+00 | 1.45E+00 | -1.00E+00 | 2.00E-02 |
| NM_009803 | Nr1i3 | 2.67E+01 | 9.77E+00 | -1.01E+00 | 1.30E-02 |
| NM_145381 | Lactb2 | 1.67E+02 | 6.08E+01 | -1.01E+00 | 3.23E-02 |
| NM_020582 | Atp5j2 | 4.34E+02 | 1.58E+02 | -1.01E+00 | 1.17E-02 |
| NM_008156 | Gpld1 | 5.76E+01 | 2.10E+01 | -1.01E+00 | 2.45E-02 |
| NM_001122829 | Upf1 | 3.94E+00 | 1.43E+00 | -1.01E+00 | 2.29E-02 |
| NR_028143 | Lrrc28 | 9.69E+00 | 3.52E+00 | -1.01E+00 | 1.55E-02 |
| NM_009344 | Phlda1 | 5.98E+01 | 2.17E+01 | -1.01E+00 | 1.05E-02 |
| NM_020560 | Mrps31 | 2.80E+01 | 1.02E+01 | -1.01E+00 | 1.13E-02 |
| NM_026527 | Chac2 | 2.75E+01 | 9.99E+00 | -1.01E+00 | 1.31E-02 |
| NM_025547 | Mterfd1 | 1.56E+01 | 5.65E+00 | -1.01E+00 | 1.97E-02 |
| NR_027778 | Hgd | 7.66E+00 | 2.78E+00 | -1.01E+00 | 3.71E-02 |
| NM_018831 | Dclre1a | 6.65E+00 | 2.41E+00 | -1.01E+00 | 1.52E-02 |
| NM_178111 | Trp53inp2 | 4.78E+01 | 1.73E+01 | -1.02E+00 | 1.59E-02 |
| NM_030691 | Igsf6 | 4.69E+00 | 1.70E+00 | -1.02E+00 | 4.23E-02 |
| NM_001042760 | Slc22a18 | 1.20E+01 | 4.33E+00 | -1.02E+00 | 2.46E-02 |
| NM_011864 | Papss2 | 7.60E+01 | 2.75E+01 | -1.02E+00 | 2.38E-02 |
| NM_001085385 | 1600014C10Rik | 2.39E+01 | 8.65E+00 | -1.02E+00 | 9.02E-03 |
| NM_026428 | Dcxr | 8.62E+01 | 3.11E+01 | -1.02E+00 | 8.92E-03 |
| NM_021524 | Nampt | 4.13E+01 | 1.49E+01 | -1.02E+00 | 1.55E-02 |
| NM_001101598 | Isoc2a | 7.45E+01 | 2.68E+01 | -1.02E+00 | 9.02E-03 |
| NM_023871 | Set | 1.53E+01 | 5.51E+00 | -1.02E+00 | 1.04E-02 |
| NM_018815 | Nup210 | 2.23E+00 | 7.99E-01 | -1.02E+00 | 2.47E-02 |
| NM_026159 | Retsat | 7.83E+01 | 2.81E+01 | -1.02E+00 | 1.16E-02 |
| NM_011386 | Skil | 2.71E+00 | 9.73E-01 | -1.03E+00 | 2.12E-02 |
| NM_007819 | Cyp3a13 | 8.46E+01 | 3.03E+01 | -1.03E+00 | 2.01E-02 |
| NM_023737 | Ehhadh | 1.24E+02 | 4.46E+01 | -1.03E+00 | 3.44E-02 |
| NM_026102 | Daam1 | 4.74E+00 | 1.70E+00 | -1.03E+00 | 1.32E-02 |
| NM_001168660 | Apol9b | 2.24E+01 | 8.02E+00 | -1.03E+00 | 1.21E-02 |
| NM_027862 | Atp5h | 2.71E+02 | 9.70E+01 | -1.03E+00 | 9.48E-03 |
| NM_011030 | P4ha1 | 9.51E+00 | 3.40E+00 | -1.03E+00 | 1.02E-02 |
| NM_001135115 | Gm12250 | 3.20E+00 | 1.14E+00 | -1.03E+00 | 4.49E-02 |
| NM_028696 | Obfc2a | 1.82E+01 | 6.50E+00 | -1.03E+00 | 8.92E-03 |
| NM_026592 | B230118H07Rik | 7.18E+00 | 2.56E+00 | -1.03E+00 | 2.45E-02 |
| NM_008225 | Hcls1 | 9.43E+00 | 3.37E+00 | -1.03E+00 | 2.13E-02 |
| NM_172907 | Olfml1 | 1.23E+01 | 4.38E+00 | -1.03E+00 | 1.64E-02 |
| NM_026826 | Mrps18c | 6.12E+01 | 2.18E+01 | -1.03E+00 | 2.01E-02 |
| NM_175341 | Mbnl2 | 9.93E+00 | 3.54E+00 | -1.03E+00 | 9.14E-03 |
| NM_011297 | Rps24 | 2.25E+02 | 8.02E+01 | -1.03E+00 | 1.18E-02 |
| NM_007808 | Cycs | 4.09E+01 | 1.46E+01 | -1.03E+00 | 9.96E-03 |
| NM_013634 | Med1 | 3.20E+00 | 1.14E+00 | -1.03E+00 | 1.79E-02 |
| NM_173350 | Osbpl9 | 3.05E+01 | 1.08E+01 | -1.03E+00 | 8.35E-03 |
| NM_013837 | Tpst1 | 5.62E+00 | 1.99E+00 | -1.04E+00 | 3.73E-02 |
| NM_001017959 | Lamp2 | 2.57E+02 | 9.10E+01 | -1.04E+00 | 4.47E-02 |
| NM_010260 | Gbp2 | 1.17E+01 | 4.13E+00 | -1.04E+00 | 1.30E-02 |
| NM_001079694 | Srsf5 | 2.17E+01 | 7.67E+00 | -1.04E+00 | 1.18E-02 |
| NM_027816 | Cyp2u1 | 7.68E+00 | 2.72E+00 | -1.04E+00 | 2.15E-02 |
| NM_027296 | Trnt1 | 1.45E+01 | 5.12E+00 | -1.04E+00 | 1.14E-02 |
| NM_001177370 | Nfkb2 | 3.70E+00 | 1.31E+00 | -1.04E+00 | 3.40E-02 |
| NM_026982 | 1810027O10Rik | 1.39E+02 | 4.91E+01 | -1.04E+00 | 1.08E-02 |
| NM_001146001 | Pdzk1 | 7.89E+00 | 2.79E+00 | -1.04E+00 | 2.01E-02 |
| NM_001077510 | Gnas | 4.73E+01 | 1.67E+01 | -1.04E+00 | 7.60E-03 |
| NM_025511 | Fam36a | 9.03E+01 | 3.18E+01 | -1.04E+00 | 1.50E-02 |
| NM_025794 | Etfdh | 1.22E+02 | 4.31E+01 | -1.04E+00 | 2.01E-02 |
| NM_025574 | Pigy | 6.35E+00 | 2.24E+00 | -1.04E+00 | 1.01E-02 |
| NM_026442 | Cmc1 | 2.17E+01 | 7.65E+00 | -1.04E+00 | 1.54E-02 |
| NM_001111050 | Cd151 | 1.47E+01 | 5.16E+00 | -1.05E+00 | 1.53E-02 |
| NM_026309 | Lsm3 | 1.73E+01 | 6.10E+00 | -1.05E+00 | 4.49E-02 |
| NM_145466 | A2ld1 | 6.65E+01 | 2.34E+01 | -1.05E+00 | 7.42E-03 |
| NM_024431 | Morf4l1 | 5.28E+01 | 1.86E+01 | -1.05E+00 | 7.74E-03 |
| NM_153778 | Atoh8 | 4.42E+00 | 1.55E+00 | -1.05E+00 | 3.53E-02 |
| NM_145385 | Mlf2 | 5.97E+00 | 2.10E+00 | -1.05E+00 | 4.20E-02 |
| NM_026402 | Atg3 | 6.11E+01 | 2.14E+01 | -1.05E+00 | 8.25E-03 |
| NM_001134427 | Cdv3 | 1.37E+01 | 4.79E+00 | -1.05E+00 | 7.51E-03 |
| NM_001113478 | Frrs1 | 4.14E+00 | 1.45E+00 | -1.05E+00 | 3.43E-02 |
| NM_025884 | Zfp830 | 5.07E+00 | 1.78E+00 | -1.05E+00 | 2.11E-02 |
| NM_001177668 | Spna2 | 7.33E+00 | 2.55E+00 | -1.06E+00 | 6.98E-03 |
| NM_133691 | Puf60 | 1.19E+01 | 4.14E+00 | -1.06E+00 | 1.56E-02 |
| NM_025323 | 0610009D07Rik | 5.42E+01 | 1.88E+01 | -1.06E+00 | 7.05E-03 |
| NM_017372 | Lyz2 | 2.25E+02 | 7.84E+01 | -1.06E+00 | 1.32E-02 |
| NM_001197147 | Slc4a4 | 6.11E+00 | 2.12E+00 | -1.06E+00 | 7.47E-03 |
| NM_010680 | Lama3 | 7.87E-01 | 2.73E-01 | -1.06E+00 | 3.92E-02 |
| NM_011490 | Stau1 | 4.26E+00 | 1.48E+00 | -1.06E+00 | 2.71E-02 |
| NM_177101 | 4833442J19Rik | 2.57E+01 | 8.89E+00 | -1.06E+00 | 9.04E-03 |
| NM_009194 | Slc12a2 | 1.56E+00 | 5.42E-01 | -1.06E+00 | 3.24E-02 |
| NM_025379 | Cox7b | 2.76E+02 | 9.55E+01 | -1.06E+00 | 1.43E-02 |
| NM_001145820 | Gpd2 | 1.28E+00 | 4.42E-01 | -1.06E+00 | 4.32E-02 |
| NM_029869 | Zkscan1 | 1.36E+00 | 4.71E-01 | -1.06E+00 | 2.95E-02 |
| NM_009044 | Rel | 2.87E+00 | 9.93E-01 | -1.06E+00 | 4.46E-02 |
| NM_025628 | Cox6b1 | 6.88E+02 | 2.37E+02 | -1.07E+00 | 1.32E-02 |
| NM_001163454 | Prorsd1 | 5.94E+00 | 2.04E+00 | -1.07E+00 | 2.71E-02 |
| NM_026121 | Bag4 | 9.02E+00 | 3.10E+00 | -1.07E+00 | 7.25E-03 |
| NM_001127346 | Ndufaf2 | 3.03E+01 | 1.04E+01 | -1.07E+00 | 2.06E-02 |
| NM_001040684 | Hsd3b7 | 3.79E+01 | 1.31E+01 | -1.07E+00 | 6.10E-03 |
| NM_175103 | Bola2 | 1.07E+02 | 3.69E+01 | -1.07E+00 | 2.05E-02 |
| NM_013532 | Lilrb4 | 9.08E+00 | 3.12E+00 | -1.07E+00 | 2.78E-02 |
| NM_028941 | 4933407C03Rik | 2.71E+00 | 9.32E-01 | -1.07E+00 | 2.72E-02 |
| NM_198703 | Wnk1 | 2.92E+00 | 1.00E+00 | -1.07E+00 | 9.24E-03 |
| NM_172648 | Ifi205 | 4.54E+00 | 1.56E+00 | -1.07E+00 | 4.57E-02 |
| NM_023063 | Lima1 | 4.30E+00 | 1.47E+00 | -1.07E+00 | 1.75E-02 |
| NM_001033041 | Acmsd | 1.03E+01 | 3.53E+00 | -1.07E+00 | 1.28E-02 |
| NM_028521 | Phospho2 | 2.08E+01 | 7.12E+00 | -1.07E+00 | 7.13E-03 |
| NM_201360 | Cyp2d12 | 6.53E+01 | 2.23E+01 | -1.07E+00 | 6.17E-03 |
| NM_007768 | Crp | 2.60E+02 | 8.88E+01 | -1.08E+00 | 3.67E-02 |
| NM_001110267 | Vegfa | 6.91E+00 | 2.36E+00 | -1.08E+00 | 1.61E-02 |
| NM_025300 | Mrpl15 | 7.30E+00 | 2.49E+00 | -1.08E+00 | 2.30E-02 |
| NM_026756 | Nfic | 3.48E+00 | 1.19E+00 | -1.08E+00 | 1.30E-02 |
| NM_010442 | Hmox1 | 3.57E+01 | 1.22E+01 | -1.08E+00 | 6.06E-03 |
| NM_026424 | Coq10b | 3.26E+01 | 1.11E+01 | -1.08E+00 | 6.42E-03 |
| NM_172146 | Ppat | 1.47E+01 | 5.00E+00 | -1.08E+00 | 5.77E-03 |
| NM_013761 | Srr | 2.64E+01 | 8.99E+00 | -1.08E+00 | 5.89E-03 |
| NM_008898 | Por | 2.08E+02 | 7.05E+01 | -1.08E+00 | 4.83E-02 |
| NM_144549 | Trib1 | 3.42E+01 | 1.16E+01 | -1.08E+00 | 7.40E-03 |
| NM_008362 | Il1r1 | 1.48E+01 | 5.02E+00 | -1.08E+00 | 5.42E-03 |
| NR_037196 | Itch | 6.97E+00 | 2.36E+00 | -1.08E+00 | 7.13E-03 |
| NM_010185 | Fcer1g | 4.60E+01 | 1.56E+01 | -1.08E+00 | 1.27E-02 |
| NM_198052 | Tbx3 | 1.18E+00 | 3.99E-01 | -1.08E+00 | 4.97E-02 |
| NM_144520 | Sec14l2 | 1.76E+02 | 5.96E+01 | -1.08E+00 | 3.52E-02 |
| NM_007507 | Atp5k | 2.96E+02 | 1.00E+02 | -1.08E+00 | 8.85E-03 |
| NR_037987 | Gm16548 | 1.09E+01 | 3.70E+00 | -1.08E+00 | 2.51E-02 |
| NM_013902 | Fkbp3 | 4.49E+01 | 1.52E+01 | -1.09E+00 | 7.51E-03 |
| NM_030686 | Dhrs4 | 1.59E+01 | 5.37E+00 | -1.09E+00 | 2.38E-02 |
| NM_001037913 | Ccdc107 | 4.67E+01 | 1.58E+01 | -1.09E+00 | 8.57E-03 |
| NM_009156 | Sepw1 | 9.71E+01 | 3.27E+01 | -1.09E+00 | 5.58E-03 |
| NM_026332 | Dnajc19 | 4.40E+01 | 1.48E+01 | -1.09E+00 | 1.26E-02 |
| NM_146165 | Aimp2 | 9.26E+00 | 3.11E+00 | -1.09E+00 | 3.40E-02 |
| NM_001109905 | Stau1 | 8.93E+00 | 3.00E+00 | -1.09E+00 | 9.67E-03 |
| NM_001168655 | Socs2 | 3.57E+00 | 1.20E+00 | -1.09E+00 | 3.79E-02 |
| NM_025650 | Uqcr11 | 4.02E+02 | 1.34E+02 | -1.09E+00 | 5.18E-03 |
| NM_001102414 | Slc2a9 | 3.50E+00 | 1.17E+00 | -1.10E+00 | 2.22E-02 |
| NM_053263 | Hnrnpa3 | 7.68E+00 | 2.57E+00 | -1.10E+00 | 6.12E-03 |
| NM_013594 | Mbd1 | 2.16E+01 | 7.20E+00 | -1.10E+00 | 5.10E-03 |
| NM_007609 | Casp4 | 1.39E+01 | 4.64E+00 | -1.10E+00 | 1.46E-02 |
| NM_173876 | Clcn3 | 2.85E+00 | 9.50E-01 | -1.10E+00 | 1.76E-02 |
| NM_026038 | 2810055F11Rik | 1.47E+01 | 4.88E+00 | -1.10E+00 | 1.06E-02 |
| NM_009945 | Cox7a2 | 2.62E+02 | 8.70E+01 | -1.10E+00 | 6.92E-03 |
| NM_016665 | Stra13 | 5.10E+01 | 1.70E+01 | -1.10E+00 | 1.06E-02 |
| NM_009278 | Ssb | 3.54E+00 | 1.18E+00 | -1.10E+00 | 3.85E-02 |
| NM_001081278 | Tbc1d4 | 1.18E+00 | 3.92E-01 | -1.10E+00 | 3.45E-02 |
| NM_001159505 | Tnfsf13 | 6.44E+00 | 2.14E+00 | -1.10E+00 | 2.71E-02 |
| NM_027118 | Cdk13 | 3.13E+00 | 1.04E+00 | -1.10E+00 | 1.10E-02 |
| NM_001105160 | Cyp3a59 | 6.30E+01 | 2.09E+01 | -1.10E+00 | 5.42E-03 |
| NM_030046 | Dnajc21 | 1.13E+01 | 3.75E+00 | -1.10E+00 | 1.09E-02 |
| NM_013685 | Tcf4 | 1.82E+00 | 6.03E-01 | -1.11E+00 | 1.92E-02 |
| NM_001003717 | Osbpl8 | 4.52E+00 | 1.49E+00 | -1.11E+00 | 6.88E-03 |
| NM_025403 | Nop10 | 1.18E+02 | 3.88E+01 | -1.11E+00 | 4.62E-03 |
| NM_025844 | Chordc1 | 4.65E+01 | 1.53E+01 | -1.11E+00 | 4.64E-03 |
| NM_001004191 | Gm5617 | 2.33E+01 | 7.68E+00 | -1.11E+00 | 2.54E-02 |
| NM_011668 | Ube3a | 9.33E+00 | 3.07E+00 | -1.11E+00 | 4.88E-03 |
| NM_153408 | Neurl3 | 4.79E+00 | 1.58E+00 | -1.11E+00 | 2.13E-02 |
| NM_001159626 | Hagh | 1.19E+02 | 3.90E+01 | -1.11E+00 | 4.74E-03 |
| NR_028355 | Sra1 | 6.05E+00 | 1.98E+00 | -1.11E+00 | 4.70E-02 |
| NM_026323 | Wfdc2 | 1.28E+01 | 4.20E+00 | -1.12E+00 | 3.83E-02 |
| NR_024069 | 2010107H07Rik | 3.24E+01 | 1.06E+01 | -1.12E+00 | 3.83E-02 |
| NM_001122676 | Zcchc2 | 4.65E+00 | 1.52E+00 | -1.12E+00 | 7.90E-03 |
| NM_178413 | Thnsl2 | 1.03E+01 | 3.39E+00 | -1.12E+00 | 1.33E-02 |
| NM_008615 | Me1 | 9.05E+01 | 2.96E+01 | -1.12E+00 | 1.32E-02 |
| NM_001122897 | Hp1bp3 | 4.02E+00 | 1.31E+00 | -1.12E+00 | 1.81E-02 |
| NM_213729 | Inca1 | 8.62E+00 | 2.81E+00 | -1.12E+00 | 2.78E-02 |
| NM_021422 | Dnaja4 | 7.60E+00 | 2.47E+00 | -1.12E+00 | 9.55E-03 |
| NM_001128096 | Atp13a3 | 2.03E+01 | 6.60E+00 | -1.12E+00 | 5.60E-03 |
| NM_027209 | Ms4a6b | 3.72E+00 | 1.21E+00 | -1.12E+00 | 4.40E-02 |
| NM_001142655 | Arpp19 | 7.42E+00 | 2.41E+00 | -1.12E+00 | 6.83E-03 |
| NM_025881 | Luc7l | 4.25E+00 | 1.38E+00 | -1.13E+00 | 3.68E-02 |
| NM_010392 | H2-Q2 | 1.07E+01 | 3.45E+00 | -1.13E+00 | 2.39E-02 |
| NM_001033530 | AW146154 | 3.14E+00 | 1.02E+00 | -1.13E+00 | 2.26E-02 |
| NM_028747 | 0610012H03Rik | 5.21E+00 | 1.69E+00 | -1.13E+00 | 3.21E-02 |
| NM_001168540 | Tsku | 4.62E+00 | 1.49E+00 | -1.13E+00 | 2.03E-02 |
| NM_011951 | Mapk14 | 1.13E+01 | 3.65E+00 | -1.13E+00 | 4.83E-03 |
| NM_008168 | Grik5 | 1.46E+00 | 4.71E-01 | -1.13E+00 | 4.30E-02 |
| NR_033581 | Adh6-ps1 | 6.47E+00 | 2.08E+00 | -1.13E+00 | 1.23E-02 |
| NM_032400 | Sucnr1 | 9.45E+00 | 3.04E+00 | -1.13E+00 | 1.67E-02 |
| NM_025343 | Rmnd1 | 9.53E+00 | 3.06E+00 | -1.14E+00 | 1.32E-02 |
| NR_027875 | 1810058I24Rik | 7.54E+00 | 2.42E+00 | -1.14E+00 | 2.78E-02 |
| NM_030704 | Hspb8 | 1.82E+02 | 5.83E+01 | -1.14E+00 | 1.19E-02 |
| NM_001191004 | Lsm6 | 4.19E+00 | 1.34E+00 | -1.14E+00 | 1.35E-02 |
| NM_170786 | Cntf | 8.51E+00 | 2.73E+00 | -1.14E+00 | 2.55E-02 |
| NM_178055 | Dnajb2 | 4.19E+00 | 1.34E+00 | -1.14E+00 | 3.13E-02 |
| NM_178050 | Atl2 | 3.55E+00 | 1.14E+00 | -1.14E+00 | 1.91E-02 |
| NM_001206383 | Mavs | 5.59E+00 | 1.79E+00 | -1.14E+00 | 1.49E-02 |
| NM_194336 | Gbp6 | 7.23E+00 | 2.31E+00 | -1.14E+00 | 5.70E-03 |
| NM_009205 | Slc3a1 | 1.09E+01 | 3.49E+00 | -1.14E+00 | 7.75E-03 |
| NM_001163684 | Nosip | 3.81E+00 | 1.21E+00 | -1.14E+00 | 3.07E-02 |
| NM_001005506 | Txlna | 3.31E+00 | 1.05E+00 | -1.14E+00 | 1.28E-02 |
| NM_178747 | Gulo | 1.86E+02 | 5.90E+01 | -1.15E+00 | 1.93E-02 |
| NM_001098836 | Atxn7l3 | 3.38E+00 | 1.07E+00 | -1.15E+00 | 1.69E-02 |
| NM_026385 | Pllp | 4.74E+00 | 1.50E+00 | -1.15E+00 | 2.62E-02 |
| NM_028091 | Osgepl1 | 1.43E+01 | 4.51E+00 | -1.15E+00 | 5.22E-03 |
| NM_010876 | Ncf1 | 2.87E+00 | 9.06E-01 | -1.15E+00 | 2.94E-02 |
| NM_009999 | Cyp2b10 | 4.27E+00 | 1.35E+00 | -1.15E+00 | 2.90E-02 |
| NM_007446 | Amy1 | 1.62E+02 | 5.11E+01 | -1.15E+00 | 9.04E-03 |
| NM_009505 | Vegfa | 6.91E+00 | 2.18E+00 | -1.16E+00 | 7.38E-03 |
| NM_001110852 | Crem | 4.01E+00 | 1.26E+00 | -1.16E+00 | 4.30E-02 |
| NM_028894 | Lonrf3 | 1.70E+00 | 5.34E-01 | -1.16E+00 | 1.62E-02 |
| NM_028260 | Immp1l | 2.00E+01 | 6.29E+00 | -1.16E+00 | 1.23E-02 |
| NM_025572 | 2610528J11Rik | 8.02E+00 | 2.52E+00 | -1.16E+00 | 3.69E-02 |
| NM_025523 | Ndufc1 | 1.32E+02 | 4.14E+01 | -1.16E+00 | 5.39E-03 |
| NR_037589 | Rbm7 | 6.00E+00 | 1.88E+00 | -1.16E+00 | 1.87E-02 |
| NM_001161618 | Cul5 | 1.44E+00 | 4.50E-01 | -1.16E+00 | 2.43E-02 |
| NM_023420 | Col4a3bp | 5.53E+00 | 1.73E+00 | -1.16E+00 | 5.16E-03 |
| NM_028360 | Ttc19 | 3.30E+00 | 1.03E+00 | -1.16E+00 | 1.80E-02 |
| NM_001163239 | Nqo2 | 5.60E+00 | 1.74E+00 | -1.17E+00 | 8.01E-03 |
| NM_011905 | Tlr2 | 7.60E+00 | 2.37E+00 | -1.17E+00 | 7.97E-03 |
| NM_001139519 | Zbp1 | 1.45E+01 | 4.51E+00 | -1.17E+00 | 2.32E-02 |
| NM_001163618 | Tars2 | 3.00E+00 | 9.35E-01 | -1.17E+00 | 3.27E-02 |
| NM_001200038 | 1700021K19Rik | 1.74E+00 | 5.43E-01 | -1.17E+00 | 2.15E-02 |
| NM_025390 | Pop4 | 1.51E+01 | 4.68E+00 | -1.17E+00 | 1.13E-02 |
| NM_001164187 | Nagk | 4.69E+00 | 1.46E+00 | -1.17E+00 | 3.83E-02 |
| NM_021542 | Kcnk5 | 7.34E+00 | 2.27E+00 | -1.17E+00 | 6.13E-03 |
| NM_007712 | Clk2 | 3.71E+00 | 1.15E+00 | -1.17E+00 | 2.41E-02 |
| NM_001103181 | Tulp4 | 1.82E+00 | 5.61E-01 | -1.17E+00 | 1.07E-02 |
| NM_175443 | Etnk2 | 1.38E+02 | 4.26E+01 | -1.17E+00 | 9.37E-03 |
| NM_001204203 | Spp1 | 5.06E+00 | 1.56E+00 | -1.17E+00 | 3.21E-02 |
| NM_020621 | P2ry4 | 1.44E+00 | 4.43E-01 | -1.18E+00 | 3.24E-02 |
| NM_133671 | U2af2 | 1.18E+01 | 3.65E+00 | -1.18E+00 | 5.91E-03 |
| NM_201410 | Ugt1a6b | 1.50E+02 | 4.61E+01 | -1.18E+00 | 1.04E-02 |
| NM_007990 | Fau | 3.41E+01 | 1.05E+01 | -1.18E+00 | 1.34E-02 |
| NM_026928 | 1810014F10Rik | 2.98E+01 | 9.13E+00 | -1.18E+00 | 1.26E-02 |
| NM_001146084 | Fastkd5 | 4.32E+00 | 1.32E+00 | -1.18E+00 | 1.31E-02 |
| NM_145457 | Paip1 | 5.77E+00 | 1.76E+00 | -1.19E+00 | 5.01E-03 |
| NM_175641 | Ltbp4 | 9.25E-01 | 2.82E-01 | -1.19E+00 | 3.97E-02 |
| NM_133641 | Rtkn | 1.43E+00 | 4.36E-01 | -1.19E+00 | 4.55E-02 |
| NM_010776 | Mbl2 | 4.77E+02 | 1.45E+02 | -1.19E+00 | 1.60E-02 |
| NM_011353 | Serf1 | 1.87E+01 | 5.71E+00 | -1.19E+00 | 2.47E-02 |
| NM_009825 | Serpinh1 | 1.97E+00 | 5.98E-01 | -1.19E+00 | 4.39E-02 |
| NM_001085510 | Gm4987 | 3.39E+01 | 1.03E+01 | -1.19E+00 | 1.60E-02 |
| NM_013759 | Sepx1 | 4.25E+02 | 1.29E+02 | -1.19E+00 | 8.31E-03 |
| NM_001166429 | Hnrnpf | 4.45E+00 | 1.34E+00 | -1.20E+00 | 1.87E-02 |
| NM_001081372 | Ces1b | 1.73E+02 | 5.19E+01 | -1.20E+00 | 8.30E-03 |
| NR_029382 | Mir17hg | 1.78E+00 | 5.34E-01 | -1.20E+00 | 4.76E-02 |
| NM_025557 | Pcp4l1 | 4.16E+00 | 1.25E+00 | -1.20E+00 | 3.03E-02 |
| NM_001166593 | Eif5a | 1.69E+01 | 5.07E+00 | -1.20E+00 | 7.28E-03 |
| NM_174848 | Crybg3 | 2.01E+00 | 6.03E-01 | -1.21E+00 | 1.16E-02 |
| NM_001161430 | Nxt2 | 3.70E+00 | 1.10E+00 | -1.21E+00 | 1.97E-02 |
| NM_001024719 | Cyp2c67 | 1.78E+02 | 5.32E+01 | -1.21E+00 | 6.71E-03 |
| NM_016874 | Deaf1 | 3.56E+00 | 1.06E+00 | -1.21E+00 | 2.30E-02 |
| NM_007385 | Apoc4 | 2.07E+03 | 6.15E+02 | -1.21E+00 | 2.78E-02 |
| NM_153196 | Rbks | 1.57E+01 | 4.66E+00 | -1.22E+00 | 1.10E-02 |
| NM_028979 | Cyp2j9 | 4.59E+00 | 1.36E+00 | -1.22E+00 | 1.92E-02 |
| NM_145935 | Glyat | 2.30E+02 | 6.81E+01 | -1.22E+00 | 5.46E-03 |
| NR_040413 | 5033403H07Rik | 2.90E+01 | 8.58E+00 | -1.22E+00 | 1.92E-02 |
| NM_013558 | Hspa1l | 3.24E+00 | 9.52E-01 | -1.22E+00 | 2.15E-02 |
| NM_001128084 | Arhgap21 | 2.98E+00 | 8.75E-01 | -1.23E+00 | 5.39E-03 |
| NM_178803 | Aebp2 | 1.01E+00 | 2.96E-01 | -1.23E+00 | 2.71E-02 |
| NM_023685 | Zkscan3 | 6.15E+00 | 1.80E+00 | -1.23E+00 | 8.46E-03 |
| NR_015519 | AI662270 | 6.00E+00 | 1.76E+00 | -1.23E+00 | 2.11E-02 |
| NM_199062 | Zfp781 | 1.08E+00 | 3.16E-01 | -1.23E+00 | 1.44E-02 |
| NM_008869 | Pla2g4a | 1.58E+00 | 4.63E-01 | -1.23E+00 | 3.91E-02 |
| NM_013521 | Fpr1 | 3.74E+00 | 1.09E+00 | -1.23E+00 | 3.91E-02 |
| NM_001081268 | Prss53 | 2.59E+00 | 7.56E-01 | -1.23E+00 | 2.68E-02 |
| NM_001115151 | Uspl1 | 1.99E+00 | 5.80E-01 | -1.23E+00 | 2.68E-02 |
| NM_025465 | 1810029B16Rik | 7.74E+00 | 2.26E+00 | -1.23E+00 | 2.19E-02 |
| NM_133969 | Cyp4v3 | 1.94E+02 | 5.64E+01 | -1.23E+00 | 2.26E-02 |
| NM_146188 | Kctd15 | 2.41E+00 | 7.01E-01 | -1.23E+00 | 2.93E-02 |
| NM_029347 | Fggy | 1.07E+01 | 3.10E+00 | -1.23E+00 | 6.02E-03 |
| NM_001080746 | Gtf2i | 3.59E+00 | 1.04E+00 | -1.24E+00 | 7.61E-03 |
| NM_007626 | Cbx5 | 1.59E+00 | 4.62E-01 | -1.24E+00 | 8.95E-03 |
| NM_001110856 | Crem | 3.97E+00 | 1.15E+00 | -1.24E+00 | 1.29E-02 |
| NM_011361 | Sgk1 | 5.61E+00 | 1.62E+00 | -1.24E+00 | 9.60E-03 |
| NM_020256 | Zbtb33 | 2.71E+00 | 7.83E-01 | -1.24E+00 | 9.48E-03 |
| NM_001139520 | Samhd1 | 5.23E+00 | 1.50E+00 | -1.25E+00 | 4.88E-03 |
| NM_029509 | Gbp8 | 3.90E+00 | 1.12E+00 | -1.25E+00 | 1.51E-02 |
| NM_001037761 | Capzb | 5.29E+00 | 1.52E+00 | -1.25E+00 | 1.79E-02 |
| NM_001110338 | Gprc5c | 1.10E+01 | 3.17E+00 | -1.25E+00 | 5.93E-03 |
| NM_001163705 | Fbxo6 | 6.11E+00 | 1.75E+00 | -1.25E+00 | 2.37E-02 |
| NM_001110306 | Keap1 | 2.34E+00 | 6.70E-01 | -1.25E+00 | 1.85E-02 |
| NM_009767 | Chic1 | 4.75E-01 | 1.36E-01 | -1.25E+00 | 4.54E-02 |
| NM_133217 | Bco2 | 5.49E+00 | 1.57E+00 | -1.25E+00 | 1.21E-02 |
| NM_025658 | Ms4a4d | 3.62E+00 | 1.03E+00 | -1.25E+00 | 3.33E-02 |
| NM_025364 | Sarnp | 2.13E+01 | 6.08E+00 | -1.25E+00 | 5.52E-03 |
| NM_010545 | Cd74 | 3.36E+02 | 9.60E+01 | -1.25E+00 | 7.75E-03 |
| NM_001190830 | Jak3 | 1.08E+00 | 3.08E-01 | -1.25E+00 | 4.09E-02 |
| NM_001033223 | Lin7a | 1.78E+00 | 5.08E-01 | -1.25E+00 | 1.25E-02 |
| NM_181325 | Slc25a15 | 9.87E+01 | 2.81E+01 | -1.25E+00 | 6.13E-03 |
| NM_001198933 | Me1 | 3.66E+00 | 1.04E+00 | -1.25E+00 | 1.02E-02 |
| NM_001164793 | Grinl1a | 8.82E+00 | 2.51E+00 | -1.26E+00 | 5.52E-03 |
| NM_001168668 | Fam114a2 | 4.61E+00 | 1.31E+00 | -1.26E+00 | 1.03E-02 |
| NM_001093764 | Myadm | 1.67E+00 | 4.72E-01 | -1.26E+00 | 3.11E-02 |
| NM_026306 | Trmt112 | 6.94E+00 | 1.96E+00 | -1.26E+00 | 2.75E-02 |
| NM_145984 | Prepl | 2.18E+00 | 6.16E-01 | -1.27E+00 | 1.85E-02 |
| NM_001033213 | Ttc7b | 4.46E+00 | 1.26E+00 | -1.27E+00 | 6.92E-03 |
| NM_001177945 | 1810020D17Rik | 6.79E+00 | 1.91E+00 | -1.27E+00 | 3.05E-02 |
| NM_001177946 | 1810020D17Rik | 6.74E+00 | 1.90E+00 | -1.27E+00 | 3.60E-02 |
| NM_011796 | Capn10 | 2.34E+00 | 6.59E-01 | -1.27E+00 | 2.15E-02 |
| NM_001161724 | Ilk | 2.75E+00 | 7.74E-01 | -1.27E+00 | 3.17E-02 |
| NM_001177947 | 1810020D17Rik | 6.77E+00 | 1.90E+00 | -1.27E+00 | 4.35E-02 |
| NM_007843 | Defb1 | 6.94E+00 | 1.94E+00 | -1.27E+00 | 2.77E-02 |
| NM_025560 | 1810049H13Rik | 8.15E+00 | 2.28E+00 | -1.27E+00 | 2.77E-02 |
| NM_001197046 | Fgfr1op | 2.60E+00 | 7.26E-01 | -1.28E+00 | 1.58E-02 |
| NM_212444 | Gyk | 1.85E+00 | 5.15E-01 | -1.28E+00 | 1.52E-02 |
| NM_001205391 | Atg16l1 | 4.09E+00 | 1.14E+00 | -1.28E+00 | 8.53E-03 |
| NM_001159318 | Il1rap | 3.46E+00 | 9.64E-01 | -1.28E+00 | 6.79E-03 |
| NR_038030 | 4930562F07Rik | 9.84E+00 | 2.74E+00 | -1.28E+00 | 2.09E-02 |
| NM_025363 | 1110001J03Rik | 4.81E+01 | 1.34E+01 | -1.28E+00 | 9.10E-03 |
| NM_020559 | Alas1 | 2.48E+02 | 6.89E+01 | -1.28E+00 | 2.78E-02 |
| NM_007744 | Comt | 2.88E+00 | 8.01E-01 | -1.28E+00 | 2.34E-02 |
| NM_183251 | 1810020D17Rik | 6.80E+00 | 1.89E+00 | -1.28E+00 | 3.46E-02 |
| NM_001110851 | Crem | 4.03E+00 | 1.12E+00 | -1.28E+00 | 2.30E-02 |
| NM_001024846 | Zfp62 | 2.21E+00 | 6.14E-01 | -1.28E+00 | 1.34E-02 |
| NM_001033490 | Pusl1 | 3.95E+00 | 1.09E+00 | -1.28E+00 | 3.36E-02 |
| NM_011561 | Tdg | 1.42E+00 | 3.94E-01 | -1.28E+00 | 3.21E-02 |
| NM_001114879 | D14Abb1e | 1.24E+00 | 3.44E-01 | -1.29E+00 | 1.25E-02 |
| NM_001243307 | NA | 9.46E-01 | 2.61E-01 | -1.29E+00 | 3.53E-02 |
| NM_021372 | Sertad2 | 1.95E+00 | 5.34E-01 | -1.30E+00 | 9.36E-03 |
| NM_013744 | Zfp354b | 1.49E+00 | 4.09E-01 | -1.30E+00 | 3.91E-02 |
| NM_025325 | Haao | 3.48E+02 | 9.52E+01 | -1.30E+00 | 5.46E-03 |
| NM_183306 | Taok3 | 3.50E+00 | 9.57E-01 | -1.30E+00 | 5.97E-03 |
| NM_019545 | Hao2 | 2.27E+00 | 6.21E-01 | -1.30E+00 | 3.06E-02 |
| NM_138312 | Fam172a | 1.14E+00 | 3.11E-01 | -1.30E+00 | 2.89E-02 |
| NM_001164672 | Dnaja1 | 2.88E+00 | 7.83E-01 | -1.30E+00 | 5.31E-03 |
| NM_001177320 | Tfpi | 1.38E+00 | 3.76E-01 | -1.30E+00 | 3.31E-02 |
| NM_030000 | Cep110 | 1.65E+00 | 4.48E-01 | -1.30E+00 | 1.03E-02 |
| NM_027206 | Tnfaip8l2 | 3.93E+00 | 1.07E+00 | -1.30E+00 | 3.62E-02 |
| NM_145938 | Rpp40 | 2.25E+00 | 6.11E-01 | -1.30E+00 | 2.02E-02 |
| NM_011231 | Rabggtb | 1.22E+01 | 3.29E+00 | -1.31E+00 | 4.87E-03 |
| NM_025905 | Ttc23 | 2.65E+00 | 7.15E-01 | -1.31E+00 | 1.96E-02 |
| NM_001141927 | Pln | 6.66E+00 | 1.79E+00 | -1.31E+00 | 4.91E-03 |
| NM_007458 | Ap2a1 | 3.25E+00 | 8.72E-01 | -1.32E+00 | 8.61E-03 |
| NM_010656 | Sspn | 7.23E-01 | 1.94E-01 | -1.32E+00 | 4.12E-02 |
| NM_008484 | Lamb3 | 1.18E+00 | 3.17E-01 | -1.32E+00 | 2.56E-02 |
| NM_010071 | Dok2 | 3.01E+00 | 8.08E-01 | -1.32E+00 | 2.56E-02 |
| NM_026835 | Ms4a6d | 5.86E+00 | 1.57E+00 | -1.32E+00 | 1.49E-02 |
| NM_019467 | Aif1 | 1.13E+01 | 3.03E+00 | -1.32E+00 | 1.30E-02 |
| NM_001161365 | Rin3 | 2.84E+00 | 7.61E-01 | -1.32E+00 | 8.69E-03 |
| NM_146097 | Cbwd1 | 1.59E+00 | 4.24E-01 | -1.32E+00 | 4.57E-02 |
| NM_012006 | Acot1 | 8.91E+00 | 2.38E+00 | -1.32E+00 | 6.61E-03 |
| NM_001161329 | Nol11 | 2.21E+00 | 5.90E-01 | -1.32E+00 | 1.59E-02 |
| NM_001169131 | Papd7 | 2.41E+00 | 6.42E-01 | -1.32E+00 | 9.89E-03 |
| NM_001167829 | Fam45a | 4.89E+00 | 1.30E+00 | -1.33E+00 | 7.92E-03 |
| NM_001204335 | Cyp4f14 | 4.10E+00 | 1.09E+00 | -1.33E+00 | 1.14E-02 |
| NM_001029855 | Psme2 | 1.82E+01 | 4.83E+00 | -1.33E+00 | 7.96E-03 |
| NM_001034867 | Pm20d2 | 1.20E+00 | 3.19E-01 | -1.33E+00 | 1.52E-02 |
| NM_001110150 | Mgat1 | 4.07E+00 | 1.08E+00 | -1.33E+00 | 8.33E-03 |
| NM_001048168 | Nfyc | 5.53E+00 | 1.46E+00 | -1.33E+00 | 9.11E-03 |
| NM_011943 | Map2k6 | 2.66E+00 | 7.01E-01 | -1.33E+00 | 2.25E-02 |
| NM_001122683 | Bdh1 | 2.48E+00 | 6.55E-01 | -1.33E+00 | 1.30E-02 |
| NM_020266 | Dnajb2 | 3.58E+00 | 9.41E-01 | -1.34E+00 | 1.70E-02 |
| NR_033442 | Orc4 | 9.88E-01 | 2.59E-01 | -1.34E+00 | 2.93E-02 |
| NM_007679 | Cebpd | 5.09E+00 | 1.33E+00 | -1.34E+00 | 7.30E-03 |
| NM_001001322 | Adamts13 | 7.82E-01 | 2.05E-01 | -1.34E+00 | 3.40E-02 |
| NM_172050 | Cd300e | 3.92E+00 | 1.03E+00 | -1.34E+00 | 1.36E-02 |
| NM_177922 | Mapk15 | 1.98E+00 | 5.19E-01 | -1.34E+00 | 3.40E-02 |
| NM_015811 | Rgs1 | 6.08E+00 | 1.59E+00 | -1.34E+00 | 1.36E-02 |
| NM_001013022 | Odf3b | 4.09E+00 | 1.07E+00 | -1.34E+00 | 3.40E-02 |
| NM_010734 | Lst1 | 1.61E+01 | 4.22E+00 | -1.34E+00 | 3.39E-02 |
| NR_024487 | Uqcc | 4.16E+00 | 1.09E+00 | -1.34E+00 | 7.47E-03 |
| NM_021496 | Pvrl3 | 6.07E+00 | 1.58E+00 | -1.34E+00 | 7.25E-03 |
| NM_001164327 | Gm4902 | 2.74E+00 | 7.15E-01 | -1.34E+00 | 4.54E-02 |
| NM_001127356 | Eif2b4 | 3.74E+00 | 9.73E-01 | -1.35E+00 | 1.24E-02 |
| NM_001163485 | Rpl30 | 1.69E+01 | 4.39E+00 | -1.35E+00 | 1.88E-02 |
| NM_001015889 | Taf9 | 9.04E+00 | 2.35E+00 | -1.35E+00 | 8.62E-03 |
| NM_001081242 | Tln2 | 6.05E-01 | 1.57E-01 | -1.35E+00 | 1.20E-02 |
| NM_001127349 | Snx10 | 3.30E+00 | 8.54E-01 | -1.35E+00 | 1.15E-02 |
| NM_001166251 | Mgll | 3.55E+00 | 9.13E-01 | -1.36E+00 | 4.77E-03 |
| NR_037958 | 1300002E11Rik | 1.40E+01 | 3.60E+00 | -1.36E+00 | 1.75E-02 |
| NR_037617 | St5 | 1.69E+00 | 4.34E-01 | -1.36E+00 | 1.15E-02 |
| NM_001164087 | Homer2 | 8.51E-01 | 2.18E-01 | -1.36E+00 | 8.19E-03 |
| NM_001163552 | Ap4b1 | 3.72E+00 | 9.51E-01 | -1.36E+00 | 8.00E-03 |
| NM_016919 | Col5a3 | 1.68E+00 | 4.29E-01 | -1.36E+00 | 7.25E-03 |
| NM_146578 | Olfr1033 | 8.59E-01 | 2.19E-01 | -1.37E+00 | 3.51E-02 |
| NM_023476 | Tinagl1 | 3.87E+00 | 9.83E-01 | -1.37E+00 | 1.06E-02 |
| NM_026673 | Apoo | 1.58E+01 | 4.00E+00 | -1.37E+00 | 5.75E-03 |
| NM_053190 | S1pr5 | 1.83E+00 | 4.62E-01 | -1.37E+00 | 2.33E-02 |
| NM_027829 | Izumo4 | 5.69E+00 | 1.44E+00 | -1.37E+00 | 2.33E-02 |
| NM_001146086 | Fbxo34 | 4.33E+00 | 1.09E+00 | -1.38E+00 | 4.52E-03 |
| NR_027939 | 4930481A15Rik | 1.09E+01 | 2.72E+00 | -1.38E+00 | 1.50E-02 |
| NM_013498 | Crem | 3.99E+00 | 1.00E+00 | -1.38E+00 | 6.54E-03 |
| NM_153790 | Scarf2 | 7.36E-01 | 1.84E-01 | -1.39E+00 | 4.75E-02 |
| NM_026125 | Fam132a | 2.05E+00 | 5.13E-01 | -1.39E+00 | 4.75E-02 |
| NM_019919 | Ltbp1 | 3.42E-01 | 8.52E-02 | -1.39E+00 | 4.91E-02 |
| NR_028354 | Mvd | 3.42E+00 | 8.50E-01 | -1.39E+00 | 1.40E-02 |
| NM_001199187 | Ascc1 | 5.95E+00 | 1.48E+00 | -1.39E+00 | 9.24E-03 |
| NM_181282 | Bre | 3.42E+00 | 8.49E-01 | -1.39E+00 | 2.76E-02 |
| NM_025663 | Gpatch4 | 2.91E+00 | 7.20E-01 | -1.40E+00 | 1.91E-02 |
| NM_001080773 | Pdpk1 | 1.53E+00 | 3.79E-01 | -1.40E+00 | 4.26E-02 |
| NM_175446 | Zmat1 | 3.00E+00 | 7.41E-01 | -1.40E+00 | 6.06E-03 |
| NM_001198874 | Dync1i2 | 3.00E+00 | 7.40E-01 | -1.40E+00 | 9.81E-03 |
| NM_001039647 | Gbp11 | 3.12E+00 | 7.68E-01 | -1.40E+00 | 8.23E-03 |
| NR_038031 | 4930562F07Rik | 9.75E+00 | 2.39E+00 | -1.41E+00 | 1.46E-02 |
| NM_001163746 | Pgm3 | 9.88E-01 | 2.42E-01 | -1.41E+00 | 2.03E-02 |
| NM_001165254 | Ctage5 | 3.63E+00 | 8.84E-01 | -1.41E+00 | 6.00E-03 |
| NM_013547 | Hgd | 3.69E+02 | 8.95E+01 | -1.42E+00 | 8.45E-03 |
| NM_201642 | Ugt1a7c | 1.22E+00 | 2.97E-01 | -1.42E+00 | 2.24E-02 |
| NM_153140 | Rab11fip3 | 1.90E+00 | 4.62E-01 | -1.42E+00 | 1.22E-02 |
| NR_037153 | Chkb | 2.99E+00 | 7.25E-01 | -1.42E+00 | 1.48E-02 |
| NR_037581 | Magix | 1.26E+00 | 3.03E-01 | -1.42E+00 | 2.79E-02 |
| NM_025580 | Pnkd | 1.46E+01 | 3.51E+00 | -1.42E+00 | 1.06E-02 |
| NM_001172097 | 2700078E11Rik | 1.34E+00 | 3.21E-01 | -1.43E+00 | 8.60E-03 |
| NM_001166507 | Sec14l1 | 1.31E+00 | 3.14E-01 | -1.43E+00 | 1.36E-02 |
| NM_133648 | Slc12a6 | 1.52E+00 | 3.65E-01 | -1.43E+00 | 6.25E-03 |
| NM_175411 | Fam118b | 6.72E+00 | 1.61E+00 | -1.43E+00 | 5.23E-03 |
| NM_001164743 | Slc17a3 | 1.00E+00 | 2.39E-01 | -1.43E+00 | 2.00E-02 |
| NM_001162918 | Ccdc90b | 1.82E+00 | 4.35E-01 | -1.43E+00 | 4.04E-02 |
| NM_053200 | Ces1d | 5.04E+02 | 1.20E+02 | -1.43E+00 | 2.81E-02 |
| NM_177868 | Fhad1 | 8.43E-01 | 2.01E-01 | -1.43E+00 | 2.46E-02 |
| NM_008360 | Il18 | 1.55E+01 | 3.69E+00 | -1.43E+00 | 4.60E-03 |
| NM_009525 | Wnt5b | 1.68E+00 | 3.99E-01 | -1.44E+00 | 2.24E-02 |
| NM_001199940 | Serpina3i | 1.70E+00 | 4.04E-01 | -1.44E+00 | 2.45E-02 |
| NM_001142968 | BC024139 | 1.19E+00 | 2.81E-01 | -1.44E+00 | 3.00E-02 |
| NM_001105196 | Tcfe3 | 3.28E+00 | 7.74E-01 | -1.44E+00 | 4.96E-03 |
| NM_177270 | Cdkl2 | 1.33E+00 | 3.12E-01 | -1.44E+00 | 1.41E-02 |
| NM_153049 | Mkl1 | 2.56E+00 | 6.02E-01 | -1.45E+00 | 4.76E-03 |
| NM_028882 | Sema3d | 1.26E+00 | 2.97E-01 | -1.45E+00 | 6.34E-03 |
| NM_001159331 | N6amt1 | 2.17E+00 | 5.06E-01 | -1.46E+00 | 2.58E-02 |
| NM_001024512 | Fam149b | 3.18E+00 | 7.40E-01 | -1.46E+00 | 7.43E-03 |
| NM_152220 | Stx3 | 1.16E+00 | 2.69E-01 | -1.46E+00 | 2.11E-02 |
| NM_001170953 | Rnmt | 1.28E+00 | 2.97E-01 | -1.46E+00 | 9.88E-03 |
| NM_001029841 | Sla | 1.51E+00 | 3.49E-01 | -1.46E+00 | 1.80E-02 |
| NM_001168656 | Socs2 | 4.18E+00 | 9.65E-01 | -1.47E+00 | 5.83E-03 |
| NM_023326 | Bmyc | 7.75E+00 | 1.79E+00 | -1.47E+00 | 1.30E-02 |
| NM_001025371 | Serinc4 | 9.23E-01 | 2.13E-01 | -1.47E+00 | 4.17E-02 |
| NM_198942 | Dhx57 | 1.96E+00 | 4.48E-01 | -1.48E+00 | 1.49E-02 |
| NR_040343 | Gm15880 | 8.15E-01 | 1.86E-01 | -1.48E+00 | 3.90E-02 |
| NM_001177670 | Golph3l | 1.92E+00 | 4.36E-01 | -1.48E+00 | 1.73E-02 |
| NM_010259 | Gbp1 | 2.47E+00 | 5.61E-01 | -1.48E+00 | 7.69E-03 |
| NM_001134646 | Tomm5 | 1.28E+01 | 2.90E+00 | -1.48E+00 | 8.35E-03 |
| NM_178056 | Tm2d3 | 6.91E+00 | 1.57E+00 | -1.48E+00 | 1.03E-02 |
| NM_001168472 | Dynll2 | 2.56E+00 | 5.79E-01 | -1.49E+00 | 9.45E-03 |
| NM_001164683 | Tmem29 | 2.89E+00 | 6.50E-01 | -1.49E+00 | 1.94E-02 |
| NM_175153 | 2010321M09Rik | 1.92E+00 | 4.31E-01 | -1.50E+00 | 9.50E-03 |
| NM_001198866 | Dctn1 | 2.62E+00 | 5.87E-01 | -1.50E+00 | 3.95E-02 |
| NM_172724 | Baat1 | 9.47E-01 | 2.12E-01 | -1.50E+00 | 1.92E-02 |
| NM_001198968 | Itsn2 | 2.38E+00 | 5.32E-01 | -1.50E+00 | 2.97E-02 |
| NM_001243100 | Gm11837 | 3.41E+00 | 7.60E-01 | -1.50E+00 | 3.22E-02 |
| NM_001113514 | Itga9 | 1.55E+00 | 3.45E-01 | -1.50E+00 | 5.22E-03 |
| NM_001033908 | Med22 | 3.92E+00 | 8.71E-01 | -1.50E+00 | 1.25E-02 |
| NM_001199061 | Wdr12 | 1.85E+00 | 4.09E-01 | -1.51E+00 | 1.11E-02 |
| NM_181391 | Chchd7 | 7.80E+00 | 1.72E+00 | -1.51E+00 | 1.76E-02 |
| NM_027271 | D3Ertd751e | 1.08E+00 | 2.38E-01 | -1.51E+00 | 1.92E-02 |
| NR_033168 | Snora28 | 5.91E+02 | 1.30E+02 | -1.51E+00 | 2.89E-02 |
| NM_030693 | Atf5 | 4.53E+00 | 9.89E-01 | -1.52E+00 | 5.71E-03 |
| NM_001025612 | Snx22 | 1.94E+00 | 4.24E-01 | -1.52E+00 | 1.16E-02 |
| NM_001037757 | ORF19 | 2.54E+00 | 5.50E-01 | -1.53E+00 | 5.71E-03 |
| NM_011488 | Stat5a | 2.06E+00 | 4.46E-01 | -1.53E+00 | 4.70E-03 |
| NM_001177771 | Clcc1 | 1.96E+00 | 4.21E-01 | -1.54E+00 | 1.35E-02 |
| NM_001167944 | Zkscan5 | 1.58E+00 | 3.41E-01 | -1.54E+00 | 8.61E-03 |
| NM_009633 | Adra2b | 1.02E+00 | 2.18E-01 | -1.54E+00 | 1.43E-02 |
| NM_030203 | Tspyl4 | 1.13E+00 | 2.42E-01 | -1.54E+00 | 1.24E-02 |
| NM_172874 | Podn | 1.80E+00 | 3.85E-01 | -1.54E+00 | 8.50E-03 |
| NM_001243021 | Zfp939 | 2.14E+00 | 4.59E-01 | -1.54E+00 | 1.43E-02 |
| NM_008968 | Ptgis | 1.97E+00 | 4.21E-01 | -1.54E+00 | 1.98E-02 |
| NM_008677 | Ncf4 | 1.52E+00 | 3.26E-01 | -1.54E+00 | 4.13E-02 |
| NM_001048176 | Cerkl | 2.07E+00 | 4.44E-01 | -1.54E+00 | 1.84E-02 |
| NM_001170744 | Ctbp2 | 4.21E-01 | 8.98E-02 | -1.54E+00 | 4.74E-02 |
| NM_007940 | Ephx2 | 3.56E+02 | 7.55E+01 | -1.55E+00 | 5.26E-03 |
| NR_040355 | 1700029I01Rik | 8.10E-01 | 1.71E-01 | -1.55E+00 | 1.56E-02 |
| NM_001163388 | 2810002N01Rik | 6.43E+00 | 1.36E+00 | -1.55E+00 | 1.84E-02 |
| NM_001099785 | D3Ertd751e | 1.01E+00 | 2.13E-01 | -1.56E+00 | 1.70E-02 |
| NM_001110159 | Nxt1 | 3.23E+00 | 6.82E-01 | -1.56E+00 | 2.14E-02 |
| NM_134105 | Txndc11 | 5.22E+00 | 1.10E+00 | -1.56E+00 | 7.95E-03 |
| NM_198931 | Ppm1m | 2.26E+00 | 4.71E-01 | -1.57E+00 | 1.43E-02 |
| NR_040569 | 4930478L05Rik | 9.44E-01 | 1.96E-01 | -1.57E+00 | 4.93E-02 |
| NM_010006 | Cyp2d9 | 6.25E+02 | 1.29E+02 | -1.58E+00 | 1.13E-02 |
| NR_027505 | Rnf126 | 4.72E+00 | 9.71E-01 | -1.58E+00 | 6.11E-03 |
| NM_001163478 | Rabggtb | 4.26E+00 | 8.73E-01 | -1.59E+00 | 8.26E-03 |
| NM_001190322 | Chchd7 | 7.85E+00 | 1.61E+00 | -1.59E+00 | 1.52E-02 |
| NM_175219 | C130026I21Rik | 9.14E-01 | 1.86E-01 | -1.59E+00 | 4.53E-02 |
| NM_001164090 | Bcap29 | 7.11E+00 | 1.44E+00 | -1.60E+00 | 3.34E-02 |
| NM_198649 | Ablim3 | 1.45E+00 | 2.93E-01 | -1.60E+00 | 4.90E-03 |
| NM_144803 | Chrna2 | 1.83E+00 | 3.68E-01 | -1.60E+00 | 5.19E-03 |
| NM_016809 | Rbm3 | 2.15E+00 | 4.31E-01 | -1.61E+00 | 5.51E-03 |
| NR_040340 | Gm16157 | 4.93E+00 | 9.87E-01 | -1.61E+00 | 7.85E-03 |
| NM_001146224 | Dpy30 | 1.04E+01 | 2.06E+00 | -1.62E+00 | 6.12E-03 |
| NM_178793 | Ccbe1 | 8.92E-01 | 1.77E-01 | -1.62E+00 | 6.87E-03 |
| NM_183147 | Sprn | 5.73E-01 | 1.13E-01 | -1.63E+00 | 3.93E-02 |
| NM_007914 | Ehf | 1.09E+00 | 2.14E-01 | -1.63E+00 | 7.63E-03 |
| NM_025520 | Lsm5 | 1.53E+01 | 3.01E+00 | -1.63E+00 | 7.62E-03 |
| NM_001012324 | Ecm2 | 5.93E-01 | 1.16E-01 | -1.63E+00 | 3.00E-02 |
| NM_001164607 | Maf1 | 3.24E+00 | 6.34E-01 | -1.63E+00 | 6.55E-03 |
| NM_001081962 | Sap25 | 2.55E+00 | 4.98E-01 | -1.64E+00 | 2.59E-02 |
| NM_008624 | Mras | 1.09E+00 | 2.13E-01 | -1.64E+00 | 8.60E-03 |
| NM_027687 | Cabyr | 2.26E+00 | 4.40E-01 | -1.64E+00 | 7.98E-03 |
| NM_001038998 | Ccdc23 | 3.03E+00 | 5.90E-01 | -1.64E+00 | 3.29E-02 |
| NM_008294 | Hsd3b4 | 4.45E+00 | 8.60E-01 | -1.64E+00 | 1.21E-02 |
| NM_001111336 | Hsd3b4 | 4.46E+00 | 8.61E-01 | -1.64E+00 | 1.08E-02 |
| NM_001190327 | Tmsb10 | 6.03E+00 | 1.16E+00 | -1.64E+00 | 1.83E-02 |
| NM_001198841 | Csrp3 | 3.30E+00 | 6.36E-01 | -1.65E+00 | 2.33E-02 |
| NM_028667 | D3Ertd751e | 1.24E+00 | 2.39E-01 | -1.65E+00 | 8.22E-03 |
| NM_001111073 | Fxyd5 | 3.90E+00 | 7.49E-01 | -1.65E+00 | 1.03E-02 |
| NM_001101534 | Sult2a4 | 4.67E+00 | 8.93E-01 | -1.65E+00 | 1.09E-02 |
| NM_001166394 | 4931428F04Rik | 5.11E-01 | 9.77E-02 | -1.65E+00 | 4.39E-02 |
| NM_177628 | Fam167a | 8.49E-01 | 1.62E-01 | -1.66E+00 | 1.13E-02 |
| NM_027372 | Alkbh7 | 3.28E+00 | 6.23E-01 | -1.66E+00 | 3.11E-02 |
| NM_008039 | Fpr2 | 4.35E+00 | 8.21E-01 | -1.67E+00 | 6.10E-03 |
| NM_001037905 | Dab2 | 3.12E+00 | 5.86E-01 | -1.67E+00 | 2.39E-02 |
| NR_002898 | Snora65 | 4.52E+02 | 8.49E+01 | -1.67E+00 | 3.30E-02 |
| NM_001200023 | Zfp963 | 1.46E+00 | 2.73E-01 | -1.67E+00 | 1.29E-02 |
| NM_001204239 | Clec1b | 3.28E+00 | 6.14E-01 | -1.68E+00 | 1.45E-02 |
| NM_001159888 | Ociad1 | 4.13E+00 | 7.71E-01 | -1.68E+00 | 5.23E-03 |
| NR_030484 | Mir703 | 1.79E+03 | 3.33E+02 | -1.68E+00 | 5.20E-03 |
| NM_001004066 | Zfp386 | 2.52E+00 | 4.68E-01 | -1.68E+00 | 5.68E-03 |
| NM_009018 | Raet1c | 4.90E+00 | 9.08E-01 | -1.69E+00 | 4.97E-03 |
| NR_038073 | Snhg4 | 3.23E+00 | 5.98E-01 | -1.69E+00 | 1.21E-02 |
| NM_007880 | Arid3a | 5.20E-01 | 9.54E-02 | -1.69E+00 | 1.54E-02 |
| NM_015732 | Axin2 | 6.56E-01 | 1.21E-01 | -1.69E+00 | 1.54E-02 |
| NM_007645 | Cd37 | 2.60E+00 | 4.78E-01 | -1.69E+00 | 1.54E-02 |
| NM_001039188 | Rreb1 | 2.46E+00 | 4.52E-01 | -1.70E+00 | 1.33E-02 |
| NM_001164406 | Sra1 | 3.63E+00 | 6.62E-01 | -1.70E+00 | 1.81E-02 |
| NM_001162998 | 1110017F19Rik | 3.56E+00 | 6.47E-01 | -1.70E+00 | 1.42E-02 |
| NR_033554 | D330041H03Rik | 1.90E+00 | 3.44E-01 | -1.71E+00 | 1.74E-02 |
| NM_001205314 | Stat1 | 8.15E+00 | 1.48E+00 | -1.71E+00 | 6.26E-03 |
| NM_013896 | Timm9 | 3.70E+00 | 6.67E-01 | -1.71E+00 | 1.17E-02 |
| NM_019732 | Runx3 | 6.19E-01 | 1.10E-01 | -1.72E+00 | 1.90E-02 |
| NM_172951 | Sntg2 | 1.07E+00 | 1.91E-01 | -1.72E+00 | 1.90E-02 |
| NM_001163747 | Tmem192 | 3.84E+00 | 6.85E-01 | -1.72E+00 | 1.13E-02 |
| NM_011576 | Tfpi | 2.82E+00 | 5.03E-01 | -1.72E+00 | 7.96E-03 |
| NM_001080742 | Vamp5 | 1.83E+00 | 3.25E-01 | -1.73E+00 | 1.59E-02 |
| NM_001102670 | Kbtbd8 | 6.37E-01 | 1.13E-01 | -1.73E+00 | 1.33E-02 |
| NM_021327 | Tnip1 | 5.56E+00 | 9.73E-01 | -1.74E+00 | 1.23E-02 |
| NM_152821 | Purg | 1.36E+00 | 2.36E-01 | -1.76E+00 | 3.87E-02 |
| NM_001195565 | Camta1 | 1.48E+00 | 2.53E-01 | -1.76E+00 | 9.35E-03 |
| NM_177034 | Apba1 | 2.96E-01 | 5.07E-02 | -1.76E+00 | 2.42E-02 |
| NM_019656 | Tspan6 | 2.44E+00 | 4.18E-01 | -1.76E+00 | 6.27E-03 |
| NM_001004361 | 5730494M16Rik | 4.50E-01 | 7.62E-02 | -1.77E+00 | 2.60E-02 |
| NM_001113532 | Wtap | 4.18E+00 | 7.08E-01 | -1.78E+00 | 2.16E-02 |
| NM_013864 | Ndrg2 | 5.63E+02 | 9.49E+01 | -1.78E+00 | 6.12E-03 |
| NM_001113529 | Csf1 | 1.41E+00 | 2.37E-01 | -1.78E+00 | 4.57E-03 |
| NM_001001333 | Hexdc | 2.23E+00 | 3.74E-01 | -1.79E+00 | 4.53E-03 |
| NM_026601 | Hyi | 4.85E+00 | 8.11E-01 | -1.79E+00 | 6.80E-03 |
| NM_029286 | Ccdc30 | 1.47E+00 | 2.45E-01 | -1.79E+00 | 7.17E-03 |
| NM_001010930 | Mrps33 | 3.17E+00 | 5.27E-01 | -1.79E+00 | 1.45E-02 |
| NM_010954 | Ncam2 | 6.53E-01 | 1.07E-01 | -1.81E+00 | 8.10E-03 |
| NM_027014 | Gins1 | 2.76E+00 | 4.50E-01 | -1.81E+00 | 1.27E-02 |
| NM_001081120 | Fam89a | 2.74E+00 | 4.40E-01 | -1.83E+00 | 7.87E-03 |
| NM_001008702 | Dab2 | 3.14E+00 | 4.94E-01 | -1.85E+00 | 1.41E-02 |
| NR_002687 | Gm5424 | 1.37E+03 | 2.13E+02 | -1.86E+00 | 3.25E-02 |
| NM_001025074 | Ntrk2 | 2.95E-01 | 4.58E-02 | -1.86E+00 | 3.27E-02 |
| NM_001001496 | Gja6 | 1.31E+00 | 2.01E-01 | -1.87E+00 | 1.14E-02 |
| NR_015555 | 4933404O12Rik | 7.07E-01 | 1.08E-01 | -1.88E+00 | 9.21E-03 |
| NR_040642 | Mipep | 5.74E-01 | 8.65E-02 | -1.89E+00 | 1.61E-02 |
| NR_028571 | Snora17 | 2.19E+02 | 3.29E+01 | -1.90E+00 | 3.03E-02 |
| NR_027829 | Gm10638 | 1.97E+00 | 2.93E-01 | -1.90E+00 | 1.16E-02 |
| NR_027769 | Deaf1 | 1.49E+00 | 2.23E-01 | -1.90E+00 | 5.69E-03 |
| NM_173732 | Tmem194 | 1.13E+00 | 1.69E-01 | -1.90E+00 | 2.48E-02 |
| NM_001206391 | Hk3 | 9.13E-01 | 1.35E-01 | -1.91E+00 | 9.68E-03 |
| NM_011079 | Phkg1 | 1.06E+00 | 1.56E-01 | -1.91E+00 | 1.14E-02 |
| NR_027969 | 4930441O14Rik | 9.94E-01 | 1.46E-01 | -1.92E+00 | 7.73E-03 |
| NM_001163431 | 1810006K21Rik | 8.34E+00 | 1.21E+00 | -1.93E+00 | 1.37E-02 |
| NM_001163467 | Phtf1 | 1.92E+00 | 2.77E-01 | -1.94E+00 | 4.62E-02 |
| NM_173383 | Dnd1 | 1.32E+00 | 1.90E-01 | -1.94E+00 | 1.95E-02 |
| NM_080448 | Srgap3 | 2.63E-01 | 3.75E-02 | -1.95E+00 | 1.08E-02 |
| NM_001166067 | Slc4a5 | 2.32E-01 | 3.31E-02 | -1.95E+00 | 3.31E-02 |
| NR_030692 | A930011G23Rik | 3.62E-01 | 5.17E-02 | -1.95E+00 | 3.31E-02 |
| NM_177567 | BC049762 | 1.68E+00 | 2.40E-01 | -1.95E+00 | 3.30E-02 |
| NM_019500 | Cldn14 | 1.66E+00 | 2.33E-01 | -1.96E+00 | 1.22E-02 |
| NM_008442 | Kif2a | 1.31E+00 | 1.83E-01 | -1.97E+00 | 1.67E-02 |
| NM_198651 | 4430402I18Rik | 7.74E-01 | 1.07E-01 | -1.98E+00 | 2.07E-02 |
| NM_011090 | Lilra6 | 1.57E+00 | 2.16E-01 | -1.98E+00 | 3.63E-02 |
| NM_177752 | Eme1 | 7.03E-01 | 9.49E-02 | -2.00E+00 | 2.31E-02 |
| NM_172575 | Zfp277 | 1.80E+00 | 2.43E-01 | -2.00E+00 | 2.02E-02 |
| NR_027913 | G630025P09Rik | 1.46E+00 | 1.96E-01 | -2.01E+00 | 2.67E-02 |
| NM_001166372 | 1-Mar | 5.02E-01 | 6.75E-02 | -2.01E+00 | 1.15E-02 |
| NM_001081387 | Ctcfl | 1.01E+00 | 1.35E-01 | -2.01E+00 | 4.60E-03 |
| NM_001142744 | Atat1 | 9.41E-01 | 1.24E-01 | -2.02E+00 | 3.33E-02 |
| NR_033336 | Snora23 | 5.30E+01 | 6.98E+00 | -2.03E+00 | 3.59E-02 |
| NR_040619 | A930019D19Rik | 1.49E+00 | 1.95E-01 | -2.03E+00 | 3.61E-02 |
| NM_007538 | Opn1sw | 4.99E-01 | 6.52E-02 | -2.04E+00 | 3.66E-02 |
| NM_017394 | Slc7a10 | 7.26E-01 | 9.39E-02 | -2.05E+00 | 2.67E-02 |
| NM_030152 | Nol3 | 3.52E-01 | 4.49E-02 | -2.06E+00 | 4.89E-02 |
| NM_001025610 | Ms4a7 | 2.87E+00 | 3.57E-01 | -2.08E+00 | 1.04E-02 |
| NM_001146324 | Hps3 | 2.82E-01 | 3.50E-02 | -2.09E+00 | 8.55E-03 |
| NM_001205361 | Dcun1d1 | 4.98E+00 | 6.16E-01 | -2.09E+00 | 6.29E-03 |
| NM_001113208 | Ncam2 | 5.59E-01 | 6.91E-02 | -2.09E+00 | 1.07E-02 |
| NR_038097 | Gm11696 | 3.89E-01 | 4.79E-02 | -2.09E+00 | 4.18E-02 |
| NM_011935 | Esrrg | 5.26E-01 | 6.45E-02 | -2.10E+00 | 5.08E-03 |
| NM_178666 | Themis | 7.04E-01 | 8.63E-02 | -2.10E+00 | 5.08E-03 |
| NM_178399 | 3110035E14Rik | 5.30E-01 | 6.46E-02 | -2.10E+00 | 1.50E-02 |
| NM_175488 | Ccdc38 | 6.44E-01 | 7.69E-02 | -2.13E+00 | 6.93E-03 |
| NM_001163131 | Elf3 | 1.94E+00 | 2.30E-01 | -2.13E+00 | 3.11E-02 |
| NM_007921 | Elf3 | 1.94E+00 | 2.31E-01 | -2.13E+00 | 3.57E-02 |
| NM_008980 | Ptpra | 4.00E+00 | 4.74E-01 | -2.13E+00 | 6.03E-03 |
| NM_001130444 | Hras1 | 5.72E+00 | 6.68E-01 | -2.15E+00 | 2.65E-02 |
| NM_028439 | 3110009E18Rik | 1.29E+00 | 1.50E-01 | -2.15E+00 | 3.17E-02 |
| NR_040342 | A330093E20Rik | 6.98E-01 | 7.98E-02 | -2.17E+00 | 1.46E-02 |
| NR_037996 | Hmga2-ps1 | 1.33E+00 | 1.51E-01 | -2.17E+00 | 1.00E-02 |
| NR_033624 | 5031434O11Rik | 2.59E+00 | 2.86E-01 | -2.20E+00 | 2.02E-02 |
| NR_030423 | Mir671 | 6.33E+02 | 6.93E+01 | -2.21E+00 | 2.98E-02 |
| NR_015470 | 2610203C22Rik | 1.08E+00 | 1.18E-01 | -2.21E+00 | 3.07E-02 |
| NM_001013578 | Thoc7 | 4.26E+00 | 4.60E-01 | -2.23E+00 | 5.90E-03 |
| NM_177055 | A630001G21Rik | 8.78E-01 | 9.41E-02 | -2.23E+00 | 1.41E-02 |
| NM_001242943 | Gm14436 | 4.13E+00 | 4.40E-01 | -2.24E+00 | 3.15E-02 |
| NM_172655 | Hecw2 | 1.45E+00 | 1.47E-01 | -2.29E+00 | 1.85E-02 |
| NM_001142697 | 5730494M16Rik | 5.22E-01 | 5.27E-02 | -2.29E+00 | 6.77E-03 |
| NM_001110195 | Echdc1 | 1.66E-01 | 1.63E-02 | -2.33E+00 | 1.09E-02 |
| NM_008783 | Pbx1 | 5.42E-01 | 5.26E-02 | -2.33E+00 | 5.11E-03 |
| NR_027478 | Gm8580 | 3.50E+00 | 3.31E-01 | -2.36E+00 | 1.51E-02 |
| NM_001168488 | Men1 | 3.53E+00 | 3.31E-01 | -2.37E+00 | 7.48E-03 |
| NM_001159486 | Mcf2l | 2.50E-02 | 2.32E-03 | -2.38E+00 | 1.82E-02 |
| NR_027970 | 0610008F07Rik | 2.97E+00 | 2.74E-01 | -2.39E+00 | 5.90E-03 |
| NR_040749 | LOC622070 | 3.11E+00 | 2.72E-01 | -2.44E+00 | 5.09E-03 |
| NR_028481 | Snora31 | 6.20E+02 | 4.59E+01 | -2.60E+00 | 4.66E-03 |
| NM_001109989 | D0H4S114 | 7.01E+00 | 4.80E-01 | -2.68E+00 | 4.82E-02 |
| NM_010678 | Aff3 | 2.62E-01 | 1.40E-02 | -2.93E+00 | 1.11E-02 |
| NM_181045 | Caln1 | 1.16E+00 | 5.52E-02 | -3.04E+00 | 3.17E-02 |
| NM_001145920 | Runx2 | 3.21E-01 | 1.45E-02 | -3.09E+00 | 3.85E-02 |
| NM_001083957 | Car1 | 3.67E+00 | 1.65E-01 | -3.10E+00 | 1.21E-02 |
| NM_009799 | Car1 | 3.66E+00 | 1.65E-01 | -3.10E+00 | 1.14E-02 |
| NR_028115 | Lilra6 | 1.56E+00 | 6.84E-02 | -3.13E+00 | 6.13E-03 |
| NM_183126 | 6030498E09Rik | 1.94E+00 | 8.31E-02 | -3.15E+00 | 5.46E-03 |
| NM_001243261 | NA | 5.13E+00 | 2.20E-01 | -3.15E+00 | 5.46E-03 |
| NM_001167976 | Slc25a21 | 2.05E+00 | 7.47E-02 | -3.31E+00 | 1.39E-02 |
| NM_001110849 | Prr5l | 1.56E-01 | 3.37E-03 | -3.84E+00 | 1.97E-02 |
| NM_001163306 | Pxmp3 | 4.74E+00 | 9.77E-02 | -3.88E+00 | 1.66E-02 |
| NM_029945 | Smpd4 | 5.44E-01 | 9.20E-03 | -4.08E+00 | 6.68E-03 |
| NM_001110234 | Ngfrap1 | 8.97E-01 | 1.20E-02 | -4.31E+00 | 8.83E-03 |
| NM_001178012 | Sfxn3 | 2.40E-01 | 2.97E-03 | -4.39E+00 | 5.88E-03 |
| NM_001146200 | Pik3cg | 9.60E-02 | 8.99E-04 | -4.67E+00 | 4.78E-03 |
| NM_001128080 | Tspan32 | 2.25E-01 | 2.00E-03 | -4.73E+00 | 3.92E-02 |
| NM_001199333 | LOC100048884 | 7.25E+03 | 6.32E+01 | -4.74E+00 | 6.03E-03 |
| NM_001177366 | Fut7 | 2.42E-01 | 1.94E-03 | -4.83E+00 | 1.06E-02 |
| NM_001135127 | Mup19 | 8.25E+03 | 5.90E+01 | -4.94E+00 | 3.11E-02 |
| NM_001199999 | Mup14 | 7.87E+03 | 5.54E+01 | -4.96E+00 | 3.20E-02 |
| NM_175099 | Repin1 | 1.34E+00 | 9.12E-03 | -4.99E+00 | 7.47E-03 |
| NM_001200004 | Mup15 | 7.54E+03 | 4.76E+01 | -5.07E+00 | 1.03E-02 |
| NM_001166668 | Rtel1 | 3.42E-01 | 1.17E-03 | -5.68E+00 | 5.54E-03 |
| NM_201394 | Plec | 3.24E-01 | 2.24E-04 | -7.28E+00 | 4.35E-02 |
| NM_201386 | Plec | 3.64E-01 | 1.41E-04 | -7.86E+00 | 1.49E-02 |

Note: The mRNA levels of differently expressed genes between HCC of Ras-Tg (n=5) and liver tissues of wild-type mice (n=5). *P* values were calculated using Student’s *t*-test and *P*<0.05 means the significant difference. W: wild-type liver; T: hepatocellular carcinoma (HCC); Fold change: ln (T mean/W mean).
